# Diversity and Distribution Characteristics of Viruses from Soda Lakes

**DOI:** 10.3390/genes14020323

**Published:** 2023-01-26

**Authors:** Ramadan A. ZeinEldin, Marwa M. Ahmed, Wael S. Hassanein, Naglaa Elshafey, Ahmed R. Sofy, Hend A. Hamedo, Mohamed E. Elnosary

**Affiliations:** 1 Deanship of Scientific Research, King AbdulAziz University, Jeddah 21589, Saudi Arabia; 2 Faculty of Graduate Studies for Statistical Research, Cairo University, Giza 12613, Egypt; 3 Department of Electrical and Computer Engineering, Faculty of Engineering-Girls Campus, King Abdulaziz University, Jeddah 80204, Saudi Arabia; 4 Department of Industrial Engineering, Faculty of Engineering, King Abdulaziz University, Jeddah 80204, Saudi Arabia; 5 Botany and Microbiology Department, Faculty of Science, Arish University, Al-Arish 45511, Egypt; 6 Department of Botany and Microbiology, Faculty of Science, Al-Azhar University, Nasr City, Cairo 11884, Egypt

**Keywords:** viruses, viromes, brine, hypersaline, haloviruses, metagenomic

## Abstract

Viruses are the most abundant living things and a source of genetic variation. Despite recent research, we know little about their biodiversity and geographic distribution. We used different bioinformatics tools, MG-RAST, genome detective web tools, and GenomeVx, to describe the first metagenomic examination of haloviruses in Wadi Al-Natrun. The discovered viromes had remarkably different taxonomic compositions. Most sequences were derived from double-stranded DNA viruses, especially from *Myoviridae*, *Podoviridae*, *Siphoviridae*, *Herpesviridae*, *Bicaudaviridae,* and *Phycodnaviridae* families; single-stranded DNA viruses, especially from the family *Microviridae*; and positive-strand RNA viruses, especially from the family *Potyviridae*. Additionally, our results showed that Myohalovirus chaoS9 has eight Contigs and is annotated to 18 proteins as follows: tail sheath protein, tco, nep, five uncharacterized proteins, HCO, major capsid protein, putative pro head protease protein, putative head assembly protein, CxxC motive protein, terl, HTH domain protein, and terS Exon 2. Additionally, Halorubrum phage CGphi46 has 19 proteins in the brine sample as follows: portal protein, 17 hypothetical proteins, major capsid protein, etc. This study reveals viral lineages, suggesting the Virus’s global dispersal more than other microorganisms. Our study clarifies how viral communities are connected and how the global environment changes.

## 1. Introduction

Viruses have been referred to as the most major untapped store of biodiversity, since they are the most prevalent and smallest well-studied living organisms on Earth [1]. Viral cell lysis directly contributes to biogeochemical cycles, and over short time scales, viruses have the potential to cause catastrophic changes in community structures. Some viruses serve as predators in addition to offering an additional gene pool that may improve the fitness of their hosts [2]. Little is understood about viral population dynamics in natural systems, despite rising recognition of the ecological importance of viruses and the amount of viral genomic data available in public databases. Viruses and bacteria are the most prevalent biological species in hypersaline habitats, such as crystallizer ponds from solar salterns. Indeed, the greatest number of virus-like particles (VLP) are found in these ecosystems [3,4,5]. Giant Mimi viruses, cyanobacteria, aerobic and anaerobic archaea, prokaryotes, and eukaryotes are among the well-adapted extremophile life that predominates in soda lakes’ alkaline–saline aquatic habitats [6]. Saline lakes can be found all around the world. Saline lakes’ estimated total volume (10^4^ × 10^3^ km^3^) is comparable to freshwater lakes’ (124 × 10^3^ km^3^) [7] total volume. Saline alkaline lakes, often known as “soda lakes”, have salty waters with Na+ and carbonate species (HCO_3_^−^ and CO_3_^2−^) as the predominant ions. The pH of these waters is typically higher than 9. In addition to having large levels of Cl− and fluoride (F^−^) ions and aqueous silica, soda lake waters also frequently have varied levels of SO_4_^2−^ and potassium (K^+^), although they have very low levels of alkaline earths (Ca^2+^ and Mg^2+^). There are soda lakes globally [8,9,10,11,12,13,14]. Most soda lakes are only found in arid or semiarid subtropical latitudes in continental interiors or rain shadow zones, such as the East African Rift System (EARS), parts of California and Nevada, the Inner Mongolia Autonomous Region that extends to Tibet, and the Kulunda Steppe in South Siberia (Russia) [15].

Inhabitants of soda lakes must necessarily be alkaliphilic or at the very least, tolerable to alkalis. The word "alkaliphile" is typically used to describe microorganisms that thrive or develop particularly well at pH levels higher than 9, frequently with pH optima for growth around 10. These organisms typically exhibit minimal or no growth at pH levels close to neutral. Such species live in more diluted lakes, but “haloalkaliphiles”, which are typically found in hypersaline soda lakes such as Lake Magadi and Lake Natron, are widely believed to be alkaliphiles with the extra-obligate requirement for at least 1.5–2 M NaCl for optimum growth [16]. Moreover, according to [1], viruses—the bulk of which are bacteriophages—are the most widespread biological organisms in aquatic environments. Given the frequency with which dense blooms of prokaryotes have been observed, particularly Cyanobacteria and Haloarchaea, it is reasonable to assume that the viral population in soda lakes must play an important role in microbial interactions and development. However, despite a sizable body of research on hypersaline virioplankton, particularly haloarchaeal bacteriophages, in neutral hypersaline lakes, very little attention has been dedicated to the soda lake environment [17]. Phase partition, direct genome isolation by pulsed-field electrophoresis, and virus isolation by culturing are methods used on neutral hypersaline sites. 

Transmission electron microscopy and filtration concentration techniques have been used in population studies, and more recently, a metagenomic approach has been used to study these settings. According to Baxter et al. [17], virioplankton numbers ranged from 3 to 6 × 109 mL^−1^, while brine cell counts were closer to 6 × 107 mL^−1^. A wide variety of morphotypes, including some fusiform types of typical haloarchaeal host kinds, and others with typical head and tail phages, were also noted. Studies on African soda lakes are limited to looking at the variables that affect cyanobacteria and lesser flamingo population fluctuations (Phoeniconaias minor). A cyanobacterium that infects Arthrospira platensis in mass cultures was described by Jacquet et al. [18]. In other parts of the world, particularly Mono Lake in California, there has been a lot more research done on viral populations in soda lakes. To characterize some of the viruses using electron microscopy [13,19,20] examined the geographical and temporal variability of prokaryotes, viruses, and viral infections in the lake. In contrast to neutral hypersaline sites, distinct viral assemblages were observed in various layers of the lake, although fusiform viruses were not.

Wadi El-Natrun is a depression in northern Egypt, about 80 km northwest of Cairo. The lakes there have depths between 0.5 and 2 m. Its lakes have high salt concentrations between 91.0 and 393.9 g/L, an alkaline pH, and rising lake temperatures from solar action, making them extreme in more ways than one [21,22].

Numerous unique prokaryotic species, Archaea, and bacteria that can adapt to various stress conditions can be found abundantly in the lakes of Wadi El-Natrun. Additionally, there have been few virological investigations on these environments. Szab et al. [23] found bacteriophages, primarily Caudovirales, in the plankton of a murky pan, while Bell et al. identified Phycodnaviridae (algae-infecting viruses) in Lake Velence [24]. The Carpathian Basin’s water from some soda pans contained between 108 and 109 virus particles per milliliter [25]. The genetic diversity of the viral community can be better understood using metagenomics strategies, which have also been utilized to define several halophilic viral communities. These analyses, which relied on small fosmids or short reads, concluded that these communities were primarily uncharacterized and unique to these habitats [26,27]. According to Breitbart et al. [28], viral metagenomics is an approach that can provide a perspective on viral diversity. It has uncovered a significant amount of previously unrecognized diversity and an unanticipated richness of viral communities. This study aims to explore viral communities to better understand the diversity and abundance of viruses in brine and the functional annotating of these viruses. In addition, this investigation is the first study on Ga’ar Lake to report a viral community.

## 2. Materials and Methods

### 2.1. Sampling and High-Throughput Sequencing

**Figure 1:**
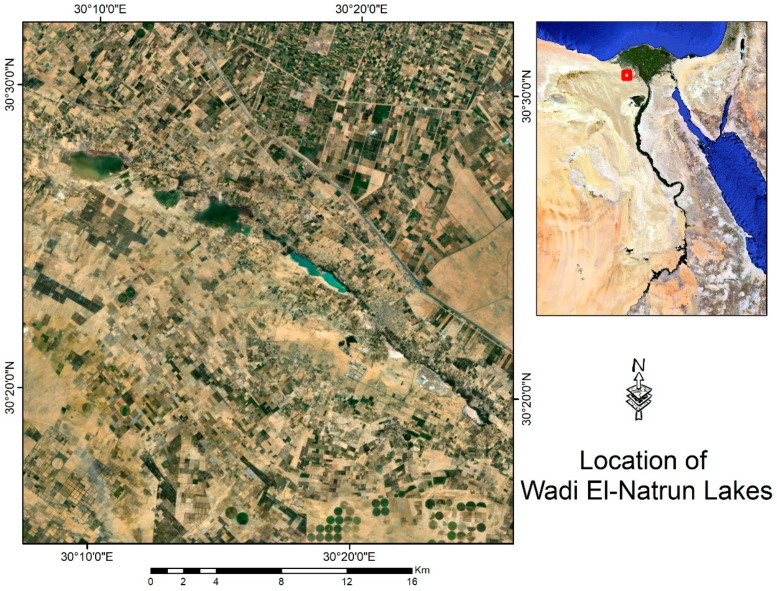
The sample-collection location (Egypt’s Wadi Al-Natrun from Soda Lake and Ga’ar Lake).

The brine samples were collected from Ga’ar Lake (30°27.222′ N, 30°10.83′ E) (Figure 1) and immediately stored at 4 °C upon arrival at the Suez Canal University for microbiological investigation. For DNA extraction, samples were immediately frozen at (−80 °C upon arrival at the biotechnology institute, in the faculty of agriculture, Suez Canal University [29]. Metagenomic DNA was extracted directly from the environmental sample, a frozen brine sample (−80 °C), using 15 mL of water (filtered through 0.2 μm membrane filters) according to Mesbah et al. (2007). The DNA sample of Lake Ga’ar brine was delivered to MacroGen Company www.dna.macrogen.com for library construction and sequencing following the Illumina Hiseq2000 manual’s instructions. The brine samples passed the QC results. Illumina HiSeq2000 generates raw images utilizing HCS (HiSeq Control Software v2.2, Macrogen company, Seoul, Republic of Korea) for system control and base calling through integrated primary analysis software RTA (Real Time Analysis. v1.18). The BCL (base calls) binary is converted into FASTQ utilizing the Illumina package bcl2fastq (v1.8.4) [30]. The taxonomic profile of this investigation showed that the main constituents of the brine sample were Archaea (81.7%), Eubacteria (18%), and viruses (0.5%).

### 2.2. Data Analysis

The Metagenomics Analysis Server (MG-RAST) [31], an automated annotation pipeline found online, was used to handle the acquired reads. This pipeline performs quality control, protein prediction, clustering, and similarity-based annotation on nucleic acid sequence datasets. MG-RAST (version 3.4-2020) generated the taxonomy analysis based on BLAST searches against the M5NR database and functional classifications based on BLAST searches against the SEED Subsystem database once the sequences were uploaded. Additionally, the default settings of SOAPdenovo [32] were used to construct the short reads. Moreover, the acquired reads were managed with the help of the Genome Detective server (http://www.genomedetective.com/app/typingtool/virus/ accessed on 1 December 2022) [33], an online automated annotation pipeline that performs quality control, protein prediction, clustering, and similarity-based annotation on nucleic acid sequence datasets. After the sequences were uploaded, the system produced Genome Detective accession numbers for each sample (Job ID e764ec65-0fd7-4320-931a-6b14a21d68b4).

Furthermore, using the protein-based alignment technique DIAMOND, Genome Detective performed taxonomy analyses [34]. The Advanced Genome Aligner (AGA), a new dynamic programming algorithm, was used to link the contigs for each species. It performs searches against the Swissprot UniRef90 protein database, and functional categories based on AGA are made to calculate the best global alignment while considering the alignment of every reference genome’s annotated coding sequence. Additionally, metaSPAdes [35] was also used to construct the short readings with default settings.

### 2.3. Phylogenetic Tree Building for Virus Classification

The VICTOR web tool uses the genomes of bacterial and archaeal viruses to compare them [36] (https://ggdc.dsmz.de/victor.php). The results include phylogenomic trees inferred using the Genome-BLAST Distance Phylogeny technique (GBDP), with branch support and recommendations for classifying the species, genus, subfamily, and family level.

### 2.4. Functional Annotation of the Halovirus of the Brine Sample

The first step in processing NGS short-read data is a de novo assembly pipe. Following trimmomatic preprocessing, reads can be sorted at the protein level and filtered using DIAMOND and the UniRef90 database and then assembled using the SPAdes (version 3.15.3 was released under GPLv2 on 22 July 2021) assembler. Consensus sequences are produced by the tool from the Genome Detective service assembled contigs. The web program GenomeVx [37] (http://wolfe.ucd.ie/GenomeVx/) was used to visualize assembled and annotated contigs on the reference genome. 

## 3. Results

### 3.1. Sequence Analysis of Brine Sample

**Figure 2:**
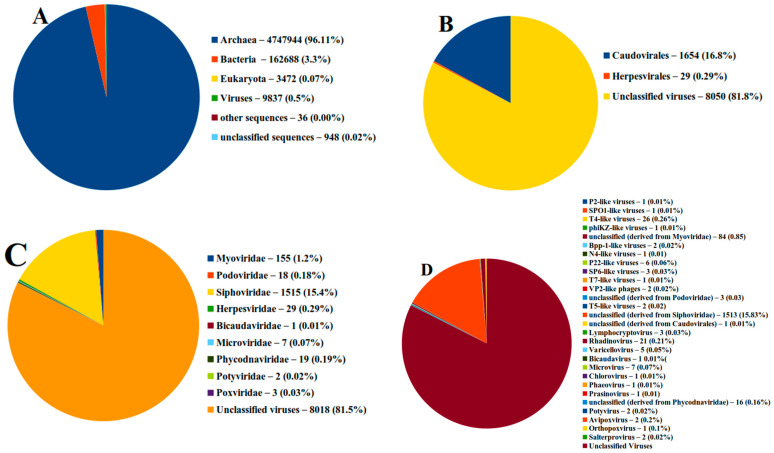
The distribution of viromes in brine sample according to (**A**) domain, (**B**) order, (**C**) family, (**D**) genus. The sequences were analyzed using MG-RAST.

The sample’s readings (19.98 million, 101 bp on average long) were uploaded to MG-RAST. A total of 0.23% of the sequences comprised rRNA genes, making up the most legitimate sequences through the quality control system. The distribution of the species-level annotations, which was 18 for the brine sample, was used to estimate the alpha diversity of the annotated samples. Then, MG-RAST was used to examine the taxonomic composition of the brine sample. Most of the sequences found indicated the presence of viruses in 9837 (0.5%), Eubacteria (3.3%), and Archaea (96.41%) (Figure 2A). MG-RAST was subsequently used to examine the viral taxonomic composition. The sample’s read was sent to the Genome Detective server for additional examination. An intuitive web-based program called Genome Detective swiftly and precisely assembled the genomes of viruses. By combining amino-acid and nucleotide scores, the application applies a novel alignment algorithm that builds genomes by reference-based linking of de novo contigs. The software was optimized using synthetic datasets to depict the wide variety of virus genomes accurately. The application was then validated using data from hundreds of viruses’ next-generation sequencing. Only the time necessary to upload the data requires any user time at all.

### 3.2. Taxonomic Diversity Analysis

The viral taxonomic distributions were as expected, and MG-RAST determined that 0.5% of the sequences were viral in origin. Figure 2 and Table 1 show that RNA and DNA viruses were present in the brine sample. Our findings demonstrated that the brine sample was divided into two orders (Caudovirales accounted for 16.8% of the total, Herpesvirales accounted for 0.29%, and unclassified viruses accounted for 82.91%). 

Since the taxonomy of viruses has undergone massive changes, the order Caudovirales has disappeared, and the families Myo-, Podo-, and Siphoviridae have disappeared. Despite the fact that the taxonomy of viruses has changed greatly, the databases are not updated frequently; thus, we stuck to the same nomenclature.

The MG-RAST results also revealed that numerous virus families, including Myoviridae, Podoviridae, Siphoviridae, Herpesviridae, Bicaudaviridae, Microviridae, Phycodnaviridae, and Potyviridae, were present in the brine samples. These virus families collectively accounted for 1.17, 0.18, 15.4, 0.29, 0.01, 0.07, and 0.19 percent (Figure 2C). The most common virus genus was T4-like viruses, which made up 0.26 percent of all viruses. Rhadinovirus (0.1 percent), Microvirus (0.07 percent), P22-like viruses (0.06 percent), and varicellovirus (0.1 percent) were the next most common virus genus (0.05 percent). 

Table 1 revealed that the brine sample under study has various types of viruses, including double strand DNA viruses (Myoviridae, Podoviridae, Siphoviridae, Bicaudaviridae, Phycodnaviridae, Myoviridae); Single-stranded DNA (Microviridae); and Positive-strand RNA viruses (Potyviridae), while all viruses family in Table 1 were not envolped except Herpesviridae.

All viral species were rapidly and precisely identified by the Genome Detective Virus Tool from the input sequence data. With a few exceptions, the viruses can be identified at the species level, and then they can be further examined using phylogenetic subtyping methods. Raw short-read NGS sequence data from FASTQ files or a FASTA file with sequences or contigs can be used as the input sequences from eukaryotic viruses, and phages are given taxonomy names by the Genome Detective Virus Tool. By constructing alignments that simultaneously optimize nucleotide and amino acid sequence similarity, the assignment is based on similarity to annotated whole genomes from the RefSeq database. Utilizing RefSeq (v. 214) sequences in conjunction with AGA ensures great sensitivity while ensuring high accuracy and speed. The taxonomic rank assigned is typically at the species level and depends on the availability of reference genomes in RefSeq. Based on 79,587 reference sequences, 11,140 unique taxonomy names are currently allocated. Our findings showed that The Genome Detective Virus Tool identified 18 viruses in the brine sample, including the following: *Myohalovirus ChaoS9*, *Halorubrum phage CGphi46*, *Archaeal BJ1 virus*, *Betapleolipovirus HRPV3*, *Myohalovirus PhiCh1*, *Halovirus HCTV-1*, *Halorubrum virus HRTV-29*, *Halovirus HGTV-1*, *Haloferax tailed virus 1*, *Betapleolipovirus HHPV4*, *Myohalovirus phiH*, *Haloarcula virus HCIV1*, *Halovirus HRTV-4*, *Alphapleolipovirus HRPV6*, *Betapleolipovirus HHPV4*, *Alphapleolipovirus HRPV2*, *Halovirus VNH-1*, and *Betapleolipovirus HRPV12* (Table 2 and Figure 2a).

In Table 2, the contigs number ranged from 2 to 41 while *Halovirus HGTV-1* has higher contigs with a depth coverage of 13.5, while *both Halovirus VNH-1* and *Betapleolipovirus HRPV12* have only 2 contigs with a depth coverage of 21.4 and 4.6, respectively.

**Figure 3:**
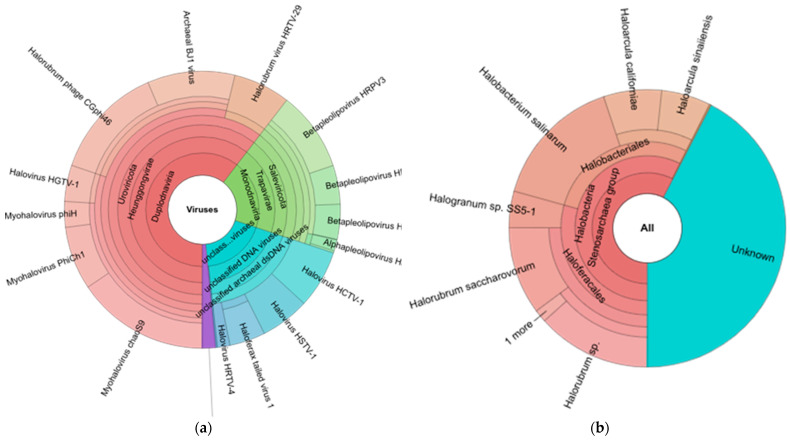
(**a**) Halo virus species and (**b**) Microorganism host of halo virus in brine sample detected by Genome Detective server.

The Genome Detective Virus Tool also identified eight viruses’ hosts in the brine sample, including the following: *Halobacterium salinarum*, *Halorubrum* sp., *Halorubrum saccharovorum*, *Haloarcula californiae*, *Haloarcula sinaiiensis*, *Halogranum* sp. *SS5-1*, *Halorubrum* sp. *S5a-3*, and *Nanohaloarchaea archaeon*, while 10 viruses’ hosts cannot be identified (Table 2 and Figure 3b).

### 3.3. Results of Phylogenetic Tree Building for Virus Classification

**Figure 4:**
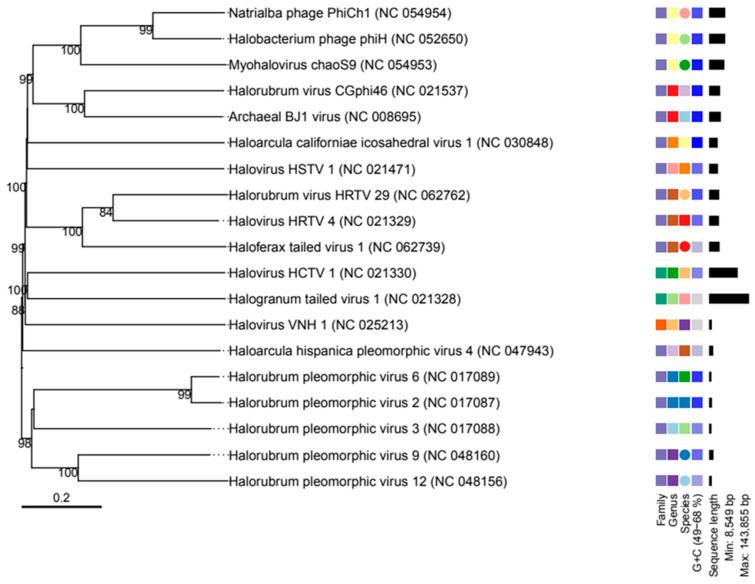
The phylogenetic tree compares archaeal viruses using their genome. The results include phylogenomic trees inferred using the Genome-BLAST Distance Phylogeny method (GBDP), with branch support, as well as suggestions for classifying the species, genus, subfamily, and family level.

The VICTOR web tool compares viruses from bacteria and archaea by analyzing their genomes. The outcomes include phylogenomic trees that demonstrated differences between the sequences (genomes) found by the Genome Detective server. Our findings demonstrated that, compared to other viruses, the families of the haloviruses HCTV 1, Halogrnum tailed virus, and VNH 1 differ in phylogenomic trees and genus and species. The phylogenomic trees showed that the virus’s genome had a GC content that ranged from 49 to 68% and that the length of the sequences was between 8549 and 143,855 (Figure 4).

### 3.4. Functional Annotation of Metavirome Contigs Discovered in Brine Sample

**Figure 5:**
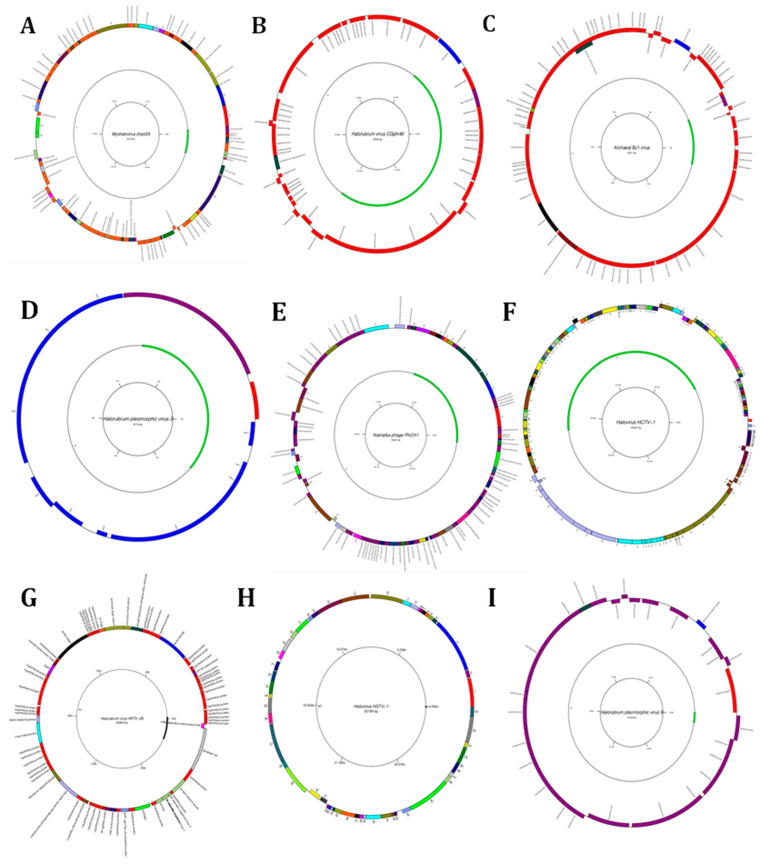

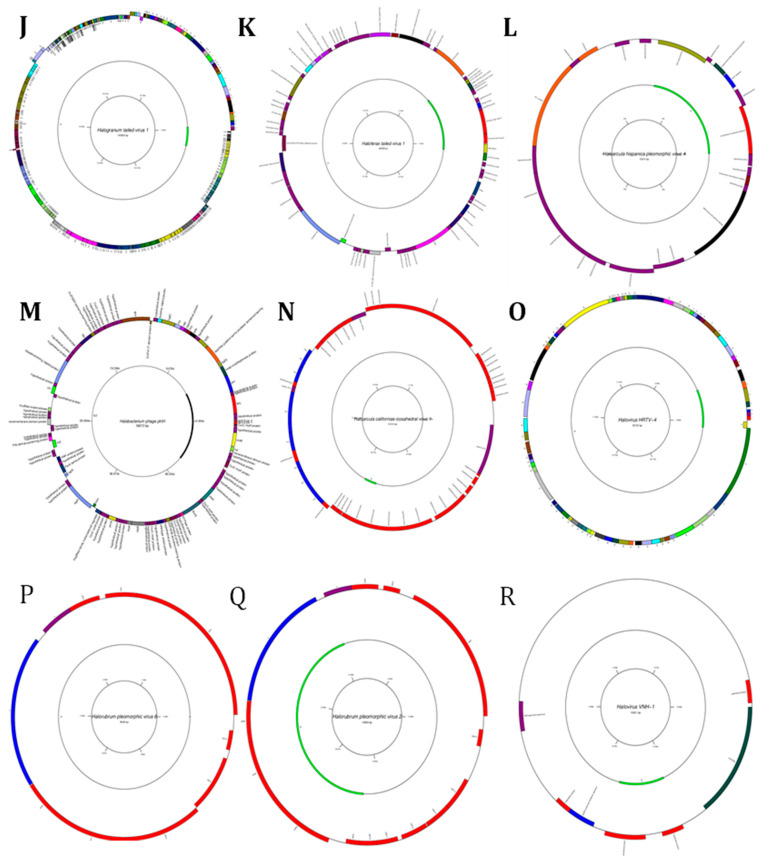

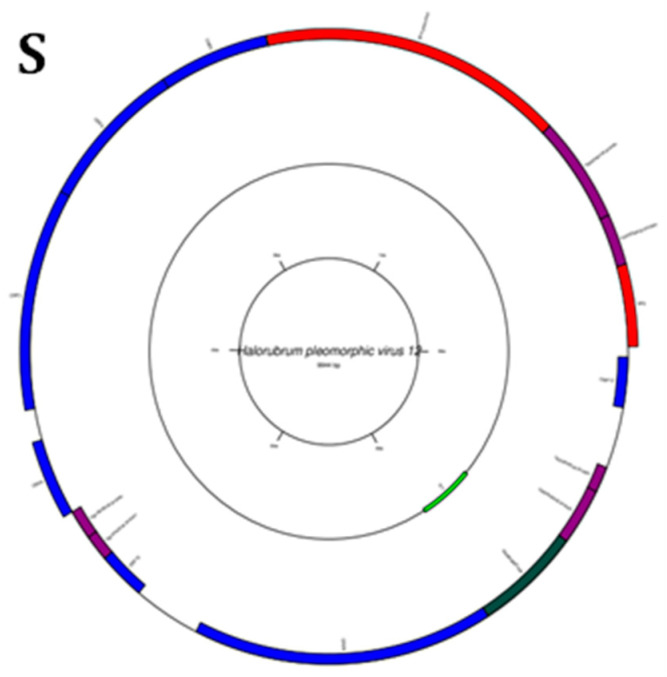
Diagrams of circular reference genome (outer circle) of metavirus, where the green segment represents query virus (s inner circle): (**A**) Myohalovirus chaoS9, (**B**) Halorubrum phage Cgphi46, (**C**) Archaeal BJ1 virus, (**D**) Betapleolipovirus HRPV3, (**E**) Myohalovirus PhiCh1, (**F**) Halovirus HCTV-1, (**G**) Halorubrum virus HRTV-29, (**H**) Halovirus HSTV-1, (**I**) Betapleolipovirus HRPV9, (**J**) Halovirus HGTV-1, (**K**) Haloferax tailed virus 1, (**L**) Betapleolipovirus HHPV4, (**M**) Myohalovirus phiH, (**N**) Haloarcula virus HCIV1, (**O**) Halovirus HRTV-4, (**P**) Alphapleolipovirus HRPV6, (**Q**) Alphapleolipovirus HRPV2, (**R**) Halovirus VNH-1, and (**S**) Betapleolipovirus HRPV12.

The typical method to annotate and characterize the function of a protein involves comparing the amino acid sequence of a protein to all functionally related sequences in the databases. Our findings revealed that the Myohalovirus chaoS9 has eight contigs in the brine sample, with a nucleotide that starts at 11,822 and ends at 57,470, and is annotated to eight proteins as follows: tail sheath protein, tco, nep, five uncharacterized proteins, HCO, major capsid protein, putative pro head protease protein, putative head assembly protein, CxxC motive protein, por, terl, and the HTH domain (Figure 5A). *Halorubrum phage CGphi46* has nineteen contigs in the brine sample, and is annotated to several proteins as follows: seventeen hypothetical proteins, one major capsid protein, etc. (Figure 5B). The Archaeal BJ1 virus has twenty-four contigs in the brine sample, and is annotated to seven hypothetical proteins (Figure 5C). The *Betapleolipovirus HRPV3* has four contigs in the brine sample, and is annotated to several proteins as follows: ORF10, ORF11 ORF12, etc. (Figure 5D). The functional annotation of the *Myohalovirus PhiCh1*, *Halovirus HCTV-1*, *Halorubrum virus HRTV-29*, *Halovirus HSTV-1*, *Betapleolipovirus HRPV9*, *Halovirus HGTV-1*, *Haloferax tailed virus 1*, *Betapleolipovirus HHPV4*, *Myohalovirus phiH*, *Haloarcula virus HCIV1*, *Halovirus HRTV-4*, *Alphapleolipovirus HRPV6*, *Alphapleolipovirus HRPV2*, *Halovirus VNH-1*, and *Betapleolipovirus HRPV12* is displayed in Table 2 and Table S1 and Figure 5E–S.

## 4. Discussion

Organisms can be conveniently grouped into three domains: Archaea, Bacteria, and Eukaryotes [38]. The three domains are joined by several features that support a common origin of life, such as the presence of ribosomes, double-stranded DNA genomes, and near-universal DNA genomes. By comparison, other types of genetic material and particles (such as viruses, plasmids, and other selfish genetic elements) are often excluded from the definition of “life” (for opposing views, see Raoult and Forterre) [39]. However, they can still influence the evolution of cellular organisms, and together they establish a complex life cycle.

Viruses impact the economy, health care, and agriculture due to their infectious nature. Viral infection converts the host cell into a virocell that no longer divides by binary fission but produces more virus particles, or a riboviro cell in which the genomes of the virus and the cell coexist, the cell still dividing while producing virions [26]. Birospheres (that is, collections of all viruses) exhibit exceptional variability in virion morphology and replication strategy. Viruses can be classified as DNA or RNA viruses, retroviruses, or intermediate types, depending on the type of replicon present within the virus particle. Furthermore, replicons can be linear, circular, single-stranded, double-stranded, and even segmented. The unprecedented diversity of replicon types has led to the proposal that viruses originally invented DNA as a means of tricking host defense systems [26]. Viruses can also transfer genes between species and increase biodiversity [40].

The distribution of viral lineages follows ancient, highly dynamic, and ongoing processes that influence the evolution of organisms. In many cases, new virus lineages arise from pre-existing lineages and can transcend species barriers to infect new hosts (e.g., parvoviruses; Shackelton et al. [41]), giving cellular organisms. It adds evolutionary pressure and encourages the development of molecular and cellular innovations [42]. This is between both simplicity and complexity.

Metagenomics has opened new possibilities that have advanced virology; other parameters, such as physical and chemical qualities, impact the diversity, abundance, and reproduction strategy of viruses in brine. By employing a meta-genomics technique and data analysis with MG-RAST and the Genome Detective service, the number of viruses in brine samples was measured to improve our understanding of the spread of brine viruses. According to our data, the brine sample contained both DNA and RNA viruses. Mg-rust results also showed that the brine sample belonged to the orders Caudovirales and Herpesvirales, as well as several virus families, including *Myoviridae*, *Podoviridae*, *Siphoviridae*, *Herpesviridae*, *Bicaudaviridae*, *Microviridae*, and *Phycodnaviridae*, according to a previous study by Hamedo et al. (2017) [29]. The environment of Ga’ar Lake was highly alkaline and hypersaline. As a result of these conditions, archaea viruses are highly represented [43]. According to Lanzen et al. [44], soda lakes are alkaline lakes that typically range in pH from 8.5 to >12, have large concentrations of carbonate ions, and have salinities that range from brackish to hypersaline. Haloalkaliphiles are the names given to groupings of bacteria that can thrive in alkaline environments when there is a lot of salt present [45]. They have specialized adaption mechanisms that allow them to flourish in environments with high salinity and alkaline pH. From fundamental research and biotechnology perspectives, these characteristics of the dual extreme of halophiles and alkaliphiles make them attractive [46]. Only viruses are known to prey on prokaryotes in these harsh settings. In contrast to [47], we described three lytic viruses isolated from Ga’ar Lake, with Halorubrum sp. as their host and one of these viruses being haloalkaliphiles [45]. Because of their unique adaption processes, they can grow and thrive in environments with high salt content and alkaline pH. They are intriguing from fundamental research and biotechnology perspectives due to the dual extremity of halophiles and alkaliphiles [46]. The only known predators of prokaryotes in these harsh conditions are viruses. In contrast to [47], we described three lytic viruses isolated from Ga’ar Lake, and their host, Halorubrum sp., one of which is a haloalkaliphiles virus. Additionally, it has previously been demonstrated that this region of the BJ1 virus is like the HF1 and HF2 haloarchaeal isometric head/contractile tail viruses, which have a wide host range. The previously discovered and characterized haloarchaeal head-and-tail viruses resemble bacterial caudovirales’ morphologicality [5,48]. Their genomes encode proteins identical to caudovirales’ virion assembly and genome packaging proteins, and the order of these genes is preserved. In stark contrast to their dominance in the bacterial domain, where they make up 96% of all known viral species and infect over 150 taxa, caudovirales are rare in the halophilic archaea [49]. The findings of this study support the theory that caudovirales have just recently entered the archaeal realm by showing that they are uncommon among archaea. In 2010, Télesphore Sime-Ngando showed that in meso- and bathypelagic waters, archaea make up to 30% of the prokaryotic community, a significant portion of the microbial community [50]. Recent research has shown that viruses primarily control Archaea in the deep seafloor, indicating that archaeal phages are crucial to the marine environment [51,52]. According to Hambly et al. [53,54], natural phage communities are a significant source of uncharacterized genetic diversity on Earth and are an essential resource for advancing contemporary biotechnology. Understanding phage biology can create a wide range of applications, such as novel nanotechnologies, bacterial detection methods, and biological control of pathogenic bacteria on an industrial scale. Despite their significance and ubiquity, there is still far too little knowledge about their diversity in natural ecosystems. However, research on viruses from these settings is currently minimal. Therefore, vast stores of immense genetic and biological diversity still need to be discovered and examined. In earlier research on the soda lakes, phages were isolated from Lake Magadi by Jamison et al. [55] and Muruga et al. [56]. In addition, Moulton et al. identified and examined a phage infecting an alkaliphilic Vibrio metschnikovii from Lake Magadi [57]. Peduzzi et al. [58] performed an electron microscopic examination of cyanophages that are uncommon near Ga’ar Lake, Egypt, but have an impact on the African flamingo population in Lake Nakuru.

## 5. Conclusions

This study describes the brine viral community structure of Wadi Al -Natrun, Egypt, based on a metagenomic analysis and its relationship with environmental factors. The dsDNA viromes, mainly from families *Myoviridae*, etc.; ssDNA viromes, mainly from the family *Microviridae*; and positive-strand RNA viromes, mainly from the family *Potyviridae*, dominated the local DNA viral community structure. Furthermore, we used the GenomeVx online server to visualize the virome-annotated protein. The results indicated that Myohalovirus chaoS9 has eight Contigs and Halorubrum phage CGphi46 has 19 proteins. The data presented here suggest that phages are abundant in viral communities in Wadi Al-Natrun, Egypt. In the future, the simultaneous study of the 16S rRNA, 18S rRNA genes, and viral profiles of the same brine samples should provide insight into the complex relationship between viruses and their possible hosts in the brine environment.

## Figures and Tables

**Table 1:** Genome and capsid described of family’s virus in brine sample.

Name of Family Virus	Genome	Not Enveloped or Enveloped
Myoviridae	Double-stranded DNA	Not enveloped
Podoviridae	Double-stranded DNA	Not enveloped
Siphoviridae	Double-stranded DNA	Not enveloped
Herpesviridae	Double-stranded DNA	Enveloped
Bicaudaviridae	Double-stranded DNA	Not enveloped
Microviridae	Single-stranded DNA	Not enveloped
Phycodnaviridae	Double-stranded DNA	Not enveloped
Potyviridae	Positive-strand RNA viruses	Not enveloped
Myoviridae	Double-stranded DNA	Not enveloped

**Table 2:** Virome species in brine sample detected by Genome Detective online server.

Virus Name	# Contigs	# Reads	Cover- Age (%)	Depth of Coverage	Identity (%)		Reference Genome # Acc	Host Name
					NT	AA		
*Myohalovirus ChaoS9*	8	8194	25.2	48.8	71.7	65.4	NC_054953.1	*Halobacterium salinarum*
*Halorubrum phage CGphi46*	19	7031	25.9	56.9	73.8	70	NC_021537.1	*Halorubrum* sp.
*Archaeal BJ1 virus*	24	5395	30	35.3	71.5	65	NC_008695.1	*Halorubrum saccharovorum*
*Betapleolipovirus HRPV3*	4	4928	45.7	98.8	66.1	65.6	NC_017088.1	*------*
*Myohalovirus PhiCh1*	10	3894	5.3	102.2	72	69.2	NC_054954.1	*------*
*Halovirus HCTV-1*	5	3568	3.7	76.5	64.8	60.4	NC_021330.1	*Haloarcula californiae*
*Halorubrum virus HRTV-29*	18	3508	17.7	44.9	67	64.6	NC_062762.1	*------*
*Halovirus HSTV-1*	10	3032	20.3	33.7	60.9	55	NC_021471.1	*Haloarcula sinaiiensis*
*Betapleolipovirus HRPV9*	7	2387	21.1	59.1	79.3	78.3	NC_048160.1	*------*
*Halovirus HGTV-1*	41	2231	9.8	13.1	68.5	67.9	NC_021328.1	*Halogranum* sp. *SS5-1*
*Haloferax tailed virus 1*	11	2212	12.5	39.6	62.9	60.7	NC_062739.1	*------*
*Betapleolipovirus HHPV4*	2	1588	13.5	72.9	64.9	62.6	NC_047943.1	*------*
*Myohalovirus phiH*	12	1580	7.6	29.4	70.9	65.6	NC_052650.1	*------*
*Haloarcula virus HCIV1*	1	817	3.1	70.9	62.9	57.5	NC_030848.1	*------*
*Halovirus HRTV-4*	6	777	8.3	21.7	73.1	72.4	NC_021329.1	*Halorubrum* sp. *s5a-3*
*Alphapleolipovirus HRPV6*	3	684	39	20.8	68.5	67.4	NC_017089.1	*------*
*Betapleolipovirus HHPV4*	3	564	6.3	49.8	61.8	57.1	NC_047943.1	*------*
*Alphapleolipovirus HRPV2*	3	133	9.3	10.9	67.7	62.9	NC_017087.1	*------*
*Halovirus VNH-1*	2	101	3.9	21.4	65.1	69.5	NC_025213.1	*Nanohaloarchaea archaeon*
*Betapleolipovirus HRPV12*	2	26	4.7	4.6	70.3	68.6	NC_048156.1	*------*

# Meaning number.

## Data Availability

Metagenome sequence data of Hagagy et al., 2021, are available on EMBL Metagenomics under accession no. PRJEEB18746 (https://www.ebi.ac.uk/ena/browser/view/ERR1770058 accessed on 1 December 2022).

## Supplementary Materials

The following supporting information can be downloaded at: https://www.mdpi.com/article/10.3390/genes14020323/s1, Table S1: Results Summary.
