# Supplementary material for: Diversity and Distribution Characteristics of Viruses from Soda Lakes
Source: Genes (Basel). 2023 Jan 26;14(2):323. doi: 10.3390/genes14020323 (PMC9957498; doi:10.3390/genes14020323)
Supplement: Supplementary file 1 [file genes-14-00323-s001.zip › genes-2106682-supplementary.pdf]

Results Summary

Discovery

Similar to	# Contigs	# Reads	Coverage (%)	Depth of Coverage	Identity (%)		Genome Coverage
					NT	AA	
Myohalovirus chaoS9	18	8194	25.2	48.8	71.7	65.4	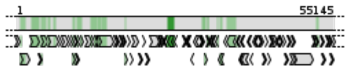
Halorubrum phage CGphi46	19	7031	25.9	56.9	73.8	70	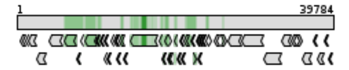
Archaeal BJ1 virus	24	5395	30	35.3	71.5	65	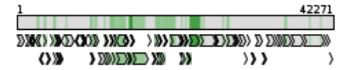
Betapleolipovirus HRPV3	4	4928	45.7	98.8	66.1	65.5	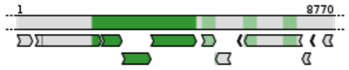
Myohalovirus PhiCh1	10	3894	5.3	102.2	72	69.2	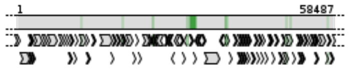
Halovirus HCTV-1	5	3568	3.7	76.5	64.8	60.4	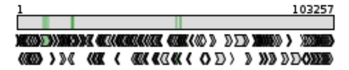
Halorubrum virus HRTV-29	18	3508	17.7	44.9	67	64.6	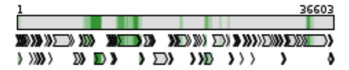
Halovirus HSTV-1	10	3032	20.3	33.7	60.9	55	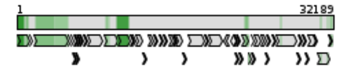
Betapleolipovirus HRPV9	7	2387	21.1	59.1	79.3	78.3	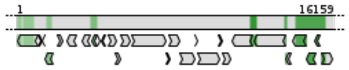
Halovirus HGTV-1	41	2231	9.8	13.1	68.5	67.9	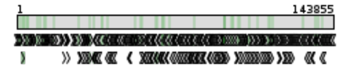
Haloferax tailed virus 1	11	2212	12.5	39.6	62.9	60.7	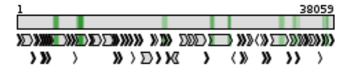
Betapleolipovirus HHPV4	2	1588	13.5	72.9	64.9	62.6	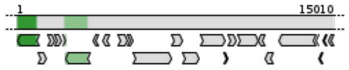
Myohalovirus phiH	12	1580	7.6	29.4	70.9	65.6	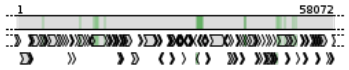
Haloarcula virus HCIV1	1	817	3.1	70.9	62.9	57.5	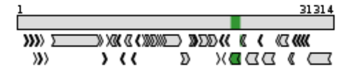
Halovirus HRTV-4	6	777	8.3	21.7	73.1	72.4	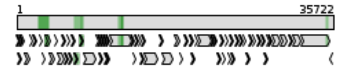

Alphapleolipovirus HRPV6	3	684	39	20.8	68.5	67.4	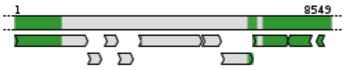
Betapleolipovirus HHPV4	3	564	6.3	49.8	61.8	57.1	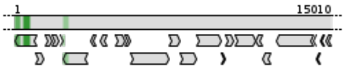
Alphapleolipovirus HRPV2	3	133	9.3	10.9	67.7	62.9	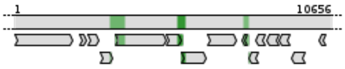
Halovirus VNH-1	2	101	3.9	21.4	65.1	69.5	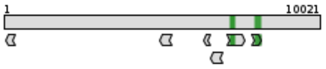
Betapleolipovirus HRPV12	2	26	4.7	4.6	70.3	68.6	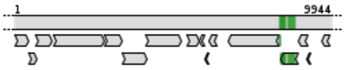

Detailed Results of Myohalovirus PhiCh1 (NC_054954.1)

NGS details

Assembly

**Coverage length**	3121 (10 contig(s))
**Est. depth of coverage**	102.2
**Est. number of reads**	3894
**Ambiguities**	0

??coverage-details?? ??variant-analysis??

Assignment

**Type**	Myohalovirus PhiCh1 (Taxonomy ID: 2845669)
**Reference Genome**	NC_054954.1 (Length: 58487bp)
**NT Identity (%)**	72.0114
**NT Quality**	0.882089

Alignment

**Alignment score**	2753 (NT) + 5297 (AA) = 8050
**Concordance (%)**	60.4445
**Alignment method**	Local, heuristic, nucleotide (BLASTN)

Genome Region

Sequence starts at position null and ends at position null relative to the null reference sequence for null.

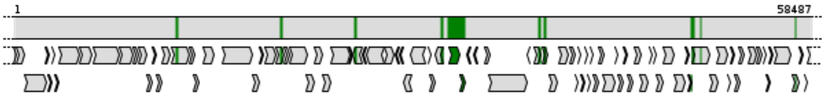

Alignment Detailed Statistics

	Begin	End	Coverage	Score	Concordance	Matches	Identities	I/D/M/F*	Stop Codons
**NT**	11822	57470	5.3%	2753.0	44.4%	3114 (98.3%)	2277 (71.9%)	48/7	
**CDS**									
cp67	100	185	20.1%	414.0	67.6%	86 (100%)	57 (66.3%)	0/0/0/0	0
KMC42_gp26	82	146	34.8%	359.0	73.1%	64 (98.5%)	43 (66.2%)	0/1/0/0	0
int1	152	215	28.4%	301.0	72.2%	64 (100%)	41 (64.1%)	0/0/0/0	0
int2	8	88	40.5%	393.0	68.5%	81 (100%)	53 (65.4%)	0/0/0/0	0
KMC42_gp45	1	257	100%	1471.0	83.3%	257 (97.7%)	218 (82.9%)	6/0/0/0	4

	Begin	End	Coverage	Score	Concordance	Matches	Identities	I/D/M/F*	Stop Codons
KMC42_gp46	1	133	100%	534.0	62.1%	133 (93.0%)	76 (53.1%)	10/0/0/0	1
KMC42_gp52	85	159	43.1%	298.0	60.3%	74 (98.7%)	43 (57.3%)	0/1/0/0	0
KMC42_gp53	19	103	57.8%	596.0	81.9%	85 (100%)	65 (76.5%)	0/0/0/0	0
KMC42_gp78	39	40	5.0%	-2.0	-33.3%	2 (100%)	1 (50.0%)	0/0/0/0	0
KMC42_gp79	1	267	29.0%	685.0	68.6%	130 (100%)	85 (65.4%)	0/0/0/0	0
KMC42_gp92	88	130	24.0%	248.0	85.8%	43 (100%)	34 (79.1%)	0/0/0/0	0
**Proteins**									
tail sheath protein (YP_010078048.1)	100	185	20.1%	414.0	67.6%	86 (100%)	57 (66.3%)	0/0/0/0	0
	N106D (11840A>G 11842C>T), E110D (11854A>C), E117A (11874A>C), D123P (11891G>C 11892A>C 11893T>G), V126H (11900G>C 11901T>A), D128E (11908C>G), F129Y (11910T>A), A131E (11916C>A 11917C>G), S137A (11933T>G 11935G>C), A138D (11937C>A 11938G>C), D141E (11947C>G), V142I (11948G>A), A143S (11951G>T), D147A (11964A>C 11965C>G), S148D (11966T>G 11967C>A 11968G>C), F154H (11984T>C 11985T>A), W158F (11997G>T 11998G>C), S159T (12000G>C 12001T>G), Q160L (12003A>T 12004A>G), S166G (12020A>G), V168A (12027T>C 12028C>G), N170R (12032A>C 12033A>G 12034C>A), V173M (12041G>A 12043C>G), F178M (12056T>A 12058C>G), D179T (12059G>A 12060A>C), L180R (12063T>G 12064G>C), K181Q (12065A>C), G182H (12068G>C 12069G>A), V183I (12071G>A)								
uncharacterized protein (YP_010078056.1)	82	146	34.8%	359.0	73.1%	64 (98.5%)	43 (66.2%)	0/1/0/0	0
	K86Q (19502A>C 19504A>G), Q87H (19507G>C), I88V (19508A>G), Q94L (19527A>T 19528G>C), E95D (19531G>C), E99S (19541G>A 19542A>G 19543A>C), R100A (19544C>G 19545G>C), V108I (19568G>A), L113I (19583C>A), L115F (19589C>T), E124D (19618G>C), D125P (19619G>C 19620A>C 19621C>T), D126G (19623A>G), F128I (19628T>A), V130L (19634G>C 19636C>G), K131E (19637A>G), S134del (19646_19648delTCG), S136T (19653G>C 19654C>T), E137V (19656A>T 19657G>C), F138V (19658T>G), L140V (19664C>G)								
site-specific integrase (YP_010078064.1)	152	215	28.4%	301.0	72.2%	64 (100%)	41 (64.1%)	0/0/0/0	0
	K157N (25126C>G), D158A (25124T>G), A159V (25120T>G 25121G>A), S163A (25108C>T 25110A>C), L165I (25102G>A 25104G>T), N166E (25099G>C 25101T>C), R167G (25096G>A 25098G>C), A173C (25078C>A 25079G>C 25080C>A), T181Y (25054C>G 25055G>T 25056T>A), E185Q (25042C>T 25044C>G), L187V (25038G>C), N188A (25033G>T 25034T>G 25035T>C), I189P (25031A>G 25032T>G), A190V (25027C>A 25028G>A), S191P (25024C>T 25026A>G), M192L (25021C>G 25023T>G), K193Q (25018C>T 25020T>G), M195L (25014T>G), A199S (25000C>G 25001G>C 25002C>T), K200D (24997C>G 24999T>C), S202A (24991G>C 24993A>C), E205Q (24982C>T 24984C>G)								
tyrosine-type recombinase/integrase (YP_010078074.1)	8	88	40.5%	393.0	68.5%	81 (100%)	53 (65.4%)	0/0/0/0	0
	A20D (31508G>T), V22A (31501G>A 31502A>G), L23H (31499A>T), E24S (31495C>A 31496T>C 31497C>T), S26R (31489A>C 31491T>G), P28G (31484G>C 31485G>C), E29L (31481T>A 31482C>G), N32E (31471G>C 31473T>C), I34L (31467T>G), L35V (31462G>T 31464G>C), L37V (31456G>C 31458G>C), S49A (31420A>T 31422A>C), G50A (31418C>G), V51L (31414G>C 31416C>G), A54D (31405C>G 31406G>T), L56V (31399G>C 31401G>C), V58L (31395C>G), D60E (31387G>T), E61G (31385T>C), L62E (31382A>T 31383G>C), S67A (31367C>G 31368T>C), R68S (31363C>T 31364C>G 31365G>A), G72D (31352C>T), D76E (31339A>C), R77N (31336C>A 31337C>T), D78T (31334T>G 31335C>T), R80S (31328C>G 31329G>A)								
ParA family protein (YP_010078075.1)	1	257	100%	1471.0	83.3%	257 (97.7%)	218 (82.9%)	6/0/0/0	4
	I3L (31933A>C), L28R (32009T>G 32010C>T), N30H (32014A>C 32016C>T), E57D (32097A>T), E67D (32127A>T), E69D (32133A>C), D72G (32141A>G), E73D (32145G>C), V78A (32159T>C 32160C>T), C86V (32182T>G 32183G>T), T92R (32200A>C 32201C>G), A99E (32222C>A 32223C>G), E108* (32248G>T), E111D (32259G>C), L114F (32266C>T), D115N (32269G>A), D115_D116insES (32271_32272insGAGAGC), G119Q (32281G>C 32282G>A 32283T>G), D122E (32292C>A), P176D (32452C>G 32453C>A 32454C>T), L180V (32464C>G 32466C>T), A181G (32468C>G), E188G (32489A>G 32490G>C), D194E (32508T>G), A198E (32519C>A 32520C>G), R201K (32528G>A), V203I (32533G>A), D206G (32543A>G), K207R (32546A>G), D209N (32551G>A), A211E (32558C>A 32559T>G), V215I (32569G>A), N227A (32605A>G 32606A>C), E237D (32637G>C), S242E (32650T>G 32651C>A), R249E (32671C>G 32672G>A 32673C>G), G253D (32684G>A 32685A>C), F254Y (32687T>A 32688C>T), F254_H255insADD* (32688_32689insGCTGATGACTGA), H255Q (32691T>G), G256* (32692G>T 32694C>A)								
uncharacterized protein (YP_010078076.1)	1	133	100%	534.0	62.1%	133 (93.0%)	76 (53.1%)	10/0/0/0	1
	M1S (32691T>G 32692G>T), A2D (32694C>A), E11D (32722A>C), D15_D16insH (32734_32735insCAC), E18D (32743G>C), E19D (32746G>T), E19_D20insHDDAVPED (32746_32747insCATGACGATGCCGTCCCCGAGGAC), A21L (32750G>C 32751C>T), E22S (32753G>T 32754A>C), N23T (32757A>C), S27E (32768T>G 32769C>A), S29T (32775G>C 32776T>A), K31N (32782A>C), E32T (32783G>A 32784A>C 32785G>A), T33A (32786A>G), K34D (32789A>G 32791G>T), D37K (32798G>A 32800T>G), V38S (32801G>A 32802T>G 32803T>C), E40K (32807G>A), A41T (32810G>A 32812C>T), S44R (32821C>A), K46E (32825A>G), S48A (32831T>G), K50M (32838A>T), T51S (32840A>T), N52K (32845T>G), T53N (32847C>A 32848A>C), E54K (32849G>A), I55M (32854C>G), T56N (32856C>A 32857G>C), S57A (32858T>G), G59S (32864G>A 32866T>C), S60K (32867T>A 32868C>A 32869A>G), E61_R62insN (32872_32873insAAC), S63E (32876T>G 32877C>A 32878C>G), G64S (32879G>A 32881G>C), K65S (32883A>G 32884A>C), P66G (32885C>G 32886C>G), D81T (32930G>A 32931A>C 32932C>G), E84D (32941G>C), E85D (32944G>C), G88L (32951G>C 32952G>T 32953C>A), F90Y (32958T>A), F91S (32961T>C 32962T>G), E94N (32969G>A 32971A>C), A95V (32973C>T 32974A>C), E96K (32975G>A), E100Q (32987G>C), L105M (33002C>A 33004C>G), K110E (33017A>G), V115A (33033T>C 33034G>A), I116V (33035A>G 33037T>C), Q117R (33039A>G 33040A>G), E121G (33051A>G), T123S (33056A>T 33058C>G), T124S (33059A>T 33061C>T), V125I (33062G>A 33064T>C), Q126E (33065C>G), Q128E (33071C>G), D130G (33078A>G)								
helix-turn-helix transcriptional regulator (YP_010078082.1)	85	159	43.1%	298.0	60.3%	74 (98.7%)	43 (57.3%)	0/1/0/0	0
	D89E (38428C>A), Q90A (38429C>G 38430A>C 38431A>C), P91R (38433C>G 38434G>C), G95A (38445G>C), I96V (38447A>G 38449A>C), R102S (38465C>T 38466G>C 38467A>C), T103Y (38468A>T 38469C>A 38470G>C), R104Y (38471C>T 38472G>A), D105E (38476C>G), V106S (38477G>T 38478T>C), V109del (38486_38488delGTC), E120D (38521G>C), I124M (38533C>G), I127V (38540A>G), E128D (38545G>C), M131E (38552A>G 38553T>A 38554G>A), V132L (38555G>C 38557G>C), R134K (38561C>A 38562G>A), E138Q (38573G>C), S140A (38579T>G 38581T>G), T141L (38582A>C 38583C>T 38584A>C), N143D (38588A>G), T144E (38591A>G 38592C>A 38593C>G), Q146Y (38597C>T 38599G>C), Q147D (38600C>G 38602G>T), L148A (38603C>G 38604T>C), V149L (38606G>C 38608C>G), D150L (38609G>C 38610A>T), Y152Q (38615T>C 38617C>G), S153F (38619C>T), S154G (38621A>G)								
HNH endonuclease (YP_010078083.1)	19	103	57.8%	596.0	81.9%	85 (100%)	65 (76.5%)	0/0/0/0	0

	Begin	End	Coverage	Score	Concordance	Matches	Identities	I/D/M/F*	Stop Codons
	G22S (38859G>A 38861G>T), S24T (38865T>A 38867A>T), E26D (38873A>C), K27E (38874A>G 38876G>A), S31T (38887G>C 38888T>C), E32D (38891G>T), D33L (38892G>C 38893A>T 38894T>C), L34G (38895C>G 38896T>G 38897C>T), I41L (38916A>C), S42P (38919T>C 38921C>G), I43F (38922A>T), S51D (38946T>G 38947C>A 38948G>T), L55V (38958C>G 38960G>C), L58V (38967T>G 38969A>G), T67K (38995C>A 38996C>G), A88H (39057G>C 39058C>A 39059T>C), H90N (39063C>A 39065T>C), Q94A (39075C>G 39076A>C 39077G>C), A97H (39084G>C 39085C>A)								
CxxC motif protein (YP_010078108.1)	39	40	5.0%	-2.0	-33.3%	2 (100%)	1 (50.0%)	0/0/0/0	0
	*40A (49709T>G 49710G>C)								
site-specific DNA-methyltransferase (YP_010078109.1)	1	267	29.0%	685.0	68.6%	130 (100%)	85 (65.4%)	0/0/0/0	0
	M1S (49709T>G 49710G>C), S3D (49714T>G 49715C>A), E11Q (49738G>C 49740G>A), E15A (49751A>C 49752G>C), V17T (49756G>A 49757T>C 49758T>G), E20K (49765G>A 49767A>G), L21M (49768C>A 49770C>G), E23A (49775A>C), S24D (49777T>G 49778C>A), C28M (49789T>A 49790G>T 49791C>G), V29C (49792G>T 49793T>G 49794C>T), V30M (49795G>A 49797C>G), F36Y (49814T>A 49815C>T), H38L (49819C>T 49820A>T 49821T>A), G42S (49831G>T 49832G>C), V43H (49834G>C 49835T>A), D44E (49839C>G), G45D (49841G>A), D51E (49860C>G), S52T (49862G>C 49863C>G), F56Y (49874T>A), E58D (49881A>C), S59R (49882T>C 49883C>G 49884A>G), D62R (49891G>C 49892A>G 49893C>T), A64G (49898C>G 49899G>T), S65N (49901G>A), E66G (49904A>G 49905G>C), I67L (49906A>C), H224D (50377C>G), N226A (50383A>G 50384A>C 50385C>T), I233V (50404A>G 50406C>G), I236V (50413A>G 50415C>G), N237T (50417A>C 50418C>G), A238T (50419G>A), A239K (50422G>A 50423C>A 50424G>A), Q240P (50426A>C), S242P (50431T>C 50433G>C), F249Y (50453T>A 50454C>T), E251P (50458G>C 50459A>C 50460G>C), K255D (50470A>G 50472G>C), D256K (50473G>A 50475C>G), S260A (50485T>G 50487A>G), E264P (50497G>C 50498A>C)								
uncharacterized protein (YP_010078122.1)	88	130	24.0%	248.0	85.8%	43 (100%)	34 (79.1%)	0/0/0/0	0
	D95E (57365C>G), E96Q (57366G>C), A99P (57375G>C 57377C>G), L116I (57426C>A), A122Q (57444G>C 57445C>A 57446C>G), V126L (57456G>C), D127E (57461C>A), V128L (57462G>C), A129S (57465G>T)								

*: Inserts / Deletes / Misaligned / Frameshifts

Detailed Results of Haloarcula virus HCIV1 (NC_030848.1)

NGS details

Assembly

**Coverage length**	962 (1 contig(s))
**Est. depth of coverage**	70.9
**Est. number of reads**	817
**Ambiguities**	0

??coverage-details?? ??variant-analysis??

Assignment

**Type**	Haloarcula virus HCIV1 (Taxonomy ID: 2049890)
**Reference Genome**	NC_030848.1 (Length: 31314bp)
**NT Identity (%)**	62.8661
**NT Quality**	0.430353

Alignment

**Alignment score**	414 (NT) + 1324 (AA) = 1738
**Concordance (%)**	43.0624
**Alignment method**	Local, heuristic, nucleotide (BLASTN)

Genome Region

Sequence starts at position null and ends at position null relative to the null reference sequence for null.

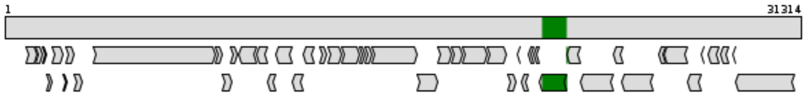

Alignment Detailed Statistics

	Begin	End	Coverage	Score	Concordance	Matches	Identities	I/D/M/F*	Stop Codons
**NT**	21153	22114	3.1%	414.0	23.0%	929 (93.9%)	601 (60.8%)	27/33	
**CDS**									
BGV91_gp35	1	313	91.0%	1300.0	59.9%	302 (94.1%)	179 (55.8%)	8/11/0/0	0
BGV91_gp36	179	187	4.8%	24.0	36.4%	9 (90.0%)	5 (50.0%)	1/0/0/0	1
**Proteins**									
hypothetical protein (YP_009272855.1)	1	313	91.0%	1300.0	59.9%	302 (94.1%)	179 (55.8%)	8/11/0/0	0

	Begin	End	Coverage	Score	Concordance	Matches	Identities	I/D/M/F*	Stop Codons
	S2T (22086G>C 22087C>G), D3G (22084T>C), G4D (22080T>G 22081C>T), A9Q (22065C>T 22066G>T 22067C>G), Q12R (22057T>C), M13R (22054A>C 22055T>G), Y14L (22051T>A 22052A>G), R16E (22045C>T 22046G>C), R17Q (22042C>T), L21I (22029G>T 22031G>T), T23D (22024G>T 22025T>C), D24E (22020G>C), D24_E25insG (22019_22020insGCC), K26E (22016T>C), A38S (21978G>C 21980C>A), F41Y (21970A>T), S43W (21964G>C), L45_K53del (21933_21959delCTTGAAGGCGTGGCCGTGCTCGTTCAA), Y56Q (21924G>C 21926A>G), K63R (21903C>G 21904T>C 21905T>G), H67S (21891G>C 21892T>G 21893G>A), I70L (21884T>G), A73G (21874G>C), L74Q (21870C>T 21871A>T), I76V (21864A>G 21866T>C), E78A (21859T>G), R81P (21850C>G), A82P (21848C>G), T84V (21841G>A 21842T>C), V86P (21835A>G 21836C>G), D87R (21831G>T 21832T>C 21833C>G), E88Q (21830C>G), H90_E91insND (21821_21822insGTCGTT), E91M (21820T>A 21821C>T), S92D (21817G>T 21818A>C), P93L (21813G>A 21814G>A), Q95E (21809G>C), F96Y (21805A>T), V97T (21802A>G 21803C>T), D98S (21798G>C 21799T>G 21800C>A), A99R (21795G>C 21796G>C 21797C>G), H100Q (21792G>T), R102D (21787C>T 21788G>C), G103A (21783G>C 21784C>G), V104I (21782C>T), W105A (21777C>G 21778C>G 21779A>C), N106D (21776T>C), A107G (21772G>C), R109F (21765C>A 21766C>A 21767G>A), N111D (21759G>A 21761T>C), F112W (21756G>C 21757A>C), N114D (21752T>C), D115E (21747G>C), T118A (21738C>A 21740T>C), Y119H (21735A>G 21737A>G), L120V (21732C>G 21734G>C), I122V (21726T>C 21728T>C), L124V (21720C>G 21722G>C), I132V (21696G>T 21698T>C), R134A (21690C>G 21691C>G 21692G>C), A137C (21681T>G 21682G>C 21683C>A), E138Q (21680C>G), E139R (21676T>C 21677C>G), F140L (21672G>C), A159T (21615C>G 21617C>T), T160A (21614T>C), Q161R (21609C>G 21610T>C), R164L (21600A>C 21601C>A 21602G>A), R164_E165insT (21599_21600insGGT), E165G (21598T>C), R166P (21594C>A 21595C>G), I167L (21591T>A 21593T>G), Q168D (21588C>G 21590G>C), A169R (21585G>T 21586G>C 21587C>G), D171Q (21579G>T 21581C>G), V174T (21571A>G 21572C>T), V175I (21567G>T 21569C>T), F181Y (21550A>T), S191G (21519C>A 21520G>C 21521A>C), E194T (21510C>G 21511T>G 21512C>T), M196L (21504C>G 21506T>G), G197Q (21502C>T 21503C>G), I201L (21489A>G 21491T>G), S204K (21481G>T 21482A>T), F205R (21478A>C 21479A>G), R206D (21475C>T 21476G>C), D207G (21471G>A 21472T>C), P208V (21469G>A 21470G>C), D209G (21466T>C), R212D (21457C>T 21458G>C), A214R (21451G>C 21452C>G), F215del (21447_21449delGAA), V219I (21435C>A 21437C>T), W220A (21432C>A 21433C>G 21434A>C), L222del (21426_21428delCAG), P225R (21417G>C 21418G>C 21419G>T), E227Q (21411C>T 21413C>G), F228L (21410A>G), G229V (21405T>G 21406C>A), D231E (21399G>C), A232D (21396G>A 21397G>T), G233_T234insEPVG (21392_21393insACCCACCGGCTC), T234S (21390G>C 21392T>A), L235I (21387C>T 21389G>T), R240V (21373C>A 21374G>C), G241A (21370C>G), I242L (21366G>C 21368T>G), L244T (21361A>G 21362G>T), E248D (21348T>G), E254T (21331T>G 21332C>T), N257S (21321G>C 21322T>G 21323T>A), T261S (21311T>A), R265K (21297G>C 21298C>T 21299G>T), Q266T (21294T>C 21295T>G 21296G>T), L267V (21291G>A 21293G>C), Q269D (21285C>G 21287G>C), R270A (21282C>G 21283C>G 21284G>C), Q272M (21277T>A 21278G>T), Q273R (21273C>T 21274T>C), S276T (21264G>C 21266A>T), D277G (21262T>C), Y291F (21220T>A), H292R (21216G>T 21217T>C), D298E (21198G>T), V300I (21192C>A 21194C>T), R301E (21189T>C 21190C>T 21191G>C), S303A (21183G>A 21184C>G 21185T>C), R305T (21178C>G 21179G>T), I308L (21170T>G), Q310D (21162C>G 21164G>C)								
hypothetical protein (YP_009272856.1)	179	187	4.8%	24.0	36.4%	9 (90.0%)	5 (50.0%)	1/0/0/0	1
	A182E (22104G>T), R183_W184insA (22099_22100insAGC), W184G (22099A>C), D185K (22094G>C 22096C>T), A186I (22092G>A 22093C>T)								

*: Inserts / Deletes / Misaligned / Frameshifts

Detailed Results of Alphapleolipovirus HRPV6 (NC_017089.1)

NGS details

Assembly

**Coverage length**	3338 (3 contig(s))
**Est. depth of coverage**	20.8
**Est. number of reads**	684
**Ambiguities**	0

??coverage-details?? ??variant-analysis??

Assignment

**Type**	Alphapleolipovirus HRPV6 (Taxonomy ID: 2747321)
**Reference Genome**	NC_017089.1 (Length: 8549bp)
**NT Identity (%)**	68.4655
**NT Quality**	0.3568

Alignment

**Alignment score**	1191 (NT) + 3652 (AA) = 4843
**Concordance (%)**	47.8227
**Alignment method**	Global, seeded, nucleotide + amino acids (AGA)

Genome Region

Sequence starts at position null and ends at position null relative to the null reference sequence for null.

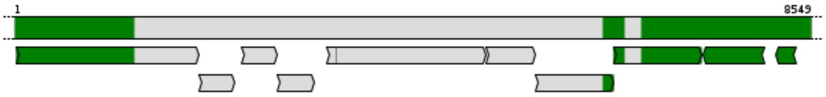

Alignment Detailed Statistics

	Begin	End	Coverage	Score	Concordance	Matches	
**NT**	2	8549	39.0%	1191.0	25.8%	2717 (81.2%)	[illegible]
**CDS**							
ORF1	1	423	64.5%	2058.0	69.9%	419 (99.1%)	[illegible]
ORF7	242	279	13.6%	221.0	79.8%	37 (92.5%)	
ORF8	1	309	81.4%	1215.0	73.1%	250 (96.9%)	[illegible]
ORF9	1	181	100%	-132.0	-76.3%	65 (29.1%)	
ORF10	1	74	100%	290.0	61.7%	74 (98.7%)	
**Proteins**							
ORF1 (YP_005454285.1)	1	423	64.5%	2058.0	69.9%	419 (99.1%)	[illegible]

	Begin	End	Coverage	Score	Concordance	Matches	
	S2T (27G>C 28T>G), S3_I5del (29_37delTCGTCGATT), S6L (38T>C 39C>T), G7A (42G>C), G8D (45G>A), S9P (47T>C 49G>C), T11R (53A>C 54C>G 55T>A), L18C (74C>T 75T>G), K20S<br>Y29H (107T>C), Q31E (113C>G), A33V (120C>T 121T>C), Y37F (132A>T 133C>T), N38T (135A>C), L49Q (168T>A 169G>A), I51V (173A>G), Q53T (179C>A 180A>C 181A>C), H56E (188<br>L73A (239C>G 240T>C 241T>C), H74K (242C>A 244C>G), D75S (245G>A 246A>G), P86S (278C>T 280G>T), E87D (283G>C), A88E (285C>A 286G>A), E90Q (290G>C), N92F (296A>T 2<br>(308A>G 309C>A 310A>T), K97T (312A>C 313A>C), P98E (314C>G 315C>A 316A>G), T99S (317A>T), H115T (365C>A 366A>C 367C>G), A116D (369C>A), K118N (376G>C), K119G (37<br>Y133T (419T>A 420A>C 421C>A), Q135E (425C>G), E136D (430A>C), L137F (431C>T), E138A (435A>C), S139D (437T>G 438C>A 439C>T), G141E (444G>A), F142L (448C>A), A143V (4<br>487T>G), N159D (497A>G), S169C (528C>G), G174A (543G>C 544C>G), S176D (548A>G 549G>A), E177S (551G>T 552A>C 553A>G), T178L (554A>C 555C>T 556G>C), H180Y (560C>T<br>594C>G), D195H (605G>C), K196D (608A>G 610A>T), K197L (611A>T 612A>T 613A>G), R198A (614C>G 615G>C 616C>T), P202S (626C>T 628A>G), L206T (638C>A 639T>C 640C>A),<br>709C>T), D230P (710G>C 711A>C 712C>G), G231A (714G>C), W232R (716T>C 718G>T), S234del (722_724delTCC), N237T (732A>C 733C>T), Q238E (734C>G), P243E (749C>G 750C><br>N257A (791A>G 792A>C 793C>G), F265L (815T>C), H267T (821C>A 822A>C 823C>G), T271D (833A>G 834C>A), D273S (839G>T 840A>C 841T>G), N274T (843A>C), C278T (854T>A 8<br>906T>C 907G>T), S296D (908A>G 909G>A 910C>T), E299D (919G>T), N300S (921A>G 922C>T), R303N (929C>A 930G>A 931G>C), M305R (935A>C 936T>G), S306N (938T>A 939C>A<br>(959A>T), D320R (980G>C 981A>G 982C>T), A321E (984C>A 985G>A), N325G (995A>G 996A>G 997T>A), M327S (1001A>T 1002T>C 1003G>T), P328E (1004C>G 1005C>A), D332S (10<br>1077C>A), A353K (1079G>A 1080C>A 1081C>G), V365P (1115G>C 1116T>C), T367R (1121A>C 1122C>G), E369D (1129G>C), T370K (1131C>A), M373C (1139A>T 1140T>G 1141G>C),<br>(1202C>G 1203T>C 1204C>A), H399Y (1217C>T 1219C>T), T411S (1253A>T 1255C>G), Q414V (1262C>G 1263A>T 1264A>C), R415H (1266G>A 1267G>C), N418L (1274A>C 1275A>T)						
ORF7 (YP_005454291.1)	242	279	13.6%	221.0	79.8%	37 (92.5%)	
	A248L (6342G>C 6343C>T 6344T>C), E249D (6347A>C), R257S (6369C>T 6370G>C 6371C>G), D258E (6374C>G), K260E (6378A>G), D261del (6381_6383delGAC), P263E (6387C>G 63						
ORF8 (YP_005454292.1)	1	309	81.4%	1215.0	73.1%	250 (96.9%)	1
	G7S (6448G>T 6449G>C 6450C>G), L18R (6482T>G 6483T>C), A19E (6485C>A 6486C>G), I22L (6493A>C), V29A (6515T>C 6516G>T), H30P (6518A>C), T34V (6529A>G 6530C>T), I35/<br>6738G>T), T104H (6739A>C 6740C>A 6741G>C), L106V (6745C>G), E107D (6750A>C), E108R (6751G>C 6752A>G 6753A>T), G111S (6760G>T 6761G>C), R113A (6766C>G 6767G>C 6<br>Q136E (6835C>G 6837A>G), L137I (6838C>A), E140R (6847G>C 6848A>G 6849G>C), V141S (6850G>T 6851T>C), H142Y (6853C>T), D144R (6859G>C 6860A>G), L145P (6863T>C 686<br>A161G (6911C>G 6912G>C), T162N (6914C>A 6915A>C), L167M (6928C>A 6930C>G), A168T (6931G>A), K169T (6935A>C), Y173H (6946T>C 6948C>T), K174Q (6949A>C), E175D (695<br>7007A>C 7008C>T), R196Y (7015C>T 7016G>A), A200S (7027G>T 7029G>C), I205L (7042A>C 7044T>C), A208S (7051G>T), M209L (7054A>C 7056G>C), K215V (7072A>G 7073A>T 707<br>(7081A>T 7082T>G 7083C>T), S236N (7135T>A 7136C>A 7137G>T), G239A (7145G>C 7146C>G), E243A (7157A>C 7158G>T), V246R (7165G>C 7166T>G), E251N (7180G>A 7182G>C)<br>S262E (7213A>G 7214G>A 7215C>G), L264V (7219C>G), A266D (7226C>A), E268T (7231G>A 7232A>C 7233G>T), V269I (7234G>A 7236A>T), T270D (7237A>G 7238C>A 7239G>C), S2<br>A281P (7270G>C), P286S (7285C>T), G298L (7321G>C 7322G>T), Y300A (7327T>G 7328A>C), S301D (7330A>G 7331G>A 7332T>C), A303Q (7336G>C 7337C>A 7338C>A), E304G (734						
ORF9 (YP_005454293.1)	1	181	100%	-132.0	-76.3%	65 (29.1%)	
	V1A (8061C>T 8062A>G), R2L (8059C>A), Y6S (8047T>G), R7T (8044C>G 8045G>T), D8A (8040G>T 8041T>G), R9del (8037_8039delGCG), T10P (8034G>C 8036T>G), A11Q (8031T>C 8<br>(8013_8015delAAG), R18G (8010C>T 8012G>C), S19G (8008G>C 8009A>C), D20E (8004G>C), T22R (7999G>C), L23G (7995C>T 7996A>C 7997G>C), V24W (7992G>C 7993A>C 7994C><br>7984A>T), D28P (7981T>G 7982C>G), A30Q (7974T>C 7975G>T 7976C>G), P33H (7966G>T), S35del (7959_7961delGGA), T37P (7953G>T 7955T>G), S38_A40del (7944_7952delGGCAT<br>7937A>C), D44C (7933T>C 7934C>A), G45Q (7929G>T 7930C>T 7931C>G), N47K (7923A>T), R48H (7920A>G 7921C>T), Y49L (7917G>T 7918T>A 7919A>G), E50T (7914C>G 7915T>G<br>P55_D58del (7890_7901delATCTGGAGAGGG), S60_G62del (7878_7886delTCCTCCTGA), G64R (7874C>T), D71_Q87del (7803_7853delCTGCGAGCGCCTGAAAACACACCTTCTGACAT<br>(7794_7796delGTG), E92_G145del<br>(7629_7790delGCCCGGAACGGCAAGCTGGCCGCGCCCGGAATCGCTCGATCTCCGAGCAGCGGAATGTTCGAGCTGTAAGACCAAGCGCTCGGACACCAGGGCAAGACTCGAAGCTGGC<br>L147G (7623C>A 7624A>C 7625G>C), R149_S179del (7527_7619delGGACACAGTACGAAAGCCCGGCAGCCCGCGCACGAGGTCAACCCGGAGCGACGAGCGAAGCGAGCCGAAGGGAA						
ORF10 (YP_005454294.1)	1	74	100%	290.0	61.7%	74 (98.7%)	
	A2E (8389G>T), S3A (8385G>T 8386C>G 8387T>C), K4D (8382T>G 8384T>C), E5R (8379C>T 8380T>C 8381C>T), R7K (8374C>T 8375G>T), A8T (8370T>C 8372C>T), W9I (8367C>G 83<br>(8337C>T 8339G>C), E22N (8328C>G 8330C>T), N26D (8318T>C), Q29N (8307C>A 8309G>T), A35T (8289G>C 8291C>T), T38E (8281G>T 8282T>C), S42T (8268G>C 8270A>T), E45D (8<br>8237T>C), N54E (8232G>C 8234T>C), E55Q (8231C>G), Q58A (8221T>G 8222G>C), Q62E (8208C>T 8210G>C), L69D (8187A>G 8188A>T 8189G>C), L70E (8184C>T 8185A>T 8186G>C<br>8177G>A)						

*: Inserts / Deletes / Misaligned / Frameshifts

Detailed Results of Betapleolipovirus HRPV12 (NC_048156.1)

NGS details

Assembly

**Coverage length**	472 (2 contig(s))
**Est. depth of coverage**	4.6
**Est. number of reads**	26
**Ambiguities**	0

??coverage-details?? ??variant-analysis??

Assignment

**Type**	Betapleolipovirus HRPV12 (Taxonomy ID: 2734336)
**Reference Genome**	NC_048156.1 (Length: 9944bp)
**NT Identity (%)**	70.339
**NT Quality**	0.813559

Alignment

**Alignment score**	384 (NT) + 830 (AA) = 1214
**Concordance (%)**	58.1975
**Alignment method**	Global, seeded, nucleotide + amino acids (AGA)

Genome Region

Sequence starts at position null and ends at position null relative to the null reference sequence for null.

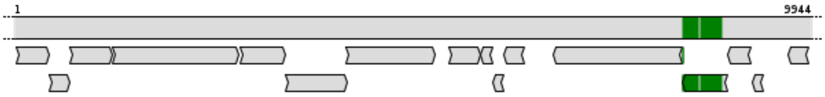

Alignment Detailed Statistics

	Begin	End	Coverage	Score	Concordance	Matches	Identities	I/D/M/F*	Stop Codons
**NT**	8343	8833	4.7%	384.0	40.7%	472 (100%)	332 (70.3%)	0/0	
**CDS**									
HOV13_gp12	1	3	0.6%	0.0	0%	3 (100%)	0 (0%)	0/0/0/0	0
HOV13_gp13	29	190	82.1%	830.0	72.4%	156 (100%)	109 (69.9%)	0/0/0/0	1
**Proteins**									
ORF9 (YP_009819946.1)	1	3	0.6%	0.0	0%	3 (100%)	0 (0%)	0/0/0/0	0
	M1V (8349T>C), I2T (8345A>G)								
AdoMet-MTase (YP_009819947.1)	29	190	82.1%	830.0	72.4%	156 (100%)	109 (69.9%)	0/0/0/0	1

	Begin	End	Coverage	Score	Concordance	Matches	Identities	I/D/M/F*	Stop Codons
	Q33R (8818T>C), T35D (8811C>G 8812G>T 8813T>C), K39E (8799T>C 8801T>C), A43K (8788G>T 8789C>T), S46T (8778G>A 8780A>T), V52A (8760T>G 8761A>G), K56R (8748T>C 8749T>C 8750T>G), D60V (8736A>G 8737T>A), R63D (8728C>T 8729G>C), N71D (8705T>C), K73D (8697C>G 8699T>C), T78L (8683G>A 8684T>G), H80V (8677T>A 8678G>C), I85V (8663T>C), S86A (8658T>C 8660A>C), E87D (8655T>G), Y88H (8652A>G 8654A>G), Y89F (8650T>A), A91P (8643A>G 8645C>G), G92E (8640A>C 8641C>T), I97V (8625T>C 8627T>C), I98V (8622T>G 8624T>C), D105Q (8601G>C 8603C>G), D109N (8591C>T), R111S (8583T>G), K125R (8541C>G 8542T>C 8543T>G), E126Q (8538T>C 8540C>G), L127F (8535C>G), N128D (8534T>C), T129E (8529G>C 8530G>T 8531T>C), I131L (8523T>G 8525T>G), T136R (8509G>C 8510T>G), I137F (8505T>G 8507T>A), Y142F (8490A>G 8491T>A), C146G (8478A>C 8480A>C), P152G (8461G>C 8462G>C), W161F (8433C>G 8434C>A), S167M (8416G>A 8417A>T), S172A (8402A>C), I174V (8394T>G 8396T>C), Q176E (8388C>T 8390G>C), E178P (8382C>G 8383T>G 8384C>G), N181L (8374T>A 8375T>G), D182E (8370A>C), S183A (8367T>G 8369A>C), E184G (8364C>A 8365T>C)								

*: Inserts / Deletes / Misaligned / Frameshifts

Detailed Results of Myohalovirus chaoS9 (NC_054953.1)

NGS details

Assembly

**Coverage length**	13916 (18 contig(s))
**Est. depth of coverage**	48.8
**Est. number of reads**	8194
**Ambiguities**	0

??coverage-details?? ??variant-analysis??

Assignment

**Type**	Myohalovirus chaoS9 (Taxonomy ID: 2496992)
**Reference Genome**	NC_054953.1 (Length: 55145bp)
**Host(s)**	Halobacterium salinarum
**NT Identity (%)**	71.65
**NT Quality**	0.845645

Alignment

**Alignment score**	11768 (NT) + 20924 (AA) = 32692
**Concordance (%)**	55.92
**Alignment method**	Local, heuristic, nucleotide (BLASTN)

Genome Region

Sequence starts at position null and ends at position null relative to the null reference sequence for null.

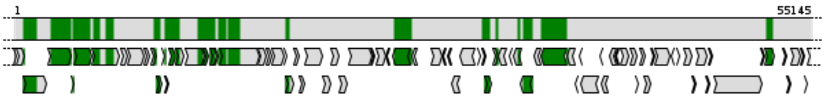

Alignment Detailed Statistics

	Begin	End	Coverage	Score	Concordance	Matches	Identities	I/D/M/F*	Stop Codons
**NT**	605	52560	25.2%	11768.0	42.8%	13845 (98.9%)	9983 (71.3%)	88/71	
**CDS**									
KMC41_gp02	170	190	11.1%	9.0	5.9%	21 (95.5%)	12 (54.5%)	1/0/1/1	0
terL	1	317	57.7%	1711.0	77.5%	314 (98.7%)	233 (73.3%)	1/3/0/0	0
por	81	536	84.0%	2004.0	62.0%	456 (98.5%)	278 (60.0%)	7/0/0/0	0
KMC41_gp05	43	49	14.3%	18.0	54.5%	7 (100%)	5 (71.4%)	0/0/0/0	1

	Begin	End	Coverage	Score	Concordance	Matches	Identities	I/D/M/F*	Stop Codons
KMC41_gp06	1	374	94.4%	1906.0	73.2%	366 (96.3%)	254 (66.8%)	6/8/0/0	0
KMC41_gp07	22	492	53.7%	1495.0	63.8%	341 (99.1%)	216 (62.8%)	1/2/0/0	0
KMC41_gp11	66	129	49.6%	255.0	57.2%	64 (100%)	38 (59.4%)	0/0/0/0	1
hco	1	117	85.4%	560.0	69.3%	117 (99.2%)	78 (66.1%)	1/0/0/0	0
KMC41_gp13	74	78	6.4%	18.0	58.1%	5 (100%)	4 (80.0%)	0/0/0/0	0
KMC41_gp14	1	96	100%	339.0	53.1%	94 (97.9%)	47 (49.0%)	0/2/0/0	1
nep	1	149	100%	593.0	60.6%	149 (100%)	86 (57.7%)	0/0/0/0	1
tco	1	77	39.1%	340.0	60.3%	77 (100%)	43 (55.8%)	0/0/0/0	0
KMC41_gp17	312	435	28.5%	449.0	53.2%	124 (100%)	65 (52.4%)	0/0/0/0	0
KMC41_gp18	1	133	100%	626.0	67.0%	133 (100%)	83 (62.4%)	0/0/0/0	0
KMC41_gp19	1	141	95.9%	427.0	45.0%	141 (100%)	60 (42.6%)	0/0/0/0	1
KMC41_gp20	54	70	24.3%	59.0	57.3%	16 (94.1%)	12 (70.6%)	0/1/0/0	1
tpm	1	476	48.5%	1743.0	63.6%	444 (100%)	276 (62.2%)	0/0/0/0	0
KMC41_gp26	3	78	41.3%	320.0	65.6%	76 (100%)	46 (60.5%)	0/0/0/0	0
tnpB	4	381	90.2%	1612.0	56.7%	376 (97.2%)	212 (54.8%)	9/2/0/0	0
KMC41_gp47	1	82	100%	237.0	47.8%	79 (95.2%)	48 (57.8%)	1/3/1/1	1
KMC41_gp48	1	104	72.7%	370.0	57.2%	104 (100%)	54 (51.9%)	0/0/0/0	0
KMC41_gp49	67	116	42.7%	317.0	88.8%	50 (100%)	40 (80.0%)	0/0/0/0	0
KMC41_gp52	6	75	38.3%	264.0	66.0%	70 (97.2%)	41 (56.9%)	2/0/0/0	0
parA	9	222	75.1%	796.0	56.3%	214 (99.5%)	143 (66.5%)	1/0/2/2	0
KMC41_gp55	1	594	100%	3577.0	93.1%	593 (99.8%)	540 (90.9%)	0/1/0/0	1
KMC41_gp56	205	205	0.5%	1.0	14.3%	1 (100%)	1 (100%)	0/0/0/0	1
KMC41_gp80	36	210	79.5%	878.0	66.6%	175 (99.4%)	120 (68.2%)	1/0/1/1	0
**Proteins**									
HTH domain protein (YP_010077949.1)	170	190	11.1%	9.0	5.9%	21 (95.5%)	12 (54.5%)	1/0/1/1	0
	M174L (619A>C), S175D (622T>G 623C>A 624G>C), K178R (631A>C 632A>G 633A>G), D179A (635A>C), G182E (644G>A 645G>A), G183E (647G>A 648T>G), D184G (650A>G 651C>T), D185_D186insX (654_655insT), E189C (664G>T 665A>G 666G>C), *190Q (667T>C 669A>G)								
terminase large subunit (YP_010077950.1)	1	317	57.7%	1711.0	77.5%	314 (98.7%)	233 (73.3%)	1/3/0/0	0
	K3S (663A>G 664G>T), S4A (665A>G 666G>C 667T>C), N5S (669A>G 670T>C), A7N (674G>A 675C>A), T8L (677A>C 678C>T 679C>A), G9S (680G>T 681G>C), A10E (684C>A 685C>G), G11D (687G>A), G12_T13del (689_694delGGCACC), A16T (701G>A 703G>A), K25G (728A>G 729A>G 730G>C), I35L (758A>C), N46L (791A>C 792A>T 793C>G), L47V (794C>G), G48T (797G>A 798G>C 799C>G), Q49E (800C>G 802G>A), R52A (809C>G 810G>C 811C>G), Q53E (812C>G), R56Q (821A>C 822G>A 823G>A), V58L (827G>C), T60Q (833A>C 834C>A 835C>A), S68T (857T>A 859C>G), I83L (902A>C), F87L (914T>C), V93T (932G>A 933T>C 934G>C), S94A (935T>G 937G>C), F95L (938T>C 940C>T), K105R (968A>C 969A>G), A114S (995G>T), S117K (1004T>A 1005C>A), D118N (1007G>A), A119G (1011C>G), I121V (1016A>G 1018C>A), P122S (1019C>T 1021G>T), E126K (1031G>A), H129S (1040C>A 1041A>G 1042C>T), P132E (1049C>G 1050C>A), I136F (1061A>T), E137D (1066G>C), G138N (1067G>A 1068G>A), E139D (1072G>T), F144W (1086T>G 1087C>G), S147T (1094T>A), R150K (1103C>A 1104G>A 1105C>G), V158A (1128T>C 1129C>T), S160E (1133T>G 1134C>A 1135C>G), A173P (1172G>C), A174E (1176C>A 1177C>A), K177E (1184A>G), R191K (1226C>A 1227G>A 1228C>G), D214del (1295_1297delGAC), N217K (1306C>G), A228S (1337G>T 1339C>G), S234D (1355T>G 1356C>A 1357G>C), M235I (1360G>C), N237H (1364A>C), I238V (1367A>G), D239E (1372C>G), R242K (1379C>A 1380G>A), I249V (1400A>G), E253R (1412G>C 1413A>G 1414G>C), V257E (1425T>A 1426C>G), N260H (1433A>C), N260_A261insP (1435_1436insCCG), A261S (1436G>T 1438C>G), S262I (1440G>T), E263P (1442G>C 1443A>C 1444G>C), L267V (1454C>G 1456C>T), T269K (1461C>A 1462A>G), L271R (1467T>G 1468G>A), V273I (1472G>A 1474C>A), N279D (1490A>G), E280A (1494A>C 1495G>C), D281S (1496G>A 1497A>G), L284P (1506T>C 1507C>G), E287T (1514G>A 1515A>C), R288T (1517C>A 1518G>C 1519C>A), D289A (1521A>C 1522C>G), A291H (1526G>C 1527C>A 1528C>T), R299E (1550C>G 1551G>A 1552C>G), P300D (1553C>G 1554C>A), H307Y (1574C>T), M314I (1597G>A)								

	Begin	End	Coverage	Score	Concordance	Matches	Identities	I/D/M/F*	Stop Codons
portal protein (YP_010077951.1)	81	536	84.0%	2004.0	62.0%	456 (98.5%)	278 (60.0%)	7/0/0/0	0
	E85A (2569A>C), N86T (2572A>C 2573C>G), R88A (2577C>G 2578G>C 2579C>T), Y90N (2583T>A), R92Q (2590G>A), Q93N (2592C>A 2594G>C), K96N (2603G>C), E97D (2606G>C), K106H (2631A>C 2633G>C), A113I (2652G>A 2653C>T), C121V (2676T>G 2677G>T), D123N (2682G>A), N125S (2689A>G), D130R (2703G>C 2704A>G), E131D (2708G>C), T134D (2715A>G 2716C>A 2717G>C), K137D (2724A>G 2726G>C), A143C (2742G>T 2743C>G 2744C>T), T150L (2763A>C 2764C>T), N151G (2766A>G 2767A>G), Q152H (2771G>C), S155R (2778T>C 2779C>G 2780C>G), I156K (2782T>A 2783C>G), I158L (2787A>C 2789C>G), E159G (2791A>G 2792G>C), R160S (2793C>T 2794G>C), I161L (2796A>C 2798C>G), Q164K (2805C>A), F172L (2829T>C 2831C>G), R179E (2850C>G 2851G>A), S180Q (2853T>C 2854C>A 2855G>A), R182P (2860G>C 2861A>G), N183D (2862A>G), T184A (2865A>G), I185T (2869T>C 2870C>G), A186V (2872C>T 2873G>C), M190S (2883A>T 2884T>C), D192E (2891T>G), M196F (2901A>T 2903G>C), D197K (2904G>A 2906C>G), K200T (2914A>C 2915G>C), N203R (2922A>C 2923A>G), T205P (2928A>C), H209Q (2942C>G), E211G (2947A>G 2948G>C), P216S (2961C>T 2963G>C), E217D (2966A>C), T221S (2976A>T 2978T>G), D223T (2982G>A 2983A>C), R224G (2985C>G 2987C>G), E225K (2988G>A), E230T (3003G>A 3004A>C 3005A>G), A233H (3012G>C 3013C>A), D234E (3017C>G), S235D (3018T>G 3019C>A 3020G>C), V236_S237insPG (3023_3024insCCTGGG), S237H (3024A>C 3025G>A), E238D (3029G>C), E240K (3033G>A), E241D (3038G>C), A244H (3045G>C 3046C>A 3047G>C), S246A (3051T>G), K247T (3055A>C 3056G>C), F253L (3072T>C 3074C>G), V254T (3075G>A 3076T>C 3077G>C), P257A (3084C>G 3086G>C), T259D (3090A>G 3091C>A 3092G>C), A262V (3100C>T), I267L (3114A>C 3116C>A), E269D (3122G>C), D272S (3129G>T 3130A>C), S277A (3144T>G), L279K (3150C>A 3151T>A 3152T>A), K280Q (3153A>C), G283E (3163G>A 3164T>G), I287F (3174A>T), W294Y (3196G>A 3197G>C), Q295P (3199A>C 3200G>C), H300S (3213C>T 3214A>C), C301S (3217G>C 3218T>G), K302D (3219A>G 3221G>C), N303S (3223A>G 3224C>T), K306Q (3231A>C), E307Q (3234G>C), Q308E (3237C>G), T310E (3243A>G 3244C>A 3245A>G), D311A (3247A>C), F312Y (3250T>A), K314E (3255A>G), S315A (3258A>G 3259G>C), Q317K (3264C>A), N318G (3267A>G 3268A>G 3269C>G), A323S (3282G>A 3283C>G 3284C>T), K324E (3285A>G), D328E (3299C>G), E330D (3305G>C), D332_N333insRGGEQ (3311_3312insCGCGGCGGCGAGCAG), P334S (3315C>T 3317C>G), G344N (3345G>A 3346G>A 3347G>C), E345T (3348G>A 3349A>C), G347E (3355G>A 3356T>G), I348V (3357A>G), E349Q (3360G>C), T350V (3363A>G 3364C>T 3365T>C), V351E (3367T>A 3368C>G), N354S (3375A>T 3376A>C 3377C>G), E357D (3386G>T), I358L (3387A>C), D359E (3392C>G), M363R (3402A>C 3403T>G), Q367E (3414C>G), S376G (3441T>G 3442C>G 3443C>G), R377K (3444C>A 3445G>A), F378Y (3448T>A), K379Q (3450A>C), E383A (3463A>C 3464G>C), E384D (3467G>C), G385D (3469G>A), I386L (3471A>C 3473C>G), E392D (3491G>C), A395R (3498G>C 3499C>G), K397R (3504A>C 3505A>G 3506G>C), D399Q (3510G>C 3512C>G), R400E (3513C>G 3514G>A 3515A>G), H403D (3522C>G), K404S (3525A>T 3526A>C 3527G>C), L413F (3552C>T), V416I (3561G>A), E418V (3568A>T), A426S (3591G>A 3592C>G), E431D (3608G>C), H435S (3618C>T 3619A>C), D450E (3665C>G), A454D (3676C>A 3677G>C), E458Q (3687G>C 3689A>G), T459A (3690A>G), L460F (3693C>T 3695G>C), T461A (3696A>G 3698A>G), T462S (3700C>G 3701T>C), L464W (3705C>T 3706T>G 3707T>G), Q465K (3708C>A 3710G>A), E466A (3712A>C 3713G>C), F467Y (3715T>A), Q469E (3720C>G), M474Q (3735A>C 3736T>A), V478A (3748T>C), L481F (3756C>T 3758G>C), F482A (3759T>G 3760T>C), I487F (3774A>T), R491E (3786C>G 3787G>A 3788G>A), Y492L (3789T>C 3790A>T), Q493R (3793A>G 3794G>C), Q494D (3795C>G 3797G>C), K495R (3798A>C 3799A>G 3800G>T), Y496H (3801T>C), D497E (3806C>A), D499E (3812C>G), P500V (3813C>G 3814C>T 3815G>C), I505L (3828A>C), E508D (3839G>C), D509E (3842C>G), T512E (3849A>G 3850C>A), D513E (3854C>G), S514R (3855T>C 3856C>G 3857G>C), M515L (3858A>C 3860G>C), P516D (3861C>G 3862C>A), D517E (3866C>A), V518L (3867G>C 3869C>G), G519E (3871G>A 3872C>G), A520S (3873G>T), E521M (3876G>A 3877A>T), N522I (3880A>T), D523Q (3882G>C 3884T>A), G525Q (3888G>C 3889G>A 3890C>G), T526Q (3891A>C 3892C>A 3893T>G), E529D (3902A>C), G530N (3903G>A 3904G>A), V533D (3913T>A 3914C>T), V534D (3916T>A)								
CxxC motif protein (YP_010077952.1)	43	49	14.3%	18.0	54.5%	7 (100%)	5 (71.4%)	0/0/0/0	1
	L45H (4070T>A 4071C>T), N46L (4072A>C 4073A>T)								
putative head assembly protein (YP_010077953.1)	1	374	94.4%	1906.0	73.2%	366 (96.3%)	254 (66.8%)	6/8/0/0	0
	P5S (4098C>T 4100G>C), N18S (4138A>G 4139C>T), A21G (4147C>G 4148G>C), H24N (4155C>A), A29S (4170G>T), S31T (4176T>A 4178T>G), A35E (4189C>A 4190T>G), V41H (4206G>C 4207T>A 4208C>T), D43_R44insE (4214_4215insGAG), R44H (4216G>A), V45I (4218G>A), D60Q (4263G>C 4265T>G), G63P (4272G>C 4273G>C 4274C>T), D67N (4284G>A), K77E (4314A>G), Q80R (4324A>G 4325G>A), E87D (4346G>C), S91A (4356A>G 4357G>C 4358C>G), Q95R (4369A>G), E99D (4382A>T), F103L (4392T>C), D104G (4396A>G), E106D (4403G>C), A107_G109del (4404_4412delGCCCGGGGC), T113G (4422A>G 4423C>G), D114E (4427T>G), D115G (4429A>G 4430C>G), V116L (4431G>C), G117del (4434_4436delGGC), A118S (4437G>T 4439C>G), G119L (4440G>C 4441G>T), V120S (4443G>T 4444T>C 4445C>T), Q122I (4449C>A 4450A>T 4451G>C), A125S (4458G>T 4460C>G), A125_D126insSEVPD (4460_4461insTCCGAAGTGCCGGAT), D126E (4463T>G), R127V (4464C>G 4465G>T 4466G>C), R129V (4470C>G 4471G>T 4472C>G), F131Y (4477T>A), D133E (4484C>G), E142D (4511G>T), R144_S147del (4515_4526delCGCGAACTGTCG), D149N (4530G>A), V151I (4536G>A), S160D (4563T>G 4564C>A 4565G>C), D161E (4568C>G), Q163V (4572C>G 4573A>T 4574G>T), T164G (4575A>G 4576C>G 4577C>G), Y167F (4585A>T), I168V (4587A>G), Q170D (4593C>G 4595G>T), D174R (4605G>C 4606A>G), Q178T (4617C>A 4618A>C), E185D (4640G>C), A186Q (4641G>C 4642C>A 4643C>G), Q187E (4644C>G), D189Q (4650G>C 4652C>G), D196E (4673C>G), E199D (4682G>C), I203V (4692A>G 4694C>T), Q204R (4696A>G 4697G>A), S205G (4698A>G 4700C>G), S206A (4701T>G 4703C>G), L211V (4716C>G), D215N (4728G>A), S216A (4731T>G), V220A (4744T>C), H222L (4750A>T), I224A (4755A>G 4756T>C 4757C>A), V226L (4761G>C), S229A (4770T>G), V230L (4773G>C), R231Q (4777G>A), M232R (4779A>C 4780T>G 4781G>C), I234V (4785A>G 4787C>T), E236Q (4791G>C), R238K (4797C>A 4798G>A 4799C>G), Q240S (4803C>T 4804A>C), F243Y (4813T>A), Y247F (4825A>T), Q251R (4837A>G 4838G>A), D255Q (4848G>C 4850T>G), D256E (4853C>G), A258D (4858C>A 4859G>C), D267Q (4884G>C 4886C>G), V273N (4902G>A 4903T>A), M278I (4919G>T), M282L (4929A>C), A283T (4932G>A), I286V (4941A>G), S288K (4947T>A 4948C>A 4949C>G), H304Y (4995C>T), G305A (4999G>C 5000C>G), A310T (5013G>A 5015C>G), R313Q (5023G>A 5024C>G), E316R (5031G>C 5032A>G), L318H (5038T>A), D322E (5051C>G), E324Q (5055G>C), I325V (5058A>G 5060C>A), E326Q (5061G>C), A327T (5064G>A), S335Q (5088T>C 5089C>A 5090T>G), D339E (5102C>G), D345E (5120T>G), E347R (5124G>C 5125A>G 5126G>C), M349L (5130A>T), S350T (5133T>A 5135C>A), I351L (5136A>C), Q352K (5139C>A), E353D (5144G>C), S356R (5151A>C), N357D (5154A>G 5156C>T), A369V (5191C>T), R370I (5193C>A 5194G>T 5195G>A), A372S (5199G>T)								
putative prohead protease protein (YP_010077954.1)	22	492	53.7%	1495.0	63.8%	341 (99.1%)	216 (62.8%)	1/2/0/0	0
	E26K (5459G>A), D27N (5462G>A), D29E (5470T>G), V30E (5472T>A 5473C>G), Y31Q (5474T>C 5476C>G), E32Q (5477G>C), I38L (5495A>C), V43I (5510G>A 5512A>C), T45R (5516A>C 5517C>G), Q47G (5522C>G 5523A>G 5524G>C), E50K (5531G>A), P51E (5534C>G 5535C>A 5536C>G), H53Y (5540C>T), T58V (5555A>G 5556C>T), L60K (5561C>A 5562T>A 5563C>A), Q61E (5564C>G 5566G>A), E64D (5575G>C), Q65S (5576C>A 5577A>G 5578G>C), G70K (5591G>A 5592G>A 5593A>G), E84D (5635G>T), D87E (5644C>G), I95V (5666A>G), G97S (5672G>T 5673G>C), V98A (5676T>C), D99G (5679A>G), E100T (5681G>A 5682A>C), W101F (5685G>T 5686G>C), T102E (5687A>G 5688C>A 5689C>G), E105D (5698G>C), G111S (5714G>T 5715G>C), H112E (5717C>G 5719C>G), H112_G113insN (5719_5720insAAT), G113A (5721G>C), K114E (5723A>G), E118D (5737G>C), R119H (5739G>A 5740G>T), M120L (5741A>C), K122E (5747A>G), I124V (5753A>G), H126A (5759C>G 5760A>C), D127G (5763A>G 5764T>G), D130E (5773C>G), F136W (5790T>G 5791T>G), L138V (5795C>G 5797G>C), D140G (5802A>G), G141E (5805G>A), N149Y (5828A>T), T150R (5831A>C 5832C>G), L155I (5846C>A 5848G>C), V162L (5867G>C 5869C>G), I298E (6275A>G 6276T>A 6277A>G), M299I (6280G>C), E300D (6283A>C), D301A (6285A>C), H302S (6287C>A 6288A>G), D303E (6292C>G), V304R (6293G>C 6294T>G), E305G (6297A>G 6298G>C), I306V (6299A>G), F308V (6305T>G 6307C>G), I309T (6309T>C), D310V (6312A>T), E312del (6317_6319delGAA), G316R (6329G>C), T324A (6353A>G 6355C>G), F325Y (6357T>A), I329F (6368A>T), I334L (6383A>C), E339A (6399A>C), S340N (6401T>A 6402C>A), S343A (6410T>G), L344I (6413C>A), D345E (6418C>G), T348S (6425A>T 6427C>G), A350T (6431G>A 6433T>C), S353E (6440T>G 6441C>A), I361V (6464A>G), V363I (6470G>A 6472G>C), I364V (6473A>G), D370A (6492A>C), D374E (6505C>G), N378Q (6515A>C 6517C>G), E384Q (6533G>C), G385D (6537G>A 6538G>C), L390V (6551C>G), V397I (6572G>A 6574T>C), E399D (6580G>C), A407G (6603C>G), A409P (6608G>C 6610G>C), R410G (6611C>G 6613T>G), D411P (6614G>C 6615A>C), P413A (6620C>G 6622T>A), G414Q (6623G>C 6624G>A 6625C>G), G415N (6626G>A 6627G>A 6628A>C), G418E (6636G>A 6637A>G), Q419R (6639A>G), S421del (6644_6646delTCT), D422E (6649C>A), E423P (6650G>C 6651A>C 6652G>C), E426G (6660A>G), P427T (6662C>A), R428G (6665C>G), V429T (6668G>A 6669T>C 6670G>T), P431D (6674C>G 6675C>A), G432S (6677G>T 6678G>C 6679T>C), S433G (6680T>G 6681C>G 6682C>T), T435S (6687C>G 6688A>C), D436G (6690A>G), P437T (6692C>A 6694A>T), T438S (6695A>T 6697C>A), S439Q (6698A>C 6699G>A 6700T>G), T442V (6707A>G 6708C>T 6709T>A), E443D (6712G>C), E444P (6713G>C 6714A>C 6715G>C), T445E (6716A>G 6717C>A 6718C>A), K455R (6746A>C 6747A>G 6748G>A), A456T (6749G>A 6751A>C), N459D (6758A>G), Q460S (6761C>A 6762A>G 6763G>T), E462A (6768A>C 6769A>C), E463D (6772A>C), T465K (6777C>A), D476E (6811C>G), Q478T (6815C>A 6816A>C), E481N (6824G>A 6826A>C), D484E (6835C>G), E491G (6855A>G)								
uncharacterized protein (YP_010077958.1)	66	129	49.6%	255.0	57.2%	64 (100%)	38 (59.4%)	0/0/0/0	1
	S73E (9614T>G 9615C>A 9616C>G), I76Q (9623A>C 9624T>A 9625C>G), T77V (9626A>G 9627C>T), T78E (9629A>G 9630C>A), S81D (9638T>G 9639C>A 9640G>C), T82I (9642C>T 9643A>C), I85V (9650A>G 9652C>G), D86S (9653G>T 9654A>C 9655T>G), R87F (9656C>T 9657G>T 9658G>C), F89Y (9663T>A), F93L (9674T>C), D94S (9677G>T 9678A>C), G95E (9681G>A 9682T>G), S97G (9686T>G 9687C>G), P102T (9701C>A), Y103H (9704T>C), T106Q (9713A>C 9714C>A), A107T (9716G>A), D109E (9724T>G), D110Y (9725G>T), E112T (9731G>A 9732A>C 9733G>C), T113S (9734A>T 9736C>G), G114D (9738G>A), E119G (9753A>G 9754A>C), G121S (9758G>A 9760C>T), L128A (9779C>G 9780T>C)								

	Begin	End	Coverage	Score	Concordance	Matches	Identities	I/D/M/F*	Stop Codons
head closure protein, type 1 (YP_010077959.1)	1	117	85.4%	560.0	69.3%	117 (99.2%)	78 (66.1%)	1/0/0/0	0
	S3A (9787T>G 9789C>G), Y4F (9791A>T 9792T>C), H5A (9793C>G 9794A>C 9795C>A), K7Q (9799A>C 9801A>G), Q8R (9803A>G), T9A (9805A>G), D13A (9818A>C), D16T (9826G>A 9827A>C), A18E (9833C>A 9834C>G), R21N (9841C>A 9842G>A), D23E (9849T>G), S24Q (9850A>C 9851G>A 9852C>G), G25T (9853G>A 9854G>C), Q27T (9859C>A 9860A>C), T28S (9862A>T), T28_S29insG (9864_9865insGGC), R32A (9874C>G 9875G>C 9876A>T), Q33P (9878A>C 9879G>C), T34R (9880A>C 9881C>G), V35T (9883G>A 9884T>C), M36Q (9886A>C 9887T>A), A37T (9889G>A), E38D (9894G>C), S39D (9895A>G 9896G>A), K42R (9904A>C 9905A>G), S43P (9907T>C 9909C>G), L55V (9943C>G), S56A (9946T>G), Q59T (9955C>A 9956A>C), W60Y (9959G>A 9960G>C), K63E (9967A>G), E65D (9975A>T), A72V (9995C>T), N92D (10054A>G), D94N (10060G>A), I99L (10075A>C), S103N (10087T>A 10088C>A 10089G>C), T104S (10090A>T 10092G>C), L105Q (10094T>A), M106P (10096A>C 10097T>C)								
uncharacterized protein (YP_010077960.1)	74	78	6.4%	18.0	58.1%	5 (100%)	4 (80.0%)	0/0/0/0	0
	*78R (10419T>C)								
uncharacterized protein (YP_010077961.1)	1	96	100%	339.0	53.1%	94 (97.9%)	47 (49.0%)	0/2/0/0	1
	E6P (10429G>C 10430A>C), D7E (10434C>G), L8A (10435C>G 10436T>C 10437C>G), R10V (10441C>G 10442G>T 10443A>C), E15A (10457A>C), A16K (10459G>A 10460C>A 10461C>G), V17A (10463T>C 10464C>G), D18R (10465G>C 10466A>G 10467C>T), D19R (10468G>C 10469A>G), V20L (10471G>C), G21D (10475G>A), A22D (10478C>A), R24Q (10484G>A 10485A>G), Q26A (10489C>G 10490A>C 10491G>C), A27D (10493C>A 10494G>C), I29V (10498A>G), D30E (10503C>G), F31Y (10505T>A), Q34E (10513C>G), K39T (10529A>C), K46E (10549A>G 10551G>A), I50V (10561A>G), R51E (10564C>G 10565G>A), I52V (10567A>G), R53I (10570C>A 10571G>T 10572A>C), D56_P57del (10579_10584delGATCCT), A58G (10586C>G 10587T>G), R59S (10588C>A 10590A>C), V62A (10598T>C 10599T>C), A63G (10601C>G), V65I (10606G>A), E69D (10620G>C), P70V (10621C>G 10622C>T 10623A>C), A71D (10625C>A 10626G>C), T72E (10627A>G 10628C>A 10629T>G), E73S (10630G>T 10631A>C 10632A>G), Q74K (10633C>A 10635G>A), I75T (10637T>C), H80R (10652A>G 10653C>G), I82V (10657A>G), D83E (10662C>G), R84T (10663C>A 10664G>C), E87A (10673A>C), D88A (10676A>C), D90A (10682A>C), V92E (10688T>A), E93V (10691A>T), V94I (10693G>A)								
putative neck protein, type 1 (YP_010077962.1)	1	149	100%	593.0	60.6%	149 (100%)	86 (57.7%)	0/0/0/0	1
	E5N (10715G>A 10717G>C), V11A (10734T>C 10735C>T), A12R (10736G>C 10737C>G 10738T>C), E13D (10741G>C), D16E (10750C>G), D17G (10752A>G 10753T>C), A20K (10760G>A 10761C>A 10762T>G), A21T (10763G>A), L23F (10769C>T), V24A (10773T>C 10774G>C), H27Y (10781C>T 10783C>T), S28L (10784A>C 10785G>T 10786T>C), T30V (10790A>G 10791C>T 10792G>C), G32R (10796G>C 10798G>A), L34T (10802C>A 10803T>C 10804C>G), D35L (10805G>C 10806A>T 10807C>G), Q36N (10808C>A 10810G>C), M38W (10814A>T 10815T>G), K39R (10817A>C 10818A>G 10819G>C), S40A (10820T>G 10822C>G), Q42K (10826C>A), D48S (10844G>T 10845A>C 10846C>G), E50P (10850G>C 10851A>C), P51T (10853C>A), I52V (10856A>G), K53E (10859A>G 10861A>G), Q58A (10874C>G 10875A>C), K61N (10885G>C), A68R (10904G>C 10905C>G), P69A (10907C>G), S74D (10922A>G 10923G>A), N75E (10925A>G 10927C>G), L76T (10928C>A 10929T>C), G78A (10935G>C 10936C>G), D79S (10937G>T 10938A>C 10939C>G), I80V (10940A>G 10942C>T), T83D (10949A>G 10950C>A 10951G>C), T85E (10955A>G 10956C>A 10957C>G), V86L (10958G>C), N89D (10967A>G), R90G (10970C>G 10972C>A), I92Q (10976A>C 10977T>A 10978C>G), G93A (10980G>C), I94V (10982A>G 10984C>G), T97S (10991A>T), T98S (10994A>T), L99K (10997C>A 10998T>A), Y101H (11003T>C), G102L (11006G>C 11007G>T 11008C>G), A103I (11009G>A 11010C>T), V104H (11012G>C 11013T>A), L107F (11021C>T 11023G>C), A112V (11037C>T 11038C>G), R116A (11048C>G 11049G>C), I119V (11057A>G), F120L (11060T>C 11062C>G), A121R (11063G>C 11064C>G), R130Q (11091G>A 11092C>G), V134T (11102G>A 11103T>C), K137A (11111A>G 11112A>C), E144Q (11132G>C), G145D (11136G>A), G147E (11142G>A)								
tail completion protein, type 1 (YP_010077963.1)	1	77	39.1%	340.0	60.3%	77 (100%)	43 (55.8%)	0/0/0/0	0
	L2F (11159C>T 11161G>C), T4P (11165A>C 11167T>C), A5E (11169C>A 11170C>A), A12S (11189G>A 11190C>G 11191T>C), T16L (11201A>C 11202C>T 11203G>C), T17E (11204A>G 11205C>A), F18H (11207T>C 11208T>A), G23D (11223G>A), A24G (11226C>G), D25N (11228G>A 11230C>T), E26S (11231G>A 11232A>G 11233G>C), F27A (11234T>G 11235T>C), Q28T (11237C>A 11238A>C), E30I (11243G>A 11244A>T 11245G>T), T32D (11249A>G 11250C>A 11251G>T), H34F (11255C>T 11256A>T), S38G (11267T>G 11268C>G 11269G>C), A40E (11274C>A 11275C>G), D41S (11276G>T 11277A>C 11278C>G), G47E (11295G>A 11296A>G), A48G (11298C>G 11299T>C), E50D (11305G>T), Y51L (11306T>C 11307A>T 11308T>C), A53S (11312G>T), V54I (11315G>A), D57E (11326C>G), V60Q (11333G>C 11334T>A 11335C>G), E63Q (11342G>C 11344G>A), P64E (11345C>G 11346C>A), D67E (11356T>G), L71V (11366C>G), R75A (11378C>G 11379G>C), A76E (11382C>A)								
tail sheath protein (YP_010077964.1)	312	435	28.5%	449.0	53.2%	124 (100%)	65 (52.4%)	0/0/0/0	0
	S316W (12713C>G), N317K (12717C>A), L319V (12721C>G 12723G>C), T320S (12724A>T 12726G>C), T324A (12736A>G), D325A (12740A>C 12741C>G), D327N (12745G>A), F328Y (12749T>A), F330Y (12755T>A), W331F (12758G>T 12759G>C), A334K (12766G>A 12767C>A 12768C>G), R335M (12769C>A 12770G>T), Q337E (12775C>G), A338I (12778G>A 12779C>T 12780G>C), R342S (12790C>A), V344I (12796G>A), T346N (12803C>A 12804G>C), G349T (12811G>A 12812G>C), D350S (12814G>T 12815A>C 12816C>G), Q354R (12827A>G), I358V (12838A>G), D362S (12850G>A 12851A>G), D363T (12853G>A 12854A>C 12855C>G), T366A (12862A>G), S369G (12871T>G 12872C>G 12873G>C), E371T (12877G>A 12878A>C), T373R (12883A>C 12884C>G 12885G>C), A374G (12887C>G 12888C>G), S377E (12895T>G 12896C>A), D378E (12900C>G), E380T (12904G>A 12905A>C 12906G>A), A383Q (12913G>C 12914C>A), D384T (12916G>A 12917A>C), T388A (12928A>G 12930C>T), V390I (12934G>A 12936G>T), D391E (12939C>G), Q393A (12943C>G 12944A>C), A396S (12952G>A 12953C>G), V397H (12955G>C 12956T>A), D398E (12960C>G), E399S (12961G>A 12962A>G 12963G>T), S401T (12968G>C), D402E (12972C>G), D407N (12985G>A 12987T>C), R409H (12992G>A 12993G>C), T411K (12998C>A 12999C>A), G414D (13007G>A 13008A>C), I415V (13009A>G), D416E (13014C>G), T418R (13018A>C 13019C>G), N422D (13030A>G), G423A (13034G>C), G427D (13046G>A), T429E (13051A>G 13052C>A), N431T (13058A>C), V432G (13061T>G 13062C>T), S433V (13063A>G 13064G>T 13065C>A), I434T (13067T>C 13068C>A), *435A (13069T>G 13070A>C 13071A>G)								
putative tail tube protein (YP_010077965.1)	1	133	100%	626.0	67.0%	133 (100%)	83 (62.4%)	0/0/0/0	0
	E3P (13088G>C 13089A>C), R5K (13094C>A 13095G>A), E11A (13113A>C 13114G>C), I15V (13124A>G 13126C>G), V16M (13127G>A), T26A (13157A>G), I27V (13160A>G), Y29F (13167A>T), S32E (13175A>G 13176G>A 13177C>G), R37L (13191G>T 13192A>C), E38D (13195G>C), Q39E (13196C>G), M41V (13202A>G 13204G>C), I50L (13229A>C), V51E (13233T>A 13234C>G), N52T (13236A>C 13237C>G), E54D (13243G>C), Y55I (13244T>A 13245A>T), E56S (13247G>A 13248A>G 13249A>C), V70T (13289G>A 13290T>C 13291T>G), L71M (13292C>A), E72Q (13295G>C), D73E (13300C>G), I74V (13301A>G 13303C>G), F75Y (13305T>A), Q76D (13307C>G 13309G>C), N77E (13310A>G 13312C>A), A78D (13314C>A), T80S (13320C>G 13321G>C), V85I (13334G>A 13336G>C), K86D (13337A>G 13339G>C), E89P (13346G>C 13347A>C 13348A>C), A90T (13349G>A 13351G>C), R93F (13358C>T 13359G>T), D94E (13363C>G), D95E (13366C>G), H97E (13370C>G 13372C>G), I99Y (13376A>T 13377T>A), A101C (13382G>T 13383C>G), S109A (13406T>G), Q114S (13421C>A 13422A>G 13423G>C), S116G (13427A>G 13429C>G), Y120W (13440A>G 13441C>G), W124F (13452G>T 13453G>C), T125G (13454A>G 13455C>G 13456G>C), A126C (13457G>T 13458C>G), V129A (13467T>C 13468G>C), R130E (13469C>G 13470G>A), N132E (13475A>G 13477C>G), *133C (13480A>C)								
uncharacterized protein (YP_010077966.1)	1	141	95.9%	427.0	45.0%	141 (100%)	60 (42.6%)	0/0/0/0	1

	Begin	End	Coverage	Score	Concordance	Matches	Identities	I/D/M/F*	Stop Codons
	M1R (13493T>G), T2A (13495A>G 13497A>T), E3N (13498G>A 13500G>C), E4Q (13501G>C), T5S (13504A>T), D6V (13508A>T 13509T>G), S7P (13510T>C 13512C>T), N8E (13513A>G 13515C>G), P9Q (13517C>A 13518C>A), E10V (13520A>T), S11N (13523G>A), E13Q (13528G>C), A14D (13532C>A 13533T>C), P16D (13537C>G 13538C>A 13539G>C), D17V (13541A>T), E18D (13545G>C), A19D (13547C>A), L21V (13552C>G), S22D (13555A>G 13556G>A), V24E (13562T>A 13563A>G), D25A (13565A>C), S27L (13570T>C 13571C>T), I29A (13576A>G 13577T>C 13578C>G), D30P (13579G>C 13580A>C), I31E (13582A>G 13583T>A 13584C>A), E32D (13587A>C), L34I (13591C>A), Q37L (13601A>T 13602A>G), E38Q (13603G>C), W39* (13608G>A), V48L (13633G>C 13635G>C), I49S (13636A>T 13637T>C 13638T>C), T50E (13639A>G 13640C>A 13641C>G), F51L (13642T>C), A52D (13646C>A 13647G>T), G53P (13648G>C 13649G>C), M54S (13652T>G 13653G>C), Q55E (13654C>G), F56V (13657T>G), L57D (13660C>G 13661T>A 13662C>T), V58L (13663G>C), E60Q (13669G>C), P61L (13673C>T), E64Q (13681G>C), D65E (13686C>G), L66I (13687C>A), L67R (13691T>G 13692G>T), N68G (13693A>G 13694A>G 13695C>G), M69I (13698G>C), I70A (13699A>G 13700T>C 13701C>G), A71S (13702G>T 13704C>G), G72A (13706G>C), A74M (13711G>A 13712C>T 13713C>G), G75A (13715G>C), G77Q (13720G>C 13721G>A 13722C>G), D78E (13725C>G), N80E (13729A>G 13731C>G), G81D (13733G>A), D82L (13735G>C 13736A>T), G83D (13739G>A), L84E (13741C>G 13742T>A 13743C>G), D85S (13744G>A 13745A>G), K92Q (13765A>C), L93Y (13768C>T 13769T>A 13770G>C), E95S (13774G>A 13775A>G 13776G>C), K99A (13786A>G 13787A>C), A100K (13789G>A 13790C>A 13791C>G), V103I (13798G>A), K107R (13810A>C 13811A>G 13812G>C), E110N (13819G>A 13821G>C), R112K (13825C>A 13826G>A 13827C>G), S113K (13829G>A 13830C>G), A120T (13849G>A 13851C>G), K122R (13855A>C 13856A>G), A127D (13871C>A 13872G>C), Q131H (13884G>C), M132L (13885A>C 13887G>C), V133M (13888G>A), D137G (13901A>G), A138G (13904C>G)								
uncharacterized protein (YP_010077967.1)	54	70	24.3%	59.0	57.3%	16 (94.1%)	12 (70.6%)	0/1/0/0	1
	G58S (14106G>A), Q61K (14115C>A), R62H (14119G>A 14120A>C), T64R (14124A>C 14125C>G), N69del (14139_14141delAAC)								
tape-measure tail protein (YP_010077968.1)	1	476	48.5%	1743.0	63.6%	444 (100%)	276 (62.2%)	0/0/0/0	0
	S3G (14153T>G 14154C>G 14155T>A), S5N (14160G>A), A6V (14163C>T 14164A>C), Q16S (14192C>A 14193A>G 14194G>C), N17D (14195A>G 14197C>T), N18Q (14198A>C 14200C>A), S20T (14205G>C 14206C>G), D22K (14210G>A 14212C>A), R24D (14216C>G 14217G>A 14218G>T), D25R (14219G>C 14220A>G 14221C>A), V26A (14223T>C 14224C>A), R28Q (14229G>A), A33V (14244C>T 14245A>C), A36S (14252G>T), E38D (14260G>C), G39Q (14261G>C 14262G>A 14263C>G), T40A (14264A>G), Q42S (14270C>T 14271A>C 14272A>G), Q43R (14274A>G 14275G>A), R45D (14279C>G 14280G>A 14281G>C), T46E (14282A>G 14283C>A 14284C>G), N49G (14291A>G 14292A>G 14293C>G), S50T (14294T>A 14296A>G), F52L (14300T>C 14302T>G), A53K (14303G>A 14304C>A), A54T (14306G>A 14308C>G), T59S (14321A>T 14323C>G), S64T (14336T>A 14338A>G), L65M (14339C>A 14341T>G), T66A (14342A>G), V69T (14351G>A 14352T>C 14353C>G), Q71R (14358A>G 14359G>C), R78T (14378C>A 14379G>C), Q83G (14393C>G 14394A>G 14395G>T), T84V (14396A>G 14397C>T 14398G>C), T86A (14402A>G), Q91D (14417C>G 14419G>C), L93M (14423C>A 14425C>G), S94A (14426T>G 14428C>G), E98D (14440A>T), T102E (14450A>G 14451C>A 14452T>G), L103M (14453T>A), R105V (14459C>G 14460G>T 14461G>C), D106E (14464T>G), D116N (14492G>A), N120Q (14504A>C 14506T>G), F126Y (14523T>A 14524T>C), S127E (14525T>G 14526C>A 14527C>G), K129E (14531A>G), M132V (14540A>G 14542G>C), S133K (14543T>A 14544C>A), E136N (14552G>A 14554G>C), S142A (14570T>G 14572G>C), T146N (14583C>A 14584G>C), M147L (14585A>C), D148N (14588G>A), A150S (14594G>A 14595C>G 14596G>C), A156T (14612G>A), G157A (14616G>C 14617A>G), N168E (14648A>G 14650T>G), E169Q (14651G>C), V207I (14765G>A), S208Q (14768T>C 14769C>A 14770C>G), L209M (14771C>A 14773C>G), T212V (14780A>G 14781C>T 14782G>C), S213A (14783A>G 14784G>C), I216V (14792A>G), Q227S (14825C>T 14826A>C 14827G>T), T235A (14849A>G 14851C>G), T238Q (14858A>C 14859C>A), L240I (14864T>A 14866G>C), M241T (14868T>C), G242T (14870G>A 14871G>C), S245G (14879A>G), Q248E (14888C>G), G249A (14892G>C 14893A>C), D252E (14902C>A), E253R (14903G>C 14904A>G), V256L (14912G>C 14914C>G), E257T (14915G>A 14916A>C 14917G>C), M258T (14919T>C 14920G>C), D259E (14923C>G), D260E (14926C>G), L261F (14927C>T), T262L (14930A>C 14931C>T), Q264E (14936C>G), F268I (14948T>A), R269Q (14952G>A 14953C>G), D270N (14954G>A 14956T>C), N272E (14960A>G 14962C>G), D273T (14963G>A 14964A>C 14965C>G), V274M (14966G>A), I275L (14969A>C), R276T (14972C>A 14973G>C), V277T (14975G>A 14976T>C), L278I (14978C>A), A279G (14982C>G), D280E (14986C>G), G281E (14988G>A 14989C>G), F282M (14990T>A 14992C>G), E283Q (14993G>C), G287N (15005G>A 15006G>A 15007T>C), Q288D (15008C>G 15010G>C), V292L (15020G>C), R293Q (15024G>A 15025C>G), E297D (15037A>C), Q304A (15056C>G 15057A>C 15058G>C), E312D (15082G>C), S313N (15083T>A 15084C>A 15085T>C), Q314T (15086C>A 15087A>C 15088G>C), E323D (15115G>C), K330R (15134A>C 15135A>G), A335E (15150C>A 15151G>A), N338R (15158A>C 15159A>G 15160C>G), D340S (15164G>T 15165A>C 15166C>G), A341D (15168C>A), Q343E (15173C>G), I344L (15176A>C), E345S (15179G>T 15180A>C), G346N (15182G>A 15183G>A), D352G (15201A>G 15202C>G), M353T (15204T>C 15205G>C), P357R (15216C>G 15217G>C), Q359D (15221C>G 15223G>C), V360F (15224G>T 15226G>C), T361A (15227A>G 15229A>G), R363E (15233C>G 15234G>A 15235A>G), E364D (15238G>C), I365L (15239A>C), D367T (15245G>A 15246A>C), R368S (15248C>T 15249G>C 15250G>C), M369L (15251A>C 15253G>C), Q370E (15254C>G), Q371E (15257C>G), L372M (15260C>A 15262C>G), R373N (15263C>A 15264G>A), E375Q (15269G>C), E377A (15276A>C), S378T (15279G>C 15280C>G), D381E (15289C>G), V382M (15290G>A 15292C>G), A384Q (15296G>C 15297C>A 15298T>G), Q387A (15305C>G 15306A>C), A388I (15308G>A 15309C>T 15310A>C), G389A (15312G>C 15313A>G), G394D (15327G>A), E398K (15338G>A), T399A (15341A>G), F400L (15344T>C), R402E (15350C>G 15351G>A 15352C>G), L404V (15356C>G), D405Q (15359G>C 15361C>G), A407S (15365G>T), S411A (15377T>G), D413E (15385T>G), A425S (15419G>A 15420C>G), D426E (15424C>G), D433N (15443G>A), S436A (15452T>G), A438M (15458G>A 15459C>T 15460C>G), F451W (15498T>G 15499C>G), K455T (15510A>C 15511G>C), Q462S (15530C>A 15531A>G 15532G>C), R467Q (15546G>A), G470T (15554G>A 15555G>C), A471G (15558C>G)								
uncharacterized protein (YP_010077973.1)	3	78	41.3%	320.0	65.6%	76 (100%)	46 (60.5%)	0/0/0/0	0
	V6E (18800T>A), Q7S (18802C>T 18803A>C 18804G>T), A8G (18806C>G), L11D (18814C>G 18815T>A 18816G>T), F12L (18817T>C 18819C>G), K14Q (18823A>C), Q15D (18826C>G 18828G>C), V17L (18832G>C 18834C>G), I20V (18841A>G), V23A (18851T>C 18852C>A), S24T (18853T>A), V27R (18862G>C 18863T>G 18864C>G), V28I (18865G>A), A30R (18871G>C 18872C>G), D34S (18883G>T 18884A>C 18885C>G), E35N (18886G>A 18888A>C), E39T (18898G>A 18899A>C 18900G>C), L42K (18907C>A 18908T>A 18909G>A), T44S (18914C>G), E46S (18919G>T 18920A>C), N47D (18922A>G), D51E (18936C>G), A61T (18964G>A 18966C>G), A64G (18974C>G 18975C>A), M66L (18979A>C 18981G>C), V68A (18986T>C 18987T>G), V70I (18991G>A), G71S (18994G>T 18995G>C 18996C>A), G73E (19001G>A 19002C>A)								
transposase (YP_010077984.1)	4	381	90.2%	1612.0	56.7%	376 (97.2%)	212 (54.8%)	9/2/0/0	0

	Begin	End	Coverage	Score	Concordance	Matches	Identities	I/D/M/F*	Stop Codons
	T12Y (27420T>A 27421G>T 27422T>A), A18Q (27402G>C 27403G>T 27404C>G), E21Q (27395C>G), C25H (27381A>G 27382C>T 27383A>G), D26N (27380C>T), L27E (27376A>T 27377G>C), L28H (27373A>T), E29G (27369T>G 27370T>C), T30R (27366G>C 27367G>C 27368T>G), R32D (27360A>G 27361C>T 27362G>C), E33T (27357C>A 27358T>G 27359C>T), G34L (27354C>G 27355C>A 27356C>G), H36R (27348G>A 27349T>C), A38S (27342C>A 27343G>C 27344C>T), D40N (27338C>T), A41N (27334G>T 27335C>T), G42E (27331C>T), T49V (27310G>A 27311T>C), S50N (27307C>T), D51E (27303G>C), V52L (27300C>A 27302C>G), T54S (27294C>G 27295G>C), P55G (27291G>A 27292G>C 27293G>C), Y56E (27288G>T 27290A>C), P59N (27279C>G 27280G>T 27281G>T), Y60W (27276A>C 27277T>C), Q61H (27273C>A), A62S (27270C>G 27272C>A), A64N (27264T>A 27265G>T 27266C>T), C67K (27255G>T 27256C>T 27257A>T), N68K (27252G>T), Q72D (27240T>G 27242G>C), H74L (27235T>A), D75_T76insEE (27230_27231insCTCCTC), N78D (27224T>C), Q80N (27216C>A 27218G>T), E81R (27214T>C 27215C>G), D83A (27207G>T 27208T>G), D85T (27202T>G 27203C>T), V88I (27192A>G 27194C>T), L90Y (27186A>G 27187A>T 27188G>A), T91A (27185T>C), Q93T (27177T>G 27178T>G 27179G>T), E96V (27168C>G 27169T>A), S100D (27156C>A 27157G>T 27158A>C), A101E (27153C>T 27154G>T), A102G (27150C>G 27151G>C), D104H (27146C>G), Y105H (27143A>G), F107I (27137A>T), T108C (27132T>G 27133G>C 27134T>A), W110E (27126C>T 27127C>T 27128A>C), A111V (27123T>G 27124G>A), Q113L (27117T>A 27118T>A), R125Q (27081A>C 27082C>T), I126V (27078G>C 27080T>C), D131Q (27063G>T 27065C>G), G132D (27060A>G 27061C>T), L133W (27058A>C 27059G>A), D136A (27048G>T 27049T>G), V138I (27042T>G 27044C>T), H139S (27040T>G 27041G>A), G140E (27036A>C 27037C>T), E141_A142insDDS (27032_27033insGCTGTCGTC), A142V (27030C>T 27031G>A), S143W (27028G>C), H152D (27002G>C), R153G (26999G>C), T154S (26994C>A 26995G>C), S155R (26991C>T 26992G>C 26993A>G), T157V (26986G>A 26987T>C), V162A (26970G>T 26971A>G), E163N (26967C>G 26969C>T), F164Y (26965A>T), P165E (26961C>T 26962G>T 26963G>C), Q168del (26952_26954delTTG), Y171H (26943A>G 26945A>G), E172S (26941T>G 26942C>A), P173C (26937C>A 26938G>C 26939G>A), T174S (26934G>C 26936T>A), D175S (26932T>G 26933C>A), E176_D177insSD (26927_26928insGTCGCT), I185V (26901A>C 26903T>C), A188S (26892C>G 26894C>A), H189K (26889G>T 26891G>T), A192V (26881G>A), C196L (26869C>A 26870A>G), K197Q (26865C>T 26867T>G), Q198D (26862C>G 26864G>C), G199D (26860C>T), T202V (26851G>A 26852T>C), L206F (26840G>A), I207V (26835G>C 26837T>C), N208D (26832G>A 26834T>C), A212V (26820A>G 26821G>A), K217Q (26805T>C 26807T>G), E218K (26802C>T 26804C>T), M219Q (26799C>T 26800A>T 26801T>G), F220A (26797A>G 26798A>C), T221S (26793T>A 26794G>C), L223E (26787C>T 26788A>T 26789G>C), K224D (26784C>G 26786T>C), Q227K (26777G>T), E228Q (26774C>G), D230Y (26768C>A), A232S (26760G>C 26762C>A), Q233T (26757C>G 26758T>G 26759G>T), W234del (26754_26756delCCA), R235L (26751C>G 26752C>A), I236L (26748A>C 26750T>A), R239L (26739T>G 26740C>A), R239_F240insV (26738_26739insCAC), F240W (26736G>C 26737A>C), D241G (26733G>T 26734T>C), H242R (26730G>C 26731T>C), Y243W (26727G>C 26728T>C), L247I (26717G>T), T248E (26713G>T 26714T>C), I250E (26706G>T 26707A>T 26708T>C), R256T (26688C>T 26689C>G 26690G>T), Q257R (26685T>C 26686T>C), I259V (26681T>C), A262T (26670G>C 26672C>T), C263S (26668C>G), R264Q (26664T>C 26665C>T), K267N (26655C>G), V270I (26648C>T), S276K (26629G>T 26630A>T), Y277D (26627A>C), E280D (26616T>G), D281N (26615C>T), L282M (26610G>C 26612G>T), E286K (26598T>C 26600C>T), W287Y (26595C>G 26596C>T), A294G (26575G>C), A296G (26569G>C), R299K (26559G>C 26560C>T 26561G>T), Q301H (26553C>G), E302A (26550C>T 26551T>G), R303H (26547A>G 26548C>T), E305P (26541C>G 26542T>G 26543C>G), D306Y (26540C>A), R309A (26529T>G 26530C>G 26531G>C), A311K (26523A>C 26524G>T 26525C>T), Y317T (26505G>C 26506T>G 26507A>T), I318V (26502A>C 26504T>C), R319N (26499G>A 26500C>T 26501G>T), E321Q (26495C>G), Q325K (26483G>T), T326P (26480T>G), E329A (26469C>A 26470T>G), H332E (26460G>T 26462G>C), I333H (26458A>T 26459T>G), D337P (26445G>T 26446T>G 26447C>G), G338R (26442G>C 26444C>G), D339Q (26439A>C 26441C>G), E340A (26437T>G), E340_F341insT (26435_26436insCGT), Q344S (26425T>G 26426G>A), E347D (26415C>G), V350L (26406T>G 26408C>G), S351G (26403C>A 26404G>C 26405A>C), H354Q (26394G>T), I357V (26385G>C 26387T>C), A359G (26380G>C), V361I (26373G>T 26375C>T), H367L (26355G>C 26356T>A), D368N (26352G>A 26354C>T), L376W (26329A>C 26330G>A), P378S (26324G>A)								
uncharacterized protein (YP_010077992.1)	1	82	100%	237.0	47.8%	79 (95.2%)	48 (57.8%)	1/3/1/1	1
	N3S (32346A>G), V4I (32348G>A 32350C>T), S7N (32357T>A 32358C>A), D10N (32366G>A), S11D (32369T>G 32370C>A 32371G>C), D14E (32380C>G), Q15R (32382A>G 32383G>C), D17E (32389C>A), D18K (32390G>A 32392C>G), A28T (32420G>A 32422T>G), R33Q (32436G>A 32437C>A), R37E (32447C>G 32448G>A 32449C>G), D38T (32450G>A 32451A>C), Q44E (32468C>G 32470G>A), V46T (32474G>A 32475T>C 32476C>T), E47N (32477G>A 32479G>T), G48E (32481G>A 32482C>G), E54S (32498G>A 32499A>G 32500G>C), D57E (32509C>G), L59I (32513C>A 32515G>C), Q62K (32522C>A 32524G>A), I63V (32525A>G), D64N (32528G>A 32530C>T), R65E (32531C>G 32532G>A), T68S (32541C>G), E69A (32544A>C 32545G>T), E69_E70insX (32545_32546insC), A71V (32550C>T), A73D (32556C>A), D74E (32560T>A), D76_P78del (32564_32572delGATGACCCG), A79T (32573G>A), A81E (32580C>A)								
PIN domain-containing protein (YP_010077993.1)	1	104	72.7%	370.0	57.2%	104 (100%)	54 (51.9%)	0/0/0/0	0
	Y3L (32587T>C 32588A>T 32589C>G), V5I (32593G>A), I9V (32605A>G 32607A>C), I10V (32608A>G 32610C>T), L11I (32611C>A), G13A (32618G>C), S16A (32626T>G), M19K (32636T>A 32637G>A), R22E (32644C>G 32645G>A), L24I (32650C>A), E26T (32656G>A 32657A>C), D29P (32665G>C 32666A>C), Y36F (32687A>T), E40G (32699A>G 32700G>A), G42E (32705G>A), K43N (32709G>C), T45E (32713A>G 32714C>A 32715C>G), M47L (32719A>C), V48I (32722G>A), S49V (32725T>G 32726C>T), L54M (32740C>A 32742C>G), S55E (32743T>G 32744C>A 32745G>A), A57D (32750C>A 32751G>T), E58R (32752G>C 32753A>G 32754G>A), E60A (32759A>C), E61Q (32761G>C), L62F (32764C>T 32766C>T), E64D (32772G>T), M65L (32773A>C 32775G>T), K68Q (32782A>C), R69Y (32785C>T 32786G>A 32787T>C), H71N (32791C>A 32793C>T), R75A (32803C>G 32804G>C), P76S (32806C>A 32807C>G 32808G>T), V77E (32810T>A), V78F (32812G>T), L79H (32816T>A), E80P (32818G>C 32819A>C), S81A (32821T>G 32823C>T), L82I (32824C>A), Q83E (32827C>G), E84R (32830G>A 32831A>G 32832G>A), A86G (32837C>G 32838G>T), R87E (32839C>G 32840G>A 32841G>A), I88A (32842A>G 32843T>C 32844T>A), M89I (32847G>C), R90G (32848C>G 32850C>A), D91E (32853C>G), A97V (32870C>T 32871G>A), V98L (32872G>C)								
uncharacterized protein (YP_010077994.1)	67	116	42.7%	317.0	88.8%	50 (100%)	40 (80.0%)	0/0/0/0	0
	T77S (33326A>T 33328G>A), S80T (33336G>C 33337C>G), V81I (33338G>A), V82T (33341G>A 33342T>C), V83I (33344G>A 33346G>C), S88D (33359T>G 33360C>A 33361G>C), Y94W (33378A>G 33379C>G), S100D (33395A>G 33396G>A), V102I (33401G>A 33403G>C)								
uncharacterized protein (YP_010077997.1)	6	75	38.3%	264.0	66.0%	70 (97.2%)	41 (56.9%)	2/0/0/0	0
	G9E (35030C>T), A12T (35022C>T), K14E (35016T>C), E18K (35004C>T), A20G (34996G>C 34997G>C), D21E (34993G>C), I23M (34987G>C), S31G (34963A>G 34965T>C), Q32A (34960C>T 34961T>G 34962G>C), S33A (34959A>C), S34T (34954G>T 34955C>G), E35D (34951T>G), A40S (34938C>A), S43G (34929T>C), E44N (34924C>G 34926C>T), A45T (34923C>T), A48T (34914C>T), T52I (34901G>A), E53D (34897T>G), T55S (34892G>C), T55_E56insGS (34890_34891insGCTCCC), E56A (34888T>C 34889T>G), T57S (34885C>G 34887T>A), E59P (34880T>G 34881C>G), T60S (34876C>A 34878T>A), E62D (34870T>A), T63M (34868G>A), A67T (34857C>T), K68E (34852T>C 34854T>C), S71T (34843G>T 34845A>T)								
plasmid partition protein (YP_010077998.1)	9	222	75.1%	796.0	56.3%	214 (99.5%)	143 (66.5%)	1/0/2/2	0
	A10T (35875C>T), T11S (35871G>C), A13T (35866C>T), T14I (35862G>A), T17S (35852G>C 35854T>A), G18P (35849G>C 35850C>G 35851C>G), S23A (35834A>C 35836A>C), V38I (35791C>T), N45A (35768G>T 35769T>G 35770T>C), G46D (35765G>A 35766C>T), H49L (35757T>A), D50N (35755C>T), F68Y (35700A>T), D70T (35694T>G 35695C>T), V71A (35690G>C 35691A>G), G73S (35684G>A 35686C>T), L77R (35672C>G 35673A>C), D78E (35669G>C), F79V (35666G>C 35668A>C), T80S (35663T>C 35665T>A), E87D (35642C>A), N89S (35637T>C), Q90R (35634T>C), Y91A (35631T>G 35632A>C), G92A (35628C>G), D93E (35624G>C), R94V (35621G>A 35622C>A 35623G>C), A95G (35618C>A 35619G>C), H96D (35615G>A 35617G>C), E97D (35612C>G), Q100L (35603C>A 35604T>A), D101A (35600G>C 35601T>G), A103E (35594C>T 35595G>T), I107L (35582G>T 35584T>G), H111N (35572G>T), N117R (35552G>C 35553T>C 35554T>G), A119G (35546C>A 35547G>C), S120G (35543G>T 35545T>C), A121E (35540C>T 35541G>T), D123N (35534G>A 35536C>T), G133K (35505C>T 35506C>T), A135V (35498G>C 35499G>A), E137D (35492C>G), D138E (35489G>C), R140G (35485G>C), Q141W (35481T>C 35482G>A), R142W (35479G>A), R142_H143insX (35476_35477insG), H143Y (35476G>A), I148V (35461T>C), E151M (35451T>A 35452C>T), Q163V (35414C>A 35415T>A 35416G>C), N164A (35412T>G 35413T>C), A167G (35402C>G 35403G>C), S172A (35387C>T 35389A>C), F182L (35357G>A 35359A>G), Q190D (35333C>A 35335G>C), I192V (35327A>G 35329T>C), E193R (35324C>A 35325T>C 35326C>G), S194E (35321G>C 35322G>T 35323A>C), Y196F (35316T>A), P198T (35309C>T 35311G>T), E200R (35303C>A 35304T>C 35305C>G), V205I (35290C>T), V206A (35286A>G), I209V (35278T>C), Q211H (35270C>G), N213K (35264G>T), V219L (35248C>G), E220D (35243C>G)								
uncharacterized protein (YP_010078000.1)	1	594	100%	3577.0	93.1%	593 (99.8%)	540 (90.9%)	0/1/0/0	1

	Begin	End	Coverage	Score	Concordance	Matches	Identities	I/D/M/F*	Stop Codons
	S2T (38238A>T), N3D (38235T>C), T5del (38227_38229delTGT), K8H (38218C>G 38220T>G), T9S (38217T>A), Q26A (38164C>T 38165T>G 38166G>C), V40M (38122G>C 38124C>T), A72V (38027G>A), A95G (37958G>C), L102I (37936C>G 37938G>T), V106T (37925A>G 37926C>T), V117I (37893C>T), E122D (37876C>G), E125Q (37869C>G), E126D (37864C>G), A138V (37828C>G 37829G>A), A151S (37789C>G 37790G>C 37791C>T), A164V (37750C>A 37751G>A), M217V (37593T>C), S226A (37566A>C), R228K (37559C>T 37560G>T), G252A (37487C>G), K273T (37424T>G), T278R (37408T>C 37409G>C 37410T>G), E294D (37360C>G), Q295E (37357C>T 37359G>C), D298N (37350C>T), A365D (37148G>T), Q394E (37062G>C), E408A (37019T>G), G411E (37010C>T), Q414K (37002G>T), A418T (36990C>T), G433S (36945C>T), V449A (36896A>G), A455T (36877C>G 36879C>T), E456D (36874C>G), V457A (36872A>G), G459T (36866C>G 36867C>T), A469S (36837C>A), V515I (36697C>G 36699C>T), S530G (36654T>C), S541G (36621T>C), D545E (36607G>C), Q546N (36604C>G 36606G>T), K553N (36583C>G), G570A (36533C>G), N571A (36530T>G 36531T>C), A577T (36513C>T), I579V (36505A>G 36507T>C), Q584E (36492G>C), S588A (36480A>C), S592A (36468A>C), N593D (36465T>C)								
uncharacterized protein (YP_010078001.1)	205	205	0.5%	1.0	14.3%	1 (100%)	1 (100%)	0/0/0/0	1
CxxC motif protein (YP_010078025.1)	36	210	79.5%	878.0	66.6%	175 (99.4%)	120 (68.2%)	1/0/1/1	0
	I40R (52049A>C 52050T>G), Q41H (52054A>C), L42I (52055C>A), P43H (52059C>A), R54G (52091C>G 52093C>G), R55E (52094C>G 52095G>A 52096A>G), I56V (52097A>G), K58G (52103A>G 52104A>G 52105G>A), K59E (52106A>G), V60I (52109G>A), E63G (52119A>G 52120G>A), H66R (52128A>G 52129C>T), Q73R (52149A>G 52150A>C), R74L (52152G>T 52153C>G), R77H (52161G>A 52162G>T), Q79E (52166C>G), E80D (52171G>C), V85I (52184G>A 52186C>T), R89H (52197G>A), H90G (52199C>G 52200A>G 52201C>A), N91D (52202A>G), V92I (52205G>A 52207A>C), D99E (52228T>A), S104P (52241T>C 52243G>C), E105R (52244G>C 52245A>G 52246A>C), Y108R (52253T>C 52254A>G 52255C>T), L109V (52256C>G), K118R (52283A>C 52284A>G), L119E (52286C>G 52287T>A 52288C>A), E120R (52289G>C 52290A>G), E121D (52294G>T), G124A (52302G>C), R125S (52304C>T 52305G>C 52306C>A), T126V (52307A>G 52308C>T 52309G>T), D129E (52318C>G), A130E (52320C>A), G133_T134insX (52330_52331insG), T134S (52331A>T 52333G>C), D143S (52358G>A 52359A>G), H145N (52364C>A 52366T>C), S149P (52376T>C), T150P (52379A>C 52381C>A), E154D (52393A>C), D159V (52407A>T), T164S (52422C>G), Y170F (52440A>T), V172I (52445G>A), D173A (52449A>C 52450T>G), Q189T (52496C>A 52497A>C), P190S (52499C>T 52501G>C), T192L (52505A>C 52506C>T 52507G>A), N195D (52514A>G), K201R (52532A>C 52533A>G 52534G>C), A203G (52539C>G 52540C>G), V208I (52553G>A)								

*: Inserts / Deletes / Misaligned / Frameshifts

Detailed Results of Betapleolipovirus HRPV9 (NC_048160.1)

NGS details

Assembly

**Coverage length**	3411 (7 contig(s))
**Est. depth of coverage**	59.1
**Est. number of reads**	2387
**Ambiguities**	0

??coverage-details?? ??variant-analysis??

Assignment

**Type**	Betapleolipovirus HRPV9 (Taxonomy ID: 2734333)
**Reference Genome**	NC_048160.1 (Length: 16159bp)
**NT Identity (%)**	79.2785
**NT Quality**	1.10847

Alignment

**Alignment score**	3781 (NT) + 5923 (AA) = 9704
**Concordance (%)**	69.654
**Alignment method**	Global, seeded, nucleotide + amino acids (AGA)

Genome Region

Sequence starts at position null and ends at position null relative to the null reference sequence for null.

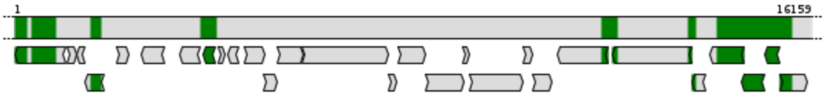

Alignment Detailed Statistics

	Begin	End	Coverage	Score	Concordance	Matches	Identities	I/D/M/F*	Stop Codons
**NT**	1	15791	21.1%	3781.0	57.6%	3347 (97.9%)	2659 (77.8%)	7/64	
**CDS**									
HOV17_gp01	56	341	75.1%	1655.0	93.2%	256 (100%)	238 (93.0%)	0/0/0/0	1
HOV17_gp04	25	102	58.2%	481.0	70.2%	78 (100%)	49 (62.8%)	0/0/0/0	0
HOV17_gp08	1	86	100%	385.0	67.1%	86 (98.9%)	58 (66.7%)	1/0/0/0	1
HOV17_gp22	1	48	13.8%	75.0	24.3%	48 (100%)	27 (56.3%)	0/0/1/1	1
HOV17_gp23	1	539	12.2%	407.0	87.3%	66 (98.5%)	56 (83.6%)	1/0/0/0	1
HOV17_gp24	62	91	33.0%	181.0	87.4%	29 (96.7%)	25 (83.3%)	0/1/0/0	1

	Begin	End	Coverage	Score	Concordance	Matches	Identities	I/D/M/F*	Stop Codons
HOV17_gp25	1	187	80.3%	1002.0	78.5%	174 (93.0%)	142 (75.9%)	0/13/1/1	0
HOV17_gp26	1	159	100%	950.0	87.3%	159 (100%)	133 (83.6%)	0/0/0/0	1
HOV17_gp27	1	103	100%	477.0	74.6%	102 (99.0%)	71 (68.9%)	0/1/0/0	1
HOV17_gp28	1	83	45.6%	310.0	57.5%	83 (100%)	49 (59.0%)	0/0/0/0	0
**Proteins**									
site-specific integrase (YP_009819997.1)	56	341	75.1%	1655.0	93.2%	256 (100%)	238 (93.0%)	0/0/0/0	1
	D57N (855C>T), L61F (843G>A), N63E (835G>C 837T>C), S111T (691G>C 693A>T), E121D (661T>A), A124E (652C>T 653G>T), G126A (647C>G), E127A (643T>C 644T>G), E129D (637C>G), L166V (526T>C 528A>C), I200V (426T>C), E202D (418T>A), K314D (82C>G 84T>C), T318E (70C>T 71G>T 72T>C), T333R (26G>C 27T>G), Y335H (21A>G), D339E (7A>C), F340I (4G>A 6A>T)								
hypothetical protein (YP_009820000.1)	25	102	58.2%	481.0	70.2%	78 (100%)	49 (62.8%)	0/0/0/0	0
	Q28H (1762C>G), C31S (1753G>A 1754C>G), P32A (1752G>C), R33Y (1747A>G 1748C>T 1749G>A), L34K (1745A>T 1746G>T), L36F (1738A>G 1740G>A), L37V (1737G>C), L39Y (1729T>A 1730A>T), T40I (1727G>A), V42A (1720G>A 1721A>G), S45R (1711C>A 1712G>C 1713A>G), E46D (1708T>G), S47V (1706G>A 1707A>C), P50S (1696C>T 1698G>A), V53L (1687A>G 1689C>G), D58N (1674C>T), R59E (1670C>T 1671G>C), Q60L (1666C>G 1667T>A), F65Y (1652A>T), R69L (1640C>A), S73T (1629A>T), K85H (1591T>G 1593T>G), A88R (1582C>G 1583G>C 1584C>G), S89R (1579G>C 1581T>G), D90N (1578C>T), Y92V (1571T>A 1572A>C), V95A (1562A>G), Q97Y (1555C>G 1557G>A), T98K (1553G>T)								
hypothetical protein (YP_009820004.1)	1	86	100%	385.0	67.1%	86 (98.9%)	58 (66.7%)	1/0/0/0	1
	R3D (4084A>G 4085C>T 4086G>C), S6T (4076C>G), K8R (4069C>T 4070T>C), I9L (4066G>C 4068T>G), T10S (4065T>A), E14K (4051T>C 4053C>T), G18E (4039T>C 4040C>T), S21T (4031C>G), N29D (4008T>C), I46V (3955A>C 3957T>C), Q47E (3952T>C 3954G>C), A50E (3943T>C 3944G>T), E54T (3931C>G 3932T>G 3933C>T), D57E (3922G>C), I59Y (3916T>G 3917A>T 3918T>A), D60P (3913A>T 3914T>G 3915C>G), P61_T62insG (3909_3910insGCC), T62E (3908G>T 3909T>C), I63V (3904T>C 3906T>C), I64K (3901A>C 3902A>T), Q65D (3898C>G 3900G>C), R66C (3895A>G 3897G>A), F67C (3892G>A 3893A>C), D70S (3884T>G 3885C>A), G73Q (3874T>C 3875C>T 3876C>G), R75K (3868T>C 3869C>T 3870G>T), Q77R (3863T>C), R79S (3856T>C 3857C>G 3858T>A), S82Q (3847G>C 3848C>T 3849T>G)								
hypothetical protein (YP_009820018.1)	1	48	13.8%	75.0	24.3%	48 (100%)	27 (56.3%)	0/0/1/1	1
	V1A (12053A>G), S2G (12051T>C), S3R (12047G>C 12048A>G), R4P (12044C>G), P8S (12033G>A), Q9R (12028T>G 12029T>C), H10P (12025G>T 12026T>G), K11* (12024T>A), R12P (12020C>G), S14G (12014G>C 12015A>C), G15D (12010T>G 12011C>T), L19P (11999A>G), L20R (11995G>T 11996A>C), D22G (11989G>T 11990T>C), A23L (11987G>A 11988C>G), E24G (11984T>C), P25S (11982G>A), L31S (11963A>G), A32H (11959C>G 11960G>T 11961C>G), D41Y (11934C>A)								
hypothetical protein (YP_009820019.1)	1	539	12.2%	407.0	87.3%	66 (98.5%)	56 (83.6%)	1/0/0/0	1
	S18_G19insD (13695_13696insATC), A22P (13686C>G), E511D (12217T>G), V513E (12211G>C 12212A>T), S514T (12210A>T), A519D (12193T>G 12194G>T), S520A (12190A>C 12192A>C), V527A (12170A>G), S529G (12165T>C), K533R (12152T>C), R538G (12136C>G 12138G>C)								
hypothetical protein (YP_009820020.1)	62	91	33.0%	181.0	87.4%	29 (96.7%)	25 (83.3%)	0/1/0/0	1
	V66L (13821A>C 13823C>G), R67P (13819C>G), L68I (13817G>T), I80V (13781T>C), S88del (13755_13757delGCT)								
hypothetical protein (YP_009820021.1)	1	187	80.3%	1002.0	78.5%	174 (93.0%)	142 (75.9%)	0/13/1/1	0
	T3A (14797T>C), P6R (14787G>C), E8R (14780C>G 14781T>C 14782C>G), R9G (14779G>C), A12P (14770C>G), L13V (14767A>C), T14I (14763G>A), R15_I17del (14753_14761delGATACGACG), E36Q (14698C>G), T38V (14690A>G 14691G>A 14692T>C), S45E (14669T>C 14670G>T 14671A>C), V53I (14645G>A 14647C>T), G55A (14639G>C 14640C>G), V65E (14610A>T), P74K (14583G>T 14584G>T), P83_H92del (14528_14558delGTGTGATTCGGGATCGCCTCCACCGCCAGGC), Y95Q (14519A>C 14521A>G), G97P (14513A>G 14514C>G 14515C>G), D101R (14502T>C 14503C>G), A102V (14498C>G 14499G>A), V111I (14473C>T), R116P (14456C>A 14457C>G), S127N (14424G>T 14425A>T), S144T (14374A>T), S146P (14368A>G), V149R (14357C>G 14358A>C 14359C>G), S152Q (14349G>T 14350A>G), W156F (14336C>G 14337C>A), T159D (14327C>G 14328G>T 14329T>C), A160R (14324C>A 14325G>C 14326C>G), N177T (14274T>G), E178D (14270C>A), G187S (14245C>T)								
hypothetical protein (YP_009820022.1)	1	159	100%	950.0	87.3%	159 (100%)	133 (83.6%)	0/0/0/0	1
	A4S (15231C>A), S5T (15227C>G), P7S (15220G>C 15222G>A), T34A (15141T>C), V39A (15125A>G), L46V (15105G>C), M48V (15099T>C), Q51P (15089T>G), G64E (15049G>C 15050C>T), A77G (15011G>C), R80Q (15001G>C 15002C>T), E89K (14976C>T), E92D (14965C>G), L93R (14963A>C), D111A (14908G>A 14909T>G), I119V (14886T>C), V124I (14871C>T), G126D (14864C>T), V129I (14856C>T), I130T (14851G>C 14852A>G), A131S (14850C>A), S143T (14812C>G 14814A>T), V145L (14806G>C 14808C>G), H152D (14787G>C), S154A (14779G>C 14780C>G 14781T>C), E157A (14770C>G 14771T>G)								
hypothetical protein (YP_009820023.1)	1	103	100%	477.0	74.6%	102 (99.0%)	71 (68.9%)	0/1/0/0	1
	M1V (15545T>C), G3P (15538C>G 15539C>G), S6R (15528A>G 15530T>G), S15T (15501C>G 15503A>T), T22A (15482T>C), L26G (15469A>C 15470G>C), V31A (15454A>G), P33V (15447C>G 15448G>A 15449G>C), S34D (15445C>T 15446T>C), I40V (15428T>C), E43G (15417C>T 15418T>C), A46E (15409G>T), M47L (15405C>G 15407T>G), A52T (15392C>T), V54L (15386C>G), T55V (15381C>G 15382G>A 15383T>C), V58A (15372C>G 15373A>G), T59A (15371T>C), G61T (15364C>G 15365C>T), G65L (15351A>G 15352C>A 15353C>G), V69T (15340A>G 15341C>T), E75D (15321C>A), T76V (15318C>G 15319G>A 15320T>C), P77S (15315C>G 15317G>A), E78P (15313T>G 15314C>G), L82F (15302G>A), I84V (15296T>C), T86V (15289G>A 15290T>C), A90V (15277G>A), V97del (15255_15257delCAC), V100I (15246C>G 15248C>T), F101L (15245A>G)								

	Begin	End	Coverage	Score	Concordance	Matches	Identities	I/D/M/F*	Stop Codons
hypothetical protein (YP_009820024.1)	1	83	45.6%	310.0	57.5%	83 (100%)	49 (59.0%)	0/0/0/0	0
	M1T (15545T>C), T3I (15551C>T), S5P (15556T>C), A10V (15572C>T), A11P (15574G>C), A14V (15584C>T), K15E (15586A>G), R16P (15590G>C), R17P (15593G>C), R18H (15596G>A), V21G (15605T>G), T22L (15607A>C 15608C>T), A23V (15611C>T), D24G (15614A>G), P25R (15617C>G), V26G (15620T>G), R34H (15644G>A), G38A (15656G>C 15657C>G), Q42E (15667C>G), R50A (15691C>G 15692G>C), A52P (15697G>C), G53R (15700G>C), G56R (15709G>C), R58W (15715C>T), V62R (15727G>C 15728T>G 15729C>G), D63R (15730G>C 15731A>G), F65L (15736T>C), S66P (15739T>C), R68G (15745C>G), A69T (15748G>A), S70P (15751T>C), A72T (15757G>A), D75N (15766G>A)								

*: Inserts / Deletes / Misaligned / Frameshifts

Detailed Results of Betapleolipovirus HRPV3 (NC_017088.1)

NGS details

Assembly

**Coverage length**	4005 (4 contig(s))
**Est. depth of coverage**	98.8
**Est. number of reads**	4928
**Ambiguities**	0

??coverage-details?? ??variant-analysis??

Assignment

**Type**	Betapleolipovirus HRPV3 (Taxonomy ID: 2747323)
**Reference Genome**	NC_017088.1 (Length: 8770bp)
**NT Identity (%)**	66.0947
**NT Quality**	0.657928

Alignment

**Alignment score**	2635 (NT) + 6274 (AA) = 8909
**Concordance (%)**	52.6879
**Alignment method**	Global, seeded, nucleotide + amino acids (AGA)

Genome Region

Sequence starts at position null and ends at position null relative to the null reference sequence for null.

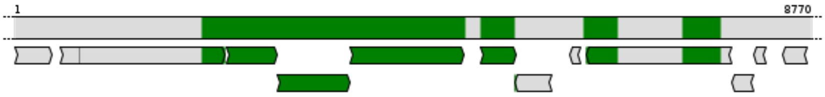

Alignment Detailed Statistics

	Begin	End	Coverage	Score	Concordance	Matches	Identities	I/D/M/F*	Stop Codons
**NT**	2102	7760	45.7%	2635.0	33.5%	3978 (95.5%)	2735 (65.7%)	160/27	
**CDS**									
2	529	614	14.0%	265.0	45.9%	85 (97.7%)	40 (46.0%)	1/1/0/0	1
ORF3	1	191	100%	791.0	67.0%	186 (96.9%)	118 (61.5%)	1/5/0/0	1
ORF4	1	262	100%	1206.0	66.9%	262 (95.6%)	169 (61.7%)	12/0/0/0	1
ORF5	1	388	100%	1701.0	62.5%	386 (90.6%)	252 (59.2%)	38/2/1/1	1
ORF6	1	132	100%	903.0	92.9%	132 (100%)	119 (90.2%)	0/0/0/0	1

	Begin	End	Coverage	Score	Concordance	Matches	Identities	I/D/M/F*	Stop Codons
ORF7	139	139	0.7%	-7.0	-140.0%	1 (100%)	0 (0%)	0/0/0/0	0
ORF9	44	546	48.0%	1415.0	78.8%	261 (99.6%)	196 (74.8%)	0/1/0/0	1
**Proteins**									
VP2 precursor (YP_005454274.1)	529	614	14.0%	265.0	45.9%	85 (97.7%)	40 (46.0%)	1/1/0/0	1
	T537E (2128A>G 2129C>A), N546D (2155A>G), E549Q (2164G>C 2166A>G), Q550N (2167C>A 2169G>C), K551Q (2170A>C), N557E (2188A>G 2190C>G), K558R (2191A>C 2192A>G), F559Y (2195T>A), E561D (2202G>C), S562A (2203T>G 2205C>G), Q563del (2206_2208delCAA), S564M (2210G>T 2211C>G), S565D (2212A>G 2213G>A), G566D (2216G>A 2217A>C), G567D (2219G>A), L569V (2224C>G 2226T>A), F570L (2227T>C), A573W (2236G>T 2237C>G 2238T>G), F574D (2239T>G 2240T>A), W576F (2246G>T 2247G>C), W576_G577insD (2247_2248insGAC), D578G (2252A>G 2253C>G), L579I (2254C>A), G580P (2257G>C 2258G>C), G581E (2261G>A), A582V (2264C>T 2265C>T), G583P (2266G>C 2267G>C 2268C>G), V585G (2273T>G), G586A (2276G>C 2277T>G), V587A (2279T>C), L588A (2281C>G 2282T>C 2283G>C), V590A (2288T>C), G592V (2294G>T 2295A>C), A595V (2303C>T 2304G>C), G597V (2309G>T 2310G>T), A598L (2311G>C 2312C>T 2313A>C), I599F (2314A>T), A600G (2318C>G), I601A (2320A>G 2321T>C), L602V (2323C>G 2325C>G), E604Q (2329G>C), A605I (2332G>A 2333C>T), I606A (2335A>G 2336T>C 2337C>G), K607S (2338A>T 2339A>C 2340A>C), F608L (2341T>C), L610I (2347C>A 2349G>C), R613K (2357G>A 2358G>A)								
hypothetical protein (YP_005454314.1)	460	544	15.6%	264.0	45.8%	84 (97.7%)	39 (45.3%)	1/1/0/0	0
	T468E (2128A>G 2129C>A), N477D (2155A>G), E480Q (2164G>C 2166A>G), Q481N (2167C>A 2169G>C), K482Q (2170A>C), N488E (2188A>G 2190C>G), K489R (2191A>C 2192A>G), F490Y (2195T>A), E492D (2202G>C), S493A (2203T>G 2205C>G), Q494del (2206_2208delCAA), S495M (2210G>T 2211C>G), S496D (2212A>G 2213G>A), G497D (2216G>A 2217A>C), G498D (2219G>A), L500V (2224C>G 2226T>A), F501L (2227T>C), A504W (2236G>T 2237C>G 2238T>G), F505D (2239T>G 2240T>A), W507F (2246G>T 2247G>C), W507_G508insD (2247_2248insGAC), D509G (2252A>G 2253C>G), L510I (2254C>A), G511P (2257G>C 2258G>C), G512E (2261G>A), A513V (2264C>T 2265C>T), G514P (2266G>C 2267G>C 2268C>G), V516G (2273T>G), G517A (2276G>C 2277T>G), V518A (2279T>C), L519A (2281C>G 2282T>C 2283G>C), V521A (2288T>C), G523V (2294G>T 2295A>C), A526V (2303C>T 2304G>C), G528V (2309G>T 2310G>T), A529L (2311G>C 2312C>T 2313A>C), I530F (2314A>T), A531G (2318C>G), I532A (2320A>G 2321T>C), L533V (2323C>G 2325C>G), E535Q (2329G>C), A536I (2332G>A 2333C>T), I537A (2335A>G 2336T>C 2337C>G), K538S (2338A>T 2339A>C 2340A>C), F539L (2341T>C), L541I (2347C>A 2349G>C), R544K (2357G>A 2358G>A)								
ORF3 (YP_005454275.1)	1	191	100%	791.0	67.0%	186 (96.9%)	118 (61.5%)	1/5/0/0	1
	M1V (2374A>G 2376G>T), N2V (2377A>G 2378A>T), T3V (2380A>G 2381C>T), F5G (2386T>G 2387T>G 2388T>A), R6V (2389A>G 2390G>T 2391A>C), I7V (2392A>G), I10A (2401A>G 2402T>C 2403C>G), G11A (2405G>C 2406G>C), L12V (2407C>G 2409G>C), L13V (2410T>G 2412A>G), I15F (2416A>T 2418C>T), A16G (2420C>G 2421T>G), M19_M21del (2428_2436delATGGGCATG), A25S (2446G>T), G28E (2456G>A 2457A>G), G30P (2461G>C 2462G>C 2463C>G), E31T (2464G>A 2465A>C 2466G>C), A32D (2468C>A), V33A (2471T>C 2472G>C), P34_I35del (2473_2478delCCGATT), T38_V39insD (2487_2488insGAC), E40I (2491G>A 2492A>T 2493A>C), G41E (2495G>A 2496C>G), G42S (2497G>T 2498G>C), T44S (2503A>T 2505G>C), E59S (2548G>T 2549A>C 2550G>C), T60S (2551A>T), T64V (2563A>G 2564C>T), G71D (2585G>A), T72Q (2587A>C 2588C>A), A73L (2590G>C 2591C>T 2592G>C), I75V (2596A>G), G76A (2600G>C), V79A (2609T>C 2610A>C), I85V (2626A>G 2628C>G), I87M (2634C>G), N90D (2641A>G), V91T (2644G>A 2645T>C 2646G>C), L94I (2653T>A 2655A>C), E97A (2663A>C 2664A>T), I102V (2677A>G), V105S (2686G>T 2687T>C 2688C>G), Q107E (2692C>G 2694G>A), P111D (2704C>G 2705C>A 2706G>C), I116L (2719A>C 2721A>C), A117V (2723C>T 2724G>C), V118F (2725G>T), S122E (2737T>G 2738C>A 2739C>G), A124G (2744C>G 2745G>C), N127H (2752A>C), V129A (2759T>C), S130T (2761T>A 2763G>C), A132G (2768C>G 2769A>C), G137S (2782G>T 2783G>C 2784A>C), I140L (2791A>C), A143D (2801C>A 2802G>C), E146A (2810A>C 2811A>C), N149D (2818A>G), R153Q (2830A>C 2831G>A), H154Y (2833C>T), L166G (2869C>G 2870T>G 2871G>A), L169I (2878T>A 2880A>C), V170L (2881G>C), S171T (2884T>A), A172S (2887G>T), V173L (2890G>C 2892C>T), W178Y (2906G>A 2907G>C), R181W (2914C>T 2916A>G), K185S (2927A>G 2928G>C), M188I (2937G>C), E189K (2938G>A 2940A>G)								
ORF4 (YP_005454276.1)	1	262	100%	1206.0	66.9%	262 (95.6%)	169 (61.7%)	12/0/0/0	1
	E3D (2951G>C), E4D (2954A>C), P5D (2955C>G 2956C>A 2957C>T), T6L (2958A>C 2959C>T 2960A>C), S8G (2964A>G 2966C>G), A14G (2983C>G 2984G>T), I17L (2991A>C), W19Y (2998G>A 2999G>C), L20V (3000C>G 3002G>C), M26L (3018A>C 3020G>C), L27V (3021C>G 3023G>C), A28V (3025C>T 3026G>T), M32L (3036A>C), T34S (3043C>G 3044T>C), L35V (3045C>G), G37A (3052G>C 3053T>C), I38A (3054A>G 3055T>C 3056C>G), L40V (3060T>G), Y42F (3067A>T), T48S (3084A>T 3086C>G), V54I (3102G>A), L57T (3111C>A 3112T>C 3113G>C), M61F (3123A>T 3125G>C), F71W (3154T>G 3155C>G), A73V (3160C>T), S80E (3180T>G 3181C>A), R81S (3183C>A), R82H (3187G>A 3188G>C), V84I (3192G>A 3194G>C), E85P (3195G>C 3196A>C 3197A>C), E88H (3204G>C 3206G>C), I89V (3207A>G), R92T (3217G>C), R93G (3219A>G 3221A>C), V96L (3228G>C 3230A>C), K97E (3231A>G 3233G>A), W99Y (3238G>A 3239G>C), E102D (3248G>C), I105M (3257T>G), S107A (3261A>G 3262G>C), S108E (3264T>G 3265C>A 3266T>G), S110N (3270T>A 3271C>A 3272G>C), D112E (3278T>G), G113D (3280G>A 3281A>C), P114A (3282C>G 3284G>C), N115T (3286A>C 3287T>C), G126A (3319G>C 3320C>G), S129E (3327T>G 3328C>A 3329C>G), Y132H (3336T>C), D133L (3339G>C 3340A>T 3341T>C), M136V (3348A>G 3350G>C), E137D (3353G>C), S138E (3354T>G 3355C>A 3356T>A), R140T (3360C>A 3361G>C 3362G>C), R142S (3366C>A), C149A (3387T>G 3388G>C 3389T>C), E157A (3412A>C 3413A>T), S163A (3429T>G), E166G (3439A>G), S174A (3462T>G 3464C>G), K176N (3470G>C), I177V (3471A>G 3473A>G), I182V (3486A>G 3488T>G), L185F (3495C>T), G191K (3513G>A 3514G>A 3515A>G), I193V (3519A>G 3521T>G), I194V (3522A>G), R196T (3528C>A 3529G>C 3530G>C), K203R (3549A>C 3550A>G 3551A>C), M207V (3561A>G 3563G>C), P209E (3567C>G 3568C>A), G211A (3574G>C 3575G>C), E220Q (3600G>C), S221E (3603T>G 3604C>A 3605C>G), E224G (3613A>G 3614A>C), I226T (3619T>C), A228P (3624G>C), L234I (3642C>A 3644T>C), E238D (3656A>C), S239L (3657T>C 3658C>T 3659G>C), M240A (3660A>G 3661T>C 3662G>C), M240_G241insDDVSGDG (3662_3663insGACGACGTGAGCGGAGACGGC), G241A (3664G>C 3665C>G), M242I (3668G>C), G243V (3670G>T), E244D (3674G>C), E247K (3681G>A), E247_P248insT (3683_3684insACG), P248T (3684C>A), D249G (3688A>G 3689C>A), S251G (3693A>G), T253E (3699A>G 3700C>A), V254I (3702G>A), V254_E255insANGT (3704_3705insGCCAACGGAACC), E255A (3706A>C), P258V (3714C>G 3715C>T)								
ORF5 (YP_005454277.1)	1	388	100%	1701.0	62.5%	386 (90.6%)	252 (59.2%)	38/2/1/1	1

	Begin	End	Coverage	Score	Concordance	Matches	Identities	I/D/M/F*	Stop Codons
	T2I (3725C>T), D3E (3729C>A), D4A (3731A>C), N5E (3733A>G 3735C>A), D6L (3736G>C 3737A>T 3738C>A), V7L (3739G>C 3741T>C), Y8R (3742T>C 3743A>G), T9A (3745A>G 3747A>C), P10V (3748C>G 3749C>T), A11G (3752C>G 3753A>G), Q12F (3754C>T 3755A>T 3756G>T), Y13W (3758A>G 3759T>G), Y13_R14insAACX (3759_3760insGCCGCGTGTGT), E15G (3764A>G 3765G>T), H16_Q17insRPVSPTAPGGCRPAP (3768_3769insCGTCCTGTATCACCGACCGCTCCGGGCGGCTGCCGCCCGGCTCCG), Q17R (3770A>G), D18G (3773A>G 3774C>T), G19S (3775G>A), G21M (3781G>A 3782G>T), V22T (3784G>A 3785T>C), R23D (3787C>G 3788G>A), P25N (3793C>A 3794C>A 3795G>C), A27V (3800C>T), H28Y (3802C>T), P29T (3805C>A 3807C>G), H30P (3809A>C 3810C>G), G32Q (3814G>C 3815G>A), I33F (3817A>T 3819A>C), I33_V34insREHQDGYG (3819_3820insCGCGAGCATCAGGACGGCTACGGG), E37A (3830A>C 3831G>C), E38D (3834G>C), V39A (3836T>C 3837A>C), S40D (3838T>G 3839C>A), Y42H (3844T>C), Y42_L43insTGVVRDPT (3846_3847insACGGGCGTCGTGCGCGACCCGACG), L43V (3847C>G 3849G>C), V45R (3853G>C 3854T>G 3855T>C), L46Y (3856C>T 3857T>A 3858T>C), S47L (3859T>C 3860C>T 3861G>C), E49V (3866A>T 3867G>C), Y50V (3868T>G 3869A>T 3870T>C), G51A (3872G>C), H52N (3874C>A 3876T>C), G53E (3878G>A), Q54Y (3880C>T 3882G>C), G55D (3884G>A 3885T>C), H56P (3887A>C 3888C>G), F57del (3889_3891delTTC), D59E (3897C>G), D61S (3901G>T 3902A>C), R62Q (3905G>A), R62_L63insPGH (3906_3907insCCGGGCCAT), L63M (3907C>A 3909A>G), R66T (3916C>A 3917G>C 3918A>C), A67Y (3919G>T 3920C>A 3921G>C), K71G (3931A>G 3932A>G 3933A>C), E74Q (3940G>C 3942G>A), R75Q (3943A>C 3944G>A 3945A>G), E78S (3952G>T 3953A>C), A85G (3974C>G), S87E (3979T>G 3980C>A 3981G>A), D90A (3989A>C 3990T>G), A91N (3991G>A 3992C>A 3993G>C), S96T (4006T>A 4008T>G), E106Q (4036G>C), K118Q (4072A>C), I119L (4075A>C 4077A>C), E121Q (4081G>C), T138A (4132A>G), L143F (4149A>C), S144A (4150T>G 4152G>C), F147L (4159T>C), K152S (4175A>G 4176G>C), E158D (4194G>C), Q159K (4195C>A), S166T (4217G>C 4218T>G), K168E (4222A>G), T171D (4231A>G 4232C>A 4233G>C), K172P (4234A>C 4235A>C), V174I (4240G>A 4242A>C), S176G (4246T>G 4247C>G 4248G>C), E177D (4251G>C), V180A (4259T>C 4260A>T), S182T (4264T>A), G186P (4276G>C 4277G>C 4278C>G), G189A (4286G>C 4287A>G), M202L (4324A>C 4326G>C), P205E (4333C>G 4334C>A), G207S (4339G>T 4340G>C 4341T>C), P208R (4343C>G 4344G>C), I212L (4354A>C), D221S (4381G>T 4382A>C), G223T (4387G>A 4388G>C 4389A>C), A226S (4396G>A 4397C>G 4398G>C), Y228H (4402T>C), K232Q (4414A>C), F244Y (4451T>A), N245D (4453A>G), T247L (4459A>C 4460C>T), V251I (4471G>A), S273A (4537T>G 4539T>C), Q276R (4547A>G), I278V (4552A>G), S282K (4564T>A 4565C>A), N285S (4573A>T 4574A>C 4575C>G), I288L (4582A>C), E291I (4591G>A 4592A>T 4593G>C), R292Q (4594A>C 4595G>A), R294E (4600C>G 4601G>A 4602T>G), A311S (4651G>T 4653G>C), A316T (4666G>A 4668C>G), S318E (4672T>G 4673C>A), E319D (4677G>C), E320D (4680G>C), E321D (4683G>C), T323E (4687A>G 4688C>A 4689G>A), S328E (4702T>G 4703C>A 4704C>A), Y331F (4712A>T 4713T>C), V335L (4723G>T 4725T>G), V339R (4735G>A 4736T>G 4737T>G), R340A (4738C>G 4739G>C), K343E (4747A>G), N348H (4762A>C), K353Q (4777A>C), V355I (4783G>A 4785G>C), Y357F (4790A>T 4791T>C), S358G (4792A>G 4794C>G), H359K (4795C>A 4797C>A), G360S (4798G>T 4799G>C), T364D (4810A>G 4811C>A 4812T>C), R367S (4819A>T 4820G>C), Y369I (4825T>A 4826A>T), E370del (4828_4830delGAG), Q371R (4832A>G 4833G>C), K374D (4840A>G 4842A>C), R376A (4846C>G 4847G>C 4848G>C), D377G (4850A>G 4851T>A), T378A (4852A>G), I379M (4857A>G), S380D (4858T>G 4859C>A), N381R (4861A>C 4862A>G 4863C>G)								
ORF6 (YP_005454278.1)	1	132	100%	903.0	92.9%	132 (100%)	119 (90.2%)	0/0/0/0	1
	G28S (5145G>T 5146G>C 5147C>G), T31K (5155C>A 5156G>A), L41I (5184C>A 5186C>T), K42R (5187A>C 5188A>G 5189A>G), R55T (5226C>A 5227G>C), G72S (5277G>T 5278G>C), K73R (5280A>C 5281A>G 5282G>C), S76A (5289T>G 5291G>C), G78S (5295G>A 5297T>C), V88R (5325G>C 5326T>G 5327C>G), K99A (5358A>G 5359A>C 5360G>C), A102P (5367G>C 5369G>T), I125V (5436A>G)								
ORF7 (YP_005454279.1)	139	139	0.7%	-7.0	-140.0%	1 (100%)	0 (0%)	0/0/0/0	0
	*139Q (5458A>G)								
ORF9 (YP_005454281.1)	44	546	48.0%	1415.0	78.8%	261 (99.6%)	196 (74.8%)	0/1/0/0	1
	G46A (7752A>G 7753C>G), E48T (7746C>G 7747T>G 7748C>T), R49D (7743T>G 7744C>T 7745G>C), T51S (7738G>C), A52V (7735G>A), G60N (7710C>G 7711C>T 7712C>T), F61del (7707_7709delAAA), L62I (7706G>T), P64H (7699G>T), E65P (7696T>G 7697C>G), S66G (7693G>C 7694A>C), G67E (7689G>T 7690C>T), V68T (7687A>G 7688C>T), S71A (7679A>C), E74P (7668C>G 7669T>G 7670C>G), I75F (7665T>G 7667T>A), E76R (7663T>C 7664C>T), E78D (7656C>A), Q79E (7655G>C), I85L (7635T>C 7637T>G), A86S (7632C>G 7634C>A), I87V (7629T>G 7631T>C), D88E (7626G>C), R89A (7623C>G 7624C>G 7625G>C), H90V (7621T>A 7622G>C), P91E (7617T>C 7618G>T 7619G>C), E93A (7612T>G), H95M (7605G>C 7606T>A 7607G>T), V96S (7603A>C 7604C>T), G97D (7599T>G 7600C>T), E98Q (7598C>G), Q99R (7594T>C), L101F (7587T>A 7589G>A), I105V (7575G>A 7577T>C), A106R (7573G>C 7574C>G), G111H (7558C>T 7559C>G), E113R (7551T>C 7552T>C 7553C>G), K117N (7539C>G), R121K (7528C>T 7529G>T), V126T (7512G>A 7513A>G 7514C>T), V143I (7461G>A 7463C>T), P150T (7440C>T 7442G>T), A154S (7430C>A), G155A (7425C>G 7426C>G), V159I (7415C>T), V161I (7407C>G 7409C>T), A167S (7389C>G 7391C>A), A180G (7350G>T 7351G>C), S427R (6610G>C 6611A>G), M438L (6576C>T 6578T>A), S442Q (6564G>C 6565G>T 6566A>G), I461L (6507G>T 6509T>G), N462S (6504G>A 6505T>G 6506T>A), S471H (6478G>T 6479A>G), D487N (6431C>T), T489A (6425T>C), S490G (6421G>C 6422A>C), G499E (6393G>T 6394C>T), Q510E (6360C>T 6362G>C), E513R (6352T>C 6353C>G), N517D (6341T>C), A523G (6321G>T 6322G>C), S527Q (6309G>C 6310G>T 6311A>G), R529K (6303T>C 6304C>T), T537H (6279C>G 6280G>T 6281T>G)								

*: Inserts / Deletes / Misaligned / Frameshifts

Detailed Results of Alphapleolipovirus HRPV2 (NC_017087.1)

NGS details

Assembly

**Coverage length**	987 (3 contig(s))
**Est. depth of coverage**	10.9
**Est. number of reads**	133
**Ambiguities**	0

??coverage-details?? ??variant-analysis??

Assignment

**Type**	Alphapleolipovirus HRPV2 (Taxonomy ID: 2747320)
**Reference Genome**	NC_017087.1 (Length: 10656bp)
**NT Identity (%)**	67.6877
**NT Quality**	0.696049

Alignment

**Alignment score**	687 (NT) + 1282 (AA) = 1969
**Concordance (%)**	51.3295
**Alignment method**	Global, seeded, nucleotide + amino acids (AGA)

Genome Region

Sequence starts at position null and ends at position null relative to the null reference sequence for null.

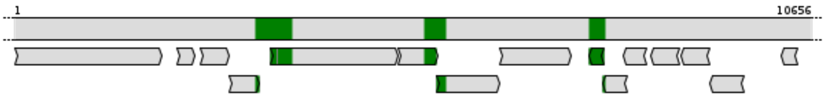

Alignment Detailed Statistics

	Begin	End	Coverage	Score	Concordance	Matches	Identities	I/D/M/F*	Stop Codons
**NT**	3227	7905	9.3%	687.0	35.1%	984 (96.9%)	685 (67.5%)	28/3	
**CDS**									
4	115	134	14.9%	104.0	78.2%	20 (100%)	14 (70.0%)	0/0/0/0	1
5	1	100	17.7%	385.0	61.6%	99 (97.1%)	58 (56.9%)	2/1/0/0	0
ORF6	123	177	31.1%	260.0	72.8%	55 (100%)	37 (67.3%)	0/0/0/0	1
ORF7	1	39	14.0%	82.0	31.2%	39 (92.9%)	13 (31.0%)	3/0/0/0	0
ORF9	1	65	98.5%	410.0	91.1%	65 (100%)	56 (86.2%)	0/0/0/0	0
ORF10	99	109	10.1%	41.0	45.1%	11 (100%)	7 (63.6%)	0/0/0/0	1
**Proteins**									

	Begin	End	Coverage	Score	Concordance	Matches	Identities	I/D/M/F*	Stop Codons
VP4 (YP_005454261.1)	115	134	14.9%	104.0	78.2%	20 (100%)	14 (70.0%)	0/0/0/0	1
	I118V (3237A>G 3239C>G), A119I (3240G>A 3241C>T), V123I (3252G>A 3254G>C), A126V (3262C>T 3263C>T), A127S (3264G>T), G129A (3271G>C 3272C>T)								
VP5 precursor (YP_005454262.1)	1	100	17.7%	385.0	61.6%	99 (97.1%)	58 (56.9%)	2/1/0/0	0
	G2S (3419G>A), T5R (3428A>C 3429C>G 3430C>G), V7M (3434G>A 3436A>G), K8Q (3437A>C), A13T (3452G>A 3454T>C), G14A (3456G>C), A15V (3459C>T), T17G (3464A>G 3465C>G 3466G>T), V18A (3468T>C), S21A (3476T>G), V22S (3479G>T 3480T>C), D23T (3482G>A 3483A>C 3484C>G), D24N (3485G>A), F26P (3491T>C 3492T>C 3493C>G), A27V (3495C>T 3496A>C), Q28G (3497C>G 3498A>G 3499A>G), D29E (3502C>G), Q31E (3506C>G), A34del (3515_3517delGCC), L36P (3522T>C), V37I (3524G>A), G38A (3528G>C 3529C>G), V39I (3530G>A), L41G (3536C>G 3537T>G 3538C>G), A43_G44insLG (3544_3545insCTTGGC), A45S (3548G>T), V46A (3552T>C 3553C>T), G47A (3555G>C 3556C>T), V48F (3557G>T), I57L (3584A>C), V58I (3587G>A), A62D (3600C>A), A69P (3620G>C), D70E (3625C>A), K73H (3632A>C 3634A>C), Q75R (3639A>G 3640G>A), Q78E (3647C>G), K81R (3656A>C 3657A>G 3658G>A), T82A (3659A>G 3661T>G), I91F (3686A>T), Q95K (3698C>A 3700G>A), L98V (3707C>G)								
hypothetical protein (YP_005454313.1)	1	68	12.8%	302.0	71.2%	67 (95.7%)	44 (62.9%)	2/1/0/0	0
	A2del (3515_3517delGCC), L4P (3522T>C), V5I (3524G>A), G6A (3528G>C 3529C>G), V7I (3530G>A), L9G (3536C>G 3537T>G 3538C>G), A11_G12insLG (3544_3545insCTTGGC), A13S (3548G>T), V14A (3552T>C 3553C>T), G15A (3555G>C 3556C>T), V16F (3557G>T), I25L (3584A>C), V26I (3587G>A), A30D (3600C>A), A37P (3620G>C), D38E (3625C>A), K41H (3632A>C 3634A>C), Q43R (3639A>G 3640G>A), Q46E (3647C>G), K49R (3656A>C 3657A>G 3658G>A), T50A (3659A>G 3661T>G), I59F (3686A>T), Q63K (3698C>A 3700G>A), L66V (3707C>G)								
ORF6 (YP_005454263.1)	123	177	31.1%	260.0	72.8%	55 (100%)	37 (67.3%)	0/0/0/0	1
	N130S (5509A>T 5510A>C), T131S (5512A>T 5514G>C), L132F (5515C>T 5517G>T), I133V (5518A>G), G134A (5522G>C 5523C>T), S140Q (5539T>C 5540C>A 5541C>G), A145S (5554G>T), I147L (5560A>C), G150A (5570G>C 5571C>G), L151I (5572C>A), A155L (5584G>C 5585C>T), V156T (5587G>A 5588T>C), I158L (5593A>C 5595C>G), F159Y (5597T>A 5598C>T), T164R (5611A>C 5612C>G), R168Q (5624G>A), E171D (5634A>C), M175I (5646G>A)								
ORF7 (YP_005454264.1)	1	39	14.0%	82.0	31.2%	39 (92.9%)	13 (31.0%)	3/0/0/0	0
	R2S (5654A>C), E3D (5657G>C), K4T (5659A>C), P5Q (5662C>A), D6H (5664G>C), T7_N8insPP (5669_5670insCCGCCA), N8Q (5670A>C 5672C>G), P10R (5677C>G), E11_K12insE (5681_5682insGAA), K12Q (5682A>C), R14A (5688C>G 5689G>C), P15Y (5691C>T 5692C>A 5693G>C), K17D (5697A>G 5699G>C), V19L (5703G>C), L22S (5712C>T 5713T>C 5714C>G), K23R (5715A>C 5716A>G 5717G>C), T24A (5718A>G 5720G>T), K25R (5721A>C 5722A>G 5723G>C), V26A (5725T>C 5726C>T), H28Q (5732T>G), V31A (5740T>C), G32V (5743G>T 5744G>C), T33A (5745A>G), I34L (5748A>C), A35G (5752C>G), L36V (5754C>G), A37S (5757G>T)								
ORF9 (YP_005454266.1)	1	65	98.5%	410.0	91.1%	65 (100%)	56 (86.2%)	0/0/0/0	0
	E6N (7862T>G 7864C>T), E26K (7804C>T), S27E (7800G>T 7801A>C), V41A (7758A>G), E46K (7744C>T), K47R (7740T>C 7741T>G), A48S (7736T>C 7738C>A), S50D (7731C>T 7732T>C), R55K (7715G>C 7716C>T 7717G>T)								
ORF10 (YP_005454267.1)	99	109	10.1%	41.0	45.1%	11 (100%)	7 (63.6%)	0/0/0/0	1
	D101G (7897T>C), M102V (7893C>G 7895T>C), M105T (7884C>T 7885A>G), W106F (7881C>G 7882C>A)								

*: Inserts / Deletes / Misaligned / Frameshifts

Detailed Results of Myohalovirus phiH (NC_052650.1)

NGS details

Assembly

**Coverage length**	4431 (12 contig(s))
**Est. depth of coverage**	29.4
**Est. number of reads**	1580
**Ambiguities**	0

??coverage-details?? ??variant-analysis??

Assignment

**Type**	Myohalovirus phiH (Taxonomy ID: 1980944)
**Reference Genome**	NC_052650.1 (Length: 58072bp)
**NT Identity (%)**	70.8586
**NT Quality**	0.820357

Alignment

**Alignment score**	3635 (NT) + 6650 (AA) = 10285
**Concordance (%)**	55.6036
**Alignment method**	Local, heuristic, nucleotide (BLASTN)

Genome Region

Sequence starts at position null and ends at position null relative to the null reference sequence for null.

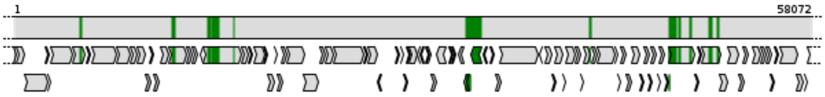

Alignment Detailed Statistics

	Begin	End	Coverage	Score	Concordance	Matches	Identities	I/D/M/F*	Stop Codons
**NT**	4754	51421	7.6%	3635.0	41.5%	4413 (98.5%)	3161 (70.6%)	48/18	
**CDS**									
JR051_gp07	171	241	22.6%	308.0	61.6%	71 (100%)	44 (62.0%)	0/0/0/0	0
hp67	83	186	24.0%	386.0	54.4%	104 (98.1%)	58 (54.7%)	2/0/0/0	0
tpm	107	775	36.1%	1436.0	72.1%	336 (99.7%)	234 (69.4%)	1/0/0/0	0
JR051_gp53	1	131	97.8%	530.0	61.0%	131 (94.2%)	79 (56.8%)	8/0/0/0	0
JR051_gp54	1	259	100%	1323.0	76.5%	259 (100%)	182 (70.3%)	0/0/0/0	1
JR051_gp67	117	165	29.7%	205.0	60.1%	49 (100%)	34 (69.4%)	0/0/0/0	1

	Begin	End	Coverage	Score	Concordance	Matches	Identities	I/D/M/F*	Stop Codons
JR051_gp81	94	100	7.0%	19.0	38.0%	7 (100%)	4 (57.1%)	0/0/0/0	0
dcm5	1	573	54.1%	1688.0	67.1%	338 (98.3%)	229 (66.6%)	4/2/0/0	0
yhdJ	180	444	34.6%	755.0	67.2%	153 (96.8%)	97 (61.4%)	1/4/0/0	0
**Proteins**									
head morphogenesis protein (YP_009981813.1)	171	241	22.6%	308.0	61.6%	71 (100%)	44 (62.0%)	0/0/0/0	0
	Y175F (4769A>T), D176E (4773C>G), S177G (4774T>G 4775C>G), S181D (4786A>G 4787G>A), H182Y (4789C>T), E185R (4798G>C 4799A>G 4800G>C), N193I (4823A>T), L198A (4837C>G 4838T>C 4839G>A), V200S (4843G>A 4844T>G 4845C>T), S203A (4852A>G 4853G>C 4854C>G), Q205E (4858C>G), E206D (4863G>T), S207A (4864T>G 4866G>C), M209S (4870A>T 4871T>C), I216L (4891A>C), A217T (4894G>A 4896G>A), I218E (4897A>G 4898T>A 4899C>G), G219I (4900G>A 4901G>T 4902G>C), D221G (4907A>G 4908C>T), T223E (4912A>G 4913C>A 4914T>G), D226S (4921G>A 4922A>G 4923C>T), N228A (4927A>G 4928A>C 4929C>A), Q229S (4930C>T 4931A>C), N231D (4936A>G 4938C>T), A236P (4951G>C 4953C>G), E238G (4958A>G), P240T (4963C>A)								
tail sheath protein (YP_009981823.1)	83	186	24.0%	386.0	54.4%	104 (98.1%)	58 (54.7%)	2/0/0/0	0
	Q85E (11511C>G 11513G>A), V88H (11520G>C 11521T>A), V89E (11524T>A 11525C>A), N92E (11532A>G 11534C>G), D94T (11538G>A 11539A>C), D95S (11541G>T 11542A>C 11543T>G), G97E (11548G>A 11549C>G), V99L (11553G>C), A100E (11557C>A), I104A (11568A>G 11569T>C 11570C>G), H105L (11572A>T), A107E (11578C>A 11579C>G), T111E (11589A>G 11590C>A 11591C>G), A112I (11592G>A 11593C>T 11594C>T), A114G (11599C>G 11600A>C), R115Y (11601C>T 11602G>A 11603G>C), T120S (11616A>T), A121T (11619G>A 11621T>C), V125I (11631G>A), T127E (11637A>G 11638C>A), P128G (11640C>G 11641C>G), P128_A129insDV (11642_11643insGACGTC), A129E (11644C>A), E130D (11648G>C), V133A (11656T>C), A134H (11658G>C 11659C>A 11660G>C), H136N (11664C>A), G138D (11671G>A 11672G>C), I142V (11682A>G), S143V (11685T>G 11686C>T), G145D (11692G>A), G147A (11698G>C 11699G>C), T148E (11700A>G 11701C>A 11702T>G), A149P (11703G>C), L152V (11712C>G 11714G>C), H156T (11724C>A 11725A>C 11726C>G), S160G (11736A>G), E163D (11747A>C), E166K (11754G>A), Y167S (11757T>A 11758A>G), N168Q (11760A>C 11762C>G), G169S (11763G>T 11764G>C 11765C>G), I170V (11766A>G), N177D (11787A>G), T178V (11790A>G 11791C>T 11792G>C), R179Q (11794G>A 11795A>G), D184H (11808G>C)								
tape-measure tail protein (YP_009981827.1)	107	775	36.1%	1436.0	72.1%	336 (99.7%)	234 (69.4%)	1/0/0/0	0
	L112M (14125C>A 14127C>G), A113S (14128G>T), S115G (14134T>G 14135C>G 14136G>C), T116A (14137A>G), Q120K (14149C>A 14151A>G), S128D (14173T>G 14174C>A 14175A>C), V133M (14188G>A 14190C>G), T134D (14191A>G 14192C>A 14193T>C), E138A (14204A>C), A146S (14227G>T), N147G (14230A>G 14231A>G 14232T>G), R149N (14236C>A 14237G>A), G150A (14240G>C), E152Q (14245G>C 14247A>G), A153S (14248G>T 14250C>G), E155K (14254G>A), V156T (14257G>A 14258T>C 14259T>C), M161L (14272A>C 14274G>C), L166M (14287C>A 14289C>G), D172N (14305G>A), S177A (14320T>G), L178M (14323C>A), S201A (14392T>G), A204S (14401G>A 14402C>G 14403C>T), L208I (14413C>A 14415T>C), V210A (14420T>C 14421C>T), G212E (14426G>A), G215A (14435G>C), L217I (14440C>A 14442G>C), N223S (14458A>T 14459A>C), I225L (14464A>C), Q226E (14467C>G 14469G>A), S244N (14522G>A), G246S (14527G>A 14529C>T), Q247E (14530C>G 14532G>A), K249Q (14536A>C), D253E (14550T>G), M259T (14567T>C 14568G>T), D260Q (14569G>C 14571C>G), D265S (14584G>T 14585A>C 14586C>G), Q268D (14593C>G 14595G>T), D271S (14602G>A 14603A>G 14604C>T), L272M (14605C>A 14607C>G), S273T (14608T>A 14610G>C), T274D (14611A>G 14612C>A 14613G>C), V275I (14614G>A 14616G>C), M276L (14617A>C), T277G (14620A>G 14621C>G 14622G>C), Q282E (14635C>G), E284D (14643G>C), L286V (14647C>G 14649G>C), G287_E288insS (14652_14653insTCG), E288Q (14653G>C 14655A>G), Q291R (14663A>G 14664G>A), R293A (14668C>G 14669G>C 14670G>T), A299V (14687C>T), N312E (14725A>G 14727C>G), R313D (14728C>G 14729G>A 14730G>C), E316K (14737G>A), I321L (14752A>C 14754C>G), Q322E (14755C>G 14757G>A), S323D (14758T>G 14759C>A 14760G>C), I336L (14797A>C), M339L (14806A>T), N341D (14812A>G 14814C>T), I344L (14821A>C), E345S (14824G>T 14825A>C), A346G (14828C>G 14829G>A), T347V (14830A>G 14831C>T), E354D (14853A>C), G357E (14861G>A), E358Q (14863G>C), D360G (14870A>G 14871T>G), Q362K (14875C>A), Q363E (14878C>G), V365I (14884G>A), S366T (14887T>A 14889C>G), Q369R (14897A>G 14898G>C), E370K (14899G>A 14901G>A), D372S (14905G>T 14906A>C 14907T>A), Q373N (14908C>A 14910G>C), Q374E (14911C>G), E379A (14927A>C 14928A>C), M382A (14935A>G 14936T>C), A716G (15938C>G), S717R (15940A>C 15942T>G), L725M (15964C>A), L731I (15982T>A 15984G>C), K735R (15995A>G 15996G>A), S742E (16015A>G 16016G>A 16017C>A), K743R (16018A>C 16019A>G), I745L (16024A>C), D746E (16029C>G), K749R (16036A>C 16037A>G), Q750S (16039C>A 16040A>G 16041G>C), G753S (16048G>T 16049G>C), N757S (16061A>G), K760R (16069A>C 16070A>G), S761Q (16072A>C 16073G>A 16074C>G), A762R (16075G>C 16076C>G), A765E (16085C>A 16086C>G), V773I (16108G>A)								
hypothetical protein (YP_009981858.1)	1	131	97.8%	530.0	61.0%	131 (94.2%)	79 (56.8%)	8/0/0/0	0
	E3D (33247T>A), E4D (33244T>G), D5_E6insQKE (33240_33241insCTCCTTCTG), E6D (33238T>G), V7L (33237C>G), R8S (33232A>G 33233C>G 33234G>A), D9E (33229A>C), R13G (33219G>C), D17_D18insPAGDQ (33204_33205insCTGGTCACCTGCTGG), D18E (33202G>T), S20D (33196G>A 33197G>T 33198A>C), T21V (33193C>G 33194G>A 33195T>C), K22D (33190T>G 33192T>C), E24T (33185T>G 33186C>T), N25D (33181A>G 33183T>C), Q27T (33176T>G 33177G>T), H28Q (33172A>C), Q30E (33168G>C), T31Q (33163A>C 33164G>T 33165T>G), K33Q (33159T>G), P34R (33155G>C), K36T (33148C>A 33149T>G), N37S (33146T>G 33147T>A), A38E (33143G>T), H40A (33137T>G 33138G>C), E44A (33124C>T 33125T>G), M45K (33122A>T), K46N (33118T>A), E47K (33117C>T), Q48R (33113T>C 33114G>T), E50A (33106T>G 33107T>G), L52R (33101A>C), A53V (33097G>T 33098G>A), E54K (33096C>T), K58E (33084T>C), E59S (33080T>G 33081C>A), Q72D (33040C>G 33042G>C), R74Q (33034T>C 33035C>T), R78T (33023C>G 33024G>T), L82A (33011A>G 33012G>C), A85E (33002G>T), Y90F (32987T>A), Q92R (32981T>C), V104L (32944C>G 32946C>G), I105V (32941T>G 32943T>C), M107L (32937T>G), I109M (32929A>C), L112I (32920C>A 32922A>T), P117A (32907G>C), T118H (32902T>A 32903G>T 32904T>G), V120L (32896G>A 32898C>G), E125K (32883C>T), R126D (32879C>T 32880G>C)								
ParA family protein (YP_009981859.1)	1	259	100%	1323.0	76.5%	259 (100%)	182 (70.3%)	0/0/0/0	1
	T3S (34016C>T 34018T>A), I6V (34009T>C), S16T (33977T>C 33979A>T), S25A (33950G>C 33952A>C), S27A (33944C>G 33946A>C), S28D (33941A>G 33942C>T 33943T>C), R32D (33929T>G 33930C>T 33931G>C), A34L (33923A>G 33924G>A 33925C>G), V35A (33920A>G 33921A>G), A36V (33918G>A), L38A (33911C>G 33912A>G 33913G>C), D49G (33879T>C), L50F (33877G>A), E53A (33867T>G), L55A (33861A>G 33862G>C), K58P (33851T>C 33852T>G 33853T>G), T60S (33847T>A), F62Y (33840A>T), T63S (33838T>A), A64L (33834G>A 33835C>G), M65F (33830C>G 33832T>A), D67S (33824A>G 33825T>C 33826C>T), A69H (33819G>T 33820C>G), G70E (33815T>C 33816C>T), T71H (33813G>T 33814T>G), G72D (33810C>T), L73A (33807A>G 33808G>C), E74D (33803T>G), I76V (33799T>C), V77I (33796C>T), V78L (33793C>G), E81A (33782T>C 33783T>G), I86L (33767A>G 33769T>G), I90V (33755G>C 33757T>C), R94K (33743A>T 33744C>T 33745G>T), E98N (33731T>A 33733C>T), L108Q (33701G>C 33702A>T), F110L (33697A>G), R111E (33692G>C 33693C>T 33694G>C), Q112D (33689C>G 33691G>C), L113V (33686C>G 33688G>C), L114F (33685G>A), E115D (33680T>G), T118G (33672G>C 33673T>C), L123V (33658G>C), I124F (33655T>A), V147I (33586C>T), T154S (33565T>A), I156M (33557G>C), I159L (33550T>G), V171R (33512T>G 33513A>C 33514C>G), I175V (33502T>C), S176Q (33497G>C 33498C>T 33499T>G), I177L (33494G>C 33496T>G), Q178R (33491C>T 33492T>C), S197E (33435G>T 33436A>C), S203G (33418T>C), I212V (33389G>C 33391T>C), H217K (33374A>C 33376G>T), S224T (33355A>T), S226N (33347A>G 33348C>T), S227N (33344A>G 33345G>T 33346A>T), I231L (33334T>G), G233S (33326A>G 33328C>T), A234D (33324G>T), E238D (33311T>G), T239C (33308G>A 33309G>C 33310T>A), N243G (33297T>C 33298T>C), N246E (33287G>C 33289T>C), E247D (33284C>G), L248I (33281A>G 33283G>T), D250A (33275G>C 33276T>G), Y251T (33272G>C 33273T>G 33274A>T), E253V (33267T>A), T254E (33264G>T 33265T>C), I255V (33262T>C), D256A (33258T>G)								
hypothetical protein (YP_009981872.1)	117	165	29.7%	205.0	60.1%	49 (100%)	34 (69.4%)	0/0/0/0	1

	Begin	End	Coverage	Score	Concordance	Matches	Identities	I/D/M/F*	Stop Codons
	L121F (41909G>C), S132R (41942T>G), E134K (41946G>A), G148E (41989G>A), K153Q (42003A>C 42005G>A), C155Q (42009T>C 42010G>A 42011T>G), A156* (42012G>T 42013C>G), T157D (42015A>G 42016C>A 42017A>T), N158T (42019A>C), H159D (42021C>G), S160T (42024T>A 42026G>C), S162P (42030T>C), P163A (42033C>G 42035C>T), C164G (42036T>G), *165R (42039T>A 42041A>G)								
NTPase protein (YP_009981886.1)	94	100	7.0%	19.0	38.0%	7 (100%)	4 (57.1%)	0/0/0/0	0
	H97R (47729A>G), D98N (47731G>A), *100R (47737T>C)								
C-5 cytosine-specific DNA methylase (YP_009981887.1)	1	573	54.1%	1688.0	67.1%	338 (98.3%)	229 (66.6%)	4/2/0/0	0
	G4del (47741_47743delGGG), P5Q (47745C>A), I7T (47751T>C 47752C>G), V8I (47753G>A 47755T>C), V22A (47796T>C), A23V (47799C>T 47800G>C), K24Q (47801A>C), E27R (47810G>C 47811A>G 47812G>A), D28N (47813G>A), L29M (47816C>A 47818C>G), N30G (47819A>G 47820A>G 47821C>G), E32S (47825G>T 47826A>C 47827A>C), G34E (47832G>A 47833T>G), E35D (47836G>C), E35_D36insG (47836_47837insGGC), L37V (47840C>G), V42I (47855G>A), W45D (47864T>G 47865G>A 47866G>C), K46E (47867A>G), P47D (47870C>G 47871C>A 47872G>T), R50A (47879C>G 47880G>C 47881G>A), E53R (47888G>C 47889A>G), E54L (47891G>C 47892A>T), G57P (47900G>C 47901G>C 47902C>G), N60D (47909A>G), R65K (47924C>A 47925G>A 47926C>G), Y70H (47939T>C 47941T>C), D76E (47959T>A), S79T (47967G>C 47968C>G), T81D (47972A>G 47973C>A), T84C (47981A>T 47982C>G 47983G>T), E88S (47993G>T 47994A>C 47995G>A), F92H (48005T>C 48006T>A), N94T (48012A>C 48013C>G), S102R (48035A>C 48037T>C), D103E (48040C>G), H111T (48062C>A 48063A>C 48064C>G), D114K (48071G>A 48073C>G), I116V (48077A>G), K118M (48084A>T), R120Q (48090G>A 48091C>G), H123N (48098C>A), V124L (48101G>C 48103C>G), L126I (48107C>A 48109A>C), G136A (48138G>C 48139G>T), D139del (48146_48148delGAC), N143E (48158A>G 48160C>G), P144C (48161C>T 48162C>G 48163G>C), T145A (48164A>G 48166G>C), K146R (48167A>C 48168A>G 48169G>A), E152Q (48185G>C), R153Q (48189G>A), W154F (48192G>T 48193G>C), S156T (48198G>C 48199C>G), L158_E159insQ (48205_48206insCAA), G162N (48215G>A 48216G>A 48217G>C), S164D (48221T>G 48222C>A 48223G>C), V166N (48227G>A 48228T>A 48229G>C), E169M (48236G>A 48237A>T), K170D (48239A>G 48241G>C), D173E (48250C>G), D185E (48286T>G), P186A (48287C>G), E190D (48301A>T), I194L (48311A>C 48313C>G), M195L (48314A>C), P222T (48395C>A), H234T (48431C>A 48432A>C 48433C>A), L236I (48437C>A 48439G>C), N243H (48458A>C), P244A (48461C>G 48463C>T), T247Q (48470A>C 48471C>A), A250S (48479G>T), K251S (48482A>T 48483A>C), G252K (48485G>A 48486G>A), A255G (48495C>G 48496C>G), S266D (48527A>G 48528G>A), D267E (48532C>G), R268Q (48534G>A 48535G>A), D274S (48551G>A 48552A>G 48553C>T), Q278A (48563C>G 48564A>C), I279L (48566A>T 48568C>G), G280D (48570G>A 48571G>C), P281A (48572C>G), E283A (48579A>C), I507L (49250A>C), D508I (49253G>A 49254A>T), D509E (49258C>G), F510Y (49260T>A), Q511Y (49262C>T 49264G>C), G512_P513insNS (49267_49268insAACTCA), P513T (49268C>A), P514S (49271C>T), K515T (49275A>C), P516A (49277C>G 49279G>C), I517V (49280A>G), S519E (49286T>G 49287C>A), N522P (49295A>C 49296A>C 49297T>G), Q524V (49301C>G 49302A>T), H525T (49304C>A 49305A>C 49306C>G), A526T (49307G>A 49309G>C), N527R (49311A>G 49312C>G), C533V (49328T>G 49329G>T 49330C>G), I534L (49331A>C 49333C>A), E537L (49340G>C 49341A>T), W540Y (49350G>A 49351G>C), R545K (49364C>A 49365G>A 49366C>G), D552R (49385G>C 49386A>G), H565E (49424C>G 49426C>A), I566L (49427A>C 49429C>G), T567A (49430A>G), S569D (49436A>G 49437G>A), S570T (49439T>A 49441G>C)								
DNA methylase N-4/N-6 domain protein (YP_009981890.1)	180	444	34.6%	755.0	67.2%	153 (96.8%)	97 (61.4%)	1/4/0/0	0
	V188I (50652G>A), Q190E (50658C>G 50660A>G), K193A (50667A>G 50668A>C 50669A>C), S194E (50670T>G 50671C>A), N195R (50673A>C 50674A>G 50675C>G), I197H (50679A>C 50680T>A), R199_S200insM (50687_50688insATG), S200T (50688T>A), Q201E (50691C>G 50693G>A), R202G (50694C>G), E203P (50697G>C 50698A>C), D204V (50701A>T), R206_H207del (50706_50711delCGGCAC), E208A (50713A>C), H209N (50715C>A), S210T (50718T>A), Q211D (50721C>G 50723A>C), R212G (50724C>G 50726C>T), Q213R (50728A>G), T214del (50730_50732delACT), A215G (50734C>G 50735T>G), A216T (50736G>A), S217N (50740G>A), E218D (50744G>C), E219D (50747G>C), T220del (50748_50750delACG), N222R (50754A>C 50755A>G), F226L (50768C>G), V227C (50769G>T 50770T>G 50771C>T), N230A (50778A>G 50779A>C), I237V (50799A>G 50801C>G), L238F (50802C>T 50804G>T), V242T (50814G>A 50815T>C), D247E (50831T>G), V260K (50868G>A 50869T>A 50870C>A), E378D (51224G>C), E381A (51232A>C), P384A (51240C>G), C398L (51282T>C 51283G>T), S400K (51288T>A 51289C>A 51290C>A), E403Q (51297G>C 51299G>A), L409V (51315C>G 51317G>C), V411I (51321G>A 51323G>C), D412E (51326T>A), P415A (51333C>G 51335G>C), V418A (51343T>C 51344C>T), A419D (51346C>A 51347G>T), A421S (51351G>A 51352C>G 51353G>C), Q422R (51355A>G), K423R (51357A>C 51358A>G), V427L (51369G>C 51371C>T), V429L (51375G>C 51377C>G), D430E (51380C>G), E431D (51383G>C), E433G (51388A>G 51389G>T), L435F (51393C>T), L436R (51396C>A 51397T>G), E438D (51404G>C)								

*: Inserts / Deletes / Misaligned / Frameshifts

Detailed Results of Betapleolipovirus HHPV4 (NC_047943.1)

NGS details

Assembly

**Coverage length**	2024 (2 contig(s))
**Est. depth of coverage**	72.9
**Est. number of reads**	1588
**Ambiguities**	0

??coverage-details?? ??variant-analysis??

Assignment

**Type**	Betapleolipovirus HHPV4 (Taxonomy ID: 2734332)
**Reference Genome**	NC_047943.1 (Length: 15010bp)
**NT Identity (%)**	64.8783
**NT Quality**	0.398221

Alignment

**Alignment score**	806 (NT) + 2761 (AA) = 3567
**Concordance (%)**	47.3768
**Alignment method**	Global, seeded, nucleotide + amino acids (AGA)

Genome Region

Sequence starts at position null and ends at position null relative to the null reference sequence for null.

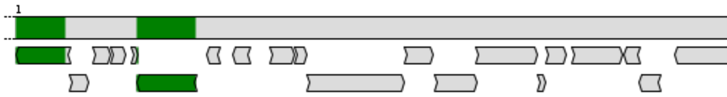

Alignment Detailed Statistics

	Begin	End	Coverage	Score	Concordance	Ma
**NT**	17	3385	13.5%	806.0	24.1%	1799
**CDS**						
HOS97_gp01	37	341	88.2%	1335.0	60.2%	305
HOS97_gp05	32	39	20.5%	10.0	26.3%	8 (
HOS97_gp06	17	382	95.8%	1416.0	72.5%	291
**Proteins**						
tyrosine-type recombinase/integrase (YP_009799432.1)	37	341	88.2%	1335.0	60.2%	305

	Begin	End	Coverage	Score	Concordance	Ma
	E41V (916C>G 917T>A), Q42E (913C>T 915G>C), F43W (910G>C 911A>C), G48_I49insA (894_895insTGC), E50D (889C>G), L52M (885G>T), D54E (877G>T), S56A (873A>C), A6<br>(819C>T), R75K (814G>C 815C>T 816G>T), R75_D76insV (813_814insTAC), I78L (805A>G 807T>G), E79A (803T>G), P80K (799G>T 800G>T 801G>T), I81K (796T>C 797A>T), R8<br>771C>G), R95K (754T>C 755C>T 756G>T), A97C (749G>C 750C>A), A98G (745A>C 746G>C), E99Q (744C>G), V100I (739T>G 741C>T), D101E (736A>C), P104H (728G>T), E105<br>(695G>T), I116L (691G>T 693T>G), S117T (688G>A 689C>G), N118R (686T>C 687T>G), A119D (682A>G 683G>T), G120D (679A>G 680C>T), A124D (667T>A 668G>T), T126Y (66<br>L136V (633G>C), D137E (628G>C), Q140S (620T>G 621G>A), M141R (616C>T 617A>C 618T>G), K143E (612T>C), V148D (596A>T), I151V (586G>C 588T>C), A152W (583A>C 58<br>546T>C), G167A (539C>G), I170L (531T>G), D171R (526A>G 527T>C 528C>G), C173L (521C>A 522A>G), E176D (511T>A), Q177G (508C>T 509T>C 510G>C), D178N (505A>G 50<br>D191G (466A>T 467T>C), G197D (448C>G 449C>T), E198A (445C>G 446T>G), K199R (443T>C 444T>G), G200S (439T>G 441C>T), Q201E (436C>T 438G>C), W203V (431C>A 43<br>V213I (402C>T), E223K (372C>T), S224E (368G>T 369A>C), V225T (364A>C 365A>G 366C>T), F226V (363A>C), E228D (355C>A), R232T (344C>G 345G>T), S238D (325A>G 326<br>309T>C), S245G (305G>C 306A>C), F247I (300A>T), T249K (292C>T 293G>T), T250A (291T>C), M251F (286C>G 288T>A), V254W (277A>C 278A>C 279C>A), W259F (262C>G 26<br>(224T>G 225C>T), E274K (217T>C 219C>T), A275F (215G>A 216C>A), T276A (213T>C), S277T (210A>T), N278F (205A>G 206T>A 207T>A), K280H (199C>G 201T>G), A281V (19<br>152C>G), A300K (140G>T 141C>T), R303N (130A>G 131C>T 132G>T), E304D (127C>G), D305G (124A>G 125T>C), R309G (112A>G 114G>C), R314Q (98C>T), D319E (82G>C), C<br>(36A>C), E336D (31T>A), Q337R (28C>G 29T>C)					
hypothetical protein (YP_009799436.1)	32	39	20.5%	10.0	26.3%	8 (
	E33V (2281A>T 2282G>C), N34I (2284A>T), L35V (2286T>G 2288A>T), *39K (2298T>A)					
HNH endonuclease (YP_009799437.1)	17	382	95.8%	1416.0	72.5%	291
	D20E (3372A>T), D21E (3369A>T), I25A (3358A>G 3359T>C), D27N (3351A>G 3353C>T), S43E (3303G>C 3304G>T 3305A>C), V49I (3285T>A 3287C>T), V51L (3279T>A 3281C>C<br>(3191T>C), S82D (3186C>G 3187G>T 3188A>C), T88A (3168A>C 3170T>C), I93H (3154A>T 3155T>G), G96N (3144A>G 3145C>T 3146C>T), V99I (3137C>T), Q104K (3120T>C 31<br>3110T>C), G109E (3106C>T), E110D (3102T>G), L120V (3072G>A 3074G>C), D123E (3063A>T), T124F (3061G>A 3062T>A), K125Q (3057C>T 3059T>G), E126Q (3054T>C 3056C<br>(3023T>G), V138E (3018T>C 3019A>T), D141E (3009G>C), N142_R144del (3000_3008delACGAAGATT), E146S (2994T>C 2995T>G 2996C>A), D148_L157del (2961_2990delCAGT<br>Q163E (2945G>C), Q165_D226del<br>(2754_2939delATCGGCCGTGTTAAGTCGATACCGGCCTTGCTCATCGAGAAATTCTATTTCATTTTGATCGCGTAGACGCTGCAGTTGTTGGCGAATCTTAGCCCTGATATGATTGTTA<br>I228V (2748T>A 2750T>C), K229E (2747T>C), Q230E (2742C>T 2744G>C), K232R (2736T>A 2737T>C 2738T>G), E236Q (2726C>G), L239T (2716A>G 2717G>T), H240D (2712G><br>S249T (2685A>C 2687A>T), T250E (2683G>T 2684T>C), F252Y (2677A>T), H262A (2646G>T 2647T>G 2648G>C), A265T (2639C>T), E266Q (2634T>C 2636C>G), K269R (2625C><br>V277A (2601T>G 2602A>G), T281S (2590G>C), G282E (2586G>T 2587C>T), T285S (2579T>A), A287D (2572G>T), A305S (2517T>G 2518G>C 2519C>T), I308V (2508G>C 2510T><br>2447T>A), S330T (2442A>G 2444A>T), E332D (2436T>G), Y333H (2435A>G), Q334E (2432G>C), K338E (2420T>C), S347H (2391A>G 2392G>T 2393A>G), D355R (2367A>C 2368<br>(2343G>C), D364E (2340G>T), E366R (2334T>C 2335T>C 2336C>G), P369S (2325T>C 2327G>A), R373E (2314C>T 2315T>C), Q377R (2302T>C), *382N (2286T>G 2288A>T)					

*: Inserts / Deletes / Misaligned / Frameshifts

Detailed Results of Betapleolipovirus HHPV4 (NC_047943.1)

NGS details

Assembly

**Coverage length**	946 (3 contig(s))
**Est. depth of coverage**	49.8
**Est. number of reads**	564
**Ambiguities**	0

??coverage-details?? ??variant-analysis??

Assignment

**Type**	Betapleolipovirus HHPV4 (Taxonomy ID: 2734332)
**Reference Genome**	NC_047943.1 (Length: 15010bp)
**NT Identity (%)**	61.8085
**NT Quality**	0.453488

Alignment

**Alignment score**	429 (NT) + 1451 (AA) = 1880
**Concordance (%)**	45.1381
**Alignment method**	Global, seeded, nucleotide + amino acids (AGA)

Genome Region

Sequence starts at position null and ends at position null relative to the null reference sequence for null.

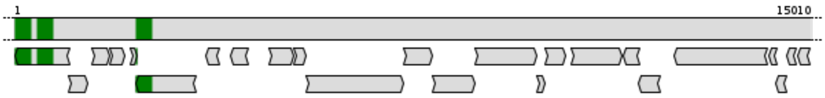

Alignment Detailed Statistics

	Begin	End	Coverage	Score	Concordance	Matches	Identities	I/D/M/F*	Stop Codons
**NT**	2	2607	6.3%	429.0	23.0%	940 (99.4%)	581 (61.4%)	0/6	
**CDS**									
HOS97_gp01	103	346	60.7%	773.0	51.4%	208 (99.0%)	97 (46.2%)	0/2/0/0	0
HOS97_gp05	37	39	7.7%	-3.0	-18.8%	3 (100%)	1 (33.3%)	0/0/0/0	0
HOS97_gp06	276	381	27.7%	681.0	82.8%	106 (100%)	83 (78.3%)	0/0/0/0	0
**Proteins**									
tyrosine-type recombinase/integrase (YP_009799432.1)	103	346	60.7%	773.0	51.4%	208 (99.0%)	97 (46.2%)	0/2/0/0	0

	Begin	End	Coverage	Score	Concordance	Matches	Identities	I/D/M/F*	Stop Codons
	R108S (715C>A 717G>T), T109E (713G>T 714T>C), P112L (704G>A), L113S (700T>G 701A>G 702G>A), T115R (695G>C), S117T (688G>C 689C>G), N118A (685A>C 686T>G 687T>C), A119D (682A>G 683G>T), G120E (679A>T 680C>T), E121N (676C>G 678C>T), V122S (673C>G 674A>G 675C>A), S123R (670A>T 672T>G), A124D (667T>G 668G>T), S125V (664A>C 665G>A 666A>C), T126M (662G>A), D128E (655G>C), P129S (652G>A 653G>C 654G>T), E130D (649C>G), R131H (647C>T), D133S (640A>G 641T>G 642C>A), V134S (638A>G 639C>A), I135V (634A>C 636T>C), D137S (629T>G 630C>A), Y138H (627A>G), Q140E (621G>C), M141K (617A>T), K143E (612T>C), S146G (601T>A 602G>C 603A>C), R147P (598C>T 599C>G), V148Q (595G>C 596A>T 597C>G), I151A (586G>C 587A>G 588T>C), A152L (583A>G 584G>A 585C>G), L153T (581A>G 582G>T), L155M (576A>T), G159M (562A>C 563C>A 564C>T), A160M (559G>C 560G>A 561C>T), M162V (553C>G 555T>C), A164G (547C>G 548G>C), I165V (546T>C), R166H (541A>G 542C>T), G167A (539C>G), L168M (537G>T), I170V (531T>C), C173_E174del (517_522delTTCGCA), L175Y (514A>G 515A>T 516G>A), E176N (511T>G 513C>T), Q177A (508C>T 509T>G 510G>C), N179E (502A>C 504T>C), P180Q (500G>T), G181Y (497C>T 498C>A), I182V (493G>C 495T>C), Q183K (490C>T 492G>T), F184V (489A>C), V185R (484G>C 485A>C 486C>G), Q189D (472C>G 474G>C), L194I (457T>G 459G>T), G197K (449C>T 450C>T), E198G (445C>G 446T>C), K199D (442C>A 444T>C), Q201E (436C>T 438G>C), S243H (311G>T 312A>G), T244R (307C>G 308G>C 309T>G), S245T (304C>T 306A>T), F247L (298G>C 300A>G), T249A (294T>C), T250H (289C>G 290G>T 291T>G), M251C (286C>G 287A>C 288T>A), V254A (278A>G), W259T (263C>G 264A>T), R260Y (259C>A 260C>T 261G>A), A262R (254G>C 255C>G), E271N (226C>A 228C>T), E274D (217T>A), S277H (208C>A 209G>T 210A>G), N278R (205A>T 206T>C 207T>G), R279D (202C>G 203C>T 204G>C), K280A (199C>T 200T>G 201T>C), S282F (193C>G 194G>A), T283E (190C>T 191G>T 192T>C), A287S (179G>C 180C>T), R288V (176C>A 177G>C), D292A (163G>C 164T>G), V293I (162C>T), S295R (154A>C 156T>G), R297S (148C>G), V298I (145G>A 147C>T), A300H (139C>G 140G>T 141C>G), Y301S (136A>T 137T>G), R302L (133C>G 134C>A), R303N (130A>G 131C>T 132G>T), E304S (127C>G 128T>C 129C>T), V306M (121G>C 123C>T), R308E (115G>C 116C>T 117G>C), R309N (112A>G 113C>T 114G>T), V310A (109C>T 110A>G), V311T (107A>G 108C>T), L315A (94C>T 95A>G 96A>C), A317V (88C>T 89G>A), D319K (82G>T 84C>T), Q320M (80T>A 81G>T), I321V (76A>T 78T>C), D323E (70G>T), K324Q (69T>G), G330T (50C>G 51C>T), E331K (48C>T), S335M (34G>C 35G>A 36A>T), D340E (19A>T), P343D (10A>G 11G>T 12G>C), E344D (7T>G), V345L (6C>G)								
hypothetical protein (YP_009799436.1)	37	39	7.7%	-3.0	-18.8%	3 (100%)	1 (33.3%)	0/0/0/0	0
	S37P (2292T>C), *39E (2298T>G)								
HNH endonuclease (YP_009799437.1)	276	381	27.7%	681.0	82.8%	106 (100%)	83 (78.3%)	0/0/0/0	0
	V277A (2601T>A 2602A>G), T281N (2589G>A 2590G>T), G282A (2586G>T 2587C>G), A287D (2572G>T), R301H (2529T>G 2530C>T), E302K (2528C>T), A305S (2517T>G 2518G>C 2519C>T), I308V (2508G>A 2510T>C), S313A (2495A>C), A328S (2448A>T 2450C>A), I329L (2447T>G), E332D (2436T>A), Q334E (2432G>C), K338T (2419T>G), S347H (2392G>T 2393A>G), Q351R (2380T>C), K361N (2349T>A), E362N (2346T>G 2348C>T), E366N (2334T>G 2336C>T), H368D (2328A>G 2330G>C), P369L (2325T>C 2326G>A), L372V (2316G>A 2318G>C)								

*: Inserts / Deletes / Misaligned / Frameshifts

Detailed Results of Halovirus HRTV-4 (NC_021329.1)

NGS details

Assembly

**Coverage length**	2975 (6 contig(s))
**Est. depth of coverage**	21.7
**Est. number of reads**	777
**Ambiguities**	0

??coverage-details?? ??variant-analysis??

Assignment

**Type**	Halovirus HRTV-4 (Taxonomy ID: 1273752)
**Reference Genome**	NC_021329.1 (Length: 35722bp)
**Host(s)**	Halorubrum sp. s5a-3
**NT Identity (%)**	73.0666
**NT Quality**	0.909916

Alignment

**Alignment score**	2707 (NT) + 5026 (AA) = 7733
**Concordance (%)**	61.398
**Alignment method**	Local, heuristic, nucleotide (BLASTN)

Genome Region

Sequence starts at position null and ends at position null relative to the null reference sequence for null.

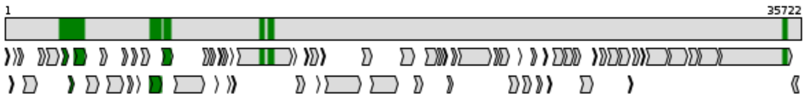

Alignment Detailed Statistics

	Begin	End	Coverage	Score	Concordance	Matches	Identities	I/D/M/F*	Stop Codons
**NT**	2416	35158	8.3%	2707.0	45.7%	2970 (99.7%)	2173 (72.9%)	4/5	
**CDS**									
8	1	105	100%	560.0	79.5%	105 (100%)	83 (79.0%)	0/0/0/0	1
9	1	84	100%	390.0	60.8%	84 (100%)	55 (65.5%)	0/0/0/0	1
10	1	160	83.8%	1084.0	93.3%	160 (100%)	146 (91.3%)	0/0/0/0	0
19	5	181	97.8%	1106.0	85.9%	177 (100%)	143 (80.8%)	0/0/0/0	1

	Begin	End	Coverage	Score	Concordance	Matches	Identities	I/D/M/F*	Stop Codons
20	8	146	82.2%	546.0	53.0%	139 (99.3%)	80 (57.1%)	1/0/1/1	0
30	333	561	22.9%	923.0	68.0%	189 (99.5%)	122 (64.2%)	0/1/0/0	0
72	937	1027	8.3%	417.0	65.1%	91 (100%)	56 (61.5%)	0/0/0/0	0
**Proteins**									
hypothetical protein (YP_008059497.1)	1	105	100%	560.0	79.5%	105 (100%)	83 (79.0%)	0/0/0/0	1
	K3R (2570A>G 2571G>A), I5T (2576T>C), E6D (2580A>C), Q7E (2581C>G), D12E (2598C>A), D13E (2601C>G), P25A (2635C>G), V36M (2668G>A), M39L (2677A>T), E51K (2713G>A 2715A>G), V55I (2725G>A 2727T>C), G75A (2786G>C), D76E (2790C>G), Q84L (2813A>T), I85M (2817T>G), G87D (2822G>A 2823C>T), E88R (2824G>C 2825A>G 2826A>T), V97F (2851G>T), D99G (2858A>G 2859C>T), G100E (2861G>A), S102N (2866T>A 2867C>A 2868G>C), F103Y (2870T>A)								
hypothetical protein (YP_008059498.1)	1	84	100%	390.0	60.8%	84 (100%)	55 (65.5%)	0/0/0/0	1
	A3P (2880G>C), P4T (2883C>A), E6G (2890A>G), M8T (2896T>C), E12D (2909G>T), P14S (2913C>T), P15A (2916C>G 2918G>T), R16A (2919A>G 2920G>C 2921A>G), E18T (2925G>A 2926A>C), G19K (2928G>A 2929G>A 2930T>G), G20A (2932G>C), G21H (2934G>C 2935G>A), K23T (2941A>C), L24P (2944T>C 2945G>A), T25Q (2946A>C 2947C>A 2948T>A), M30N (2962T>A 2963G>C), K31T (2965A>C), S36C (2980C>G), L42F (2999A>C), C45G (3006T>G), S46L (3010C>T), P52L (3028C>T), R67S (3074A>T), N68K (3077C>G), G69A (3079G>C 3080G>A), H70D (3081C>G), T74N (3094C>A), R81G (3114C>G), Y82C (3118A>G)								
hypothetical protein (YP_008059499.1)	1	160	83.8%	1084.0	93.3%	160 (100%)	146 (91.3%)	0/0/0/0	0
	M1V (3118A>G), I23V (3184A>G 3186A>G), D27N (3196G>A), V38L (3229G>C 3231C>T), L42M (3241T>A), D70R (3325G>C 3326A>G 3327C>A), R72H (3332G>A), T75A (3340A>G), T77G (3346A>G 3347C>G), Y90F (3386A>T), R91Q (3389G>A), I107V (3436A>G 3438T>G), E149D (3564A>T), V156E (3584T>A 3585G>A)								
hypothetical protein (YP_008059508.1)	5	181	97.8%	1106.0	85.9%	177 (100%)	143 (80.8%)	0/0/0/0	1
	S14T (6534G>C 6535T>A), N15Q (6536A>C 6538C>A), S20G (6551A>G), L25I (6566C>A 6568T>A), A26S (6569G>T 6571G>A), K28R (6575A>C 6576A>G 6577G>C), T30D (6581A>G 6582C>A 6583G>T), N41A (6614A>G 6615A>C 6616C>T), K44E (6623A>G), A51Q (6644G>C 6645C>A 6646C>A), Y57W (6663A>G 6664C>G), G65C (6686G>T 6688G>T), S66G (6689T>G 6690C>G 6691G>A), S81T (6735G>C 6736C>G), K86E (6749A>G), K89Q (6758A>C), E101N (6794G>A 6796G>C), S102T (6798G>C 6799T>C), H112K (6827C>A 6829C>G), T116L (6839A>T 6840C>T), I118L (6845A>T 6847C>G), E120K (6851G>A), E123N (6860G>A 6862G>T), I132V (6887A>G 6889C>G), K137D (6902A>G 6904A>C), K142H (6917A>C 6919A>T), Q145K (6926C>A), Y151Q (6944T>C 6946C>A), D154N (6953G>A 6955T>C), K156V (6959A>G 6960A>T 6961G>C), Q164S (6983C>T 6984A>C), Y171W (7005A>G 7006C>G), K179E (7028A>G 7030G>A), V180L (7031G>C 7033G>A)								
hypothetical protein (YP_008059509.1)	8	146	82.2%	546.0	53.0%	139 (99.3%)	80 (57.1%)	1/0/1/1	0
	S16T (7119G>C 7120T>G), G19H (7127G>C 7128G>A 7129T>C), E20P (7130G>C 7131A>C), A24P (7142G>C), E26D (7150G>C), N32D (7166A>G 7168C>T), V33G (7170T>G 7171T>C), E35G (7176A>G 7177G>C), T36A (7178A>G 7180G>A), P37Q (7182C>A), D38A (7185A>C), G39E (7188G>A 7189C>A), G40P (7190G>C 7191G>C 7192C>A), G44L (7202G>C 7203G>T 7204A>C), K47E (7211A>G 7213A>G), D48Q (7214G>C 7216C>A), D50E (7222C>A), L51I (7223C>A 7225G>T), I52K (7227T>A 7228T>G), D53E (7231C>A), R54I (7233G>T 7234G>C), V55I (7235G>A 7237G>C), A56V (7239C>T), D57G (7242A>G 7243C>T), R58E (7244C>G 7245G>A 7246C>G), A60Q (7250G>C 7251C>A 7252C>G), K62G (7256A>G 7257A>G 7258G>C), D63A (7260A>C 7261C>A), A66P (7268G>C), I72G (7286A>G 7287T>G), I75V (7295A>G), E77K (7301G>A), D78R (7304G>C 7305A>G), A82S (7316G>T 7318G>A), R86K (7328C>A 7329G>A 7330T>A), E89_D90insS (7339_7340insTCG), D90E (7342C>A), G91T (7343G>A 7344G>C 7345G>A), A95R (7355G>C 7356C>G 7357C>T), K96D (7358A>G 7360G>C), K99D (7367A>G 7369G>C), R103H (7380G>A), K106R (7388A>C 7389A>G 7390G>A), G107I (7391G>A 7392G>T), G109S (7397G>T 7398G>C 7399T>A), R110K (7400C>A 7401G>A), Q111E (7403C>G), A114R (7412G>C 7413C>G), E115N (7415G>A 7417A>C), N119R (7427A>C 7428A>G 7429C>G), L120C (7430C>T 7431T>G 7432C>T), R121K (7433C>A 7434G>A 7435C>G), Q123T (7439C>A 7440A>C 7441G>C), G124D (7443G>A), M134H (7472A>C 7473T>A 7474G>C), K135D (7475A>G 7477A>C), Q140T (7490C>A 7491A>C 7492A>C), D143K (7499G>A 7501C>A), D144E (7504C>G)								
hypothetical protein (YP_008059519.1)	333	561	22.9%	923.0	68.0%	189 (99.5%)	122 (64.2%)	0/1/0/0	0
	F338E (11438T>G 11439T>A 11440C>G), A339T (11441G>A), L340I (11444T>A 11446G>C), A344D (11457C>A 11458G>C), K346M (11463A>T), N348T (11469A>C 11470T>A), S351T (11477T>A), F352Y (11481T>A 11482T>C), S353T (11484G>C 11485C>G), T354A (11486A>G), F356W (11493T>G 11494T>G), V357C (11495G>T 11496T>G), R359K (11501C>A 11502G>A 11503C>A), M361L (11507A>T), R362K (11510C>A 11511G>A), V364I (11516G>A 11518G>C), I365L (11519A>C 11521T>C), R366D (11522C>G 11523G>A 11524G>C), D367K (11525G>A 11527C>A), H373G (11543C>G 11544A>G 11545T>G), T376P (11552A>C 11554A>G), T378Q (11558A>C 11559C>A 11560G>A), A380R (11564G>C 11565C>G), I381L (11567A>C 11569T>C), D382E (11572C>A), E386T (11582G>A 11583A>C 11584G>T), D387G (11586A>G 11587T>G), I388K (11589T>A 11590T>A), G389D (11592G>A), E390D (11596G>C), T391M (11598C>T), N396S (11613A>G), S397R (11615T>C 11616C>G), I398V (11618A>G), D400G (11625A>G), N404T (11637A>C), A405N (11639G>A 11640C>A), R409S (11651C>T 11652G>C), R410K (11654C>A 11655G>A), G464T (11816G>A 11817G>C 11818C>A), D465P (11819G>C 11820A>C 11821C>G), Y466del (11822_11824delTAC), D468Q (11828G>C 11830C>A), A469D (11832C>A), A470V (11835C>T 11836G>A), N473A (11843A>G 11844A>C 11845C>A), R475Q (11850G>A 11851C>A), M476A (11852A>G 11853T>C), E479D (11863A>C), K481R (11867A>C 11868A>G 11869A>C), D486N (11882G>A), R493Q (11904G>A 11905C>A), E497Q (11915G>C), D507S (11945G>A 11946A>G), L508V (11948T>G), D511E (11959T>G), E514N (11966G>A 11968G>C), A517V (11976C>T), S534A (12026A>G 12027G>C 12028C>A), D535N (12029G>A), E551A (12078A>C), Q554E (12086C>G 12088A>G), R555N (12089C>A 12090G>A), S557V (12095A>G 12096G>T 12097C>A), E558D (12100A>T), Q559E (12101C>G)								
hypothetical protein (YP_008059561.1)	937	1027	8.3%	417.0	65.1%	91 (100%)	56 (61.5%)	0/0/0/0	0
	N939D (34892A>G), E942Q (34901G>C 34903G>A), R944N (34907C>A 34908G>A 34909G>C), A946N (34913G>A 34914C>A 34915A>C), D951N (34928G>A), S953D (34934A>G 34935G>A), G954N (34937G>A 34938G>A 34939C>T), L955E (34940T>G 34941T>A), Y957F (34947A>T 34948C>T), S965Q (34970A>C 34971G>A 34972C>A), I970F (34985A>T 34987C>T), N971D (34988A>G 34990C>T), T977S (35007C>G), V978T (35009G>A 35010T>C 35011G>A), S982E (35021A>G 35022G>A 35023T>A), N984D (35027A>G 35029T>C), Q985T (35030C>A 35031A>C), G986S (35033G>A), Y996F (35064A>T 35065C>T), G997S (35066G>A 35068A>C), S998D (35069T>G 35070C>A 35071G>C), R999G (35072C>G 35074G>C), T1000Q (35075A>C 35076C>A 35077G>A), T1001D (35078A>G 35079C>A 35080G>C), A1002R (35081G>C 35082C>G 35083C>A), Q1005L (35091A>T 35092A>T), Q1006D (35093C>G 35095A>C), F1008N (35099T>A 35100T>A), N1009S (35102A>T 35103A>C 35104C>A), I1013V (35114A>G), Q1014A (35117C>G 35118A>C 35119G>T), D1015A (35121A>C 35122T>G), D1017N (35126G>A), N1021D (35138A>G), V1025I (35150G>A 35152G>C)								

*: Inserts / Deletes / Misaligned / Frameshifts

Detailed Results of Halovirus HGTV-1 (NC_021328.1)

NGS details

Assembly

**Coverage length**	14078 (41 contig(s))
**Est. depth of coverage**	13.1
**Est. number of reads**	2231
**Ambiguities**	0

??coverage-details?? ??variant-analysis??

Assignment

**Type**	Halovirus HGTV-1 (Taxonomy ID: 1273749)
**Reference Genome**	NC_021328.1 (Length: 143855bp)
**Host(s)**	Halogranum sp. SS5-1
**NT Identity (%)**	68.5455
**NT Quality**	0.726168

Alignment

**Alignment score**	10223 (NT) + 23220 (AA) = 33443
**Concordance (%)**	55.5581
**Alignment method**	Local, heuristic, nucleotide (BLASTN)

Genome Region

Sequence starts at position null and ends at position null relative to the null reference sequence for null.

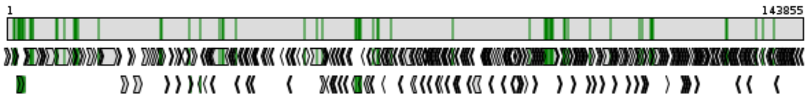

Alignment Detailed Statistics

	Begin	End	Coverage	Score	Concordance	Matches	Identities	I/D/M/F*	Stop Codons
**NT**	1111	138694	9.8%	10223.0	36.7%	14017 (98.8%)	9680 (68.3%)	105/61	
**CDS**									
3	51	203	75.4%	760.0	74.8%	152 (99.3%)	105 (68.6%)	0/1/0/0	1
4	1	39	31.7%	163.0	59.9%	39 (100%)	20 (51.3%)	0/0/0/0	0
5	10	432	91.3%	1870.0	67.1%	400 (99.3%)	290 (72.0%)	3/0/2/2	1
7	163	408	31.7%	1027.0	64.9%	233 (95.5%)	148 (60.7%)	10/1/0/0	0

	Begin	End	Coverage	Score	Concordance	Matches	Identities	I/D/M/F*	Stop Codons
11	28	548	24.5%	1165.0	75.6%	217 (99.1%)	156 (71.2%)	1/1/0/0	0
13	1	279	95.0%	1327.0	76.5%	265 (98.5%)	189 (70.3%)	4/0/0/0	1
19	4	77	49.7%	323.0	67.7%	74 (98.7%)	48 (64.0%)	1/0/0/0	0
31	1	153	47.1%	669.0	66.5%	150 (96.8%)	93 (60.0%)	2/3/0/0	0
39	188	248	24.6%	299.0	66.6%	61 (100%)	35 (57.4%)	0/0/0/0	1
40	1	6	5.8%	12.0	32.4%	6 (100%)	2 (33.3%)	0/0/0/0	0
42	4	66	33.0%	331.0	78.6%	63 (100%)	49 (77.8%)	0/0/0/0	0
49	274	580	22.9%	1114.0	71.3%	220 (100%)	145 (65.9%)	0/0/0/0	0
52	24	112	59.3%	409.0	58.3%	89 (96.7%)	47 (51.1%)	3/0/0/0	0
106	64	102	19.4%	219.0	75.0%	39 (100%)	28 (71.8%)	0/0/0/0	0
108	358	737	13.9%	609.0	75.5%	109 (100%)	82 (75.2%)	0/0/0/0	0
124	24	444	66.9%	2059.0	72.0%	407 (99.8%)	284 (69.6%)	1/0/0/0	0
129	139	497	46.9%	972.0	57.9%	229 (94.2%)	140 (57.6%)	4/10/1/1	0
134	105	187	24.9%	356.0	67.4%	82 (96.5%)	54 (63.5%)	2/1/0/0	0
163	84	151	38.4%	406.0	76.0%	68 (98.6%)	50 (72.5%)	1/0/0/0	0
197	134	204	18.9%	389.0	74.2%	71 (100%)	48 (67.6%)	0/0/0/0	0
203	146	675	58.4%	2603.0	71.8%	509 (99.6%)	358 (70.1%)	2/0/0/0	0
207	1	319	82.9%	1573.0	77.4%	281 (100%)	212 (75.4%)	0/0/0/0	0
217	16	83	21.2%	398.0	79.3%	68 (100%)	54 (79.4%)	0/0/0/0	0
223	382	455	15.5%	417.0	76.1%	74 (100%)	55 (74.3%)	0/0/0/0	0
236	75	305	48.3%	837.0	75.5%	151 (99.3%)	113 (74.3%)	0/1/0/0	0
241	47	251	79.8%	1048.0	67.4%	204 (99.5%)	127 (62.0%)	0/1/0/0	0
279	14	196	66.5%	798.0	62.2%	182 (97.8%)	111 (59.7%)	3/1/0/0	0
294	88	180	40.8%	407.0	65.8%	93 (100%)	57 (61.3%)	0/0/0/0	0
296	105	143	25.0%	276.0	80.2%	39 (100%)	28 (71.8%)	0/0/0/0	0
300	66	146	55.5%	384.0	74.9%	81 (100%)	60 (74.1%)	0/0/0/0	1
**Proteins**									
hypothetical protein (YP_008059211.1)	51	203	75.4%	760.0	74.8%	152 (99.3%)	105 (68.6%)	0/1/0/0	1
	A51G (1112C>G), T52A (1114A>G), K54E (1120A>G), L56N (1126T>A 1127T>A 1128A>C), N58Y (1132A>T), A59R (1135G>A 1136C>G 1137C>G), K61T (1142A>C), Y65F (1154A>T), N67D (1159A>G 1161C>T), K73E (1177A>G), S76T (1186T>A 1188G>C), S78Q (1192A>C 1193G>A 1194C>A), R80K (1199G>A 1200A>G), A81Q (1201G>C 1202C>A 1203G>A), V87I (1219G>A 1221A>T), E88G (1223A>G 1224A>T), L91M (1231C>A 1233C>G), E92D (1236A>T), M94A (1240A>G 1241T>C 1242G>C), G96F (1246G>T 1247G>T 1248T>C), G97K (1249G>A 1250G>A 1251T>G), D98E (1254C>G), D99E (1257C>G), P100D (1258C>G 1259C>A), S101del (1261_1263delTCG), L105Y (1274T>A 1275G>T), T118L (1312A>C 1313C>T), K119R (1315A>C 1316A>G 1317A>T), V128K (1342G>A 1343T>A), K133R (1357A>C 1358A>G 1359G>A), E148Q (1402G>C), Q150E (1408C>G 1410A>G), A153V (1418C>T), I154L (1420A>C 1422T>C), N162D (1444A>G 1446T>C), L165M (1453C>A), Q168K (1462C>A), I174L (1480A>C 1482C>A), Q181D (1501C>G 1503G>C), N182K (1506C>G), K186A (1516A>G 1517A>C), D191T (1531G>A 1532A>C 1533C>A), A194S (1540G>T 1542G>A), A195R (1543G>C 1544C>G 1545G>C), E198Q (1552G>C 1554A>G), A199E (1556C>A), E201D (1563G>T), N202G (1564A>G 1565A>G)								
hypothetical protein (YP_008059212.1)	1	39	31.7%	163.0	59.9%	39 (100%)	20 (51.3%)	0/0/0/0	0

	Begin	End	Coverage	Score	Concordance	Matches	Identities	I/D/M/F*	Stop Codons
	M1L (1572A>C 1574G>T), K2V (1575A>G 1576A>T 1577A>G), V3L (1578G>T 1580T>A), S8G (1593T>G 1594C>G 1595C>G), T13A (1608A>G 1610C>T), L15M (1614T>A 1616A>G), L16V (1617C>G 1619C>T), V18Q (1623G>C 1624T>A 1625C>G), D22G (1636A>G 1637T>A), V25A (1645T>C 1646A>T), M26I (1649G>T), S28K (1653T>A 1654C>A), E29S (1656G>T 1657A>C 1658G>T), T30L (1659A>C 1660C>T), F31Y (1663T>A), D32N (1665G>A 1667C>T), Y33F (1669A>T), N35E (1674A>G 1676C>G)								
terminase large subunit (YP_008059213.1)	10	432	91.3%	1870.0	67.1%	400 (99.3%)	290 (72.0%)	3/0/2/2	1
	H19E (1987C>G 1989T>G), Y20F (1991A>T), L21I (1993T>A 1995G>T), D22G (1997A>G 1998C>T), E23V (2000A>T 2001G>C), D31E (2025C>G), N36P (2038A>C 2039A>C 2040C>T), A37S (2041G>T 2043C>T), S38Q (2044T>C 2045C>A), A41_A42insX (2055_2056insT), A42S (2056G>T), C44P (2062T>C 2063G>C 2064T>C), T52R (2086A>C 2087C>G 2088G>T), M53T (2090T>C 2091G>T), S55A (2095T>G 2097T>G), Y61K (2113T>A 2115T>A), G62A (2117G>C 2118C>A), H65F (2125C>T 2126A>T), N67G (2131A>G 2132A>G 2133C>G), A68S (2134G>A 2135C>G 2136C>T), I70V (2140A>G 2142T>A), E86Q (2188G>C 2190G>A), Q90E (2200C>G 2202G>A), D95N (2215G>A), I96V (2218A>G 2220T>C), E97G (2222A>G 2223G>T), D99E (2229C>A), Q100E (2230C>G 2232A>G), T104S (2242A>T 2244G>A), D114Q (2272G>C 2274C>G), C121S (2294G>C 2295T>C), T126R (2308A>C 2309C>G 2310T>A), T136A (2338A>G 2340T>C), L138Y (2344C>T 2345T>A), N148E (2374A>G 2376T>G), S165G (2425T>G 2426C>G), S166T (2429G>C 2430T>G), L180F (2472G>C), E182Q (2476G>C), E182_R183insX (2478_2479insA), F184* (2483T>G 2484C>A), E187A (2492A>C 2493G>C), K188D (2494A>G 2496G>C), T191D (2503A>G 2504C>A 2505A>C), L192Q (2507T>A 2508T>G), Q193R (2510A>G 2511G>T), W197E (2521T>G 2522G>A), A198E (2525C>A 2526T>G), K224L (2602A>C 2603A>T 2604A>C), V228E (2615T>A 2616A>G), E229A (2618A>C), S230A (2620T>G 2622C>T), A231S (2623G>T 2625A>T), T236Q (2638A>C 2639C>A 2640A>G), S247K (2672G>A 2673T>A), D249E (2679T>G), K251E (2683A>G), K253E (2689A>G), Q254V (2692C>G 2693A>T), A256T (2698G>A), Y257F (2702A>T), L262V (2716T>G 2718G>T), S264A (2722T>G 2724C>A), T265S (2726C>G 2727G>C), G267A (2732G>C 2733T>A), G277A (2762G>C 2763T>C), D278E (2766T>A), D283N (2779G>A), I284V (2782A>G 2784T>C), K288A (2794A>G 2795A>C 2796G>C), N289D (2797A>G 2799T>C), S291P (2803T>C), M292L (2806A>C), D294E (2814C>A), G297N (2821G>A 2822G>A 2823T>C), R300K (2830C>A 2831G>A 2832T>A), S304A (2842T>G 2844G>C), T309A (2857A>G 2859G>A), M312L (2866A>C 2868G>C), I313V (2869A>G 2871T>A), S315A (2875A>G 2876G>C 2877C>G), T320Q (2890A>C 2891C>A 2892T>A), Q325T (2905C>A 2906A>C 2907A>G), V326L (2908G>C), K327Q (2911A>C 2913G>A), L330V (2920C>G 2922C>T), R332P (2927G>C 2928C>A), H354Q (2994T>G), D357Q (3001G>C 3003T>G), R361S (3013C>T 3014G>C), Y362F (3017A>T), S363Y (3020C>A 3021G>C), F364Y (3023T>A 3024T>C), D369N (3037G>A), E370D (3042A>C), P371L (3044C>T), E372D (3048G>C), N373E (3049A>G 3051T>A), N373_K374insV (3051_3052insGTA), D377N (3061G>A), Y384R (3082T>C 3083A>G 3084T>A), E385S (3085G>A 3086A>G 3087A>T), S388P (3094A>C 3095G>C 3096T>C), T389G (3097A>G 3098C>G 3099G>T), H395E (3115C>G 3117T>A), H396A (3118C>G 3119A>C 3120C>T), N419R (3188A>G 3189T>A), L420H (3191T>A), E426G (3209A>G 3210A>T)								
hypothetical protein (YP_008059215.1)	163	408	31.7%	1027.0	64.9%	233 (95.5%)	148 (60.7%)	10/1/0/0	0
	F163M (4091T>A 4093C>G), V171A (4116T>C 4117T>A), V172N (4118G>A 4119T>A 4120T>C), N176G (4130A>G 4131A>G 4132T>A), S178A (4136T>G 4138G>A), E184D (4156G>C), D186E (4162T>A), N187K (4165C>A), V188E (4167T>A 4168T>G), Y189E (4169T>G 4171T>G), E190W (4172G>T 4173A>G), A192P (4178G>C 4180G>A), T194L (4184A>C 4185C>T), D196P (4190G>C 4191A>C 4192T>A), D197E (4195T>G), E199K (4199G>A 4201A>G), I207L (4223A>C), G211del (4235_4237delGGT), T213V (4241A>G 4242C>T 4243T>G), R214S (4244C>T 4245G>C 4246A>G), Q217R (4254A>G 4255G>T), Q217_P218insSEKIR (4255_4256insTCGGAGAAAATCCGC), P218R (4257C>G), I219V (4259A>G 4261C>T), E220G (4263A>G 4264G>A), D221A (4266A>C 4267T>A), R222G (4268A>G), Y223P (4271T>C 4272A>C 4273T>A), N224D (4274A>G), E226D (4282G>C), E226_N227insP (4282_4283insCCG), K228R (4286A>C 4287A>G 4288A>T), I229V (4289A>G), A230G (4293C>G), E235D (4309G>C), F239L (4319T>C), T240E (4322A>G 4323C>A 4324G>A), A243N (4331G>A 4332C>A), E247D (4345A>C), V248I (4346G>A 4348A>C), L254I (4364C>A 4366T>C), A256S (4370G>T 4372A>C), E262D (4390G>T), S269Q (4409T>C 4410C>A 4411T>G), M286R (4460A>C 4461T>G 4462G>C), E289V (4470A>T 4471G>C), R300N (4502C>A 4503G>A 4504T>C), S301N (4505T>A 4506C>A), K304E (4514A>G 4516G>A), A305H (4517G>C 4518C>A 4519G>C), N310E (4532A>G 4534T>G), S324E (4574A>G 4575G>A 4576T>A), V325I (4577G>A), K326D (4580A>G 4582G>T), I328V (4586A>G), E334N (4604G>A 4606G>C), Y345W (4638A>G 4639C>G), I347L (4643A>C), P353A (4661C>G 4663G>T), K354R (4664A>C 4665A>G), Y355Q (4667T>C 4669T>A), E362G (4689A>G), E362_S363insQASG (4690_4691insCAAGCATCTGGT), S363G (4691A>G), V364G (4695T>G 4696G>T), G365A (4698G>C 4699G>A), Q366E (4700C>G 4702G>A), I367V (4703A>G 4705T>A), V370M (4712G>A 4714T>G), Q373E (4721C>G), Q374N (4724C>A 4726G>C), D375N (4727G>A 4729T>C), V376I (4730G>A 4732T>C), Q377V (4733C>G 4734A>T 4735G>T), T381E (4745A>G 4746C>A 4747T>G), E382D (4750G>T), R385K (4757C>A 4758G>A 4759T>G), E388S (4766G>T 4767A>C 4768G>T), S389H (4769T>C 4770C>A 4771T>C), E390R (4772G>C 4773A>G 4774G>A), N392T (4779A>C 4780C>T), V394C (4784G>T 4785T>G 4786C>T), V395I (4787G>A), R396Q (4791G>A 4792T>G), E397D (4795G>C), F404L (4814T>C)								
prohead protease (YP_008059219.1)	28	548	24.5%	1165.0	75.6%	217 (99.1%)	156 (71.2%)	1/1/0/0	0
	Y31W (8913A>G 8914T>G), V36I (8927G>A), L38I (8933C>A), S40A (8939T>G 8941C>A), G41D (8943G>A), H45K (8954C>A 8956C>A), L53F (8980A>T), G56A (8988G>C 8989T>C), K60Q (8999A>C), K61E (9002A>G), T65S (9014A>T 9016G>T), Q67E (9020C>G), K69R (9026A>C 9027A>G 9028G>A), N70P (9029A>C 9030A>C 9031C>T), N76D (9047A>G 9049T>C), S80A (9059A>G 9060G>C 9061C>A), V86I (9077G>A 9079G>T), Y91W (9093A>G 9094T>G), E93D (9100A>T), F104Y (9132T>A 9133T>C), E107D (9142A>T), L108Y (9143C>T 9144T>A), K113D (9158A>G 9160G>C), D114N (9161G>A), I121V (9182A>G 9184T>C), D127P (9200G>C 9201A>C 9202C>A), D129E (9208T>G), E132D (9217G>C), E133R (9218G>C 9219A>G), E133_N134insR (9220_9221insCGT), N134P (9221A>C 9222A>C), E135K (9224G>A 9226G>A), V138A (9234T>C), V140I (9239G>A), G143N (9248G>A 9249G>A), E462Q (10205G>C 10207G>A), K465S (10214A>T 10215A>C 10216G>T), M470Q (10229A>C 10230T>A 10231G>A), Q477D (10250C>G 10252G>T), G479D (10257G>A), D483N (10268G>A), D487E (10282T>A), Q491R (10292C>A 10293A>G 10294G>A), S498N (10313T>A 10314C>A 10315A>C), G499N (10316G>A 10317G>A 10318T>C), S501T (10322T>A 10324C>T), I507V (10340A>G 10342C>T), E508A (10344A>C 10345G>T), Q512A (10355C>G 10356A>C 10357G>T), N514R (10361A>C 10362A>G 10363C>G), R517Q (10371G>A 10372T>A), Q518E (10373C>G 10375G>A), S520A (10379T>G), M522Q (10385A>C 10386T>A), S523del (10388_10390delTCT), D524G (10392A>G 10393T>A), D525E (10396T>A), K528R (10403A>C 10404A>G 10405G>C), R529E (10406C>G 10407G>A 10408C>A), D530N (10409G>A), D542E (10447T>A), D546S (10457G>T 10458A>C)								
major capsid protein (YP_008059221.1)	1	279	95.0%	1327.0	76.5%	265 (98.5%)	189 (70.3%)	4/0/0/0	1
	T4K (12005C>A), T5V (12007A>G 12008C>T), E11R (12025G>C 12026A>G 12027G>C), V21I (12055G>A), L22V (12058C>G), N25D (12067A>G), Y28W (12077A>G 12078C>G), S36D (12100A>G 12101G>A), Q41G (12115C>G 12116A>G 12117G>C), S44A (12124T>G), N48F (12136A>T 12137A>T), I49V (12139A>G 12141T>G), S56T (12160T>A 12162G>A), S58E (12166T>G 12167C>A 12168C>G), V59I (12169G>A), V60I (12172G>A 12174T>C), A61G (12176C>G 12177T>G), E67P (12193G>C 12194A>C 12195G>A), S70K (12203G>A 12204T>G), S72T (12208T>A), V73L (12211G>C), N74R (12214A>C 12215A>G), Q75E (12217C>G), V76I (12220G>A 12222A>C), T79N (12230C>A), K81D (12235A>G 12237G>C), Y83F (12242A>T 12243T>C), G85D (12248G>A), I99V (12289A>G 12291T>G), F101M (12295T>A 12297C>G), E105Q (12307G>C), V106I (12310G>A 12312A>T), A112R (12328G>C 12329C>G 12330G>T), E115Q (12337G>C), K116H (12340A>C 12342A>C), D119E (12351T>A), E120K (12352G>A), F122Y (12359T>A), T123E (12361A>G 12362C>A), E124Q (12364G>C 12366A>G), D126E (12372C>A), N127H (12373A>C), N128I (12377A>T), V129D (12380T>A 12381T>C), D130E (12384T>G), S131T (12385T>A), T132A (12388A>G), T132_V133insSGDD (12390_12391insAGTGGTGACGAC), V133I (12391G>A 12393T>C), S136G (12400A>G 12402C>T), D138G (12407A>G), V139E (12410T>A 12411T>A), S143A (12421T>G 12423G>C), A151G (12446C>G 12447C>A), Q154S (12454C>T 12455A>C 12456G>T), N155Y (12457A>T), N158T (12467A>C 12468C>A), S176D (12520A>G 12521G>A 12522C>T), E183D (12543A>T), S184G (12544A>G), A190R (12562G>C 12563C>G), T191S (12566C>G), V194I (12574G>A), Q196R (12581A>G 12582G>T), R201D (12595C>G 12596G>A 12597A>C), F226Y (12671T>A), T230S (12682A>T 12684G>C), A231N (12685G>A 12686C>A 12687G>C), H232Y (12688C>T), V242I (12718G>A 12720C>T), S243A (12721T>G), E248N (12736G>A 12738G>C), F260Y (12773T>A), K266A (12790A>G 12791A>C 12792G>A), A267T (12793G>A 12795A>C), V274A (12815T>C)								
hypothetical protein (YP_008059227.1)	4	77	49.7%	323.0	67.7%	74 (98.7%)	48 (64.0%)	1/0/0/0	0
	I7M (16417T>G), E11N (16427G>A 16429G>C), T14V (16436A>G 16437C>T 16438G>A), Q15K (16439C>A 16441A>G), Q19N (16451C>A 16453G>T), K20H (16454A>C 16456G>C), S24T (16466T>A), A31S (16487G>T 16489G>T), T32A (16490A>G 16492T>G), M38V (16508A>G 16510G>A), M45T (16530T>C 16531G>A), S48A (16538T>G 16540C>A), Q51E (16547C>G), D55E (16561C>G), E56S (16562G>T 16563A>C 16564G>T), M57L (16565A>C 16567G>T), D59E (16573T>G), D62E (16582C>A), E63D (16585A>C), E65G (16590A>G 16591G>C), E65_D66insG (16591_16592insGGT), D68E (16600T>G), A69V (16602C>T 16603G>T), D72E (16612T>A), A73D (16614C>A 16615G>T), I74M (16618T>G)								
hypothetical protein (YP_008059239.1)	1	153	47.1%	669.0	66.5%	150 (96.8%)	93 (60.0%)	2/3/0/0	0

	Begin	End	Coverage	Score	Concordance	Matches	Identities	I/D/M/F*	Stop Codons
	A5V (27573C>T 27574G>A), I9L (27584A>T 27586T>A), T14S (27600C>G), A15S (27602G>T 27604A>G), H17K (27608C>A 27610C>G), G23A (27627G>C 27628T>A), S28_R30del (27641_27649delTCCTCTCGT), S31G (27650A>G 27652C>A), D32G (27654A>G 27655C>A), P33G (27656C>G 27657C>G), S41N (27681G>A), A46V (27696C>T 27697A>T), Q47T (27698C>A 27699A>C), D49_G50insQ (27706_27707insCAA), G50D (27708G>A 27709T>C), I51L (27710A>T 27712T>G), D52N (27713G>A 27715T>C), T54V (27719A>G 27720C>T), Q55N (27722C>A 27724G>T), F56M (27725T>A 27727T>G), D57N (27728G>A), T58A (27731A>G 27733G>C), S60Q (27737A>C 27738G>A 27739T>A), A65G (27753C>G 27754A>T), S66T (27755T>A 27757T>G), F68L (27761T>C), G69D (27765G>A 27766G>T), I70V (27767A>G), T71D (27770A>G 27771C>A 27772G>C), A73D (27777C>A 27778A>C), P74A (27779C>G 27781G>A), I84L (27809A>C), K86I (27816A>T 27817A>T), D89S (27824G>A 27825A>G), T90A (27827A>G 27829A>G), S91T (27830T>A 27832G>A), L92V (27833T>G 27835G>A), S93T (27837G>C 27838T>G), L94_P95insD (27841_27842insGAC), P95A (27842C>G 27844G>A), S97T (27848T>A 27850G>A), S98A (27851T>G 27853T>A), T99G (27854A>G 27855C>G 27856T>A), T100Q (27857A>C 27858C>A 27859T>G), N109D (27884A>G 27886T>C), T111A (27890A>G 27892T>G), N112D (27893A>G), I116V (27905A>G 27907T>C), T118L (27911A>C 27912C>T 27913G>T), S119A (27914T>G 27916G>T), S120T (27917T>A 27919G>T), N124D (27929A>G), S126T (27935T>A 27937C>A), D128N (27941G>A), T129A (27944A>G 27946T>A), L131Q (27950T>C 27951T>A 27952G>A), K132R (27953A>C 27954A>G 27955A>C), T139D (27974A>G 27975C>A), G142A (27984G>C 27985T>A), S143D (27986T>G 27987C>A)								
PD-(D/E)XK nuclease (YP_008059247.1)	188	248	24.6%	299.0	66.6%	61 (100%)	35 (57.4%)	0/0/0/0	1
	I188M (32893T>G), V190M (32897G>A), V194I (32909G>A 32911C>T), S200T (32927T>A 32929T>A), E201Q (32930G>C), S202T (32933T>A 32935T>G), I205V (32942A>G 32944T>A), D208S (32951G>T 32952A>C 32953T>G), S209I (32955G>T 32956C>A), S212M (32963T>A 32964C>T), K213E (32966A>G), N214D (32969A>G 32971T>C), N216D (32975A>G), I218L (32981A>C 32983T>A), W219Y (32985G>A 32986G>C), G220D (32988G>A 32989C>T), E221G (32991A>G 32992G>C), V223S (32996G>T 32997T>C), S224E (32999A>G 33000G>A 33001C>G), Q227K (33008C>A), T230E (33017A>G 33018C>A), E233D (33028G>T), K237R (33038A>C 33039A>G 33040G>A), K238E (33041A>G 33043G>A), D241K (33050G>A 33052C>G), E242K (33053G>A)								
hypothetical protein (YP_008059248.1)	1	6	5.8%	12.0	32.4%	6 (100%)	2 (33.3%)	0/0/0/0	0
	M1T (33064T>C), T2M (33067C>T), G3A (33070G>C)								
holliday junction resolvase (YP_008059250.1)	4	66	33.0%	331.0	78.6%	63 (100%)	49 (77.8%)	0/0/0/0	0
	E13T (34979T>G 34980C>T), S16K (34970G>T 34971A>T), F19M (34960G>C 34962A>T), L21Y (34955A>T 34956G>A), G23D (34948T>G 34949C>T), S25R (34942A>G 34943G>C 34944A>G), I27L (34936G>C 34938T>G), P31A (34926G>C), V39I (34900T>A 34902C>T), D49N (34872C>T), S51T (34866A>T), L55V (34854A>C), G61N (34835C>T 34836C>T)								
DNA polymerase elongation subunit (family B) (YP_008059257.1)	274	580	22.9%	1114.0	71.3%	220 (100%)	145 (65.9%)	0/0/0/0	0
	E275D (39202C>G), D290E (39157G>C), N293S (39148G>T 39149T>G 39150T>A), Q305E (39112C>T 39114G>C), K306R (39109C>T 39110T>C 39111T>G), I307V (39106A>C 39108T>C), F312Y (39092A>T), S313R (39090T>G), E314D (39085T>G), L318T (39074A>G 39075G>T), N319D (39072T>C), Q320S (39067T>C 39068T>G 39069G>A), T321C (39064C>G 39065G>C 39066T>A), T323V (39058C>G 39059G>A 39060T>C), S326R (39049G>A 39050G>C 39051A>G), T328S (39043C>A 39044G>C), F330V (39037G>A 39039A>C), V331I (39036C>T), K332N (39031C>A), M336L (39021T>A), F337M (39016G>C 39018A>T), L340M (39007A>C 39009G>T), K344M (38996T>A), K352R (38971C>G 38972T>C 38973T>G), S355A (38962G>C 38963C>G 38964T>C), G357D (38957C>T), N361E (38944G>T 38946T>C), D362E (38941G>T), Y366S (38930T>G), E369D (38920T>A), I371V (38914A>C 38916T>C), S373D (38908C>G 38909G>T 38910A>C), E376D (38899C>G), G377A (38896G>T 38897C>G), F380H (38888A>T 38889A>G), E381S (38884C>A 38885T>G 38886C>A), D384E (38875G>C), M386L (38871T>G), R387K (38866T>C 38867C>T 38868G>T), V393T (38848A>C 38849A>G 38850C>T), E394L (38846T>A 38847C>G), A395L (38843G>A 38844C>G), K398E (38833T>C 38835T>C), E401R (38825T>C 38826C>G), A402T (38821T>C 38823C>T), G404S (38815G>A 38816C>G 38817C>A), L406I (38811G>T), H414G (38786T>C 38787G>C), V415I (38782G>A 38784C>T), T416A (38779G>A 38781T>C), T418A (38775T>C), S421T (38765C>G), C423I (38759C>A 38760A>T), V433I (38728C>G 38730C>T), F435Y (38723A>T), Y440F (38708T>A), S441G (38705G>C 38706A>C), G443N (38699C>T 38700C>T), S534K (38425G>T 38426C>T), P535A (38422C>T 38424G>C), I536L (38419A>G 38421T>G), Y538V (38414T>A 38415A>C), T539L (38411G>A 38412T>G), K544Q (38397T>G), E545Q (38392T>C 38394C>G), G546S (38389T>G 38390C>G 38391C>A), T553Q (38368C>T 38369G>T 38370T>G), M554S (38365C>G 38366A>C), M555L (38362C>G 38364T>G), T556I (38360G>A), K566E (38329T>C 38331T>C), S568A (38323G>A 38325A>C), K571A (38314C>T 38315T>G 38316T>C), G573A (38309C>G)								
hypothetical protein (YP_008059260.1)	24	112	59.3%	409.0	58.3%	89 (96.7%)	47 (51.1%)	3/0/0/0	0
	G27Q (41505G>T 41506C>T 41507C>G), Q29H (41499C>G), E30D (41496C>G), D31E (41493G>T), V32L (41490T>C 41492C>A), S33V (41487T>G 41488G>A 41489A>C), D34E (41484G>T), Y39H (41469G>A 41471A>G), D40E (41466G>C), Q46T (41448C>T 41449T>G 41450G>T), I47L (41445A>C 41447T>A), G48N (41442C>A 41443C>T 41444C>T), D49_D50insF (41438_41439insAAA), V51L (41435C>A), D52G (41430G>A 41431T>C), P54D (41425G>T 41426G>C), P54_Y55insEV (41423_41424insCACTTC), D56E (41418A>C), N57R (41415G>A 41416T>C 41417T>G), D59A (41409A>T 41410T>G), I61V (41403A>G 41405T>C), M62V (41400C>A 41402T>C), V65A (41391T>G 41392A>G), V66L (41388G>A 41390C>G), K68A (41383T>G 41384T>C), M69V (41381T>C), K70A (41377T>G 41378T>C), D71S (41374T>G 41375C>A), V72A (41370C>T 41371A>G), S78R (41352C>T 41353G>C 41354A>G), Q80D (41346C>G 41348G>C), A88S (41322T>A 41323G>C 41324C>T), V89A (41319A>C 41320A>G), V90M (41316G>C 41318C>T), Y91E (41313A>T 41315A>C), M92I (41310C>T), Y93K (41307G>C 41309A>T), E94H (41304T>A 41306C>G), K97E (41295T>C 41297T>C), R98H (41292G>A 41293C>T), I100V (41288T>C), I102V (41280G>A 41282T>C), Q107D (41265C>A 41267G>C), K109W (41260T>C 41261T>A)								
TMP kinase (YP_008059282.1)	64	102	19.4%	219.0	75.0%	39 (100%)	28 (71.8%)	0/0/0/0	0
	N75E (53828A>T 53830T>C), R76D (53826C>T 53827G>C), R78K (53819A>C 53820C>T 53821G>T), V81I (53812C>T), Q82A (53807C>A 53808T>G 53809G>C), G84D (53801T>A 53802C>T), Q85K (53800G>T), M86I (53795C>T), I88V (53791T>C), S101P (53750A>T 53752A>G), I102V (53749T>C)								
ribonucleotide reductase (YP_008059284.1)	358	737	13.9%	609.0	75.5%	109 (100%)	82 (75.2%)	0/0/0/0	0
	Q364K (55927C>A 55929G>A), A369V (55943C>T 55944G>A), K371E (55948A>G), H374E (55957C>G 55959T>G), E376R (55963G>C 55964A>G 55965A>C), N378E (55969A>G 55971T>A), A390E (56006C>A 56007A>G), Y391F (56009A>T 56010C>T), A678V (56870C>T 56871A>C), F682G (56881T>G 56882T>G 56883C>A), I691V (56908A>G), A693L (56914G>C 56915C>T 56916T>G), N695S (56920A>T 56921A>C 56922C>T), S696D (56923A>G 56924G>A), A697S (56926G>T), T698N (56930C>A 56931C>T), R699H (56933G>A 56934T>C), N700S (56935A>T 56936A>C), E704D (56949G>T), F706Y (56954T>A 56955T>C), E712P (56971G>C 56972A>C 56973G>C), Q717A (56986C>G 56987A>C), F724V (57007T>G 57009C>G), G728Q (57019G>C 57020G>A 57021T>G), D731E (57030C>G)								
hypothetical protein (YP_008059300.1)	24	444	66.9%	2059.0	72.0%	407 (99.8%)	284 (69.6%)	1/0/0/0	0

	Begin	End	Coverage	Score	Concordance	Matches	Identities	I/D/M/F*	Stop Codons
	D26E (64023G>T), H28S (64017G>T 64018T>G 64019G>A), D29E (64014A>C), A32V (64006G>A), I33N (64003A>T), K37E (63992T>C), R42D (63976C>T 63977G>C), L51T (63948C>G 63949A>G 63950A>T), T53G (63942C>G 63943G>C 63944T>C), V58L (63929C>G), S59T (63924G>A 63925C>G), E63D (63912T>A), S65P (63906C>G 63908A>G), S66G (63903A>T 63904G>C 63905A>C), I69V (63894A>C 63896T>C), S70C (63891T>A 63892G>C), G71W (63888A>C 63890C>A), L73M (63882T>C 63884G>T), T82S (63857T>A), L84I (63849A>G 63851G>T), H86N (63843A>G 63845G>T), Q87V (63840C>A 63841T>A 63842G>C), V88I (63839C>T), D90E (63831G>T), G95R (63818C>G), Q99K (63804C>T 63806G>T), S102T (63795G>A 63796C>G), T106K (63783G>C 63784G>T), A107S (63782C>A), E115D (63756C>A), W118Y (63747C>G 63748C>T), S121A (63738A>T 63739C>G 63740T>C), G129S (63715C>G 63716C>A), E130D (63711C>G), K131E (63710T>C), G141_D142insG (63677_63678insTCC), D142E (63675G>T), E144D (63669C>G), T147R (63660G>A 63661G>C 63662T>G), Q165V (63606C>G 63607T>A 63608G>C), S167K (63600A>C 63601C>T), A168P (63597T>C 63599C>G), I169L (63594G>C 63596T>G), Y171F (63589T>A), W172S (63585C>G 63586C>G), M184L (63551T>G), L188M (63537G>C 63539G>T), V191I (63530C>T), K197E (63510C>T 63512T>C), L204V (63491G>C), K207Q (63482T>G), S211K (63469G>T 63470A>T), I213V (63464T>C), A214S (63461C>A), D219N (63446C>T), H220A (63442T>G 63443G>C), E221Y (63438C>G 63440C>A), H230K (63411G>C 63413G>T), Q254E (63341G>C), R258W (63329G>A), F260A (63321G>T 63322A>G 63323A>C), K262E (63317T>C), Y263H (63314A>G), A264V (63310G>A), T270I (63292G>A), D271N (63288A>G 63290C>T), G279S (63265C>G 63266C>A), A280G (63262G>C), S288A (63237G>T 63239A>C), A290C (63231A>G 63232G>C 63233C>A), Y294H (63221A>G), K295E (63216C>T 63218T>C), S296N (63213A>G 63214C>T), D298E (63207G>C), K299T (63205T>G), A301R (63198G>T 63199G>C 63200C>G), E304V (63190T>A), S305R (63186A>T 63187G>C 63188A>T), N306D (63185T>C), E307D (63180C>G), G308E (63177G>T 63178C>T), L320V (63141G>T 63143G>C), T326C (63124G>C 63125T>A), I329V (63114A>C 63116T>C), M331L (63110T>G), V346L (63063G>C 63065C>G), Q350E (63051C>T 63053G>C), T351K (63048A>C 63049G>T), R355T (63036T>C 63037C>G 63038G>T), L357A (63030G>A 63031A>G 63032G>C), S358A (63027G>C 63029A>C), N361P (63018G>A 63019T>G 63020T>G), Q364I (63009C>G 63010T>A 63011G>T), H366Y (63003A>G 63005G>A), K369E (62996T>C), H378T (62967G>T 62968T>G 62969G>T), I380V (62961A>G 62963T>C), Q382A (62955T>C 62956T>G 62957G>C), A385Q (62947G>T 62948C>G), Q389D (62934C>G 62936G>C), Y391H (62928A>G 62930A>G), Q394E (62919C>T 62921G>C), H396M (62913G>C 62914T>A 62915G>T), E404A (62889T>A 62890T>G), E408K (62877T>C 62879C>T), K410N (62871C>G), W412F (62865C>G 62866C>A), A414Q (62859A>C 62860G>T 62861C>G), F418L (62847A>G 62849A>G), E419D (62844C>G), D421N (62840C>T), D423E (62832G>C), K425Q (62828T>G), D426R (62823G>T 62824T>C 62825C>G), F427V (62820G>C 62822A>C), E429D (62814C>G), T432H (62806G>T 62807T>G), T436K (62793G>C 62794G>T), G437D (62790T>G 62791C>T), E438K (62787T>C 62789C>T), V441I (62778C>A 62780C>T), T443S (62772A>C 62774T>A)								
hypothetical protein (YP_008059305.1)	139	497	46.9%	972.0	57.9%	229 (94.2%)	140 (57.6%)	4/10/1/1	0
	K143R (67170C>G 67171T>C 67172T>G), K144N (67167T>G), D148A (67155A>T 67156T>G), N152Q (67143A>C 67145T>G), F153Y (67140G>A 67141A>T), N154D (67139T>C), Q157E (67130G>C), F158Y (67126A>T), Q169R (67093T>C), R171H (67087C>T), R180T (67059A>T 67060C>G 67061G>T), R182Q (67054C>T), E184D (67047C>G), A188R (67035T>A 67036G>C 67037C>G), Q192K (67023C>T 67025G>T), L195Y (67015A>T 67016G>A), E197N (67008T>G 67010C>T), S200D (67000G>T 67001A>C), P202del (66993_66995delAGG), P206A (66981C>T 66983G>C), L207_K208insX (66977_66978insT), Q209K (66974G>T), D211E (66966A>C), N215T (66954G>A 66955T>G), E216A (66951T>C 66952T>G), M217K (66949A>T), E219D (66942T>G), A220E (66939G>C 66940G>T), G221E (66936A>C 66937C>T), K222T (66933T>G 66934T>G), E223D (66930C>G), G224S (66928C>G 66929C>A), R225_Q227del (66918_66926delCTGACTCCG), A228E (66915A>T 66916G>T), E229D (66912C>G), V230A (66909A>T 66910A>G), E233D (66900T>G), Q234S (66897T>A 66898T>G 66899G>A), E236D (66891T>G), E237D (66888T>G), N238_S243del (66870_66887delCGAGTAGCCATCCTCATT), P245G (66864A>G 66865G>C 66866G>C), I246L (66863T>G), F247K (66858G>C 66859A>T 66860A>T), V250M (66849A>C 66851C>T), R251M (66846G>C 66847C>A 66848G>T), D258Q (66825G>T 66827C>G), A259D (66822G>A 66823G>T), S262T (66813G>C 66815A>T), G263A (66811C>G), E264N (66807C>G 66809C>T), F267L (66800A>G), D272E (66783G>T), R276A (66771T>G 66772C>G 66773G>C), Q279R (66762C>G 66763T>C), M280L (66759C>T 66761T>A), Y281W (66756G>C 66757T>C), I420V (66341T>C), L427I (66320G>T), N429A (66313T>G 66314T>C), D430N (66311C>T), S431T (66306G>C 66308A>T), T432P (66303A>C 66305T>G), W433F (66300C>G 66301C>A), R434T (66297T>C 66298C>G), T435A (66296T>C), N436R (66292T>C 66293T>G), T437D (66288C>A 66289G>T 66290T>C), S438_F439insQT (66284_66285insCGTCTG), F439V (66284A>C), E442A (66273T>C 66274T>G), W443F (66270C>G 66271C>A), K447R (66259T>C 66260T>G), A448D (66255C>G 66256G>T), N450V (66249G>C 66250T>A 66251T>C), P451S (66248G>A), P451_N452insA (66245_66246insCGC), N452D (66245T>C), A453T (66240T>G 66242C>T), S454A (66237G>C 66238C>G 66239T>C), I458V (66227T>C), Q461A (66216T>C 66217T>G 66218G>C), H462A (66214T>G 66215G>C), A472Y (66183A>G 66184G>T 66185C>A), H473E (66180G>C 66182G>C), D474N (66179C>T), M478I (66165C>G), Q484D (66147C>A 66149G>C), V485I (66144G>A 66146C>T), D487R (66139T>C 66140C>G), E490K (66131C>T), K491Q (66126C>T 66128T>G), V492A (66123G>A 66124A>G)								
hypothetical protein (YP_008059310.1)	105	187	24.9%	356.0	67.4%	82 (96.5%)	54 (63.5%)	2/1/0/0	0
	N106T (69509T>G), E107D (69505T>G), V108Y (69503A>T 69504C>A), V110I (69498C>T), N112D (69492T>C), A113S (69487A>C 69489C>A), D114E (69484G>C), K116T (69479T>G), G117D (69476C>T), D119K (69469G>C 69471C>T), L121V (69463A>G 69465G>C), D122E (69460A>C), V124M (69454G>C 69456C>T), E127K (69447C>T), Y128F (69443T>A), K134E (69424C>T 69426T>C), V138_D139insDA (69411_69412insAGCATC), S140N (69407G>T 69408A>T), D142del (69400_69402delGTC), I146A (69388G>T 69389A>G 69390T>C), A148V (69382T>A 69383G>A), S151D (69373A>G 69374C>T 69375T>C), G152R (69370G>T 69372C>G), S155A (69361G>T 69363A>C), T156V (69359G>A 69360T>C), K160R (69346C>G 69347T>C 69348T>G), L162I (69340C>A 69342A>T), E168V (69323T>A), V179P (69289G>C 69290A>G 69291C>G)								
hypothetical protein (YP_008059338.1)	84	151	38.4%	406.0	76.0%	68 (98.6%)	50 (72.5%)	1/0/0/0	0
	M84I (80639C>A), E86S (80633C>A 80634T>G 80635C>A), G87D (80631C>T), F88Y (80627A>G 80628A>T), T91V (80618A>C 80619G>A 80620T>C), P96V (80603C>G 80604G>A 80605G>C), T97S (80600C>G 80602T>A), M103G (80582C>T 80583A>C 80584T>C), S114T (80551A>T), D116E (80543A>C), M117L (80540C>A 80542T>G), S118G (80537C>G 80538G>C 80539A>C), D119A (80534G>T 80535T>G), D120T (80531G>C 80532T>G 80533C>T), S124T (80519C>T 80521A>T), S124_M125insS (80518_80519insGCT), N136Q (80483G>T 80485T>G), R141H (80468A>G 80469C>T)								
class I lysyl tRNA synthetase (YP_008059372.1)	134	204	18.9%	389.0	74.2%	71 (100%)	48 (67.6%)	0/0/0/0	0
	E137G (94355A>G), M139L (94360A>T), L143F (94372C>T), A146P (94381G>C), A150D (94394C>A 94395T>C), T151L (94396A>C 94397C>T 94398C>G), E153D (94404A>C), Y159F (94421A>T), F168W (94448T>G 94449T>G), E169P (94450G>C 94451A>C 94452A>G), A171G (94457C>G), A172V (94460C>T 94461A>C), R173D (94462C>G 94463G>A 94464A>C), E176R (94471G>C 94472A>G 94473A>G), A177T (94474G>A), A178V (94478C>T), I181N (94487T>A), D186P (94501G>C 94502A>C 94503C>G), F187M (94504T>A 94506C>G), D195E (94530C>G), A197E (94535C>A 94536C>G), M199L (94540A>C)								
hypothetical protein (YP_008059378.1)	146	675	58.4%	2603.0	71.8%	509 (99.6%)	358 (70.1%)	2/0/0/0	0

	Begin	End	Coverage	Score	Concordance	Matches	Identities	I/D/M/F*	Stop Codons
	R151Q (97217G>A 97218C>G), T156A (97231A>G 97233G>T), G159M (97240G>A 97241G>T 97242A>G), R163V (97252C>G 97253G>T), L164T (97255C>A 97256T>C), M169L (97270A>C 97272G>T), A183W (97312G>T 97313C>G), R185Q (97319G>A 97320T>G), F187S (97325T>C 97326C>T), R189K (97330C>A 97331G>A), L192M (97339C>A 97341C>G), E194D (97347A>T), S198A (97357A>G 97358G>C), V200Q (97363G>C 97364T>A), Y202F (97370A>T 97371C>T), G211L (97396G>C 97397G>T 97398C>T), A212E (97400C>A 97401A>G), E214D (97407G>C), T215E (97408A>G 97409C>A 97410T>G), E224S (97435G>T 97436A>C 97437G>T), A229G (97451C>G 97452G>C), H233N (97462C>A), A239E (97481C>A 97482A>G), Y241F (97487A>T 97488T>C), R246Q (97502G>A 97503C>A), P248T (97507C>A), N255D (97528A>G), S256E (97531T>G 97532C>A 97533C>G), A262G (97550C>G 97551G>C), E263K (97552G>A), D266E (97563C>G), R270T (97573C>A 97574G>C), N271F (97576A>T 97577A>T 97578T>C), D275Y (97588G>T 97590T>C), S277P (97594A>C 97595G>C 97596C>T), D279N (97600G>A 97602T>C), S280E (97603A>G 97604G>A 97605T>A), T284S (97615A>T 97617G>C), D288E (97629C>G), D297E (97656T>A), K298N (97659G>C), P299Q (97661C>A), T300E (97663A>G 97664C>A), N301I (97667A>T 97668C>T), R302G (97669C>G), P303G (97672C>G 97673C>G 97674C>T), N304H (97675A>C), H306D (97681C>G), E307G (97685A>G 97686A>C), V309I (97690G>A), R318K (97717C>A 97718G>A 97719A>G), L321T (97726C>A 97727T>C 97728C>T), K329S (97750A>T 97751A>C), E331D (97758G>T), K342R (97789A>C 97790A>G 97791G>T), K349Q (97810A>C), A354P (97825G>C 97827C>A), V356I (97831G>A 97833C>T), V362I (97849G>A), E363D (97854G>T), N364D (97855A>G), S365T (97859G>C), D368Q (97867G>C 97869C>A), N369G (97870A>G 97871A>G 97872T>C), M370L (97873A>C 97875G>C), I371G (97876A>G 97877T>G), Q372E (97879C>G 97881G>A), E373D (97884A>C), Q374R (97886A>G 97887G>A), Q375V (97888C>G 97889A>T 97890G>A), A376D (97892C>A), K377D (97894A>G 97896G>C), L379V (97900C>G 97902C>T), V390R (97933G>C 97934T>G), G393K (97942G>A 97943G>A 97944T>G), M402K (97970T>A), H405N (97978C>A), D407E (97986C>G), K410R (97993A>C 97994A>G 97995G>T), C415Q (98008T>C 98009G>A 98010C>G), N417E (98014A>G 98016C>G), E418D (98019A>T), I423V (98032A>G 98034T>G), K425A (98038A>G 98039A>C 98040G>C), E426D (98043G>C), N430E (98053A>G 98055C>G), S432D (98059T>G 98060C>A 98061G>C), S433T (98062T>A), D435Q (98068G>C 98070T>G), G460S (98143G>A 98145G>T), T461N (98147C>A 98148G>T), D473E (98184T>A), G476D (98192G>A), K481V (98206A>G 98207A>T 98208G>C), I487L (98224A>C 98226T>G), K492S (98239A>T 98240A>C), S493M (98243G>T 98244T>G), M495K (98249T>A), H506T (98281C>A 98282A>C 98283C>G), K507L (98284A>C 98285A>T 98286G>C), Q519E (98320C>G), D547N (98404G>A), I551V (98416A>G), Q554S (98425C>T 98426A>C 98427G>C), D556A (98432A>C 98433C>A), S561T (98446T>A 98448T>G), E570D (98475A>C), K575D (98488A>G 98490G>C), N582S (98509A>T 98510A>C 98511C>G), E587Q (98524G>C), R588K (98527C>A 98528G>A 98529T>G), D593P (98542G>C 98543A>C 98544C>G), E598D (98559G>C), E601_N602insSR (98568_98569insTCACGG), N602S (98570A>G 98571C>T), D604T (98575G>A 98576A>C 98577T>A), Y605D (98578T>G), V606R (98581G>C 98582T>G), N608T (98588A>C 98589C>G), T609G (98590A>G 98591C>G 98592G>T), S611G (98596T>G 98597C>G 98598G>T), G613A (98603G>C 98604C>G), G614S (98605G>A), D620N (98623G>A), T622A (98629A>G 98631C>G), K632T (98660A>C 98661G>T), H635R (98669A>G 98670C>G), Y637K (98674T>A 98676C>G), F638L (98679C>A), R640N (98683C>A 98684G>A), S641I (98686T>A 98687C>T 98688G>C), Y645T (98698T>A 98699A>C 98700C>T), I646L (98701A>T 98703C>G), V647E (98705T>A), D648S (98707G>A 98708A>G), Q649R (98711A>G 98712G>C), L650V (98713T>G 98715G>C), E651S (98716G>T 98717A>C), D654E (98727T>G), G655N (98728G>A 98729G>A 98730G>C), I656F (98731A>T 98733T>C), R657S (98734C>A 98736T>C), N660R (98743A>C 98744A>G 98745C>A), T662N (98750C>A 98751G>C), V663C (98752G>T 98753T>G 98754G>C), K664S (98755A>T 98756A>C 98757G>T), I668V (98767A>G), K673E (98782A>G), V674I (98785G>A 98787G>T)								
hypothetical protein (YP_008059382.1)	1	319	82.9%	1573.0	77.4%	281 (100%)	212 (75.4%)	0/0/0/0	0
	R3E (100519C>G 100520G>A), D4N (100522G>A 100524T>C), Y5F (100526A>T), N6D (100528A>G), S8N (100535G>A), E12N (100546G>A 100548A>C), G32D (100607G>A 100608A>C), D35R (100615G>C 100616A>G 100617C>T), L36P (100619T>C), S38N (100625G>A), T40S (100630A>T), H43N (100639C>A), D49E (100659T>A), G50D (100661G>A), G65A (100706G>C), N67D (100711A>G 100713C>T), Q69E (100717C>G), N70T (100721A>C), E72D (100728G>C), T74V (100732A>G 100733C>T), E76N (100738G>A 100740G>C), G78D (100745G>A 100746C>T), D80E (100752T>G), E81T (100753G>A 100754A>C 100755G>T), V84I (100762G>A 100764T>C), T86N (100769C>A 100770G>C), H89Q (100779C>G), S95A (100795A>G 100796G>C 100797C>A), H97R (100802A>G 100803C>A), Q118T (100864C>A 100865A>C 100866G>T), V120L (100870G>C), E130Q (100900G>C 100902G>A), A131D (100904C>A 100905G>C), T133S (100910C>G), D134E (100914C>G), E139D (100929A>T), L143V (100939C>G), S145D (100945T>G 100946C>A 100947G>T), S151A (100963T>G 100965G>A), G154A (100973G>C 100974C>G), H157S (100981C>A 100982A>G 100983C>T), V166I (101008G>A 101010T>C), R168Q (101015G>A), D171K (101023G>A 101025T>A), V174A (101033T>C), E177T (101041G>A 101042A>C), D178S (101044G>A 101045A>G), Q180E (101050C>G), S187D (101071T>G 101072C>A), D228G (101195A>G 101196C>A), Q233D (101209C>G 101211G>C), A234S (101212G>T 101214G>C), R239H (101228G>A), V241T (101233G>A 101234T>C 101235C>G), G242A (101237G>C), G255S (101275G>T 101276G>C), S257G (101281A>G), L287M (101371C>A 101373C>G), Y288K (101374T>A 101376C>G), N292D (101386A>G), T296N (101399C>A), A306P (101428G>C 101430C>G), D308N (101434G>A), P310A (101440C>G 101442C>T), S311D (101443T>G 101444C>A), S314R (101452A>C 101454C>T), Q317H (101463G>C)								
RNA ligase (YP_008059392.1)	16	83	21.2%	398.0	79.3%	68 (100%)	54 (79.4%)	0/0/0/0	0
	K21S (105232A>T 105233A>C 105234G>T), L30M (105259C>A 105261T>G), H39L (105287A>T 105288C>T), E46Q (105307G>C 105309A>G), Y47F (105311A>T 105312T>C), S51D (105322T>G 105323C>A), Q62I (105355C>A 105356A>T 105357G>C), F63Y (105359T>A), V66Q (105367G>C 105368T>A), R67K (105370C>A 105371G>A 105372C>G), N70K (105381C>G), R75E (105394C>G 105395G>A 105396A>G), N78A (105403A>G 105404A>C 105405C>A)								
DNA ligase (YP_008059398.1)	382	455	15.5%	417.0	76.1%	74 (100%)	55 (74.3%)	0/0/0/0	0
	D384E (108995T>G), T387Q (109002A>C 109003C>A 109004G>A), E389D (109010A>C), H398A (109035C>G 109036A>C), I399L (109038A>C 109040T>G), S401T (109045G>C), V405S (109056G>T 109057T>C), V407I (109062G>A), N409K (109070C>G), S415T (109086T>A 109088G>A), Q418M (109095C>A 109096A>T), R420K (109101C>A 109102G>A 109103T>G), K421E (109104A>G), D428E (109127C>G), N431G (109134A>G 109135A>G 109136C>G), C433T (109140T>A 109141G>C), I441V (109164A>G), K446N (109181A>C)								
AdoMet-MTase (YP_008059411.1)	75	305	48.3%	837.0	75.5%	151 (99.3%)	113 (74.3%)	0/1/0/0	0
	V77I (114217G>A 114219T>A), D93N (114265G>A), S94P (114268T>C 114270T>C), E97D (114279G>C), Q100R (114287A>G), A115D (114332C>A 114333A>C), T116A (114334A>G 114336G>A), D129N (114373G>A), L130del (114376_114378delCTA), P131T (114379C>A 114381C>G), Y133E (114385T>G 114387C>G), D134G (114389A>G 114390C>G), G135E (114392G>A), S136V (114394A>G 114395G>T), P138T (114400C>A 114402T>G), D139E (114405T>G), D143R (114415G>C 114416A>G 114417T>C), R146G (114424C>G 114426C>A), S147E (114427T>G 114428C>A), T234S (114688A>T 114690A>G), Q236S (114694C>A 114695A>G 114696G>T), A238S (114700G>T), K240Y (114706A>T 114708G>T), H243E (114715C>G 114717T>A), L245D (114721T>G 114722T>A 114723G>C), K246E (114724A>G), P249I (114733C>A 114734C>T 114735G>C), Y262P (114772T>C 114773A>C 114774C>G), S263W (114776C>G), D265N (114781G>A 114783C>T), T274A (114808A>G 114810G>C), N281D (114829A>G), N282D (114832A>G), R286Q (114845G>A 114846T>G), T288S (114851C>G 114852G>T), S291A (114859A>G 114860G>C), E303K (114895G>A)								
hypothetical protein (YP_008059416.1)	47	251	79.8%	1048.0	67.4%	204 (99.5%)	127 (62.0%)	0/1/0/0	0
	A50D (116275C>A 116276T>C), V51L (116277G>C 116279T>C), E53H (116283G>C 116285G>T), K54A (116286A>G 116287A>C 116288G>A), Y63H (116313T>C 116315C>T), P64S (116316C>T 116318C>G), L66V (116322T>G), A67R (116325G>C 116326C>G 116327A>T), H69N (116331C>A), S73N (116343T>A 116344C>A 116345G>C), N74D (116346A>G 116348T>C), M77V (116355A>G 116357G>T), V79I (116361G>A 116363G>C), D83K (116373G>A 116375T>G), N86H (116382A>C 116384T>C), Y88V (116388T>G 116389A>T 116390C>G), R94Q (116407G>A 116408C>A), F95L (116409T>C 116411C>G), G96del (116412_116414delGGT), G97N (116415G>A 116416G>A), K98Q (116418A>C), R99K (116421C>A 116422G>A 116423C>A), N102G (116430A>G 116431A>G 116432C>T), K103E (116433A>G), V105M (116439G>A), T106G (116442A>G 116443C>G), Q107S (116445C>A 116446A>G 116447G>T), T108Q (116448A>C 116449C>A), K109P (116451A>C 116452A>C 116453G>A), H110T (116454C>A 116455A>C), S112N (116461G>A), I116T (116473T>C), N117I (116476A>T 116477T>C), I123V (116493A>G 116495T>C), Q126D (116502C>G 116504G>T), N128G (116508A>G 116509A>G), V129K (116511G>A 116512T>A 116513C>A), D131E (116519C>A), L133V (116523C>G 116525C>T), T135V (116529A>G 116530C>T 116531G>T), V140L (116544G>T), V145C (116559G>T 116560T>G 116561T>C), E147T (116565G>A 116566A>C), M148I (116570G>T), N158K (116600C>G), L162M (116610C>A 116612C>G), C173T (116643T>A 116644G>C 116645T>A), S175H (116649T>C 116650C>A), K176R (116652A>C 116653A>G 116654G>C), N184K (116678C>G), G188H (116688G>C 116689G>A 116690A>C), K195E (116709A>G), K198R (116718A>C 116719A>G), K200R (116725A>G), R201M (116727C>A 116728G>T 116729C>G), S202C (116730A>T), Q207K (116745C>A 116747A>G), N210H (116754A>C 116756T>C), M212K (116761T>A), E218H (116778G>C 116780G>C), T219S (116782C>G 116783G>C), G220P (116784G>C 116785G>C 116786A>G), M221L (116787A>C), V222S (116790G>T 116791T>C 116792G>C), F224Y (116797T>A 116798T>C), D225E (116801T>G), D228E (116810T>A), M230P (116814A>C 116815T>C), D232T (116820G>A 116821A>C 116822C>G), N234S (116827A>G 116828C>T), A238E (116839C>A), E240D (116846G>C), S241R (116847T>C 116848C>G), I242V (116850A>G 116852C>G), D245N (116859G>A 116861T>C), N246D (116862A>G), K247E (116865A>G)								
hypothetical protein (YP_008059454.1)	14	196	66.5%	798.0	62.2%	182 (97.8%)	111 (59.7%)	3/1/0/0	0

	Begin	End	Coverage	Score	Concordance	Matches	Identities	I/D/M/F*	Stop Codons
	R17E (130379C>T 130380T>C), R18S (130375T>G 130377G>T), N19L (130372G>A 130373T>A 130374T>G), T20I (130369A>T 130370G>A), Y21R (130367T>C 130368A>G), T24P (130359T>G), S25F (130354A>G 130355G>A), P26Y (130351T>A 130352G>T 130353G>A), N27L (130348A>C 130349T>A 130350T>A), K28I (130345C>A 130346T>A), G29V (130342A>T 130343C>A), A31S (130336G>A 130338C>A), Y32del (130333_130335delGTA), I36L (130321T>G 130323T>G), D37A (130318G>T 130319T>G), D38K (130315G>T 130317C>T), S40E (130309C>T 130310G>T 130311A>C), D44K (130297G>T 130299C>T), R46Q (130291A>T 130292C>T), A50E (130279G>T 130280G>T), D51E (130276G>C), E53D (130270T>A), T54S (130267T>A 130268G>C), N55V (130265T>A 130266T>C), R56N (130262C>T 130263G>T), F60H (130250A>T 130251A>G), T67S (130228C>A 130229G>C), S68F (130225C>G 130226G>A), T74A (130207A>G 130209T>C), N75D (130206T>C), T76H (130201C>G 130202G>T 130203T>G), T77V (130198G>C 130199G>A 130200T>C), A78G (130196G>C), M82L (130183C>T 130185T>G), H86T (130172T>G 130173G>T), F91A (130156G>T 130157A>G 130158A>C), G92K (130153T>C 130154C>T 130155C>T), A94Q (130147G>T 130148G>T 130149C>G), S96N (130141C>G 130142G>T 130143A>T), K108W (130105T>C 130106T>C 130107T>A), S110K (130100G>T 130101A>T), A114G (130087A>T 130088G>C), N118R (130075G>A 130076T>C 130077T>G), I119V (130074T>C), P121K (130066G>T 130067G>T 130068G>T), M137L (130018C>G 130020T>G), T138D (130015C>G 130016G>T 130017T>C), M141T (130007A>G), W142F (130003C>G 130004C>A), A143G (130000G>C 130001G>C), E144K (129999C>T), N147L (129989T>A 129990T>G), S148A (129986C>G 129987T>C), L149M (129982G>C 129984G>T), N150V (129979A>T 129980T>A 129981T>C), K155N (129964C>G), P156D (129961C>G 129962G>T 129963G>C), F157V (129958G>T 129960A>C), D158T (129956T>G 129957C>T), S159A (129952A>C 129953C>G 129954T>C), I160L (129949A>T 129951T>G), F162L (129945A>G), S170H (129920C>T 129921T>G), A172G (129913G>A 129914G>C), I175Y (129904A>G 129905A>T 129906T>A), V177C (129898C>A 129899A>C 129900C>A), N178D (129895A>G 129897T>C), L183I (129882A>T), V184L (129877A>G 129879C>G), D185A (129874G>T 129875T>G), D185_V186insQEG (129873_129874insACCCTCTTG), V186I (129871C>G 129873C>T)								
endonuclease III (YP_008059469.1)	88	180	40.8%	407.0	65.8%	93 (100%)	57 (61.3%)	0/0/0/0	0
	K90S (135582C>A 135583T>C), C92V (135577C>A 135578A>C), Y95K (135567G>C 135569A>T), K96N (135564C>A), M103R (135543C>G 135544A>C 135545T>G), D104H (135540A>G 135542C>G), Q107R (135531C>A 135532T>C), S108N (135528A>G 135529G>T 135530A>T), R110H (135523C>T), E111L (135519C>T 135520T>A 135521C>A), E113A (135513C>G 135514T>G), K114D (135510C>G 135512T>C), N117D (135503T>C), L118M (135498G>C 135500G>T), M120V (135492C>G 135494T>C), S121E (135489C>T 135490G>T 135491A>C), D122G (135487T>C), E123D (135483T>G), L124T (135480T>C 135481A>G 135482A>T), K126T (135474C>G 135475T>G), K129R (135465C>A 135466T>C 135467T>G), V131I (135459G>A 135461C>T), L132I (135456A>G 135458G>T), V146T (135414G>A 135415A>G 135416C>T), E154D (135390T>A), M156A (135385A>G 135386T>C), T160A (135372A>C 135374T>C), M163Q (135364A>T 135365T>G), Q164N (135360C>G 135362G>T), T166L (135354T>A 135355G>A 135356T>G), G167D (135352C>T), T168V (135348C>T 135349G>A 135350T>C), R170A (135343C>G 135344G>C), V174I (135330C>T 135332C>T), E177D (135321T>G)								
hypothetical protein (YP_008059471.1)	105	143	25.0%	276.0	80.2%	39 (100%)	28 (71.8%)	0/0/0/0	0
	K107Q (136716T>G), A109N (136709G>T 136710C>T), N110K (136705G>C), R111A (136703C>G 136704G>C), A113K (136697G>T 136698C>T), T114M (136694G>A), T116E (136688G>T 136689T>C), D117G (136684G>T 136685T>C), K124E (136665T>C), A137S (136624C>A 136625G>C 136626C>T)								
hypothetical protein (YP_008059475.1)	66	146	55.5%	384.0	74.9%	81 (100%)	60 (74.1%)	0/0/0/0	1
	D68E (138684G>T), A72E (138672G>T 138673G>T), E73T (138669T>A 138670T>G 138671C>T), L74I (138668G>T), E76G (138660C>T 138661T>C), L97A (138597G>C 138598A>G 138599G>C), E101A (138585T>C 138586T>G), T102V (138582C>T 138583G>A 138584T>C), S103T (138579A>G 138581A>T), V105I (138573C>G 138575C>T), A110S (138558C>T 138560C>A), N114D (138546G>A 138548T>C), F115Y (138544A>T), H116T (138541T>G 138542G>T), D117Q (138537G>T 138539C>G), S120D (138529C>T 138530T>C), K128S (138504T>G 138505T>C), E129D (138501C>G), D132E (138492G>T), E138K (138474T>C 138476C>T), Q139H (138471C>G)								

*: Inserts / Deletes / Misaligned / Frameshifts

Detailed Results of Halovirus VNH-1 (NC_025213.1)

NGS details

Assembly

**Coverage length**	395 (2 contig(s))
**Est. depth of coverage**	21.4
**Est. number of reads**	101
**Ambiguities**	0

??coverage-details?? ??variant-analysis??

Assignment

**Type**	Halovirus VNH-1 (Taxonomy ID: 1500510)
**Reference Genome**	NC_025213.1 (Length: 10021bp)
**Host(s)**	Nanohaloarchaea archaeon AB578-D14
**NT Identity (%)**	65.051
**NT Quality**	0.567089

Alignment

**Alignment score**	224 (NT) + 657 (AA) = 881
**Concordance (%)**	51.9151
**Alignment method**	Global, seeded, nucleotide + amino acids (AGA)

Genome Region

Sequence starts at position null and ends at position null relative to the null reference sequence for null.

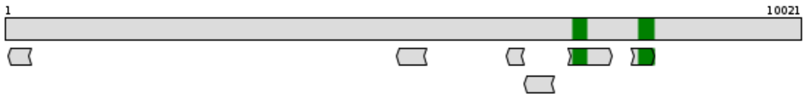

Alignment Detailed Statistics

	Begin	End	Coverage	Score	Concordance	Matches	Identities	I/D/M/F*	Stop Codons
**NT**	7147	8173	3.9%	224.0	29.0%	392 (99.2%)	255 (64.6%)	0/3	
**CDS**									
MZ08_gp5	17	79	34.2%	312.0	66.4%	62 (98.4%)	41 (65.1%)	0/1/0/0	0
MZ08_gp6	30	98	70.4%	345.0	72.2%	69 (100%)	50 (72.5%)	0/0/0/0	0
**Proteins**									
hypothetical protein (YP_009072429.1)	17	79	34.2%	312.0	66.4%	62 (98.4%)	41 (65.1%)	0/1/0/0	0

	Begin	End	Coverage	Score	Concordance	Matches	Identities	I/D/M/F*	Stop Codons
	E22Q (7162G>C), C23Y (7166G>A), M24L (7168A>C 7170G>C), D29E (7185T>G), E34R (7198G>C 7199A>G 7200A>C), P37D (7207C>G 7208C>A), F40Y (7217T>A 7218T>C), G44S (7228G>T 7229G>C 7230T>C), N46S (7235A>G 7236T>C), C47G (7237T>G 7239T>G), N48del (7240_7242delAAC), G49S (7243G>T 7244G>C), E50S (7246G>T 7247A>C 7248G>C), T55A (7261A>G), T57Q (7267A>C 7268C>A 7269C>G), E58G (7271A>G 7272A>T), S59A (7273T>G 7275A>C), K63P (7285A>C 7286A>C 7287G>C), K66R (7294A>C 7295A>G 7296A>G), E71D (7311A>C), I77V (7327A>G)								
hypothetical protein (YP_009072430.1)	30	98	70.4%	345.0	72.2%	69 (100%)	50 (72.5%)	0/0/0/0	0
	V34I (7980G>A 7982G>T), E36D (7988G>C), R37D (7989C>G 7990G>A), D39T (7995G>A 7996A>C 7997T>C), T41Q (8001A>C 8002C>A 8003C>A), I44R (8010A>C 8011T>G 8012T>G), R60S (8058C>T 8059G>C), S64A (8070T>G 8072A>C), Q66R (8077A>G), T71S (8091A>T 8093T>A), F72H (8094T>C 8095T>A 8096T>C), I76V (8106A>G), E80A (8119A>C), H82E (8124C>G 8126T>G), D83R (8127G>C 8128A>G 8129T>G), K86R (8136A>C 8137A>G 8138A>C), M87R (8140T>G), L88Q (8142T>C 8143T>A)								

*: Inserts / Deletes / Misaligned / Frameshifts

Detailed Results of Halorubrum phage CGphi46 (NC_021537.1)

NGS details

Assembly

**Coverage length**	10297 (19 contig(s))
**Est. depth of coverage**	56.9
**Est. number of reads**	7031
**Ambiguities**	0

??coverage-details?? ??variant-analysis??

Assignment

**Type**	Halorubrum phage CGphi46 (Taxonomy ID: 754066)
**Reference Genome**	NC_021537.1 (Length: 39784bp)
**Host(s)**	Halorubrum sp.
**NT Identity (%)**	73.7991
**NT Quality**	0.916675

Alignment

**Alignment score**	9439 (NT) + 16277 (AA) = 25716
**Concordance (%)**	60.4897
**Alignment method**	Local, heuristic, nucleotide (BLASTN)

Genome Region

Sequence starts at position null and ends at position null relative to the null reference sequence for null.

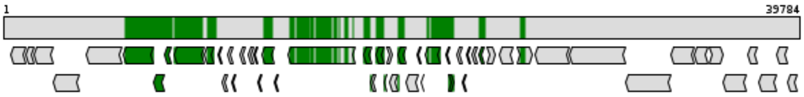

Alignment Detailed Statistics

	Begin	End	Coverage	Score	Concordance	Matches	Identities	I/D/M/F*	Stop Codons
**NT**	6058	26049	25.9%	9439.0	46.8%	10214 (98.7%)	7574 (73.2%)	49/83	
**CDS**									
HALG_00007	1	489	97.0%	2859.0	84.2%	487 (99.2%)	410 (83.5%)	2/2/1/1	0
HALG_00008	1	184	100%	684.0	58.9%	177 (95.2%)	105 (56.5%)	2/7/0/0	1
HALG_00009	1	106	100%	312.0	44.2%	106 (99.1%)	55 (51.4%)	1/0/1/1	1
HALG_00010	23	489	94.5%	2409.0	72.5%	465 (99.6%)	320 (68.5%)	0/2/0/0	0

	Begin	End	Coverage	Score	Concordance	Matches	Identities	I/D/M/F*	Stop Codons
HALG_00011	1	121	72.9%	569.0	64.7%	121 (100%)	76 (62.8%)	0/0/0/0	0
HALG_00020	29	192	79.6%	1039.0	85.8%	164 (100%)	139 (84.8%)	0/0/0/0	0
HALG_00022	41	1103	73.0%	3956.0	69.7%	816 (98.9%)	532 (64.5%)	4/5/0/0	0
HALG_00023	2	119	91.5%	573.0	73.8%	112 (94.9%)	77 (65.3%)	0/6/0/0	0
HALG_00025	1	134	95.0%	675.0	69.5%	134 (100%)	91 (67.9%)	0/0/0/0	0
HALG_00026	45	52	15.4%	30.0	100%	7 (87.5%)	7 (87.5%)	0/1/0/0	1
HALG_00028	1	3	2.2%	4.0	15.4%	3 (100%)	1 (33.3%)	0/0/0/0	0
HALG_00029	21	131	84.7%	508.0	67.3%	111 (99.1%)	75 (67.0%)	1/0/0/0	0
HALG_00033	1	228	83.9%	1049.0	78.5%	209 (100%)	159 (76.1%)	0/0/0/0	0
HALG_00034	1	63	100%	178.0	45.3%	62 (96.9%)	31 (48.4%)	1/1/0/0	1
HALG_00035	1	87	88.8%	356.0	55.8%	87 (96.7%)	49 (54.4%)	3/0/0/0	1
HALG_00040	1	90	100%	518.0	84.9%	90 (100%)	78 (86.7%)	0/0/0/0	1
HALG_00043	30	119	35.6%	558.0	80.8%	89 (98.9%)	73 (81.1%)	0/1/0/0	0
**Proteins**									
hypothetical protein (YP_008126542.1)	1	489	97.0%	2859.0	84.2%	487 (99.2%)	410 (83.5%)	2/2/1/1	0
	V4del (7513_7515delGAC), P5T (7510G>C 7512G>T), D6S (7507G>C 7508T>G 7509C>A), A9S (7498T>C 7500C>A), G10del (7495_7497delTCC), A11V (7492G>C 7493G>A), P13E (7486T>C 7487G>T 7488G>C), E15D (7480C>G), D19K (7468G>T 7470C>T), E21A (7462C>G 7463T>G), L34I (7425G>T), Q35R (7421T>C), K41E (7404T>C), N49E (7378G>T 7380T>C), Q51E (7374G>C), V55I (7360C>G 7362C>T), F65Y (7330A>G 7331A>T), S100A (7227A>C), E104D (7213C>G), E111R (7192C>G 7193T>C 7194C>G), T112K (7189C>T 7190G>T), S126A (7149A>C), E152D (7069C>G), E164D (7033C>G), D182N (6979A>G 6981C>T), D201E (6922G>C), D206P (6907A>C 6908T>G 6909C>G), R207K (6905C>T 6906G>T), E228T (6842T>G 6843C>T), S230G (6835G>C 6837T>C), L239K (6808G>C 6809A>T 6810G>T), K243V (6796T>G 6797T>A 6798T>C), D258E (6751G>C), E259D (6748T>G), R261K (6742T>C 6743C>T 6744G>T), K265E (6730C>T 6732T>C), A271S (6712A>C 6714C>A), V272K (6709G>T 6710A>T 6711C>T), E289S (6659T>G 6660C>A), W293F (6646C>G 6647C>A), K299E (6630T>C), A300S (6625G>A 6626G>C 6627C>T), R304Q (6614C>T), V306A (6608A>G), P308A (6603G>C), E309D (6598C>G), M310T (6596A>G), A311T (6594C>T), P312_D313insTX (6588_6589insTGCGT), A316L (6578G>A 6579C>G), G333A (6527C>G), L334N (6523C>G 6524A>T 6525G>T), E337D (6514C>G), D338T (6511G>C 6512T>G 6513C>T), R341T (6503C>G 6504G>T), A355T (6460C>G 6462C>T), S358C (6451C>A 6452G>C), A360L (6446G>A 6447C>G), I361L (6444T>G), E362D (6439C>G), Q363R (6437T>C), T367A (6426T>C), I370V (6417T>C), A386S (6367G>C 6369C>A), V393I (6346C>G 6348C>T), Q399E (6330G>C), Q403K (6318G>T), E404K (6315C>T), D416E (6277G>T), G423S (6257C>G 6258C>A), E424D (6253C>A), E428D (6241C>G), N429H (6240T>G), T446K (6187G>C 6188G>T), E466D (6127C>G), H467F (6125T>A 6126G>A), G481S (6083C>G 6084C>A), A482S (6081C>A), V484A (6073A>C 6074A>G), A485V (6070C>G 6071G>A)								
hypothetical protein (YP_008126543.1)	1	184	100%	684.0	58.9%	177 (95.2%)	105 (56.5%)	2/7/0/0	1
	D3N (8066C>T), P4_D5del (8058_8063delATCGGG), I7L (8054T>G), R8T (8050C>G 8051G>T), D9N (8048C>T), T11M (8040T>C 8041G>A), E15P (8029T>G 8030C>G), R16Q (8025G>C 8026C>T), Q20L (8014T>A 8015G>A), D23E (8004G>C), F26Y (7995A>G 7996A>T), A30D (7983C>G 7984G>T), D31E (7980G>T), A34E (7972G>T), D36P (7965G>C 7966T>G 7967C>G), L37T (7963A>G 7964G>T), D39E (7956G>C), T41A (7950A>G 7952T>C), A42E (7948G>T), R45G (7938A>C 7940G>C), I46L (7937T>G), S49K (7927G>T 7928A>T), V50I (7925C>T), A51V (7920C>G 7921G>A), E52S (7918T>G 7919C>A), G53A (7915C>G), Q55K (7910G>T), N56S (7905G>C 7906T>G 7907T>A), P59V (7897G>A 7898G>C), R60V (7893C>G 7894C>A 7895G>C), Q61S (7890C>G 7891T>G 7892G>A), A63P (7884C>G 7886C>G), A64D (7881C>G 7882G>T), T65P (7880T>G), N66_E70del (7863_7877delCTCCACCTTCTTGTT), E74D (7851C>G), D77E (7842G>C), E78A (7840T>G), K81E (7830T>C 7832T>C), H82G (7828T>C 7829G>C), A84R (7821T>G 7822G>C 7823C>G), Q85V (7819T>A 7820G>C), P86A (7817G>C), K90T (7804T>G), V92I (7799C>T), V96A (7786A>G), A99T (7778C>T), E101P (7770T>C 7771T>G 7772C>G), Y104R (7761A>C 7762T>C 7763A>G), E111P (7740C>G 7741T>G 7742C>G), G112E (7738C>T), S115R (7728T>C 7729G>C 7730A>G), K119Q (7718T>G), V123A (7704A>C 7705A>G), R127E (7692G>C 7693C>T 7694G>C), E130R (7683C>G 7684T>C 7685C>G), G132A (7677G>C 7678C>G), D153S (7614G>C 7615T>G 7616C>A), T155N (7608C>G 7609G>T), E157Q (7602T>C 7604C>G), R159S (7597C>G 7598G>A), H160L (7594T>A), K161D (7590C>G 7592T>C), I162V (7589T>C), A164M (7581G>C 7582G>A 7583C>T), V166A (7575G>C 7576A>G), E167T (7573T>G 7574C>T), H168_S169insDV (7568_7569insCACGTC), G170A (7564C>G), I172L (7557A>G 7559T>G), G173A (7554G>C 7555C>G), D176K (7545G>C 7547C>T), A180D (7533T>G 7534G>T), A181E (7530G>T 7531G>T), Q182D (7527T>G 7529G>C)								
hypothetical protein (YP_008126544.1)	1	106	100%	312.0	44.2%	106 (99.1%)	55 (51.4%)	1/0/1/1	1
	T2S (8378G>C), T4A (8371C>T 8373T>C), H6R (8366T>C), R8T (8359C>G 8360C>G 8361G>T), T9A (8358T>C), E10R (8354T>C 8355C>G), A11T (8352C>T), R17L (8333C>A), D18G (8330T>C), K19S (8327T>G 8328T>A), A20_V21insX (8322_8323insT), E22D (8317C>G), H24Y (8313G>A), K25R (8308C>G 8309T>C 8310T>G), Q26E (8305T>C 8307G>C), A27T (8304C>T), I28L (8299G>C 8301T>G), R29A (8297C>G 8298G>C), S30A (8294C>G 8295T>C), D35L (8278G>C 8279T>A 8280C>G), P36S (8275G>T 8277G>A), T37M (8273G>A), M38I (8269C>A), K41T (8261T>G), T43K (8254G>C 8255G>T), L45A (8249A>G 8250G>C), I48L (8241T>G), I49L (8238T>G), L52I (8229G>T), G56A (8216C>G), L57I (8214G>T), S58G (8209C>G 8210G>C 8211A>C), L62I (8197C>G 8199G>T), M63A (8194C>T 8195A>G 8196T>C), L64A (8191G>C 8192A>G 8193G>C), V68P (8179G>C 8180A>G 8181C>G), W70L (8173C>G 8174C>A 8175A>G), T72A (8167C>G 8169T>C), I73T (8164G>C 8165A>G), F74L (8163A>G), G75A (8159C>G), A80G (8144G>C), I85L (8130T>G), M87L (8122C>G 8124T>G), E89D (8116C>G), L90Y (8113C>G 8114A>T 8115G>A), F93L (8106A>G), F94V (8103A>C), Q98K (8091G>T), E101Q (8080T>C 8082C>G), K102N (8077C>G), G103S (8075C>G 8076C>A)								
hypothetical protein (YP_008126545.1)	23	489	94.5%	2409.0	72.5%	465 (99.6%)	320 (68.5%)	0/2/0/0	0

	Begin	End	Coverage	Score	Concordance	Matches	Identities	I/D/M/F*	Stop Codons
	P24S (9962G>A), D28E (9948G>T), D31G (9940T>C), A34S (9932C>A), V37I (9921C>G 9923C>T), N42R (9906G>C 9907T>C 9908T>G), A43S (9905C>A), L44I (9900G>A 9902G>T), K45Q (9899T>G), K47A (9891C>G 9892T>G 9893T>C), F53Y (9874A>T), R55H (9868C>T), E56A (9865T>G), S57A (9863A>C), Y61F (9850T>A), V66E (9834A>T 9835A>T), E67D (9831C>G), N73D (9815T>C), T74D (9811G>T 9812T>C), D75G (9807A>G 9808T>C), D76S (9805T>G 9806C>A), D77G (9802T>C), A86T (9774G>C 9776C>T), E87del (9771_9773delCTC), A88P (9770C>G), L90I (9762C>G 9764G>T), P91del (9759_9761delCGG), D95A (9748T>G), E97D (9741C>G), P98V (9739G>A 9740G>C), G101E (9729G>C 9730C>T), A102G (9727G>C), D108E (9708A>T), A111E (9699G>T 9700G>T), Y115F (9687A>G 9688T>A), L116I (9684C>G 9686G>T), R118K (9678G>T 9679C>T 9680G>T), E119Q (9677C>G), A124T (9660G>T 9662C>T), V126L (9654G>C 9656C>G), G128E (9648C>T 9649C>T), A130T (9644C>T), D131E (9639G>T), L133M (9635G>T), D134G (9630G>C 9631T>C), P137K (9622G>T 9623G>T), I139V (9615G>A 9617T>C), E142Q (9606T>C 9608C>G), R145K (9597G>C 9598C>T 9599G>T), R148K (9588G>T 9589C>T 9590G>T), R149Q (9585C>T 9586C>T), R150Q (9582G>C 9583C>T), V156I (9564G>T 9566C>T), S159T (9556C>G), A160S (9552C>A 9553G>C 9554C>T), G161E (9549G>C 9550C>T), H162T (9546G>T 9547T>G 9548G>T), V163I (9545C>T), A164G (9540G>A 9541G>C), D168E (9528G>C), A170T (9522C>T 9524C>T), Q186R (9475T>C), R190L (9462C>T 9463C>A 9464G>A), S191R (9459C>T 9460G>C 9461A>G), A194K (9451G>T 9452C>T), Q198A (9438C>G 9439T>G 9440G>C), A201D (9429C>A 9430G>T), E206T (9414C>T 9415T>G 9416C>T), T208E (9408C>T 9409G>T 9410T>C), S210D (9402C>G 9403G>T 9404A>C), S212D (9396C>A 9397G>T 9398A>C), D213S (9393G>C 9394T>G 9395C>A), S215N (9387C>A 9388G>T 9389A>T), I230E (9342G>T 9343A>T 9344T>C), R233D (9333C>G 9334C>T 9335G>C), A234E (9330C>T 9331G>T), E237N (9321C>A 9323C>T), V238I (9318G>T 9320C>T), V239Q (9315G>C 9316A>T 9317C>G), S241C (9309A>G 9310G>C), D243E (9303G>T), E244N (9300C>G 9302C>T), V249I (9285G>A 9287C>T), A252V (9277G>A), E254R (9271T>C 9272C>G), S258N (9258C>A 9259G>T 9260A>T), A260K (9252C>T 9253G>T 9254C>T), A264G (9241G>C), D266S (9234G>C 9235T>G 9236C>A), V279I (9195C>G 9197C>T), E286S (9175T>G 9176C>A), W289F (9165C>A 9166C>A), F295W (9147G>C 9148A>C), E301D (9129C>G), V302A (9126C>G 9127A>G), R309K (9106C>T 9107G>T), V311L (9099G>C 9101C>G), A315S (9087C>G 9088G>C 9089C>T), R317E (9081G>C 9082C>T 9083G>C), P319E (9076G>T 9077G>C), Q322N (9066C>G 9068G>T), Q332K (9038G>T), N333H (9033G>A 9035T>G), K340R (9013T>C 9014T>G), T351S (8981T>A), T355A (8969T>C), I356V (8966T>C), S363F (8943C>A 8944G>A), D364G (8940G>C 8941T>C), A365E (8938G>T), D367E (8931G>T), V372Q (8917A>T 8918C>G), A378P (8898C>G 8900C>G), F379Y (8896A>T), R380K (8893C>T 8894G>T), E383Q (8883C>T 8885C>G), M384L (8880C>T 8882T>G), E388K (8868C>T 8870C>T), T390K (8862G>T 8863G>T), D409E (8805G>T), T413V (8794G>A 8795T>C), D418E (8778G>T), E420D (8772C>G), V423I (8763G>T 8765C>T), Y425A (8757G>C 8758T>G 8759A>C), S438E (8718G>T 8719C>T 8720T>C), L443I (8703G>A 8705G>T), A444C (8700C>A 8701G>C 8702C>A), P446A (8694C>T 8696G>C), A451P (8679G>C 8681C>G), G452D (8676G>A 8677C>T), S457Q (8661G>T 8662C>T 8663T>G), G459S (8655G>A 8657C>T), A460G (8653G>C), M462V (8646C>T 8648T>C), P463E (8643G>T 8644G>T 8645G>C), G464Q (8640G>T 8641C>T 8642C>G), D465Q (8637G>C 8639C>G), H466Y (8634G>A 8636G>A), Q467R (8631C>T 8632T>C), G468D (8628G>A 8629C>T), V469I (8625G>T 8627C>T), H473F (8613G>A 8614T>A 8615G>A), D478Y (8598G>A 8600C>A), L480I (8592C>A 8594G>T), G482C (8586G>A 8588C>A)								
hypothetical protein (YP_008126546.1)	1	121	72.9%	569.0	64.7%	121 (100%)	76 (62.8%)	0/0/0/0	0
	V1L (10527C>T 10529C>G), A2P (10526C>G), H3F (10522T>A 10523G>A), I5N (10515A>G 10516A>T), L6S (10512G>T 10513A>G 10514G>A), T7N (10510G>T), S8N (10507C>T), T9L (10503A>G 10504G>A 10505T>G), N11S (10498T>C), G12E (10494G>T 10495C>T), P13A (10491T>A 10493G>C), A15Q (10485G>T 10486G>T 10487C>G), R17H (10480C>T), L18I (10476C>G 10478G>T), D20N (10472C>T), P22S (10464C>G 10465G>C 10466G>T), V24T (10459A>G 10460C>T), K26S (10453T>G 10454T>A), I31V (10439T>C), I34V (10428G>A 10430T>C), A40T (10410A>C 10412C>T), G42D (10405C>T), V44M (10398G>C 10400C>T), T45V (10395C>T 10396G>A 10397T>C), F48Y (10387A>T), Y52F (10375T>A), D55H (10365G>A 10367C>G), T57R (10360G>C 10361T>G), T58N (10356C>G 10357G>T), G70S (10320G>C 10321C>G 10322C>A), E71N (10317C>G 10319C>T), Q72V (10314C>G 10315T>A 10316G>C), A78D (10297G>T), E79R (10293C>G 10294T>C 10295C>G), D92S (10255T>C 10256C>T), D93G (10251G>C 10252T>C), G97D (10240C>T), E98A (10237T>G), R101E (10227G>C 10228C>T 10229G>C), Q105K (10215C>T 10217G>T), H106Q (10212G>C), I108Y (10207A>T 10208T>A), E112K (10196C>T), A113H (10191C>G 10192G>T 10193C>G)								
hypothetical protein (YP_008126555.1)	29	192	79.6%	1039.0	85.8%	164 (100%)	139 (84.8%)	0/0/0/0	0
	D32A (13457G>C 13458T>G), C41A (13430G>C 13431C>G 13432A>C), H45R (13418G>T 13419T>C), A47T (13414C>T), R48Q (13409G>C 13410C>T), T56E (13385C>T 13386G>T 13387T>C), E69R (13346C>T 13347T>C 13348C>G), Q78K (13321G>T), A80S (13313C>G 13315C>A), A108I (13229C>G 13230G>A 13231C>T), V110I (13225C>T), M114Q (13212A>T 13213T>G), E123A (13185T>G), A141E (13131G>T), D143G (13125T>C), D144E (13121G>C), I146V (13117T>C), S152E (13097G>C 13098C>T 13099T>C), Y158G (13080T>C 13081A>C), E162D (13067C>G), T164S (13063T>A), E168A (13049C>G 13050T>G), A183V (13005G>A), A184D (13001C>G 13002G>T)								
hypothetical protein (YP_008126557.1)	41	1103	73.0%	3956.0	69.7%	816 (98.9%)	532 (64.5%)	4/5/0/0	0

	Begin	End	Coverage	Score	Concordance	Matches	Identities	I/D/M/F*	Stop Codons
	E46D (17468C>G), T47D (17465C>G 17466G>T 17467T>C), L49H (17460A>T), V127I (17227C>T), A128V (17223G>A), V132M (17212C>T), T142S (17181G>C), D145E (17171G>C), S157C (17135C>G 17136G>C), V163Q (17117G>C 17118A>T 17119C>G), G169D (17100C>T), D170E (17096G>C), V172A (17091A>G), P176D (17078C>G 17079G>T 17080G>C), S263R (16817C>G 16818G>C 16819A>G), S273D (16788C>T 16789T>C), L296I (16720G>T), E302Q (16700C>T 16702C>G), G318R (16652C>G 16654C>G), S319D (16649C>G 16650G>T 16651A>C), V320N (16647A>T 16648C>T), D321V (16644T>A), T322P (16642T>G), S324K (16634G>C 16635C>T), T326N (16628C>G 16629G>T), R329T (16620C>G 16621G>T), V330L (16616C>A 16618C>G), G331S (16613G>A 16615C>T), G332S (16610G>C 16611C>G 16612C>A), A338G (16592C>T 16593G>C), G339D (16590C>T), E340K (16586C>T 16588C>T), T341N (16583G>A 16584G>T), G342A (16580G>A 16581C>G), E343V (16578T>A), S346P (16570A>G), S347C (16567T>A), T348S (16562G>A 16564T>A), A349T (16561C>T), S350A (16556G>T 16557C>G 16558T>C), G351A (16554C>G), D352E (16550G>T), I353A (16548A>G 16549T>C), V354F (16546C>A), S355A (16541G>C 16543A>C), V356H (16538C>G 16539A>T 16540C>G), A358D (16532C>A 16533G>T), G361P (16523G>C 16524C>G 16525C>G), H362E (16520G>T 16522G>C), R363S (16517C>A 16519G>T), F369W (16499G>C 16500A>C), S402D (16400C>G 16401G>T 16402A>C), A403M (16398G>A 16399C>T), A404T (16394C>T 16396C>T), S409T (16380C>G), D412E (16370G>C), E415D (16361C>G), I416M (16358G>C), I418V (16352A>C 16354T>C), T426A (16328C>G 16330T>C), Y441F (16284T>A), P445Q (16271C>T 16272G>T), A446S (16268C>G 16269G>C 16270C>T), E450D (16256C>G), R452K (16250C>T 16251C>T 16252G>T), K453E (16249T>C), L454F (16246G>A), G456A (16238A>G 16239C>G), G457D (16235C>G 16236C>T), V462T (16220C>T 16221A>G 16222C>T), R463L (16217C>G 16218C>A), K464D (16214T>G 16216T>C), T466S (16208G>C 16210T>A), H467K (16205G>C 16207G>T), M469I (16199C>G), H472_P473insTT (16189_16190insCGTCGT), P473A (16189G>C), D474A (16185T>G), E475G (16181C>T 16182T>C), Q476P (16178C>G 16179T>G), A480T (16168C>T), D482E (16160G>C), V484S (16155A>G 16156C>A), T486G (16149G>C 16150T>C), V487P (16145G>C 16146A>G 16147C>G), G488D (16143C>T), T491L (16134G>A 16135T>G), E495A (16122T>G), M496V (16120T>C), S497L (16115C>G 16116G>A 16117A>G), I499T (16110A>G), E500D (16106C>G), R501E (16104C>T 16105G>C), R501_E502insSS (16102_16103insTGAACT), E502Q (16100C>T 16102C>G), I503L (16099T>G), A504I (16094G>T 16095G>A 16096C>T), A505P (16091C>T 16093C>G), E506L (16088C>G 16089T>A 16090C>G), R507W (16085G>C 16087G>A), A508Q (16082C>T 16083G>T 16084C>G), E510D (16076C>G), I511D (16073T>G 16074A>T 16075T>C), S512G (16071G>C 16072A>C), P513R (16067C>G 16068G>C), E514D (16064C>G), T515del (16061_16063delCGT), H516P (16059T>G), A518D (16052C>G 16053G>T), Y519V (16050T>A 16051A>C), S521R (16045T>G), E522D (16040C>G), V524I (16036C>T), Q533E (16009G>C), T536D (15998C>G 15999G>T 16000T>C), S537A (15996C>G 15997T>C), L547F (15967G>A), Q558D (15932C>G 15934G>C), E560S (15926C>G 15927T>G 15928C>A), Q562E (15922G>C), T565V (15912G>A 15913T>C), H567N (15907G>T), T569L (15899C>G 15900G>A 15901T>G), E571D (15893C>G), S577T (15877A>T), S578A (15872G>C 15873C>G 15874T>C), S581E (15863G>C 15864G>T 15865A>C), V582T (15860C>T 15861A>G 15862C>T), A583L (15857C>G 15858G>A 15859C>G), Q585R (15852T>C), L587F (15847G>A), N590D (15838T>C), D592E (15830G>C), P593L (15827C>A 15828G>A), M594L (15824C>G 15826T>G), A595T (15823C>T), C596A (15818A>G 15819C>G 15820A>C), D597E (15815G>T), D598S (15812G>C 15813T>G 15814C>A), K600P (15807T>G 15808T>G), A602T (15802C>T), L603M (15799G>T), T604L (15795G>A 15796T>A), M607L (15785C>G 15787T>G), T610S (15778T>A), I613L (15767G>T 15769T>G), M615V (15763T>C), W616I (15758C>A 15759C>A 15760A>T), G623A (15737G>C 15738C>G), K626N (15728C>G), W628R (15722C>G 15724A>G), E629R (15719T>C 15720T>C 15721C>G), L630K (15716A>T 15717A>T 15718G>T), R632K (15711C>T 15712G>T), F633L (15707G>A 15709A>G), Q634H (15704C>G), T635R (15701T>G 15702G>C 15703T>G), L636Y (15698G>A 15699A>T 15700G>A), M637L (15695C>A 15697T>G), K639T (15690T>G), R642K (15680A>T 15681C>T 15682G>T), V645I (15673C>T), T647S (15667T>A), R648K (15663C>T 15664G>T), K653R (15648T>C 15649T>G), S654T (15644C>G 15646A>T), E656D (15638T>G), E657N (15635C>G 15637C>T), E659H (15629T>G 15631C>G), G661H (15624C>T 15625C>G), L664V (15614G>C 15616G>C), M665C (15611C>A 15612A>C 15613T>A), I666L (15610T>G), S667A (15607A>C), A671P (15595C>G), T693S (15527G>C 15529T>A), E703K (15499C>T), E710D (15476C>A), A712G (15471G>C), D713G (15467G>C 15468T>C), G714E (15464G>T 15465C>T), E719D (15449C>G), I728L (15424T>G), E736Q (15398T>C 15400C>G), R737Q (15395A>C 15396C>T), A739E (15389G>T 15390G>T), E740D (15386C>G), Q741R (15384T>C 15385G>T), F742L (15382A>G), R744T (15374T>G 15375C>G 15376G>T), L745Q (15371C>T 15372A>T), R747S (15366C>G 15367G>A), Q748R (15363T>C), E751D (15353C>G), I753V (15349T>C), D755E (15341A>T), L773I (15289G>T), V776I (15278G>A 15280C>T), L780M (15266G>C 15268G>T), M781S (15264A>G 15265T>A), R784S (15254C>A 15255C>G 15256G>A), L785R (15251C>T 15252A>C), D788T (15242G>T 15243T>G 15244C>T), T789S (15239C>A 15241T>A), R794E (15224G>C 15225C>T 15226G>C), T795L (15221C>T 15222G>A 15223T>A), A797T (15217C>T), P798T (15214G>T), G799D (15210C>T), D800E (15206G>C), V801I (15205C>T), D803A (15197G>C 15198T>G), T817S (15157T>A), L826V (15128G>A 15130G>C), L838I (15094G>T), A840E (15086G>T 15087G>T), E841D (15083C>G), Q842E (15080C>T 15082G>C), R849E (15059G>T 15060C>T 15061G>C), Q855E (15043G>C), V856I (15040C>T), V863T (15018A>G 15019C>T), G875A (14981G>A 14982C>G), M881T (14964A>G), S884T (14955C>G), T886A (14950T>C), R893Q (14927G>T 14928C>T), D897E (14915A>C), P899G (14910G>C 14911G>C), E900G (14907T>C), A904N (14895G>T 14896C>T), S905T (14891C>G 14893A>T), K908Q (14884T>G), L909Y (14879C>A 14880A>T 14881G>A), S910T (14878A>T), R911T (14874C>G 14875G>T), P912G (14870C>T 14871G>C 14872G>C), T921S (14844G>C), T922A (14842T>C), D923A (14837G>C 14838T>G), V925T (14832A>G 14833C>T), S926D (14828C>G 14829G>T 14830A>C), D929T (14820T>G 14821C>T), I930F (14816G>A 14818T>A), A932P (14810G>C 14812C>G), G940D (14787C>T), S941A (14784C>G 14785T>C), E943D (14777C>G), S944_S946del (14768_14776delAGAGTCGGA), D948E (14762A>C), G950S (14756G>T 14757C>G 14758C>A), A951D (14753C>G 14754G>T), S953T (14749A>T), R954del (14744_14746delCCT), S955T (14741G>C 14742C>G), S957T (14735T>G 14737A>T), A958Q (14732C>T 14733G>T 14734C>G), A960P (14726T>C 14728C>G), I966V (14710T>C), D975E (14681G>T), V1007A (14585C>G 14586A>G), S1008D (14583C>T 14584T>C), V1020I (14548C>T), N1025D (14533T>C), M1027V (14527T>C), Q1029R (14520T>C), V1030L (14518C>G), T1046D (14469G>T 14470T>C), T1049A (14461T>C), N1050S (14457T>C), V1052A (14451A>G), T1061A (14423A>G 14425T>C), A1064V (14415G>A), L1066V (14410G>C), Q1069R (14400T>C), A1073V (14388G>A), D1077N (14377C>T), A1087P (14345A>G 14347C>G), V1089I (14341C>T), I1093V (14329T>C), G1094D (14325C>T), D1098G (14313T>C), T1099S (14309G>C 14311T>A)								
hypothetical protein (YP_008126558.1)	2	119	91.5%	573.0	73.8%	112 (94.9%)	77 (65.3%)	0/6/0/0	0
	T6A (18344G>C 18346T>C), Q8H (18338C>G), D14E (18320A>C), A15T (18317G>C 18319C>T), R16Y (18315C>T 18316G>A), D17E (18311A>T), I18V (18308T>C 18310T>C), D21E (18299A>C), N23D (18293G>A 18295T>C), R24A (18290T>G 18291C>G 18292T>C), F25Y (18288A>T), L35V (18259G>C), A42S (18236G>C 18238C>A), D43H (18233G>A 18235C>G), N45D (18227A>G 18229T>C), V47I (18223C>T), V48I (18220C>T), R54T (18200C>G 18201C>G 18202G>T), T61A (18181T>C), A63P (18173A>C 18175C>G), R64S (18172G>T), F82I (18116G>T 18118A>T), S83_S86del (18104_18115delTGAGTCGGCCGA), M88I (18098C>T), S90N (18093G>T 18094A>T), E91del (18089_18091delCTC), T92S (18088T>A), A93S (18085C>A), T95A (18077C>G 18079T>C), Y96F (18075T>A), A97S (18073C>A), E105D (18047C>G), N109G (18035G>T 18036T>C 18037T>C), P110A (18032C>G 18034G>C), R111del (18029_18031delCCT), K112A (18027T>G 18028T>C), A116V (18015G>A)								
hypothetical protein (YP_008126560.1)	1	134	95.0%	675.0	69.5%	134 (100%)	91 (67.9%)	0/0/0/0	0
	V1M (19025C>T), S6D (19009G>T 19010A>C), T7S (19005C>G 19006G>C), G8S (19004C>T), D9N (19001C>T), A10L (18997G>A 18998C>G), D11A (18993G>C 18994T>G), T15A (18981A>C 18983T>C), A16T (18980C>T), S21T (18963G>C 18965A>T), V22A (18961A>G), N26D (18950T>C), P38A (18912G>C 18914G>C), T39S (18910G>C), C40A (18906A>C 18907C>G 18908A>C), Q41T (18903C>A 18904T>G 18905G>T), G43P (18897G>C 18898C>G 18899C>G), S51G (18873C>A 18874G>C 18875A>C), P53A (18867C>G 18869G>C), K55R (18861C>G 18862T>C 18863T>G), S58G (18854T>C), A63E (18837A>C 18838G>T), E64D (18834C>A), D67E (18825G>C), S70E (18816G>C 18817G>T 18818A>C), H77A (18795G>C 18796T>G 18797G>C), D80R (18787T>C 18788C>G), V86I (18770C>T), I90V (18758T>C), E91V (18753C>G 18754T>A), G93Q (18747G>T 18748C>T 18749C>G), T95Q (18742G>T 18743T>G), S96T (18740A>T), Q97R (18735C>A 18736T>C), P98V (18733G>A 18734G>C), I99D (18730A>T 18731T>C), S100N (18726T>G 18727G>T 18728A>T), D101E (18723G>C), T102H (18720C>G 18721G>T 18722T>G), Y112L (18690G>C 18691T>A 18692A>G), V117I (18677C>T), D118E (18672G>C)								
hypothetical protein (YP_008126561.1)	45	52	15.4%	30.0	100%	7 (87.5%)	7 (87.5%)	0/1/0/0	1
	F49del (19031_19033delGAA)								
hypothetical protein (YP_008126563.1)	1	3	2.2%	4.0	15.4%	3 (100%)	1 (33.3%)	0/0/0/0	0
	P2S (19737G>C 19738G>T)								
hypothetical protein (YP_008126564.1)	21	131	84.7%	508.0	67.3%	111 (99.1%)	75 (67.0%)	1/0/0/0	0
	V23R (20059A>C 20060C>G), F27M (20046G>C 20048A>T), L35V (20024G>C), E37D (20016C>G), T38S (20013C>A 20015T>A), D39E (20010G>T), H40L (20007G>C 20008T>A), A41E (20005G>T), M45L (19992C>G 19994T>G), V50I (19977A>G 19979C>T), E51D (19974C>G), V55L (19964C>G), D56R (19959G>C 19960T>C 19961C>G), A57P (19958C>G), T61A (19946T>C), R62T (19942C>G 19943G>T), V64I (19935C>G 19937C>T), E68A (19924T>G), I69V (19922T>C), G70E (19917G>T 19918C>T), A71R (19914G>A 19915G>C 19916C>G), E80D (19887C>G), Q83D (19878C>G 19880G>C), T84R (19876G>C 19877T>G), T94E (19846G>T 19847T>C), V95A (19842G>C 19843A>G), Q97V (19836C>G 19837T>A 19838G>C), N99R (19830G>C 19831T>C 19832T>G), M109T (19801A>G), Y121H (19766A>G), P122A (19763G>C), T123_E124insV (19757_19758insGAC), E124S (19756T>G 19757C>A), D127G (19746G>A 19747T>C), G128A (19744C>G), A130E (19737G>C 19738G>T)								

	Begin	End	Coverage	Score	Concordance	Matches	Identities	I/D/M/F*	Stop Codons
proliferating cell nuclear antigen PcnA (YP_008126568.1)	1	228	83.9%	1049.0	78.5%	209 (100%)	159 (76.1%)	0/0/0/0	0
	S2T (21811G>C 21812C>G), V7A (21797A>G), P8E (21794G>T 21795G>C), Q10E (21789G>C), S11T (21786A>T), E13Q (21778T>C 21780C>G), A14D (21776G>T), D17N (21768C>T), D20A (21757G>C 21758T>G), A21T (21754G>C 21756C>T), E32D (21721C>G), A34G (21715C>G 21716G>C), P42A (21691G>C 21693G>C), G46A (21679G>C 21680C>G), V48A (21673G>T 21674A>G), M50V (21667C>G 21669T>C), T51D (21664C>G 21665G>T 21666T>C), D53P (21658G>A 21659T>G 21660C>G), S55R (21653G>C 21654A>G), D61E (21634G>C), E72D (21601C>G), L75Q (21592G>C 21593A>T), V78L (21585C>G), G79S (21581C>G 21582C>A), V87A (21556G>C 21557A>G), A90S (21549C>A), S94E (21535G>C 21536C>T 21537T>C), E99H (21520C>G 21522C>G), D103A (21508G>C 21509T>G), D127E (21436G>C), S131D (21424C>G 21425G>T 21426A>C), E137S (21406C>G 21407T>G 21408C>A), Q138E (21405G>C), F139L (21400G>C), A140S (21398G>C 21399C>T), T144S (21387T>A), T146A (21381T>C), L177V (21288G>C), E183D (21268C>G), I184L (21267T>G), V185A (21262G>C 21263A>G), S186T (21261A>T), D190S (21247G>C 21248T>G 21249C>A), A192R (21242G>C 21243C>G), G194E (21236C>T), T206S (21199C>A 21200G>C), V209I (21192C>T), D210G (21188T>C), L217F (21168G>A)								
hypothetical protein (YP_008126569.1)	1	63	100%	178.0	45.3%	62 (96.9%)	31 (48.4%)	1/1/0/0	1
	H5D (22259G>C), E7D (22251C>G), T8R (22248T>C 22249G>C), T8_D9insR (22247_22248insCCG), D9S (22245G>A 22246T>G 22247C>A), G10P (22242G>C 22243C>G 22244C>G), G11A (22239G>C 22240C>G), E14A (22230C>T 22231T>G), T15R (22227T>G 22228G>C 22229T>G), A16V (22225G>A), F18L (22220A>G), K19A (22215T>C 22216T>G 22217T>C), L20I (22212C>A 22214G>T), A22E (22206T>C 22207G>T), T23Q (22204G>T 22205T>G), S26A (22194C>G 22196A>C), Q27D (22191C>G 22193G>C), A30S (22184C>A), A31R (22180G>C 22181C>G), D33N (22175C>T), G34A (22170A>C 22171C>G), P35T (22167C>G 22169G>T), L36D (22165A>T 22166G>C), Q44E (22142G>C), Q47R (22131C>T 22132T>C), D51G (22120T>C), A52R (22116T>G 22117G>C 22118C>G), S53E (22114G>T 22115A>C), E55T (22107C>A 22108T>G 22109C>T), A57del (22101_22103delTGC), P59L (22095C>T 22096G>A), T60V (22093G>A 22094T>C), L61S (22090A>G 22091G>A)								
hypothetical protein (YP_008126570.1)	1	87	88.8%	356.0	55.8%	87 (96.7%)	49 (54.4%)	3/0/0/0	1
	R2C (22260C>T), H5D (22269C>G), P7S (22275C>T), V8L (22278G>C 22280T>A), N9K (22283T>G), G10D (22285G>A 22286T>C), V11R (22287G>C 22288T>G), L12V (22290T>G 22292A>G), G16N (22302G>A 22303G>A), R22Q (22321G>A 22322T>A), S23H (22323T>C 22324C>A), S25K (22329T>A 22330C>A), S25_V26insTHR (22331_22332insACACACCGT), T27I (22336C>T 22337T>C), T28A (22338A>G), L30M (22344C>A 22346A>G), L31F (22347C>T), N32I (22351A>T), T33K (22354C>A 22355C>G), L34T (22356T>A 22357T>C), S37* (22366C>A), G39V (22372G>T), D40G (22375A>G), E42K (22380G>A), N45H (22389A>C), P52E (22410C>G 22411C>A 22412G>A), G53Q (22413G>C 22414G>A 22415C>G), A54T (22416G>A 22418A>T), L56V (22422C>G), E57D (22427G>C), Q58E (22428C>G), S61G (22437A>G), E64T (22446G>A 22447A>C 22448A>G), D75N (22479G>A), C77I (22485T>A 22486G>T), R79E (22491C>G 22492G>A 22493C>G), E82A (22501A>C), G83R (22503G>C)								
hypothetical protein (YP_008126575.1)	1	90	100%	518.0	84.9%	90 (100%)	78 (86.7%)	0/0/0/0	1
	N17D (23970T>C), D27A (23938A>G 23939T>G), R32N (23924C>T 23925G>T), V37Y (23908C>G 23909A>T 23910C>A), G55D (23855C>T), S58E (23846G>T 23847A>C), N61L (23837T>A 23838T>G), R68K (23816C>T 23817G>T), D74G (23798T>C), F82Y (23774A>T), E83A (23771T>G), R84P (23768C>G)								
hypothetical protein (YP_008126578.1)	30	119	35.6%	558.0	80.8%	89 (98.9%)	73 (81.1%)	0/1/0/0	0
	D46E (25832C>G), G49Q (25839G>C 25840G>A 25841C>G), P51D (25845C>G 25846C>A 25847G>C), H56R (25861A>G), T61V (25875A>G 25876C>T 25877G>C), M63Q (25881A>C 25882T>A), E79D (25931A>C), D81S (25935G>T 25936A>C 25937C>G), E91V (25966A>T 25967G>C), E96H (25980G>C 25982G>C), Q97D (25983C>G 25985G>C), R100K (25992C>A 25993G>A 25994C>G), S101G (25995T>G 25996C>G 25997G>C), Q102E (25998C>G), G106V (26011G>T), P107del (26013_26015delCCG)								

*: Inserts / Deletes / Misaligned / Frameshifts

Detailed Results of Halovirus HCTV-1 (NC_021330.1)

NGS details

Assembly

**Coverage length**	3870 (5 contig(s))
**Est. depth of coverage**	76.5
**Est. number of reads**	3568
**Ambiguities**	0

??coverage-details?? ??variant-analysis??

Assignment

**Type**	Halovirus HCTV-1 (Taxonomy ID: 1273746)
**Reference Genome**	NC_021330.1 (Length: 103257bp)
**Host(s)**	Haloarcula californiae
**NT Identity (%)**	64.8335
**NT Quality**	0.568992

Alignment

**Alignment score**	2202 (NT) + 5547 (AA) = 7749
**Concordance (%)**	46.9665
**Alignment method**	Local, heuristic, nucleotide (BLASTN)

Genome Region

Sequence starts at position null and ends at position null relative to the null reference sequence for null.

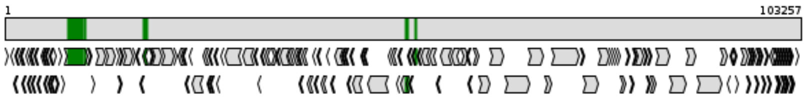

Alignment Detailed Statistics

	Begin	End	Coverage	Score	Concordance	Matches	Identities	I/D/M/F*	Stop Codons
**NT**	8111	53408	3.7%	2202.0	28.9%	3849 (98.8%)	2511 (64.5%)	24/21	
**CDS**									
26	104	913	79.8%	3353.0	61.5%	778 (98.5%)	475 (60.1%)	6/6/0/0	0
38	1	250	100%	1187.0	68.9%	250 (100%)	164 (65.6%)	0/0/0/0	1
90	18	180	47.5%	694.0	61.4%	162 (99.4%)	93 (57.1%)	0/1/0/0	0
93	7	95	45.2%	313.0	51.6%	89 (97.8%)	45 (49.5%)	2/0/0/0	0

	Begin	End	Coverage	Score	Concordance	Matches	Identities	I/D/M/F*	Stop Codons
**Proteins**									
ribonucleotide reductase (YP_008059588.1)	104	913	79.8%	3353.0	61.5%	778 (98.5%)	475 (60.1%)	6/6/0/0	0
	K111R (8133A>C 8134A>G), L113F (8139C>T), D114A (8143A>C 8144C>A), E116D (8150A>C), S117T (8152G>C), S119V (8157T>G 8158C>T 8159C>T), E120P (8160G>C 8161A>C 8162G>C), V121D (8164T>A 8165T>C), A123del (8169_8171delGCA), E125A (8176A>C 8177A>G), A126S (8178G>T), R127V (8181C>G 8182G>T 8183A>T), L130V (8190C>G), L130_E131insV (8192_8193insGTT), S132H (8196T>C 8197C>A), T133W (8199A>T 8200C>G), D135E (8207C>A), D139E (8219C>G), E142G (8227A>G 8228G>T), F146W (8239T>G 8240C>G), I147M (8243C>G), T150S (8251C>G), L153I (8259C>A), I154M (8264C>G), M175N (8326T>A 8327G>C), E176V (8329A>T 8330G>C), D180E (8342C>G), A182I (8346G>A 8347C>T 8348G>C), K183T (8350A>C), A186S (8358G>A 8359C>G 8360G>T), L187Q (8362T>A 8363C>G), T188I (8365C>T 8366G>C), Q190K (8370C>A), S191T (8373T>A 8375A>C), M195I (8387G>C), A198D (8395C>A), F199W (8398T>G 8399C>G), R201K (8403C>A 8404G>A 8405A>G), A208P (8424G>C 8426T>G), I220L (8460A>C 8462C>G), R224Q (8473G>A 8474T>G), Y226A (8478T>G 8479A>C), K231E (8493A>G), T238V (8514A>G 8515C>T 8516G>C), G245A (8536G>C 8537T>G), V246N (8538G>A 8539T>A), S248H (8544T>C 8545C>A 8546G>C), D250T (8550G>A 8551A>C 8552C>A), I254V (8562A>G 8564C>G), Q256E (8568C>G 8570A>G), N262R (8586A>C 8587A>G 8588C>G), L269H (8608T>A 8609T>C), S270T (8610T>A), G272K (8616G>A 8617G>A 8618T>A), K277D (8631A>G 8633G>C), P279F (8637C>T 8638C>T 8639G>T), N281H (8643A>C), S283T (8649T>A 8651C>G), T285S (8656C>G 8657T>C), D289E (8669C>G), G293E (8680G>A 8681C>A), E296D (8690G>C), D297_G298insE (8693_8694insGAG), K299R (8698A>G 8699G>A), N302E (8706A>G 8708C>A), F303H (8709T>C 8710T>A), I308V (8724A>G), Q310G (8730C>G 8731A>G), H311N (8733C>A 8735T>C), G320T (8760G>A 8761G>C 8762T>C), H323D (8769C>G), R324E (8772C>G 8773G>A), M326L (8778A>T 8780G>A), L329K (8787C>A 8788T>A 8789C>G), E330D (8792G>C), D334S (8802G>T 8803A>C), Y335L (8805T>C 8806A>T 8807C>G), F337M (8811T>A 8813C>G), I338V (8814A>G), D343A (8830A>C 8831C>A), S349T (8848G>C 8849T>C), Q350E (8850C>G 8852A>G), E351Q (8853G>C), L355T (8865C>A 8866T>C), W356Y (8869G>A 8870G>C), S357G (8871T>G 8872C>G 8873T>C), D360G (8881A>G 8882C>G), H363D (8889C>G 8891C>T), H364Q (8894C>A), S366E (8898A>G 8899G>A 8900C>G), P370V (8910C>G 8911C>T 8912G>T), S372E (8916T>G 8917C>A 8918T>A), P374D (8922C>G 8923C>A 8924C>T), E376T (8928G>A 8929A>C 8930G>C), D377A (8932A>C 8933T>G), V378L (8934G>T), D380E (8942C>G), H381R (8944A>G 8945T>C), V383I (8949G>A), Y387H (8961T>C 8963T>C), F396H (8988T>C 8989T>A), L397I (8991C>A 8993G>C), D398S (8994G>A 8995A>G 8996C>T), D402R (9006G>C 9007A>G), E403H (9009G>C 9011G>C), S405A (9015T>G 9017C>A), D407P (9021G>C 9022A>C 9023C>G), V408A (9025T>C 9026G>C), E410_H411del (9030_9035delGAGCAT), E413D (9041G>T), H414A (9042C>G 9043A>C 9044C>G), E415T (9045G>A 9046A>C), I416L (9048A>C), I417G (9051A>G 9052T>G 9053C>G), A418S (9054G>T 9056C>A), T419E (9057A>G 9058C>A), N420E (9060A>G 9062C>G), P421Y (9063C>T 9064C>A), C422H (9066T>C 9067G>A), G423R (9069G>C 9071C>T), E424K (9072G>A), Q425E (9075C>G 9077A>G), P426Y (9078C>T 9079C>A 9080T>C), L427I (9081C>A), E428T (9084G>A 9085A>C), E429A (9088A>C), Y430T (9090T>A 9091A>C 9092C>G), E431N (9093G>A 9095G>C), A432P (9096G>C 9098C>G), N434G (9102A>G 9103A>G), L435E (9105C>G 9106T>A 9107C>G), A436E (9109C>A 9110T>A), H437P (9112A>C 9113T>G), I438L (9114A>T 9116C>G), N439M (9118A>T 9119C>G), L440E (9120C>G 9121T>A 9122C>G), S441Y (9124C>A 9125G>C), T442D (9126A>G 9127C>A), V443A (9130T>C), V444C (9132G>T 9133T>G), T445N (9136C>A), E446L (9138G>C 9139A>T 9140A>G), G447_A448insHINL (9143_9144insCACATCAACCTC), A448S (9144G>T 9146G>T), P449T (9147C>A 9149T>G), D450A (9151A>C), W451V (9153T>G 9154G>T), R452A (9156C>G 9157G>C), E453N (9159G>A 9161G>C), V454D (9163T>A), R455A (9165C>G 9166G>C 9167T>A), E457D (9173G>C), Y458W (9175A>G 9176T>G), L459R (9178T>G 9179C>A), E460T (9180G>A 9181A>C 9182A>G), G461F (9183G>T 9184G>T), T462del (9186_9188delACA), E463K (9189G>A), D464A (9193A>C 9194C>G), E465Q (9195G>C), W466S (9199G>C), M467E (9201A>G 9202T>A), D468A (9205A>C 9206C>A), E470S (9210G>A 9211A>G 9212A>C), L471R (9214T>G), R473E (9219C>G 9220G>A 9221T>A), H474Q (9224C>G), V475I (9225G>A 9227T>A), R476A (9228C>G 9229G>C), E477A (9232A>C 9233A>C), D480E (9242C>G), D481K (9243G>A 9245C>A), A487R (9261G>C 9262C>G), F488L (9264T>C 9266C>G), D489N (9267G>A), I492V (9276A>G), Y493D (9279T>G), H497R (9292A>G), E514D (9344G>C), S518G (9354T>G 9355C>G), S519A (9357T>G), L520T (9360C>A 9361T>C), V523I (9369G>A), Y530L (9390T>C 9391A>T), L534Y (9402C>T 9403T>A), V535I (9405G>A 9407T>C), I537V (9411A>G), V539L (9417G>C 9419G>C), P540K (9420C>A 9421C>A 9422A>G), T543S (9429A>T 9431G>C), P544E (9432C>G 9433C>A 9434T>G), A545D (9436C>A 9437G>C), V549A (9448T>C), Q555T (9465C>A 9466A>C 9467G>C), Y556H (9468T>C), E560R (9480G>C 9481A>G 9482G>C), E564A (9493A>C), H566R (9499A>G 9500T>C), H567Q (9503T>A), D570E (9512T>A), D571E (9515C>G), A574S (9522G>T 9524G>T), E577W (9531G>T 9532A>G), Y578W (9535A>G 9536C>G), E579D (9539G>C), K580D (9540A>G 9542G>C), Y583F (9550A>T), N585D (9555A>G 9557C>T), Q588A (9564C>G 9565A>C), E591D (9575G>C), S595R (9585T>C 9586C>G 9587T>C), P601S (9603C>T 9605G>C), S602A (9606A>G 9607G>C 9608T>G), E605A (9616A>C 9617A>C), F608Y (9625T>A 9626C>T), E609P (9627G>C 9628A>C), V610M (9630G>A 9632T>G), H613Y (9639C>T), G628A (9685G>C 9686A>G), N629D (9687A>G 9689C>T), S639N (9717T>A 9718C>A 9719T>C), N642F (9726A>T 9727A>T), L643F (9729C>T 9731C>T), Q648D (9744C>G 9746G>C), I650V (9750A>G), E653D (9761G>C), D654E (9764C>G), E655M (9765G>A 9766A>T), Q658E (9774C>G 9776A>G), V666T (9798G>A 9799T>C 9800T>G), V672I (9816G>A), N673D (9819A>G 9821C>T), K676E (9828A>G), T677A (9831A>G 9833G>C), K679R (9837A>C 9838A>G), Y682V (9846T>G 9847A>T), L684del (9852_9854delCTT), S687N (9862G>A 9863C>T), E688K (9864G>A 9866G>A), D689N (9867G>A), G690A (9871G>C 9872G>C), H695E (9885C>G 9887C>G), Q696S (9888C>T 9889A>C 9890A>T), S698E (9894A>G 9895G>A 9896C>G), A703E (9910C>A 9911C>A), I704L (9912A>C), D706E (9920C>A), S712Q (9936A>C 9937G>A 9938C>G), G716A (9949G>C), R717L (9952G>T), F726L (9978T>C), E728H (9984G>C 9986A>C), S732A (9996T>G 9998G>C), F740A (10020T>G 10021T>C 10022C>A), S742H (10026T>C 10027C>A 10028G>C), T743D (10029A>G 10030C>A), R746V (10038C>G 10039G>T 10040C>G), V749T (10047G>A 10048T>C), R750A (10050C>G 10051G>C 10052T>A), E751D (10055G>C), V752A (10057T>C 10058G>C), Y753F (10060A>T), E754D (10064G>T), I756V (10068A>G), H757L (10072A>T), A758K (10074G>A 10075C>A 10076G>A), V766Y (10098G>T 10099T>A 10100G>C), V768R (10104G>C 10105T>G 10106T>C), S773A (10119T>G 10121G>A), D815R (10245G>C 10246A>G 10247C>T), S816G (10248T>G 10249C>G), T817V (10251A>G 10252C>T 10253G>C), V818T (10254G>A 10255T>C 10256G>C), Y819Q (10257T>C 10259C>A), P820R (10261C>G), N822D (10266A>G 10268C>T), E827K (10281G>A), L828V (10284C>G 10286T>G), N829Y (10287A>T), F831N (10293T>A 10294T>A), V832I (10296G>A), N836E (10308A>G 10310C>A), A837H (10311G>C 10312C>A), D839Q (10317G>C 10319C>G), V843L (10329G>T), E846N (10338G>A 10340A>C), K849N (10349G>C), H855A (10365C>G 10366A>C 10367C>A), V861L (10383G>C 10385C>G), G862A (10387G>C 10388A>G), R863K (10389C>A 10390G>A 10391T>G), L864T (10392C>A 10393T>C 10394C>G), V865I (10395G>A 10397G>C), L867T (10401C>A 10402T>C 10403C>T), I873V (10419A>G 10421C>G), P874D (10422C>G 10423C>A 10424G>T), E876N (10428G>A 10430A>C), V878L (10434G>C), I879A (10437A>G 10438T>C 10439C>T), Q881E (10443C>G 10445G>A), D883Q (10449G>C 10451T>A), T890I (10471C>T 10472G>C), G891A (10474G>C), N893D (10479A>G 10481C>T), T894K (10483C>A 10484G>A), G896del (10488_10490delGGT), Q897E (10491C>G), V906I (10518G>A), A907G (10522C>G 10523G>C), H908T (10524C>A 10525A>C 10526C>G)								
thymidylate synthase (YP_008059600.1)	1	250	100%	1187.0	68.9%	250 (100%)	164 (65.6%)	0/0/0/0	1
	Q2K (18600G>T), K4D (18592T>A 18594T>C), E7N (18583C>G 18585C>T), D10E (18574G>C), E11N (18571T>G 18573C>T), A12P (18568T>C 18570C>G), E14R (18562C>T 18563T>C 18564C>G), V15L (18561C>G), I16V (18558T>C), R18Q (18550T>C 18551C>T), S26E (18526G>C 18527C>T 18528T>C), G29V (18517A>G 18518C>A), A30G (18514T>G 18515G>C), M32T (18508C>T 18509A>G), P33A (18505C>G 18507G>C), S35A (18499G>C 18500C>G 18501T>C), S39A (18487C>G 18489A>C), S40D (18485C>T 18486T>C), D44E (18472G>C), S45T (18469G>T 18471A>T), I46L (18468T>G), E47D (18463C>G), G51E (18451T>C 18452C>T), I54V (18442G>C 18444T>C), G55H (18439C>G 18440C>T 18441C>G), N56R (18437T>C 18438T>G), M58L (18432T>G), D59A (18427G>T 18428T>G), Y63F (18415A>G 18416T>A), V69P (18397G>C 18398A>G 18399C>G), Q70H (18394C>G), H74A (18382G>T 18383T>G 18384G>C), V75I (18381C>T), I78V (18372T>C), I86L (18346A>G 18348T>G), P103E (18296G>T 18297G>C), T110D (18274C>G 18275G>T 18276T>C), A114I (18262A>T 18263G>A 18264C>T), V115Y (18260A>T 18261C>A), T117R (18254G>C 18255T>G), E122D (18238C>G), D123E (18235G>C), P124A (18232C>T 18234G>C), T125G (18229G>C 18230G>C 18231T>C), I126L (18226G>C 18228T>G), F133L (18205A>G 18207A>G), V136K (18196C>T 18197A>T 18198C>T), F137H (18194A>T 18195A>G), E138R (18191T>C 18192C>G), D141S (18182T>G 18183C>A), P142D (18178A>G 18179G>T 18180G>C), E143A (18175T>C 18176T>G), E144A (18172C>T 18173T>G), I145L (18169A>C 18171T>G), E147D (18163C>G), K148R (18160T>C 18161T>C 18162T>G), D151E (18151G>C), V152R (18148C>G 18149A>C 18150C>G), E154H (18142C>G 18144C>G), Q155E (18139T>C 18141G>C), A156S (18136C>G 18138C>A), M157I (18133C>G), K158E (18130T>C 18132T>C), E159A (18127C>G 18128T>G), M162D (18118C>G 18119A>T 18120T>C), N165G (18110T>C 18111T>C), E166D (18106C>G), D169E (18097G>T), A172V (18088A>G 18089G>A), K186R (18047T>C), M189I (18037C>G), S192T (18028C>G 18030A>T), M197T (18013C>G 18014A>G), A202G (17998T>G 17999G>C), M204L (17992C>G 17994T>G), I214V (17962A>G 17964T>C), K216E (17958T>C), G220Q (17944T>C 17945C>T 17946C>G), I221M (17941G>C), E226A (17926T>G 17927T>G), D227E (17923A>C), Y231H (17913A>G), N237E (17893G>C 17895T>C), E238S (17890C>G 17891T>G 17892C>A), N239E (17887G>T 17889T>C), N242G (17879T>C 17880T>C)								
replication factor C small subunit (YP_008059651.1)	18	180	47.5%	694.0	61.4%	162 (99.4%)	93 (57.1%)	0/1/0/0	0

	Begin	End	Coverage	Score	Concordance	Matches	Identities	I/D/M/F*	Stop Codons
	V22L (52343A>C 52345C>G), K24Q (52339T>G), E28Q (52327C>G), T29R (52322T>C 52323G>C 52324T>G), D31E (52316G>T), I33V (52310T>G 52312T>C), H36Q (52301G>C), E38A (52296T>G), V39I (52294C>T), K41E (52288T>C), M43L (52280C>G 52282T>G), R44Q (52278C>T 52279T>G), K45T (52275T>G), F46Y (52272A>T), L47V (52270G>C), T49Q (52262G>C 52263G>T 52264T>G), E50N (52259C>G 52261C>T), V52L (52253C>G 52255C>G), P53S (52250A>C 52252G>A), V55L (52246C>G), V56L (52243C>G), S58A (52237A>C), K60P (52229C>T 52230T>G 52231T>G), Q61A (52226T>G 52227T>G 52228G>C), I63V (52222T>C), A67T (52210C>T), I68S (52206A>C), I69A (52203A>G 52204T>C), Q70L (52200T>A), F72I (52195A>T), K74R (52187C>G 52188T>C 52189T>G), K76L (52182T>A 52183T>G), D79E (52172G>T), N80D (52171T>C), R82E (52163T>C 52164C>T 52165G>C), N83Q (52160G>T 52162T>G), N84H (52157A>G 52159T>G), I85F (52154A>G 52156T>A), T98V (52115G>C 52116G>A 52117T>C), D101E (52106G>T), K102R (52104T>C 52105T>G), N105S (52095T>C), Y106F (52092T>A), V108del (52085_52087delCAC), Q109R (52083T>C), G110T (52080C>G 52081C>T), T111A (52076C>G 52078T>C), I112F (52073A>G 52075T>A), D114G (52067G>C 52068T>C), H115A (52064A>G 52065T>G 52066G>C), Q116E (52063G>C), K118R (52055T>C 52056T>C 52057T>G), V120I (52051C>T), Q127A (52028C>G 52029T>G 52030G>C), K130S (52019T>G 52020T>C), A134S (52007T>G 52009C>A), I139T (51992A>C 51993A>G), E142Q (51985C>G), H143F (51981T>A 51982G>A), A144S (51979C>A), D145N (51976C>T), V146N (51971C>G 51972A>T 51973C>T), F150I (51961A>T), L156S (51941C>G 51942A>G 51943G>A), G161D (51926A>G 51927C>T), P169V (51902C>G 51903G>A 51904G>C), S171R (51896A>C 51898T>G), I172Y (51894A>T 51895T>A), S173A (51892A>C), T176G (51881C>G 51882G>C 51883T>C)								
TMP kinase (YP_008059654.1)	7	95	45.2%	313.0	51.6%	89 (97.8%)	45 (49.5%)	2/0/0/0	0
	I12L (53392T>G), A15S (53381T>G 53382G>C 53383C>T), S19T (53369C>A 53371A>T), T21W (53364G>C 53365T>A), D22E (53360G>C), E25G (53351C>G 53352T>C), E27R (53345T>G 53346T>C 53347C>G), F28Y (53343A>T), F28_D29insP (53341_53342insGGG), D29E (53339G>T), V30A (53337A>G), R32M (53331C>A), Q34R (53324C>G 53325T>C), S37T (53315A>G 53317A>T), E38D (53312C>A), L39S (53309G>C 53310A>G 53311G>A), T41Y (53303C>G 53304G>T 53305T>A), K43D (53297C>G 53299T>C), Q44A (53294C>G 53295T>G 53296G>C), R46Y (53288A>G 53289C>T 53290G>A), A48S (53284C>A), L49V (53281G>C), T50G (53277G>C 53278T>C), T50_E51insD (53275_53276insGTC), E51D (53273C>G), T53A (53267A>G 53269T>C), P54D (53264C>G 53265G>T 53266G>C), F56L (53258G>C 53260A>G), T57A (53257T>C), D58E (53252G>T), F59L (53251A>G), F62Y (53240A>G 53241A>T), M63T (53238A>G), R66H (53228T>G 53229C>T), H67A (53226T>G 53227G>C), L68D (53222C>G 53223A>T 53224G>C), I70L (53218T>G), E71A (53213C>G 53214T>G), T72R (53210T>G 53211G>C 53212T>G), Q73V (53207T>C 53208T>A 53209G>C), K75R (53201T>C 53202T>C 53203T>G), E80D (53186T>G), K82A (53181T>G 53182T>C), F89Y (53160A>T), T90S (53157G>C)								

*: Inserts / Deletes / Misaligned / Frameshifts

Detailed Results of Halorubrum virus HRTV-29 (NC_062762.1)

NGS details

Assembly

**Coverage length**	6480 (18 contig(s))
**Est. depth of coverage**	44.9
**Est. number of reads**	3508
**Ambiguities**	0

??coverage-details?? ??variant-analysis??

Assignment

**Type**	Halorubrum virus HRTV-29 (Taxonomy ID: 2878010)
**Reference Genome**	NC_062762.1 (Length: 36603bp)
**NT Identity (%)**	66.9701
**NT Quality**	0.661265

Alignment

**Alignment score**	4285 (NT) + 9981 (AA) = 14266
**Concordance (%)**	51.1953
**Alignment method**	Local, heuristic, nucleotide (BLASTN)

Genome Region

Sequence starts at position null and ends at position null relative to the null reference sequence for null.

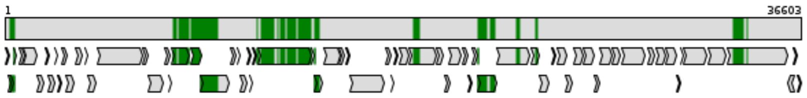

Alignment Detailed Statistics

	Begin	End	Coverage	Score	Concordance	Matches	Identities	I/D/M/F*	Stop Codons
**NT**	208	34196	17.7%	4285.0	33.4%	6458 (99.3%)	4341 (66.7%)	24/22	
**CDS**									
M1M40_gp02	15	93	84.9%	334.0	57.4%	79 (100%)	48 (60.8%)	0/0/0/0	1
M1M40_gp03	1	2	5.0%	6.0	54.5%	2 (100%)	1 (50.0%)	0/0/0/0	0
M1M40_gp21	17	265	84.2%	929.0	53.7%	229 (99.6%)	122 (53.0%)	0/1/0/0	0
M1M40_gp22	1	165	100%	799.0	66.6%	165 (100%)	104 (63.0%)	0/0/0/0	0
M1M40_gp23	1	260	57.9%	1373.0	76.4%	260 (99.2%)	193 (73.7%)	2/0/0/0	0
M1M40_gp29	63	63	1.6%	1.0	16.7%	1 (100%)	1 (100%)	0/0/0/0	1

	Begin	End	Coverage	Score	Concordance	Matches	Identities	I/D/M/F*	Stop Codons
M1M40_gp30	1	817	85.0%	3043.0	62.3%	693 (98.7%)	432 (61.5%)	5/4/1/1	0
M1M40_gp31	41	45	11.1%	24.0	75.0%	5 (100%)	4 (80.0%)	0/0/0/0	0
M1M40_gp32	1	72	53.7%	368.0	70.6%	72 (100%)	50 (69.4%)	0/0/0/0	0
M1M40_gp41	73	178	25.2%	403.0	59.7%	106 (100%)	61 (57.5%)	0/0/0/0	0
M1M40_gp47	79	80	2.5%	9.0	100%	2 (100%)	2 (100%)	0/0/0/0	1
M1M40_gp48	3	271	75.6%	1466.0	89.3%	229 (100%)	200 (87.3%)	0/0/0/0	0
M1M40_gp49	286	353	13.5%	293.0	64.5%	66 (97.1%)	40 (58.8%)	0/2/0/0	0
M1M40_gp50	65	116	40.3%	232.0	65.2%	52 (100%)	35 (67.3%)	0/0/0/0	0
M1M40_gp68	97	325	21.0%	701.0	51.2%	194 (99.5%)	105 (53.8%)	1/0/0/0	0
**Proteins**									
hypothetical protein (YP_010358376.1)	15	93	84.9%	334.0	57.4%	79 (100%)	48 (60.8%)	0/0/0/0	1
	N15D (210A>G 212T>C), P18A (219C>G 221G>A), A19K (222G>A 223C>A), R20K (225C>A 226G>A), I21V (228A>G 230T>C), T24K (238C>A 239A>G), D26R (243G>C 244A>G), W28R (249T>C), R29N (253G>A 254A>C), N30S (256A>G), D31E (260T>A), N33T (265A>C), R35Y (270C>T 271G>A 272G>C), Q36T (273C>A 274A>C), V39T (282G>A 283T>C), S45A (300A>G 301G>C 302T>G), A50T (315G>A), S51M (319G>T 320C>G), D53E (326C>A), S56G (333A>G), D57E (338C>G), T58K (340C>A), P60N (345C>A 346C>A 347G>C), H63G (354C>G 355A>G), A65T (360G>A), G83A (415G>C 416T>C), V85E (421T>A 422C>G), P88T (429C>A 431T>C), K89E (432A>G), E91S (438G>T 439A>C 440G>C), P92A (441C>G)								
hypothetical protein (YP_010358377.1)	1	2	5.0%	6.0	54.5%	2 (100%)	1 (50.0%)	0/0/0/0	0
phosphoadenosine phosphosulfate reductase (YP_010358395.1)	17	265	84.2%	929.0	53.7%	229 (99.6%)	122 (53.0%)	0/1/0/0	0
	N25H (7761A>C 7763C>T), V28L (7770G>C 7772T>C), V33K (7785G>A 7786T>A 7787C>G), Q35M (7791C>A 7792A>T 7793A>G), E37N (7797G>A 7799G>C), Y38G (7800T>G 7801A>G 7802T>C), I40A (7806A>G 7807T>C 7808T>A), W42K (7812T>A 7813G>A), T43V (7815A>G 7816C>T 7817G>T), V44A (7819T>C 7820G>A), Y45H (7821T>C), L46I (7824C>A 7826T>A), N49G (7833A>G 7834A>G), T50I (7837C>T), L52I (7842T>A 7844A>C), D53P (7845G>C 7846A>C), N55T (7852A>C 7853T>G), L56Q (7855T>A 7856T>G), H58F (7860C>T 7861A>T), D61E (7871C>G), V62T (7872G>A 7873T>C), R64E (7878C>G 7879G>A 7880C>A), R65Q (7882G>A), S67G (7887A>G 7889T>G), G98A (7981G>C 7982C>G), I99V (7983A>G 7985C>G), S101I (7990G>T), W102I (7992T>A 7993G>T 7994G>C), A103M (7995G>A 7996C>T 7997A>G), Q105S (8001C>T 8002A>C), Y106K (8004T>A 8006C>A), F107L (8007T>C 8009C>A), Q111A (8019C>G 8020A>C 8021G>A), A114I (8028G>A 8029C>T 8030C>T), T117R (8037A>C 8038C>G), H118W (8040C>T 8041A>G 8042T>G), T119Y (8043A>T 8044C>A 8045C>T), R124I (8058C>A 8059G>T), Y126I (8064T>A 8065A>T 8066C>A), S131K (8079T>A 8080C>A), N135E (8091A>G 8093C>A), R136T (8094C>A 8095G>C 8096C>A), M138F (8100A>T 8102G>C), K139R (8103A>C 8104A>G), G143P (8115G>C 8116G>C), G144E (8119G>A 8120C>A), R145Q (8122G>A 8123G>A), V146del (8124_8126delGTC), E149S (8133G>T 8134A>C), R150S (8136C>A 8138A>T), T152I (8143C>T 8144G>T), V154R (8148G>C 8149T>G 8150A>G), S155A (8151A>G 8152G>C 8153C>G), P156N (8154C>A 8155C>A 8156G>T), V157I (8157G>A 8159A>C), W160F (8167G>T 8168G>C), R161T (8170G>C), D162Q (8172G>C 8174T>G), A164D (8179C>A 8180A>C), M165V (8181A>G 8183G>T), Q167A (8187C>G 8188A>C 8189G>C), R169I (8193C>A 8194G>T), E170D (8198G>C), P175K (8211C>A 8212C>A 8213C>G), E176R (8214G>C 8215A>G 8216G>C), N177S (8218A>G), A180K (8226G>A 8227C>A), K181Q (8229A>C 8231G>A), I183F (8235A>T), G184H (8238G>C 8239G>A 8240C>T), R185H (8242G>A 8243A>T), D188E (8252T>A), Y190L (8256T>C 8257A>T 8258C>G), Y194F (8269A>T 8270T>C), A195G (8272C>G 8273C>A), D198T (8280G>A 8281A>C 8282C>G), A199E (8284C>A 8285A>G), A202L (8292G>T 8293C>T 8294G>A), E203L (8295G>T 8296A>T), D210E (8318T>A), H211T (8319C>A 8320A>C 8321T>G), W214R (8328T>C), L215I (8331C>A), E217A (8338A>C 8339A>G), V218L (8340G>T 8342T>A), R220S (8346C>A), E224G (8359A>G), I226Y (8364A>T 8365T>A 8366A>C), T228N (8371C>A), D231P (8379G>C 8380A>C 8381C>A), Y232R (8382T>C 8383A>G 8384T>G), A233S (8385G>A 8386C>G 8387G>T), G236A (8395G>C 8396C>G), F237H (8397T>C 8398T>A), L240M (8406T>A), E242S (8412G>A 8413A>G 8414A>C), K243T (8416A>C 8417A>C), E244D (8420A>C), K248L (8430A>C 8431A>T 8432A>C), M249L (8433A>T), N251D (8439A>G), D252N (8442G>A), M254E (8448A>G 8449T>A), A255T (8451G>A 8453T>G), S258M (8460T>A 8461C>T), S261A (8469A>G 8470G>C 8471T>A)								
terminase small subunit (YP_010358396.1)	1	165	100%	799.0	66.6%	165 (100%)	104 (63.0%)	0/0/0/0	0
	E4D (8563G>C), L5I (8564C>A), S12K (8586G>A 8587C>A), E14T (8591G>A 8592A>C 8593G>A), E21D (8614G>C), P31A (8642C>G 8644G>A), A33D (8649C>A), S38R (8663T>C 8664C>G 8665C>A), Y42H (8675T>C), E43A (8679A>C 8680G>C), R44K (8681C>A 8682G>A 8683C>G), E47A (8691A>C 8692G>T), C49A (8696T>G 8697G>C), Q50A (8699C>G 8700A>C 8701A>C), A51D (8703C>A), V52I (8705G>A 8707C>T), A54Q (8711G>C 8712C>A 8713G>A), K56R (8717A>C 8718A>G), L58M (8723C>A 8725C>G), T59R (8726A>C 8727C>G 8728G>C), M64K (8742T>A 8743G>A), A65Q (8744G>C 8745C>A 8746C>A), G66D (8748G>A), V67L (8750G>C), D68T (8753G>A 8754A>C 8755C>G), R69P (8757G>C 8758C>G), T70Q (8759A>C 8760C>A), Y73M (8768T>A 8769A>T 8770C>G), G74N (8771G>A 8772G>A), I76M (8779T>G), D77Q (8780G>C 8782C>A), H83L (8798C>T 8799A>T 8800T>A), A85E (8805C>A 8806C>G), G86D (8808G>A), D88E (8815C>G), F91Y (8823T>A), E93Q (8828G>C), Y95F (8835A>T 8836C>T), D96E (8839C>A), L98I (8843C>A 8845T>C), H104Y (8861C>T), D107E (8872C>A), F108W (8874T>G 8875C>G), F110M (8879T>A 8881C>G), G111Q (8882G>C 8883G>A 8884C>G), L112T (8885C>A 8886T>C 8887T>G), A113I (8888G>A 8889C>T 8890G>C), L114I (8891C>A 8893G>C), D115E (8896C>A), K118E (8903A>G 8905A>G), G121E (8913G>A 8914C>G), I126L (8927A>C), A127M (8930G>A 8931C>T 8932C>G), D140E (8971C>A), K151N (9004G>C), L152I (9005C>A 9007G>A), D157R (9020G>C 9021A>G), K159R (9026A>C 9027A>G 9028G>C), R160K (9029C>A 9030G>A 9031C>A), E164N (9041G>A 9043G>C), *165E (9044T>G 9046A>G)								
terminase large subunit (YP_010358397.1)	1	260	57.9%	1373.0	76.4%	260 (99.2%)	193 (73.7%)	2/0/0/0	0

	Begin	End	Coverage	Score	Concordance	Matches	Identities	I/D/M/F*	Stop Codons
	M1V (9021A>G), S3A (9027A>G 9028G>C 9029C>A), A4K (9030G>A 9031C>A), P7Q (9040C>A 9041G>A), S8T (9043G>C 9044T>G), N9S (9046A>G 9047C>T), E11D (9053G>C), E11_T12insGG (9053_9054insGGCGGC), T12G (9054A>G 9055C>G), T13P (9057A>C), L14I (9060T>A 9062A>T), A15D (9064C>A 9065G>C), E16F (9066G>T 9067A>T 9068G>C), F17T (9069T>A 9070T>C), P19L (9076C>T 9077G>C), P21D (9081C>G 9082C>A 9083G>C), A25E (9094C>A), G29A (9106G>C), I50V (9168A>G 9170C>T), R58V (9192C>G 9193G>T), R61A (9201C>G 9202G>C), V76I (9246G>A 9248T>C), D85E (9275T>A), K91N (9293G>T), D93T (9297G>A 9298A>C), I104L (9330A>C), S106A (9336A>G 9337G>C 9338C>A), Q120K (9378C>A), N144D (9450A>G 9452C>T), A147K (9459G>A 9460C>A), S151M (9472G>T 9473C>G), Y163F (9508A>T), S169K (9525T>A 9526C>A 9527C>A), K171R (9532A>G), E173G (9538A>G 9539G>T), G180E (9559G>A 9560C>A), G181R (9561G>C 9563C>A), T182D (9564A>G 9565C>A 9566G>T), D183N (9567G>A), A184V (9571C>T 9572C>A), Q186E (9576C>G), R187T (9579C>A 9580G>C), A188K (9582G>A 9583C>A), S193T (9597T>A 9599A>T), E196D (9608A>T), A197S (9609G>T 9611C>A), L201M (9621C>A 9623T>G), N213E (9657A>G 9659C>A), T214L (9660A>C 9661C>T), D216E (9668C>G), K219Q (9675A>C 9677G>A), D220E (9680C>G), E223V (9688A>T 9689A>T), K224Q (9690A>C 9692G>A), P227A (9699C>G), A232Q (9714G>C 9715C>A 9716C>G), R236M (9726C>A 9727G>T 9728C>G), E238Q (9732G>C), E241Q (9741G>C), S244G (9750A>G), I246L (9756A>C 9758T>C), L247F (9759C>T), T248Q (9762A>C 9763C>A 9764A>G), L252F (9774C>T), H253N (9777C>A), A254W (9780G>T 9781C>G), T255A (9783A>G 9785G>A), N257Q (9789A>C 9791C>A)								
hypothetical protein (YP_010358403.1)	63	63	1.6%	1.0	16.7%	1 (100%)	1 (100%)	0/0/0/0	1
portal protein (YP_010358404.1)	1	817	85.0%	3043.0	62.3%	693 (98.7%)	432 (61.5%)	5/4/1/1	0
	D4N (11632G>A), D4_D5insT (11634_11635insACC), I53L (11779A>T 11781C>G), D54S (11782G>A 11783A>G 11784C>T), E56D (11790G>C), D60E (11802C>A), E66D (11820G>C), Q73A (11839C>G 11840A>C 11841A>G), D76Q (11848G>C 11850T>A), Y77S (11852A>C), E88D (11886G>T), L89I (11887C>A 11889T>A), H90T (11890C>A 11891A>C 11892C>G), E93D (11901G>C), E98A (11915A>C 11916A>C), E99Q (11917G>C 11919G>A), V100S (11920G>A 11921T>G 11922G>C), D102Q (11926G>C 11928C>A), E104T (11932G>A 11933A>C), P105A (11935C>G), T107P (11941A>C), A110E (11951C>A), T114S (11962A>T 11964G>A), Q115E (11965C>G), F116L (11968T>C 11970C>G), R118D (11974C>G 11975G>A 11976G>C), S128M (12005G>T 12006C>G), L131T (12013C>A 12014T>C 12015C>G), W132Y (12017G>A 12018G>T), V137Y (12031G>T 12032T>A 12033T>C), L140V (12040C>G 12042C>T), R141V (12043C>G 12044G>T 12045C>G), E142P (12046G>C 12047A>C 12048G>C), T143N (12050C>A 12051G>C), Q144R (12053A>G 12054G>A), A145L (12055G>C 12056C>T 12057G>T), G146D (12059G>A 12060C>T), D147G (12062A>G 12063C>G), K149S (12067A>T 12068A>C), E150H (12070G>C 12072G>C), F151I (12073T>A 12075C>T), L152E (12076C>G 12077T>A 12078C>G), P153G (12079C>G 12080C>G), A154I (12082G>A 12083C>T 12084G>A), V159I (12097G>A 12099G>C), L160K (12100T>A 12101T>A), V162E (12107T>A), N164D (12112A>G 12114C>T), R166Y (12118C>T 12119G>A), E168D (12126G>C), I169del (12127_12129delATC), A171L (12133G>C 12134C>T), W172R (12136T>C), K173Y (12139A>T 12141A>T), Q174W (12142C>T 12143A>G), I177L (12151A>T 12153T>G), N179S (12157A>T 12158A>C 12159C>G), T181del (12163_12165delACG), P182E (12166C>G 12167C>A 12168C>A), Q185A (12175C>G 12176A>C), T186L (12178A>C 12179C>T 12180G>T), L187E (12181C>G 12182T>A 12183C>A), N188P (12184A>C 12185A>C 12186C>A), V191I (12193G>A 12195G>T), L192G (12196C>G 12197T>G 12198G>C), N194del (12202_12204delAAC), Q200A (12220C>G 12221A>C 12222G>A), A202S (12226G>T 12228A>T), V211L (12253G>C), D215K (12265G>A 12267T>A), D216H (12268G>C 12270C>T), I218V (12274A>G), Q219E (12277C>G), A220T (12280G>A 12282G>A), K222R (12286A>C 12287A>G 12288A>C), E225Q (12295G>C), I228V (12304A>G 12306T>G), N229V (12307A>G 12308A>T 12309C>T), N230E (12310A>G 12312C>G), A231V (12314C>T 12315G>T), I232M (12318T>G), E233R (12319G>C 12320A>G 12321A>T), L234R (12323T>G 12324C>G), H235M (12325C>A 12326A>T 12327C>G), F237H (12331T>C 12332T>A), Q239W (12337C>T 12338A>G), V242A (12347T>C 12348G>A), E248G (12365A>G 12366G>A), G249A (12368G>C), P251G (12373C>G 12374C>G), V252L (12376G>T 12378G>A), R253N (12379C>A 12380G>A), D255T (12385G>A 12386A>C 12387C>G), D256Q (12388G>C 12390C>A), H297Q (12513C>A), M299R (12517A>C 12518T>G), N303M (12530A>T 12531C>G), T305A (12535A>G), L308I (12544C>A), L312I (12556C>A), A314L (12562G>C 12563C>T 12564G>C), G315L (12565G>T 12566G>T 12567C>G), V317E (12572T>A 12573G>A), A319S (12577G>A 12578C>G 12579G>C), K353Q (12679A>C 12681G>A), V354F (12682G>T), V355L (12685G>C 12687G>T), V358I (12694G>A), I359V (12697A>G), D361Q (12703G>C 12705C>G), S363T (12709T>A 12711G>C), P364E (12712C>G 12713C>A), A369Q (12727G>C 12728C>A 12729G>A), T370D (12730A>G 12731C>A 12732G>C), V371F (12733G>T), T372G (12736A>G 12737C>G 12738G>C), M373I (12741G>T), S374K (12743G>A 12744C>A), D376K (12748G>A 12750C>G), D377P (12751G>C 12752A>C 12753C>G), P378F (12754C>T 12755C>T 12756G>C), E380D (12762G>C), D381K (12763G>A 12765T>G), I382K (12767T>A 12768T>A), G383E (12770G>A 12771C>G), T385D (12775A>G 12776C>A 12777G>T), D387A (12782A>C 12783C>G), L388F (12784C>T 12786G>C), I389V (12787A>G 12789T>C), I392V (12796A>G 12798C>G), G393E (12800G>A 12801G>A), D394E (12804T>G), M396L (12808A>C 12810G>T), T397R (12811A>C 12812C>G 12813C>G), N398T (12815A>C 12816C>G), A399D (12818C>A), A401I (12823G>A 12824C>T 12825G>A), R403E (12829C>G 12830G>A 12831T>A), P408E (12844C>G 12845C>A 12846C>A), E409P (12847G>C 12848A>C 12849A>G), P410L (12851C>T 12852G>T), V415K (12865G>A 12866T>A 12867G>A), E417Q (12871G>C), Y419F (12878A>T), A423Q (12889G>C 12890C>A 12891C>A), D424A (12893A>C 12894T>G), I425V (12895A>G), R427E (12901C>G 12902G>A 12903C>G), D428Q (12904G>C 12906C>A), Q433D (12919C>G 12921A>T), D444A (12953A>C), K446R (12958A>C 12959A>G), T447S (12961A>T), E450D (12972G>C), E457D (12993G>C), D464S (13012G>T 13013A>C), A485E (13076C>A 13077C>A), P489L (13088C>T), D511N (13153G>A), D512E (13158C>A), F518Y (13175T>A), E520Q (13180G>C 13182G>A), K539Q (13237A>C), D541S (13243G>A 13244A>G), A545E (13256C>A 13257G>A), A547E (13262C>A 13263C>A), G553S (13279G>A 13281G>T), F556W (13289T>G 13290T>G), D569T (13327G>A 13328A>C 13329C>G), E580S (13360G>T 13361A>C), G584D (13373G>A), S591del (13393_13395delTCG), Q592E (13396C>G), G593A (13400G>C), P633Q (13520C>A 13521G>A), N634D (13522A>G), A636T (13528G>A), V638F (13534G>T 13536G>T), A641S (13543G>T 13545C>A), A642D (13547C>A 13548A>C), E644A (13553A>C 13554G>C), T645M (13556C>T), V649I (13567G>A 13569G>C), L650K (13570C>A 13571T>A 13572T>A), I653L (13579A>C 13581C>G), E655D (13587G>C), A656V (13589C>T 13590T>A), M658L (13594A>T 13596G>A), G660_A661insE (13602_13603insGAA), T662L (13606A>C 13607C>T 13608G>C), S664G (13612A>G 13614C>G), H665A (13615C>G 13616A>C 13617C>G), F666I (13618T>A), E667Q (13621G>C 13623G>A), D670S (13630G>A 13631A>G), D671S (13633G>A 13634A>G 13635C>T), G672D (13637G>A), R673E (13639C>G 13640G>A 13641C>A), I674L (13642A>C 13644T>C), E676S (13648G>A 13649A>G 13650G>C), Q679T (13657C>A 13658A>C), T680Q (13660A>C 13661C>A), F681L (13665T>G), A684G (13673C>G 13674G>A), G686T (13678G>A 13679G>C 13680C>A), T687Q (13681A>C 13682C>A 13683C>A), D689G (13688A>G), F690W (13691T>G 13692T>G), L692I (13696C>A 13698C>T), S694Q (13702A>C 13703G>A 13704C>G), M695I (13707G>T), E697G (13712A>G 13713G>C), D698R (13714G>C 13715A>G 13716C>G), I699L (13717A>T 13719T>G), M700Q (13720A>C 13721T>A 13722G>A), F702V (13726T>G 13728T>G), F702_A703insQ (13728_13729insCAA), A703P (13729G>C 13731C>G), G706S (13738G>T 13739G>C 13740G>A), D708S (13744G>A 13745A>G 13746C>T), R712T (13756C>A 13757G>C 13758T>C), S718T (13775G>C 13776T>C), S719A (13777A>G 13778G>C 13779T>C), A720S (13780G>T), L722V (13786C>G), K724T (13793A>C), I728Q (13804A>C 13805T>A 13806T>A), E731K (13813G>A), Q733T (13819C>A 13820A>C 13821G>C), E735F (13825G>T 13826A>T 13827G>T), G736E (13829G>A 13830T>A), R739E (13837C>G 13838G>A), T743V (13849A>G 13850C>T 13851C>T), D746T (13858G>A 13859A>C), P747K (13861C>A 13862C>A 13863C>A), G748A (13865G>C), D749E (13869T>A), D749_S750insQP (13869_13870insCAACCC), S750D (13870A>G 13871G>A 13872T>C), Q752T (13876C>A 13877A>C), E757D (13893G>T), Q762E (13906C>G), F766N (13918T>A 13919T>A), D770N (13930G>A), P773S (13939C>A 13940C>G 13941G>T), M774L (13942A>C 13944G>T), Q776R (13949A>G), R778K (13954C>A 13955G>A 13956C>G), D779E (13959C>A), M780L (13960A>C), V781I (13963G>A 13965A>T), D789P (13987G>C 13988A>C), A792Q (13996G>C 13997C>A 13998T>A), N793D (13999A>G), T794D (14002A>G 14003C>A 14004G>C), E799D (14019A>T), S800N (14021G>A), W801F (14024G>T 14025G>C), V802T (14026G>A 14027T>C 14028G>C), S809K (14048G>A 14049C>G), F811Y (14054T>A), A812V (14057C>T 14058G>C), L813R (14060T>G)								
hypothetical protein (YP_010358405.1)	41	45	11.1%	24.0	75.0%	5 (100%)	4 (80.0%)	0/0/0/0	0
	*45R (14256T>C)								
peroxide stress protein YaaA (YP_010358406.1)	1	72	53.7%	368.0	70.6%	72 (100%)	50 (69.4%)	0/0/0/0	0
	A4V (14261C>T 14262C>A), S8Q (14272T>C 14273C>A), G11N (14281G>A 14282G>A 14283G>C), T12A (14284A>G 14286A>G), H16E (14296C>G 14298C>A), E17P (14299G>C 14300A>C), R21K (14311C>A 14312G>A 14313G>A), M22D (14314A>G 14315T>A 14316G>C), F30Y (14339T>A), R31N (14341C>A 14342G>A 14343T>C), K32R (14344A>C 14345A>G), A35K (14353G>A 14354C>A), S39A (14365T>G 14367A>T), C42D (14374T>G 14375G>A), E43P (14377G>C 14378A>C), L54V (14410C>G 14412A>G), S55D (14413T>G 14414C>A 14415A>T), E58T (14422G>A 14423A>C 14424G>C), P59V (14425C>G 14426C>T 14427G>T), V60L (14428G>C), A61D (14432C>A)								
major capsid protein (YP_010358415.1)	73	178	25.2%	403.0	59.7%	106 (100%)	61 (57.5%)	0/0/0/0	0

	Begin	End	Coverage	Score	Concordance	Matches	Identities	I/D/M/F*	Stop Codons
	T81M (18807C>T), D84Q (18815G>C 18817C>G), I87D (18824A>G 18825T>A 18826C>T), N88D (18827A>G), A89N (18830G>A 18831C>A), D90A (18834A>C), S91L (18836A>C 18837G>T 18838T>C), S92P (18839T>C), K93G (18842A>G 18843A>G 18844G>C), L94D (18845C>G 18846T>A 18847C>T), P95A (18848C>G 18850C>T), K96R (18851A>C 18852A>G 18853G>T), K99S (18861A>G 18862G>T), S100A (18863A>G 18864G>C), K102E (18869A>G 18871G>A), R103P (18873G>C 18874A>C), L104K (18875C>A 18876T>A 18877T>G), E106L (18881G>C 18882A>T 18883G>A), D107S (18884G>T 18885A>C 18886C>A), G108D (18888G>A), D109G (18891A>G), V110T (18893G>A 18894T>C), I111L (18896A>C), E112A (18900A>C), R114P (18906G>C 18907G>C), A118T (18917G>A 18919G>T), P120K (18923C>A 18924C>A), T126S (18941A>T 18943C>T), D128T (18947G>A 18948A>C), P129N (18950C>A 18951C>A), V143T (18992G>A 18993T>C 18994C>G), I144V (18995A>G), R148S (19007C>A), E149R (19010G>C 19011A>G), L150R (19014T>G), K152Q (19019A>C), V153I (19022G>A 19024G>T), A157S (19034G>A 19035C>G 19036G>T), S158A (19037T>G), I161L (19046A>C 19048T>C), A163V (19053C>T 19054C>T), N164D (19055A>G), V167K (19064G>A 19065T>A 19066C>G), V172I (19079G>A 19081C>T), S174E (19085T>G 19086C>A)								
hypothetical protein (YP_010358421.1)	79	80	2.5%	9.0	100%	2 (100%)	2 (100%)	0/0/0/0	1
N4-cytosine methyltransferase (YP_010358422.1)	3	271	75.6%	1466.0	89.3%	229 (100%)	200 (87.3%)	0/0/0/0	0
	N10D (21755A>G), A11V (21759C>T 21760G>T), E21S (21788G>T 21789A>C), N24H (21797A>C 21799C>T), C25M (21800T>A 21801G>T 21802C>G), E41A (21849A>C), F53Y (21885T>A 21886T>C), K55Q (21890A>C 21892A>G), E65K (21920G>A), K83D (21974A>G 21976G>C), N100S (22025A>T 22026A>C 22027C>G), I132V (22121A>G 22123A>G), Y142W (22152A>G 22153T>G), E151S (22178G>T 22179A>C), V155A (22191T>C 22192A>G), S156T (22193T>A), A209T (22352G>A 22354A>G), I210T (22356T>C 22357A>C), S211K (22359G>A 22360C>A), G212P (22361G>C 22362G>C), L213F (22364C>T 22366T>C), S214P (22367T>C 22369A>C), V216A (22374T>C 22375T>A), P235A (22430C>G 22432G>T), K236P (22433A>C 22434A>C 22435A>G), I239V (22442A>G), D260K (22505G>A 22507T>G)								
calcineurin-like phosphoesterase domain, ApaH type (YP_010358423.1)	286	353	13.5%	293.0	64.5%	66 (97.1%)	40 (58.8%)	0/2/0/0	0
	V290I (23510G>A 23512G>T), L295F (23525C>T), N297_D298del (23531_23536delAACGAC), V300L (23540G>T), E301D (23545A>T), F303W (23550T>G 23551C>G), I308H (23564A>C 23565T>A), D309E (23569C>G), L310I (23570T>A), H312T (23576C>A 23577A>C 23578T>A), D313T (23579G>A 23580A>C 23581C>G), V315L (23585G>C 23587A>C), L316I (23588T>A 23590A>C), R317G (23591C>G), L318Q (23595T>A 23596T>A), I319L (23597A>C 23599A>C), E320K (23600G>A), Y322F (23607A>T), A323S (23609G>A 23610C>G 23611C>T), R325E (23615C>G 23616G>A), N328T (23625A>C 23626C>T), V330Q (23630G>C 23631T>A 23632T>G), K340Q (23660A>C 23662G>A), K348R (23684A>C 23685A>G 23686G>C), N350A (23690A>G 23691A>C), G352S (23696G>T 23697G>C)								
hypothetical protein (YP_010358424.1)	65	116	40.3%	232.0	65.2%	52 (100%)	35 (67.3%)	0/0/0/0	0
	A68Y (24400G>T 24401C>A 24402T>C), G71A (24410G>C 24411C>G), L72I (24412C>A 24414A>C), H78N (24430C>A), T82E (24442A>G 24443C>A 24444A>G), H83Y (24445C>T), M85V (24451A>G 24453G>C), M88P (24460A>C 24461T>C), V91I (24469G>A 24471T>C), G99A (24494G>C 24495A>C), L102Q (24503T>A 24504T>G), A108T (24520G>A), D109S (24523G>A 24524A>G), A110L (24526G>C 24527C>T), D111E (24531C>G), E112D (24534A>C)								
baseplate hub (YP_010358442.1)	97	325	21.0%	701.0	51.2%	194 (99.5%)	105 (53.8%)	1/0/0/0	0
	D101E (33526C>A), A102Q (33527G>C 33528C>A), V105A (33537T>C), R116G (33569C>G 33571G>C), N117D (33572A>G 33574C>T), T118E (33575A>G 33576C>A 33577C>G), T120S (33582C>G 33583G>C), R124T (33593C>A 33594G>C), N126H (33599A>C), T129F (33608A>T 33609C>T 33610G>T), Q130E (33611C>G), S133E (33620A>G 33621G>A 33622C>A), I135V (33626A>G 33628T>G), I136L (33629A>T 33631C>G), Q137T (33632C>A 33633A>C), D139_T140insV (33640_33641insGTG), T140P (33641A>C), S143E (33650T>G 33651C>A 33652C>A), Y144W (33654A>G 33655C>G), V145E (33657T>A 33658C>A), A146P (33659G>C 33661G>C), G147A (33663G>C 33664G>C), D150E (33673C>A), G152V (33678G>T 33679C>T), D154N (33683G>A), Q155V (33686C>G 33687A>T 33688A>T), R159Q (33699G>A), Y161F (33705A>T), P165T (33716C>A 33718G>C), N168E (33725A>G 33727C>G), V169F (33728G>T 33730G>C), R171F (33734C>T 33735G>T), D172A (33738A>C), F173L (33742T>A), A174R (33743G>C 33744C>G 33745G>C), F175Q (33746T>C 33747T>A 33748T>A), N178G (33755A>G 33756A>G 33757C>T), N179G (33758A>G 33759A>G 33760C>G), E181L (33764G>C 33765A>T 33766G>A), F182P (33767T>C 33768T>C 33769T>C), D186E (33781T>G), F191T (33794T>A 33795T>C 33796T>G), F192W (33798T>G 33799C>G), R193G (33800C>G 33802G>A), R195A (33806C>G 33807G>C 33808T>C), T197R (33812A>C 33813C>G), E198T (33815G>A 33816A>C 33817G>A), H199R (33819A>G 33820T>C), I200T (33822T>C 33823T>C), D201R (33824G>C 33825A>G), G203D (33831G>A 33832C>T), T207S (33842A>T 33844A>G), Q208E (33845C>G), F210I (33851T>A), R211N (33854C>A 33855G>A 33856G>C), Y212H (33857T>C), P215G (33866C>G 33867C>G 33868C>T), L217E (33872C>G 33873T>A 33874C>A), G218S (33875G>T 33876G>C), K219G (33878A>G 33879A>G 33880A>C), A221T (33884G>A), I222K (33888T>A 33889C>G), E224Q (33893G>C 33895G>A), E226K (33899G>A 33901G>A), W228S (33906G>C), N230A (33911A>G 33912A>C 33913C>G), E233D (33922G>C), E234R (33923G>C 33924A>G 33925G>A), V236I (33929G>A), V237I (33932G>A 33934T>C), G241T (33944G>A 33945G>C), T242G (33947A>G 33948C>G 33949G>C), K244Q (33953A>C), D246T (33959G>A 33960A>C 33961T>G), T292P (34097A>C), G297E (34113G>A 34114G>A), Y299N (34118T>A 34120T>C), E302V (34128A>T), D305Q (34136G>C 34138C>A), T306V (34139A>G 34140C>T 34141G>A), D308G (34146A>G), I309V (34148A>G 34150A>G), T310E (34151A>G 34152C>A 34153G>A), D312E (34159C>A), S313P (34160T>C 34162G>C), S318G (34175T>G 34176C>G 34177G>C), E319D (34180G>C), V321R (34184G>C 34185T>G)								

*: Inserts / Deletes / Misaligned / Frameshifts

Detailed Results of Halovirus HSTV-1 (NC_021471.1)

NGS details

Assembly

??coverage-details?? ??variant-analysis??

Assignment

Alignment

Genome Region

Sequence starts at position null and ends at position null relative to the null reference sequence for null.

Alignment Detailed Statistics

	Begin
**NT**	45
**CDS**	
1	16
3	68
13	52
14	1
36	99
37	1
45	151
52	120

	Begin
53	14
**Proteins**	
terminase large subunit (YP_008083051.1)	16
	L20Q (58T>C 59T>A 60A>G), R21E (61C>G 62G>A 63C>G), F23L (67T>C 69T>C), L26V (76C>G 78G>C), E27D (81G>C), Q30E (88C>G), F31H (91T>C 92T>A), L33V (97C>G), V35A (104<br>(392G>A 393G>C), N132S (394A>T 395A>C), S137A (409T>G 411G>C), N140G (418A>G 419A>G), I142V (424A>G 426C>G), Y143W (428A>G 429C>G), C144M (430T>A 431G>T 432C><br>(692C>A 693C>G), K232R (694A>C 695A>G), L233F (697C>T), R234K (700C>A 701G>A 702C>G), Q236K (706C>A), F237Y (710T>A), S241A (721A>G 722G>C 723T>A), R242K (724C>A<br>(1131A>C), Q381K (1141C>A), D382G (1145A>G 1146C>A), V387L (1159G>C 1161A>G), I388V (1162A>G 1164C>A), A389S (1165G>T), D390N (1168G>A), S391R (1171T>C 1172C>G 1
portal (YP_008083053.1)	68
	D75E (1971C>G), P77V (1975C>G 1976C>T 1977A>C), T84D (1996A>G 1997C>A 1998A>T), A85P (1999G>C), E86S (2002G>T 2003A>C 2004A>C), K90Q (2014A>C), M93I (2025G>C), Q<br>2283G>C), S180T (2284T>A 2286T>G), I181V (2287A>G), Q184N (2296C>A 2298A>C), D185_D186insG (2301_2302insGGG), D186N (2302G>A), R187A (2305A>G 2306G>C 2307G>C), N<br>2567A>G 2568C>G), A275S (2569G>A 2570C>G), T276V (2572A>G 2573C>T 2574G>C), R277K (2575C>A 2576G>A), R279V (2581C>G 2582G>T 2583G>C), Q282K (2590C>A), V283P (2<br>(3961_3962insATCGAGTTTGACTCGGAAGAAGTGATGTGGCAGGACATTCACAAGCGCTCGAACCGGCGATATGGCTTCCCGCCGACGCTGTGGTGTCGGGAGTTCCTGCGGTTGCTTGAC<br>T740Q (3967A>C 3968C>A), I741V (3970A>G 3972C>G), D743E (3978C>G), G746A (3986G>C), E751D (4002G>C), N754G (4009A>G 4010A>G 4011C>T), R756T (4015C>A 4016G>C 40
head maturation protease (YP_008083063.1)	52
	E53Q (8307G>C 8309A>G), D54R (8310G>C 8311A>G 8312T>C), A56K (8316G>A 8317C>A 8318C>G), V59I (8325G>A 8327G>C), L66I (8346C>A), V67L (8349G>C 8351C>G), N71D (83<br>L189V (8715C>G), V191I (8721G>A), G194E (8731G>A 8732C>A), A195P (8733G>C 8735G>C), D196E (8738T>G), A197T (8739G>A 8741A>G), S199Q (8745T>C 8746C>A 8747T>G), I2
capsid protein (YP_008083064.1)	1
	S2A (9578T>G), I3T (9582T>C 9583C>A), G4A (9585G>C), V6S (9590G>A 9591T>G 9592C>T), T7F (9593A>T 9594C>T 9595A>C), R8G (9596C>G), D9G (9600A>G 9601C>A), G10R (960<br>(9857T>C 9858T>G 9859C>G), G96I (9860G>A 9861G>T 9862A>C), L97I (9863C>A 9865C>T), A98K (9866G>A 9867C>A 9868C>G), E99D (9871G>T), T100A (9872A>G), K102D (9878A><br>10032C>G), T153_D154insL (10033_10034insCTT), D154E (10036C>A), H155D (10037C>G), S157D (10043T>G 10044C>A), I158V (10046A>G 10048C>A), S159_E163del (10049_10063d<br>10324G>C), N251D (10325A>G 10327T>C), Q252G (10328C>G 10329A>G 10330G>C), I257V (10343A>G 10345C>T), G258A (10347G>C 10348G>C), D259N (10349G>A), A264T (10364C<br>10615C>T), R348E (10616C>G 10617G>A 10618C>G), A349S (10619G>T), D350E (10624C>G), G351_S352del (10625_10630delGGATCG), Y354Q (10634T>C 10636C>G), V357I (10643C
hypothetical protein (YP_008083086.1)	99
	N101D (22945A>G), S108R (22966A>C 22968T>G), E119D (23001G>C), T124S (23015C>G), S146Q (23080T>C 23081C>A 23082G>A), A148S (23086G>A 23087C>G 23088G>C), E150_V
hypothetical protein (YP_008083087.1)	1
MCM DNA helicase (YP_008083095.1)	151
	T163N (26682C>A 26683A>C), R164Y (26684C>T 26685G>A), D169R (26699G>A 26700A>G 26701C>A), D171E (26707C>A), H172Y (26708C>T), Q182E (26738C>G 26740A>G), Q184K
adenine methyltransferase (YP_008083102.1)	120
	S122A (30862A>G 30863G>C 30864T>G), Y124K (30868T>A 30870C>G), S125T (30872G>C), D133E (30897C>A), T150N (30947C>A 30948G>C), S151A (30949T>G), R152T (30952C>A
hypothetical protein (YP_008083103.1)	14
	I14V (31771A>G), T18K (31784C>A 31785G>A), E21N (31792G>A 31794G>T), N26V (31807A>G 31808A>T 31809C>G), I32E (31825A>G 31826T>A 31827T>G), R38E (31843C>G 31844G

*: Inserts / Deletes / Misaligned / Frameshifts

Detailed Results of Archaeal BJ1 virus (NC_008695.1)

NGS details

Assembly

**Coverage length**	12665 (24 contig(s))
**Est. depth of coverage**	35.3
**Est. number of reads**	5395
**Ambiguities**	0

??coverage-details?? ??variant-analysis??

Assignment

**Type**	Archaeal BJ1 virus (Taxonomy ID: 416419)
**Reference Genome**	NC_008695.1 (Length: 42271bp)
**Host(s)**	Halorubrum saccharovorum
**NT Identity (%)**	71.4917
**NT Quality**	0.81666

Alignment

**Alignment score**	10343 (NT) + 20867 (AA) = 31210
**Concordance (%)**	55.8209
**Alignment method**	Local, heuristic, nucleotide (BLASTN)

Genome Region

Sequence starts at position null and ends at position null relative to the null reference sequence for null.

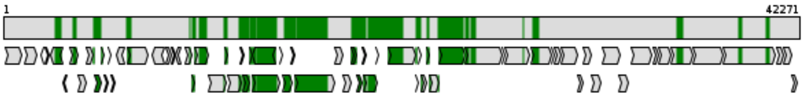

Alignment Detailed Statistics

	Begin	End	Coverage	Score	Concordance	Matches	Identities	I/D/M/F*	Stop Codons
**NT**	2767	40683	30.0%	10343.0	42.2%	12500 (97.3%)	9063 (70.6%)	177/165	
**CDS**									
BJ1_gp05	36	142	49.3%	679.0	88.8%	107 (100%)	94 (87.9%)	0/0/0/0	0
BJ1_gp07	47	113	45.0%	425.0	88.0%	67 (97.1%)	58 (84.1%)	2/0/0/0	0
BJ1_gp09	121	132	9.1%	57.0	64.0%	12 (100%)	8 (66.7%)	0/0/0/0	1
BJ1_gp10	1	125	100%	583.0	68.3%	123 (98.4%)	86 (68.8%)	0/2/0/0	2

	Begin	End	Coverage	Score	Concordance	Matches	Identities	I/D/M/F*	Stop Codons
BJ1_gp11	1	6	12.0%	33.0	78.6%	6 (100%)	5 (83.3%)	0/0/0/0	0
BJ1_gp16	1	59	16.4%	309.0	77.8%	59 (100%)	45 (76.3%)	0/0/0/0	0
BJ1_gp21	81	119	32.2%	153.0	61.2%	39 (100%)	22 (56.4%)	0/0/0/0	0
BJ1_gp22	17	44	63.6%	87.0	47.5%	28 (80.0%)	16 (45.7%)	7/0/0/0	1
BJ1_gp23	1	229	80.1%	1156.0	84.7%	196 (99.5%)	161 (81.7%)	0/1/0/0	0
BJ1_gp24	286	309	7.8%	134.0	72.8%	24 (100%)	17 (70.8%)	0/0/0/0	1
BJ1_gp25	1	46	95.8%	235.0	80.8%	46 (97.9%)	36 (76.6%)	1/0/0/0	0
BJ1_gp26	183	242	24.8%	185.0	47.7%	59 (98.3%)	29 (48.3%)	0/1/0/0	1
BJ1_gp27	1	31	100%	37.0	21.9%	30 (93.8%)	7 (21.9%)	1/1/0/0	1
BJ1_gp28	1	112	100%	417.0	55.3%	110 (95.7%)	61 (53.0%)	3/2/0/0	0
BJ1_gp29	12	57	80.7%	225.0	66.4%	46 (100%)	28 (60.9%)	0/0/0/0	1
BJ1_gp30	1	430	88.0%	1657.0	57.9%	422 (99.5%)	247 (58.3%)	0/2/0/0	0
BJ1_gp31	1	406	98.5%	2350.0	83.0%	400 (100%)	315 (78.8%)	0/0/0/0	1
BJ1_gp33	42	163	74.8%	625.0	76.5%	122 (100%)	93 (76.2%)	0/0/0/0	1
BJ1_gp34	1	74	100%	468.0	82.1%	74 (100%)	62 (83.8%)	0/0/0/0	2
BJ1_gp35	1	598	84.8%	2349.0	57.2%	596 (99.3%)	341 (56.8%)	2/2/2/1	0
BJ1_gp38	6	102	95.1%	597.0	72.3%	97 (100%)	68 (70.1%)	0/0/0/0	1
BJ1_gp39	1	87	100%	490.0	79.8%	87 (100%)	68 (78.2%)	0/0/0/0	0
BJ1_gp40	1	57	100%	63.0	16.8%	54 (87.1%)	17 (27.4%)	5/3/0/0	0
BJ1_gp41	1	226	91.2%	76.0	5.4%	199 (90.5%)	43 (19.5%)	14/7/0/0	5
BJ1_gp42	1	60	100%	36.0	11.2%	52 (86.7%)	13 (21.7%)	0/8/0/0	0
BJ1_gp43	1	493	55.4%	1547.0	82.8%	266 (97.4%)	215 (78.8%)	0/7/0/0	1
BJ1_gp44	1	53	100%	344.0	91.5%	53 (98.1%)	48 (88.9%)	1/0/0/0	1
BJ1_gp45	1	37	100%	181.0	56.2%	37 (94.9%)	22 (56.4%)	2/0/0/0	1
BJ1_gp46	1	2	1.9%	10.0	100%	2 (100%)	2 (100%)	0/0/0/0	0
BJ1_gp47	4	54	94.4%	427.0	93.0%	51 (100%)	46 (90.2%)	0/0/0/0	1
BJ1_gp48	1	170	18.8%	155.0	62.8%	32 (97.0%)	21 (63.6%)	1/0/0/0	1
BJ1_gp49	1	434	96.9%	1937.0	63.7%	417 (94.6%)	268 (60.8%)	7/17/0/0	0
BJ1_gp50	11	196	22.1%	815.0	79.5%	144 (100%)	110 (76.4%)	0/0/0/0	0
BJ1_gp52	304	346	9.6%	54.0	18.6%	43 (95.6%)	11 (24.4%)	2/0/0/0	0

	Begin	End	Coverage	Score	Concordance	Matches	Identities	I/D/M/F*	Stop Codons
BJ1_gp53	19	149	36.3%	602.0	67.6%	131 (100%)	87 (66.4%)	0/0/0/0	0
BJ1_gp65	90	211	30.8%	591.0	70.8%	122 (100%)	80 (65.6%)	0/0/0/0	0
BJ1_gp67	260	819	15.7%	778.0	75.3%	144 (100%)	104 (72.2%)	0/0/0/0	0
**Proteins**									
J domain-containing protein (YP_919032.1)	36	142	49.3%	679.0	88.8%	107 (100%)	94 (87.9%)	0/0/0/0	0
	E41Q (3071C>G), V43I (3065C>T), A52S (3038C>A), G54D (3031C>T), E56R (3024C>A 3025T>C 3026C>G), L71P (2979C>T 2980A>G), R91E (2919T>C 2920C>T 2921G>C), E95A (2907C>G 2908T>G), L108R (2868C>A 2869A>C), S113A (2855A>C), R119K (2835C>T 2836C>T 2837G>T), R126K (2814G>C 2815C>T 2816G>T)								
hypothetical protein (YP_919034.1)	47	113	45.0%	425.0	88.0%	67 (97.1%)	58 (84.1%)	2/0/0/0	0
	S48E (3688A>G 3689G>A 3690C>G), V55I (3709G>A), P57E (3715C>G 3716C>A 3717C>G), P57_A58insQI (3717_3718insCAGATC), E59L (3721G>C 3722A>T 3723G>C), D75N (3769G>A), E80D (3786G>C), V89I (3811G>A 3813G>C), A108E (3869C>A 3870C>G), D109G (3872A>G)								
hypothetical protein (YP_919036.1)	121	132	9.1%	57.0	64.0%	12 (100%)	8 (66.7%)	0/0/0/0	1
	G124A (4826G>C), D125E (4830C>G), M126G (4831A>G 4832T>G 4833G>A), E127G (4835A>G 4836G>T)								
hypothetical protein (YP_919037.1)	1	125	100%	583.0	68.3%	123 (98.4%)	86 (68.8%)	0/2/0/0	2
	R3E (4850A>G 4851G>A 4852A>G), P6R (4860C>G), G9A (4869G>C), E10G (4872A>G 4873G>C), R11D (4874C>G 4875G>A 4876G>C), E13G (4881A>G 4882A>G), G14K (4883G>A 4884G>A 4885A>G), S23T (4910T>A 4912G>C), N29D (4928A>G), Q30S (4931C>T 4932A>C), T31L (4934A>C 4935C>T 4936G>C), F32D (4937T>G 4938T>A), N33H (4940A>C), E34D (4945G>C), S44T (4973T>A), T50R (4991A>C 4992C>G), A67G (5043C>G 5044G>A), P68_D69del (5045_5050delCCCGAC), E70A (5052A>C), G71R (5054G>C 5056C>G), R75Q (5066A>C 5067G>A 5068A>G), L76R (5070T>G 5071C>T), A79D (5079C>A 5080G>C), F80Y (5082T>A), E84A (5094A>C 5095A>C), R85V (5096C>G 5097G>T), I92N (5118T>A), E98D (5137G>C), A105S (5156G>T 5158G>A), D107S (5162G>A 5163A>G), L109R (5169T>G 5170T>C), V111A (5175T>C 5176A>G), N112T (5178A>C 5179T>G), M113P (5180A>C 5181T>C), Q114D (5183C>G 5185A>C), R116Q (5190G>A), D121T (5204G>A 5205A>C), R124* (5213C>T)								
hypothetical protein (YP_919038.1)	1	6	12.0%	33.0	78.6%	6 (100%)	5 (83.3%)	0/0/0/0	0
AAA family ATPase (YP_919043.1)	1	59	16.4%	309.0	77.8%	59 (100%)	45 (76.3%)	0/0/0/0	0
	Q4N (6646C>A 6648G>T), I7V (6655A>G 6657C>A), L9R (6662T>G 6663C>G), D11E (6669C>A), Q15R (6680A>G 6681G>A), T16D (6682A>G 6683C>A 6684G>T), P22S (6700C>A 6701C>G), I24L (6706A>C 6708C>G), F27V (6715T>G), E38A (6749A>C), D41E (6759C>G), V43M (6763G>A 6765C>G), T57A (6805A>G)								
hypothetical protein (YP_919048.1)	81	119	32.2%	153.0	61.2%	39 (100%)	22 (56.4%)	0/0/0/0	0
	L86F (9915C>T), A88E (9922C>A), S90G (9927A>G 9929C>T), L92I (9933C>A), K94D (9939A>G 9941G>C), N98C (9951A>T 9952A>G), A100T (9957G>A 9959G>C), E103D (9968G>C), L104E (9969C>G 9970T>A), A105G (9973C>G 9974T>G), A106Y (9975G>T 9976C>A 9977G>C), L108V (9981C>G 9983G>C), D110Q (9987G>C 9989C>G), Q111R (9991A>G), R113N (9996C>A 9997G>A), A114E (10000C>A)								
hypothetical protein (YP_919049.1)	17	44	63.6%	87.0	47.5%	28 (80.0%)	16 (45.7%)	7/0/0/0	1
	D18E (10075C>G), T22N (10086C>A 10087G>C), A24S (10091G>A 10092C>G), S32A (10115A>G 10116G>C 10117T>G), A33S (10118G>T), Y35H (10124T>C), P36T (10127C>A), V37_D38insL (10132_10133insCTC), L39I (10136C>A), T40D (10139A>G 10140C>A 10141G>C), E41Q (10142G>C), A42V (10146C>T 10147C>G), A42_R43insSEQEVS (10147_10148insAGCGAGCAGGAGGTGTCG), R43G (10148C>G 10150G>C)								
hypothetical protein (YP_919050.1)	1	229	80.1%	1156.0	84.7%	196 (99.5%)	161 (81.7%)	0/1/0/0	0
	A9T (10177G>A), H21R (10214A>G 10215C>G), I22L (10216A>C), V32A (10247T>C), R37A (10261C>G 10262G>C 10263C>G), T41S (10273A>T 10275C>G), V43I (10279G>A), T86S (10408A>T 10410C>G), L90V (10420C>G), V107T (10471G>A 10472T>C 10473G>C), K108N (10476G>C), L111H (10484T>A 10485G>C), I119V (10507A>G), S124A (10522A>G 10523G>C 10524C>G), N131S (10543A>T 10544A>C 10545C>G), T137V (10561A>G 10562C>T), Q139R (10568A>G), S152A (10606T>G), K155Q (10615A>C), N161H (10633A>C), A163S (10639G>T), E164D (10644G>C), G166Q (10648G>C 10649G>A 10650C>G), G167K (10651G>A 10652G>A), Q170E (10660C>G), E177R (10681G>C 10682A>G), F179del (10687_10689delTTC), W180Q (10690T>C 10691G>A), N181K (10695C>A), T185R (10705A>C 10706C>G 10707C>T), E199D (10749A>C), G205E (10766G>A 10767C>G), T206Q (10768A>C 10769C>A), A209E (10778C>A)								
hypothetical protein (YP_919051.1)	286	309	7.8%	134.0	72.8%	24 (100%)	17 (70.8%)	0/0/0/0	1
	Q286E (11735C>G), E287A (11739A>C), A289E (11745C>A 11746A>G), A291T (11750G>A 11752G>T), T307A (11798A>G 11800G>C), A308* (11801G>T 11802C>G 11803G>A), *309C (11806A>T)								
hypothetical protein (YP_919052.1)	1	46	95.8%	235.0	80.8%	46 (97.9%)	36 (76.6%)	1/0/0/0	0
	V1M (11803G>A), S2_E3insA (11808_11809insGCT), T19S (11857A>T), E25D (11877G>C), D31E (11895T>G), N35S (11906A>G), L37V (11911C>G), E39D (11919G>C), L40I (11920C>A 11922G>C), A42E (11927C>A 11928G>A)								
hypothetical protein (YP_919053.1)	183	242	24.8%	185.0	47.7%	59 (98.3%)	29 (48.3%)	0/1/0/0	1
	V186L (12501G>C), D188V (12508A>T 12509T>C), P189E (12510C>G 12511C>A), D191A (12517A>C 12518C>A), T192E (12519A>G 12520C>A), L196A (12531C>G 12532T>C), S197G (12534A>G), V198C (12537G>T 12538T>G 12539G>T), R200E (12543C>G 12544G>A 12545C>G), I205L (12558A>C), A208del (12567_12569delGCT), D209S (12570G>A 12571A>G), E210T (12573G>A 12574A>C), R211M (12576C>A 12577G>T), L212T (12579C>A 12580T>C 12581C>A), T214S (12585A>T), S215R (12590T>G), D216Q (12591G>C 12593T>G), G217R (12594G>C), T219N (12601C>A 12602G>T), S221P (12606T>C), L224I (12615C>A), V225A (12619T>C), I226L (12621A>C 12623C>T), V229L (12630G>C 12632G>T), G233D (12643G>A 12644G>T), R234V (12645C>G 12646G>T), N235T (12649A>C), T237E (12654A>G 12655C>A), D240E (12665T>G), A241L (12666G>C 12667C>T)								

	Begin	End	Coverage	Score	Concordance	Matches	Identities	I/D/M/F*	Stop Codons
hypothetical protein (YP_919054.1)	1	31	100%	37.0	21.9%	30 (93.8%)	7 (21.9%)	1/1/0/0	1
	S2T (12672G>C 12673T>G), W3F (12675G>T 12676G>C), T4P (12677A>C 12679C>A), F5del (12680_12682delTTC), E6P (12683G>C 12684A>C), F7R (12686T>A 12687T>G 12688T>G), L8P (12690T>C), A9V (12693C>T), R10L (12696G>T), L12R (12702T>G 12703G>A), T13L (12704A>C 12705C>T 12706A>C), A14F (12707G>T 12708C>T), G15W (12710G>T 12712A>G), G15_L16insS (12712_12713insAGC), L16V (12713C>G), A17V (12717C>T), A18V (12720C>T), T19L (12722A>C 12723C>T), A21V (12729C>T), A22V (12732C>T 12733G>C), L23T (12734C>A 12735T>C), L24T (12737C>A 12738T>C), L25I (12740C>A), I29A (12752A>G 12753T>C), A30G (12756C>G)								
hypothetical protein (YP_919055.1)	1	112	100%	417.0	55.3%	110 (95.7%)	61 (53.0%)	3/2/0/0	0
	T3I (12764C>T), Q4D (12766C>G 12768G>C), R5T (12769C>A 12770G>C), T6V (12772A>G 12773C>T), R7D (12775C>G 12776G>A 12777G>C), Y8R (12778T>C 12779A>G), T9I (12782C>T 12783G>T), E11P (12787G>C 12788A>C 12789G>A), H12_A13insC (12792_12793insTGT), E15R (12799G>C 12800A>G), F17_R18del (12805_12810delTTCCGT), I19V (12811A>G), R20D (12814C>G 12815G>A 12816A>C), A21R (12817G>C 12818C>G), A23S (12823G>A 12824C>G), A25E (12830C>A 12831C>G), A26I (12832G>A 12833C>T 12834T>C), A28S (12838G>T 12840A>G), P29A (12841C>G), A30_W31insVA (12846_12847insGTCGCC), W31Y (12848G>A 12849G>C), L35V (12859C>G 12861C>T), R36T (12862C>A 12863G>C 12864T>C), N38A (12868A>G 12869A>C 12870C>G), A41T (12877G>A 12879C>G), E42T (12880G>A 12881A>C 12882G>C), R47K (12895C>A 12896G>A 12897A>G), A49D (12902C>A 12903T>C), R52V (12910C>G 12911G>T 12912G>C), A55E (12920C>A 12921A>G), R56Q (12923G>A 12924C>G), S57T (12925T>A 12927G>T), L59T (12931C>A 12932T>C 12933C>G), E65D (12951G>T), G66R (12952G>C), T67A (12955A>G), T69V (12961A>G 12962C>T), C71V (12967T>G 12968G>T 12969C>G), D75S (12979G>T 12980A>C 12981C>G), A76P (12982G>C 12984C>G), E77A (12986A>C), I79L (12991A>C 12993C>G), H83L (13004A>T), V85I (13009G>A 13011G>C), T91V (13027A>G 13028C>T), A93E (13034C>A 13035A>G), F95C (13040T>G 13041C>T), G98E (13049G>A 13050C>G), S101A (13057T>G 13059C>G), R102Q (13061G>A 13062T>G), A106R (13072G>C 13073C>G)								
hypothetical protein (YP_919056.1)	12	57	80.7%	225.0	66.4%	46 (100%)	28 (60.9%)	0/0/0/0	1
	A15V (13135C>T), D17E (13142T>A), R18Q (13144G>A 13145C>G), A20E (13150C>A 13151G>A), D21A (13153A>C 13154C>A), E22K (13155G>A), L25V (13164C>G), T28M (13174C>T), V30I (13179G>A), D31E (13184C>A), G33D (13189G>A), Y36F (13198A>T 13199C>T), I43V (13218A>G 13220C>G), V46A (13228T>C 13229G>A), R50H (13240G>A 13241C>T), Q51K (13242C>A), E52R (13245G>C 13246A>G), D54E (13253C>G)								
hypothetical protein (YP_919057.1)	1	430	88.0%	1657.0	57.9%	422 (99.5%)	247 (58.3%)	0/2/0/0	0
	P2L (13259C>T), T4_A5del (13264_13269delACCGCG), S6L (13271C>T), T8A (13276A>G), G9R (13279G>C), L11T (13285C>A 13286T>C 13287C>G), K13E (13291A>G), R15L (13298G>T), E16K (13300G>A), D17N (13303G>A), E22K (13318G>A), G23R (13321G>C), V24I (13324G>A), F27L (13333T>C), D28K (13336G>A 13338C>G), T30L (13342A>C 13343C>T 13344G>T), V33I (13351G>A), L34I (13354C>A), L35T (13357C>A 13358T>C 13359T>C), V36I (13360G>A), E37K (13363G>A), L42P (13379T>C), E44K (13384G>A 13386A>G), P45A (13387C>G), F47I (13393T>A), T48S (13396A>T), R49K (13399C>A 13400G>A 13401T>G), L50F (13402C>T), F51L (13405T>C), G52R (13408G>C 13410T>C), G55C (13417G>T), F56L (13420T>C), R58V (13426C>G 13427G>T 13428T>C), R60C (13432C>T), T63A (13441A>G), T66A (13450A>G), S67A (13453T>G), V76L (13480G>C), T77A (13483A>G), A80T (13492G>A), L82A (13498C>G 13499T>C), G89V (13520G>T), V91L (13525G>C), D94N (13534G>A), V102A (13559T>C 13560C>G), G104V (13565G>T 13566A>T), G105R (13567G>C 13569A>C), V107P (13573G>C 13574T>C), S110G (13582A>G), V112I (13588G>A), A113P (13591G>C), S114G (13594A>G), C115S (13597T>A), I117V (13603A>G), A123P (13621G>C), V125M (13627G>A), D128E (13638T>G), S136R (13660T>A 13661C>G 13662G>A), S137G (13663A>G), I139V (13669A>G), A143G (13682C>G 13683T>A), H157E (13723C>G 13725T>A), R159G (13729C>G), A161P (13735G>C 13737T>G), A173P (13771G>C), G174A (13775G>C 13776A>T), R178G (13786C>G), R179A (13789A>G 13790G>C 13791A>T), E181D (13797G>T), P182A (13798C>G), E185Q (13807G>C), V186I (13810G>A), V188A (13817T>C 13818C>T), T189A (13819A>G 13821G>C), V190R (13822G>C 13823T>G 13824C>A), C203D (13861T>G 13862G>A 13863T>C), A211S (13885G>T), Q223E (13921C>G), A224S (13924G>T), V225E (13928T>A), V226L (13930G>C), G227S (13933G>T 13934G>C), F228L (13938C>G), G229S (13939G>A), D230E (13944T>A), R232V (13948C>G 13949G>T 13950A>T), E235Q (13957G>C), Q238E (13966C>G), L239F (13969C>T), A242S (13978G>T), G243R (13981G>C), G250R (14002G>C), K252E (14008A>G), C253R (14011T>C), L254F (14014C>T), V255I (14017G>A), A256P (14020G>C), N257D (14023A>G), A259R (14029G>C 14030C>G 14031T>A), R260G (14032C>G 14034A>C), G261L (14035G>C 14036G>T), V262A (14039T>C 14040C>G), H267D (14053C>G), M268V (14056A>G), L269F (14059C>T 14061T>C), L270V (14062C>G 14064G>C), S272A (14068T>G), E273V (14072A>T), T276A (14080A>G), R277G (14083A>G), V278F (14086G>T), V283I (14101G>A), I285T (14108T>C), S289R (14119A>C 14121C>A), G290R (14122G>C), D294E (14136T>G), V296L (14140G>C), R297G (14143C>G), F300V (14152T>G), A301D (14156C>A 14157T>C), R303E (14161C>G 14162G>A 14163T>G), E308K (14176G>A), G309R (14179G>C), A312T (14188G>A), I316A (14200A>G 14201T>C), P317A (14203C>G), G319R (14209G>C), V323L (14221G>C), P326T (14230C>A), L327V (14233C>G 14235G>T), D332N (14248G>A), G335S (14257G>A 14259T>C), R336G (14260C>G), L337R (14264T>G 14265C>T), T338L (14266A>T 14267C>T), L339F (14269C>T), K341E (14275A>G), S344G (14284A>G), T346P (14290A>C), P347A (14293C>G), G355A (14318G>C 14319C>T), T356S (14320A>T), N359D (14329A>G), R366G (14350C>G), E367D (14355G>T), P368L (14357C>T 14358G>C), G370P (14362G>C 14363G>C 14364C>G), Q373E (14371C>G), H375D (14377C>G), L376V (14380C>G), T377P (14383A>C), G379S (14389G>A), G383D (14402G>A), S385G (14407A>G), G389A (14420G>C 14421A>T), L391P (14426T>C), V392L (14428G>C), L394V (14434C>G), D399N (14449G>A), V402I (14458G>A), D403N (14461G>A), R404G (14464C>G), V405G (14468T>G), P406A (14470C>G), R407G (14473C>G), I409V (14479A>G), R413L (14492G>T 14493G>T), G414I (14494G>A 14495G>T), V415L (14497G>C), Y417P (14503T>C 14504A>C), W418S (14507G>C), A419P (14509G>C), H422Y (14518C>T 14520C>T), W425R (14527T>C)								
tyrosine-type recombinase/integrase (YP_919058.1)	1	406	98.5%	2350.0	83.0%	400 (100%)	315 (78.8%)	0/0/0/0	1
	N7T (14468T>G), E14Q (14448C>G), E18D (14434C>G), D20E (14428G>C), E21G (14426T>C), A29V (14402G>A), Q36H (14380C>G), M37I (14377C>G), A42R (14362G>C 14363G>C 14364C>G), R44E (14357C>T 14358G>C), L45I (14355G>T), K47E (14349T>C), A57S (14318G>C 14319C>T), N69D (14283T>C), R74Q (14266A>T 14267C>T), E75T (14264T>G 14265C>T), T77A (14257G>A 14259T>C), Q85N (14233C>G 14235G>T), D89E (14221G>C), D96G (14200A>G 14201T>C), T109L (14161C>G 14162G>A 14163T>G), R110G (14160G>C), S111V (14156C>A 14157T>C), E112D (14152T>G), P114T (14148G>T), D116E (14140G>C), S117T (14139A>T), I118L (14136T>G), A123S (14119A>C 14121C>A), N127S (14108T>C), D134E (14086G>T), F139Y (14072A>T), Q142D (14062C>G 14064G>C), K143E (14059C>T 14061T>C), V149I (14043C>T), D150R (14039T>C 14040C>G), A151E (14035G>C 14036G>T), S152A (14032C>G 14034A>C), S180N (13948C>G 13949G>T 13950A>T), N181D (13947T>C), I182F (13944T>A), E184Q (13938C>G), A185G (13933G>T 13934G>C), D186E (13930G>C), Y187F (13928T>A), T192S (13914T>A), T209V (13861T>G 13862G>A 13863T>C), D222S (13822G>C 13823T>G 13824C>A), R223G (13819A>G 13821G>C), D224S (13817T>C 13818C>T), L231I (13797G>T), T245A (13755T>C), S251R (13735G>C 13737T>G), A254P (13728C>G), M255F (13723C>G 13725T>A), A259S (13713C>A), R276S (13660T>A 13661C>G 13662G>A), T282A (13644T>C), I284L (13638T>G), D305G (13573G>C 13574T>C), S307A (13567G>C 13569A>C), S308N (13565G>T 13566A>T), D310R (13559T>C 13560C>G), D321E (13525G>C), I322V (13524T>C), T323N (13520G>T), S328T (13506A>T), Q330R (13498C>G 13499T>C), D336E (13480G>C), T354D (13426C>G 13427G>T 13428T>C), T360A (13408G>C 13410T>C), T363L (13399C>A 13400G>A 13401T>G), E365D (13393T>A), F368L (13384G>A 13386A>G), K370R (13379T>C), K377G (13357C>A 13358T>C 13359T>C), E378D (13354C>A), R382K (13342A>C 13343C>T 13344G>T), V384L (13336G>A 13338C>G), V386I (13332C>T), A397E (13298G>T), A398T (13296C>T), E401R (13285C>A 13286T>C 13287C>G), V402I (13284C>T)								
hypothetical protein (YP_919060.1)	42	163	74.8%	625.0	76.5%	122 (100%)	93 (76.2%)	0/0/0/0	1
	E46D (14956A>C), D47E (14959C>G), A50S (14966G>T 14968C>G), H58F (14990C>T 14991A>T), D60E (14998C>G), N61D (14999A>G 15001T>C), D63G (15006A>G), A65K (15011G>A 15012C>A), Q66E (15014C>G), D70A (15027A>C), K72R (15032A>C 15033A>G 15034G>A), M75L (15041A>C), Q82S (15062C>T 15063A>C), R86S (15074C>A 15076T>C), G87R (15077G>C 15079C>G), S88T (15080T>A 15082C>T), V90T (15086G>A 15087T>C), M99K (15114T>A), H111Y (15149C>T), K117D (15167A>G 15169G>T), E126D (15196A>C), K128R (15200A>C 15201A>G), P131A (15209C>G), S132G (15212A>G), D140N (15236G>A), R142Q (15243G>A 15244C>G), V149A (15264T>C), A157T (15287G>A), S158T (15290T>A)								
hypothetical protein (YP_919061.1)	1	74	100%	468.0	82.1%	74 (100%)	62 (83.8%)	0/0/0/0	2
	S2T (15313T>A), T5S (15323C>G), P15A (15352C>G 15354T>A), E16V (15356A>T), P18* (15361C>T 15362C>A), K20N (15369G>T), A31T (15400G>A 15402A>T), R32G (15403A>G), N50D (15457A>G), P62Q (15494C>A), P63Q (15497C>A), P64A (15499C>G)								
hypothetical protein (YP_919062.1)	1	598	84.8%	2349.0	57.2%	596 (99.3%)	341 (56.8%)	2/2/2/1	0

	Begin	End	Coverage	Score	Concordance	Matches	Identities	I/D/M/F*	Stop Codons
	T4S (15499C>G), T19I (15544C>T), L22Y (15552C>T 15553T>A), E24N (15558G>A 15560G>C), R25Y (15561C>T 15562G>A 15563G>C), A29E (15574C>A 15575A>G), V30I (15576G>A), T32E (15582A>G 15583C>A), H36K (15594C>A 15596C>G), V45Y (15621G>T 15622T>A 15623G>C), I46V (15624A>G), E50D (15638G>T), E53P (15645G>C 15646A>C), R56P (15655G>C), E57D (15659G>T), I58L (15660A>C), T62F (15672A>T 15673C>T), L63R (15676T>G), S64T (15678T>A 15680G>C), E67D (15689G>C), E68Q (15690G>C), G70R (15696G>C), L73A (15705C>G 15706T>C), Q79L (15724A>T), F80Y (15727T>A), D87K (15747G>A 15749C>A), A89G (15754C>G 15755G>C), N90H (15756A>C), S96T (15774T>A), P100R (15787C>G 15788C>A), E101D (15791G>C), H102D (15792C>G 15794C>T), Y104T (15798T>A 15799A>C 15800C>A), Y105S (15802A>C), P106A (15804C>G 15806T>G), G107A (15808G>C 15809T>A), G108F (15810G>T 15811G>T 15812G>C), F109A (15813T>G 15814T>C 15815C>G), P111T (15819C>A), Q114H (15830G>T), A115I (15831G>A 15832C>T), S117K (15838G>A 15839T>A), H118L (15841A>T), R119V (15843C>G 15844G>T 15845G>C), A120S (15846G>T 15848G>C), I121V (15849A>G), T122Q (15852A>C 15853C>A 15854C>A), E124I (15858G>A 15859A>T 15860G>T), S126R (15864T>C 15865C>G 15866G>C), Y132R (15882T>C 15883A>G), S133P (15885T>C), R134K (15888C>A 15889G>A 15890T>G), V136T (15894G>A 15895T>C), E137D (15899G>T), K143Q (15915A>C 15917A>G), F148M (15930T>A 15932C>G), S149T (15934G>C 15935C>G), T154S (15949C>G 15950T>C), A185S (16041G>T), Q187K (16047C>A), I190V (16056A>G 16058T>C), A191Q (16059G>C 16060C>A), T192E (16062A>G 16063C>A), P193S (16065C>T 16067A>G), E195_I196insG (16073_16074insGGG), I196S (16074A>T 16075T>C), S198A (16080T>G 16082A>G), G199V (16084G>T 16085C>G), T200R (16087C>G 16088T>A), G201R (16089G>C), H202Q (16094T>G), I204L (16098A>C), T207D (16107A>G 16108C>A), A208I (16110G>A 16111C>T), L212I (16122C>A), A213T (16125G>A), D214G (16129A>G), L215K (16131C>A 16132T>A 16133A>G), V218P (16140G>C 16141T>C), R221H (16150G>A 16151T>C), I224V (16158A>G), T225A (16161A>G 16163G>C), T227V (16167A>G 16168C>T 16169A>G), I228L (16170A>C), L230I (16176C>A 16178A>C), D231E (16181C>G), K233M (16186A>T), T234S (16188A>T), S235K (16192G>A 16193C>A), R237N (16197C>A 16198G>A 16199G>C), T240S (16206A>T 16208G>C), G241S (16209G>T 16210G>C 16211A>G), K242L (16212A>T 16213A>T 16214A>G), E244D (16220G>T), A245L (16221G>C 16222C>T 16223G>C), V247M (16227G>A 16229C>G), A249G (16234C>G 16235C>T), G250V (16237G>T 16238T>C), A251S (16239G>T), T253K (16246C>A 16247G>A), V254I (16248G>A), Q256D (16254C>G 16256G>C), T257E (16257A>G 16258C>A 16259C>G), D258E (16262C>G), H259F (16263C>T 16264A>T), T260E (16266A>G 16267C>A 16268C>G), I262M (16274C>G), S265T (16281T>A 16283G>C), A266D (16285C>A), E267D (16289G>C), E268D (16292G>T), R269V (16293C>G 16294G>T), E270A (16297A>C 16298G>C), R271E (16299C>G 16300G>A 16301C>A), H273I (16305C>A 16306A>T), D277K (16317G>A 16319C>G), G278E (16321G>A 16322C>A), A279P (16323G>C), E280D (16328G>T), G281I (16329G>A 16330G>T 16331C>T), T282Y (16332A>T 16333C>A 16334A>T), P283E (16335C>G 16336C>A), I284K (16339T>A 16340C>A), D285M (16341G>A 16342A>T 16343C>G), A288_E289del (16350_16355delGCCGAG), L291V (16359C>G), K294S (16368A>T 16369A>C 16370A>G), V295I (16371G>A 16373G>C), N298Y (16380A>T), I301E (16389A>G 16390T>A 16391T>G), R303L (16396G>T), N304A (16398A>G 16399A>C 16400C>G), L305M (16401C>A 16403T>G), R307L (16408G>T 16409A>G), A308Q (16410G>C 16411C>A 16412G>A), M309L (16413A>T), V310F (16416G>T 16418C>T), G311S (16419G>T 16420G>C 16421C>G), S313V (16425T>G 16426C>T), K314T (16429A>C 16430G>A), V315K (16431G>A 16432T>A 16433C>A), V316N (16434G>A 16435T>A 16436C>T), Y317L (16437T>C 16438A>T), G318P (16440G>C 16441G>C), D320G (16447A>G), D321S (16449G>T 16450A>C 16451C>A), H322R (16453A>G), D323I (16455G>A 16456A>T), E326D (16466G>C), F327L (16467T>C 16469C>T), I329M (16475C>G), A338G (16501C>G 16502G>A), K341Q (16509A>C), L342M (16512C>A 16514T>G), I343L (16515A>C 16517C>A), Q344S (16518C>T 16519A>C 16520G>A), R345Y (16521C>T 16522G>A 16523C>T), V346I (16524G>A 16526C>A), E347Q (16527G>C), E348N (16530G>A 16532G>T), L349I (16533C>A 16535C>T), G350A (16537G>C 16538C>T), W351P (16539T>C 16540G>C), T353S (16545A>T 16547T>G), G355Y (16551G>T 16552G>A), V356T (16554G>A 16555T>C), A361S (16569G>A 16570C>G 16571G>T), T362S (16572A>T), V363S (16575G>A 16576T>G 16577C>T), V366L (16584G>C 16586C>T), S369A (16593A>G 16594G>C), Q372R (16603A>G 16604G>C), E379Q (16623G>C), A380Q (16626G>C 16627C>A), A380_T381insW (16628_16629insTGG), K383E (16635A>G 16637A>G), F387L (16647T>C), K389L (16653A>T 16654A>T), H391D (16659C>G), A394I (16668G>A 16669C>T 16670G>C), V395A (16672T>C), C396A (16674T>G 16675G>C 16676C>T), I397V (16677A>G), D402K (16692G>A 16694T>G), P404R (16699C>G), A405P (16701G>C 16703A>G), D406E (16706C>G), V407D (16708T>A), A409S (16713G>A 16714C>G 16715A>T), L412H (16723T>A), D413E (16727C>G), P414A (16728C>G 16730C>G), M415L (16731A>C 16733G>C), S416E (16734T>G 16735C>A 16736G>A), K417Q (16737A>C 16739G>A), K419Q (16743A>C), H421S (16749C>A 16750A>G), V422I (16752G>A), N423S (16755A>T 16756A>C 16757C>G), W425A (16761T>G 16762G>C 16763G>C), M431L (16779A>C 16781G>T), R432K (16782C>A 16783G>A 16784G>A), T433S (16785A>T), E434R (16788G>C 16789A>G 16790A>G), A435C (16791G>T 16792C>G 16793C>T), A436S (16794G>T), V437L (16797G>T 16799T>G), V438L (16800G>C), A439G (16804C>G 16805C>G), H445Y (16821C>T), Q450R (16837A>G 16838G>C), D456E (16856C>A), F458I (16860T>A), S462P (16872A>C 16873G>C 16874C>G), N463A (16875A>G 16876A>C 16877T>G), L465I (16881C>A 16883G>T), L474V (16908C>G 16910G>C), S475T (16911T>A 16913G>C), T477Q (16917A>C 16918C>A), P480E (16926C>G 16927C>A), D481E (16931C>A), E482H (16932G>C 16934G>C), E484T (16938G>A 16939A>C 16940A>C), R485K (16941C>A 16942G>A 16943T>A), I486L (16944A>C), S487A (16947T>G), L491I (16959C>A), R492K (16962C>A 16963G>A 16964C>A), T502A (16992A>G 16994C>G), R504M (16998C>A 16999G>T 17000T>G), E505D (17003G>C), L506V (17004C>G), S507D (17007T>G 17008C>A), E508D (17012G>C), N509E (17013A>G 17015C>G), G510L (17016G>C 17017G>T 17018C>G), E512D (17024G>T), S515A (17031T>G), I522L (17052A>C), V527I (17067G>A), R532Q (17083G>A 17084C>G), E539A (17104A>C), S540N (17107G>A), E541D (17111G>C), K542E (17112A>G), F544N (17118T>A 17119T>A 17120C>T), Q548K (17130C>A 17132A>G), N552A (17142A>G 17143A>C), T553D (17145A>G 17146C>A 17147G>T), H558N (17160C>A), N561D (17169A>G), E562D (17174G>C), D563N (17175G>A), I577V (17217A>G), D583A (17236A>C), L586F (17244C>T 17246G>C)								
hypothetical protein (YP_919065.1)	6	102	95.1%	597.0	72.3%	97 (100%)	68 (70.1%)	0/0/0/0	1
	V15L (18554G>C), A18G (18564C>G 18565C>T), A20G (18570C>G 18571G>A), R26Q (18588G>A 18589C>G), K28V (18593A>G 18594A>T), R35Q (18615G>A 18616C>G), W36F (18618G>T 18619G>C), A38S (18623G>A 18624C>G 18625A>T), P40D (18629C>G 18630C>A 18631G>T), D50E (18661C>G), P55S (18674C>T), P58L (18684C>T 18685A>C), D59Q (18686G>C 18688C>G), T61K (18693C>A), D66N (18707G>A 18709T>C), N68D (18713A>G), A71R (18722G>A 18723C>G 18724G>A), D73N (18728G>A), V75L (18734G>C 18736C>G), M76I (18739G>C), T77I (18741C>T), S79T (18746T>A 18748A>G), G81A (18753G>C 18754C>G), E85D (18766G>T), C86R (18767T>C 18769C>T), R87H (18771G>A 18772G>T), T89G (18776A>G 18777C>G), S101* (18813C>A 18814G>A), *102C (18817A>T)								
MarR family transcriptional regulator (YP_919066.1)	1	87	100%	490.0	79.8%	87 (100%)	68 (78.2%)	0/0/0/0	0
	V1M (18814G>A), Q12R (18848A>G 18849G>C), T13S (18850A>T), E16K (18859G>A 18861A>G), R17Q (18863G>A), R19G (18868A>G 18870G>C), V30L (18901G>C 18903G>C), S37G (18922A>G 18924C>A), V49A (18959T>C 18960C>T), E52R (18967G>C 18968A>G), R53K (18970C>A 18971G>A 18972C>G), R56H (18980G>A), Q61T (18994C>A 18995A>C 18996G>A), D66G (19010A>G 19011C>G), Q79A (19048C>G 19049A>C 19050G>C), E83K (19060G>A 19062G>A), A84I (19063G>A 19064C>T 19065T>A), H85A (19066C>G 19067A>C), *87M (19072T>A 19073G>T 19074A>G)								
hypothetical protein (YP_919067.1)	1	57	100%	63.0	16.8%	54 (87.1%)	17 (27.4%)	5/3/0/0	0
	V1D (19072T>A 19073G>T), S2V (19074A>G 19075G>T), D3del (19077_19079delGAC), K4T (19081A>C), A6T (19086G>A 19088G>A), L7H (19090T>A), G8Y (19092G>T 19093G>A 19094G>C), R10W (19098C>T 19100A>G), L11Y (19101C>T 19102T>A), G13S (19107G>T 19108G>C), W14M (19110T>A 19111G>T), G18H (19122G>C 19123G>A 19124A>C), S19T (19125T>A), G20S (19128G>A), A25V (19144C>T), G27_D28insAAGCQ (19152_19153insCGGCAGGGTGCCAGA), D28N (19152_19153insCGGCAGGGTGCCAGA), L29Q (19156T>A 19157C>G), M30R (19159T>G), P31R (19162C>G), R32L (19165G>T), T33A (19167A>G), Q34L (19171A>T), G35M (19173G>A 19174G>T), S36T (19176T>A), V37A (19180T>C), R38P (19183G>C), Q39H (19187G>C), G40P (19188G>C 19189G>C 19190C>A), R41P (19192G>C), G44D (19201G>A 19202T>C), P46A (19206C>G 19208T>C), P48_F49del (19212_19217delCCTTTC), C50E (19218T>G 19219G>A 19220C>G), S52V (19224T>G 19225C>T 19226G>C), I53G (19227A>G 19228T>G 19229A>T), H54R (19231A>G), H55E (19233C>G 19235T>G), A56F (19236G>T 19237C>T 19238C>T), *57F (19240G>T 19241A>T)								
hypothetical protein (YP_919068.1)	1	226	91.2%	76.0	5.4%	199 (90.5%)	43 (19.5%)	14/7/0/0	5

	Begin	End	Coverage	Score	Concordance	Matches	Identities	I/D/M/F*	Stop Codons
	R6* (19144C>T), A8_T9insRQGAR (19152_19153insCGGCAGGGTGCCAGA), S10R (19156T>A 19157C>G), C11G (19159T>G), L12V (19162C>G), A13S (19165G>T 19167A>G), R15* (19171A>T 19173G>A), G16* (19174G>T 19176T>A), Y18H (19180T>C), D19H (19183G>C), R20T (19187G>C 19188G>C), A21H (19189G>C 19190C>A), G22R (19192G>C 19194A>C), T24M (19199C>T), V25T (19201G>A 19202T>C 19203C>A), A26G (19205C>G 19206C>G), L27P (19208T>C), G28_L29del (19211G>A 19212_19217delCCTTTC), S30E (19212_19217delCCTTTC 19218T>G), A31R (19219G>A 19220C>G), R33S (19225C>T 19226G>C 19227A>G), Y34V (19228T>G 19229A>T), I35V (19231A>G 19233C>G), M36S (19235T>G 19236G>T), P37F (19237C>T 19238C>T), E38L (19240G>T 19241A>T), A41H (19249G>C 19250C>A), H43A (19255C>G 19256A>C), H43_E44ins*A (19257_19258insTGAGCA), E44T (19258G>A 19259A>C 19260G>C), H45C (19261C>T 19262A>G), L47P (19268T>C 19269C>A), D48R (19270G>C 19271A>G 19272C>A), S50P (19276T>C 19278G>T), D51A (19280A>C 19281C>T), V52R (19282G>C 19283T>G 19284G>A), C53R (19285T>C), N54I (19289A>T), S55* (19292C>G 19293C>A), H57V (19297C>G 19298A>T 19299C>G), R58S (19300A>T 19301G>C 19302A>T), V59L (19303G>C), R82E (19372C>G 19373G>A 19374C>G), H83Y (19375C>T), R84E (19378C>G 19379G>A 19380C>G), D85T (19381G>A 19382A>C 19383T>C), T87H (19387A>C 19388C>A), V88A (19391T>C 19392A>G), V89R (19393G>C 19394T>G), D90H (19396G>C), Y91R (19399T>C 19400A>G), G92E (19403G>A), P93H (19406C>A 19407G>C), A94T (19408G>A), D95V (19412A>T 19413C>G), T96V (19414A>G 19415C>T), V97D (19418T>A), G98Y (19420G>T 19421G>A), E99G (19424A>G 19425A>C), Q100P (19427A>C 19428G>T), Q101A (19429C>G 19430A>C 19431A>C), G102D (19433G>A 19434G>C), T103S (19435A>T), F104V (19438T>G), C105T (19441T>A 19442G>C), A106D (19445C>A 19446T>C), R107Q (19448G>A 19449C>A), C108Q (19450T>C 19451G>A 19452C>G), E111F (19459G>T 19460A>T 19461G>T), S112C (19463C>G 19464G>C), P113T (19465C>A), W114_D115del (19468_19473delTGGGAT), R116H (19475G>A), Y117C (19478A>G), W118G (19480T>G), D119T (19483G>A 19484A>C 19485C>G), D120E (19488C>A), H121T (19489C>A 19490A>C), R122A (19492C>G 19493G>C), T123F (19495A>T 19496C>T), T125_H126del (19501_19506delACGCAC), E127R (19507G>C 19508A>G), A129W (19513G>T 19514C>G), T130N (19517C>A 19518G>C), A131D (19520C>A 19521G>C), L132H (19523T>A), G133del (19525_19527delGGC), W134R (19528T>C), L135T (19531C>A 19532T>C), D137A (19538A>C), V138H (19540G>C 19541T>A), D139E (19545C>G), E140I (19546G>A 19547A>T 19548G>C), P141A (19549C>G 19551T>G), V143A (19556T>C), T144L (19558A>C 19559C>T 19560T>C), P145G (19561C>G 19562C>G 19563G>C), C146W (19566C>G), Q147L (19568A>T 19569G>C), E148D (19572G>C), R149S (19573C>A), E150V (19577A>T 19578G>C), E150_V151insDKP (19578_19579insGACAAACCC), V151A (19580T>C), M153T (19586T>C), P154_K155insC (19590_19591insTGC), K155P (19591A>C 19592A>C), F156E (19594T>G 19595T>A 19596C>G), Q158E (19600C>G), L159I (19603C>A 19605G>C), L160S (19606C>T 19607T>C 19608C>G), K161T (19610A>C), E162A (19613A>C), T163K (19616C>A 19617C>G), I164F (19618A>T), T166D (19624A>G 19625C>A 19626G>T), L167_E168insI (19629_19630insATC), E168Q (19630G>C), E169A (19634A>C), K170T (19637A>C 19638A>T), G171I (19639G>A 19640G>T), V172R (19642G>C 19643T>G 19644C>G), H175E (19651C>G 19653C>G), R176H (19655G>A 19656T>C), E177K (19657G>A), E177_T178insGV (19659_19660insGGCGTC), A180D (19667C>A 19668C>T), E182Q (19672G>C), A183T (19675G>A), R185A (19681C>G 19682G>C 19683G>C), R187E (19687C>G 19688G>A 19689T>G), R188A (19690C>G 19691G>C 19692C>A), D189L (19693G>C 19694A>T 19695C>G), G190R (19696G>C 19698C>A), E191R (19699G>C 19700A>G 19701G>T), H192R (19703A>G 19704C>T), V193R (19705G>C 19706T>G), D195G (19712A>G), C196E (19714T>G 19715G>A 19716T>G), L197H (19718T>A), G198V (19721G>T), E199D (19725G>C), A200D (19727C>A 19728G>C), T201C (19729A>T 19730C>G), E202L (19732G>C 19733A>T 19734G>C), A203G (19736C>G 19737G>C), A204E (19739C>A 19740C>G), I205A (19741A>G 19742T>C 19743C>G), R206T (19745G>C 19746A>T), R207E (19747C>G 19748G>A 19749C>G), I209A (19753A>G 19754T>C), T210I (19757C>T), S212R (19762T>C 19763C>G), G213A (19766G>C), A214I (19768G>A 19769C>T), D215T (19771G>A 19772A>C 19773C>T), T217S (19777A>T), D218S (19780G>A 19781A>G), S219A (19783T>G 19785G>T), R220D (19786C>G 19787G>A 19788G>T), R221Q (19790G>A 19791C>A), E222K (19792G>A), V223T (19795G>A 19796T>C), P224R (19799C>G 19800G>C), A225R (19801G>C 19802C>G), *226E (19804T>G 19805G>A 19806A>G)								
hypothetical protein (YP_919069.1)	1	60	100%	36.0	11.2%	52 (86.7%)	13 (21.7%)	0/8/0/0	0
	M1R (19804T>G 19805G>A), S2G (19806A>G 19808C>T), V3L (19809G>C), Q5M (19815C>A 19816A>T), Y6del (19818_19820delTAC), N7S (19822A>G), R8A (19824C>G 19825G>C 19826T>C), V9S (19827G>T 19828T>C 19829C>A), A10Q (19830G>C 19831C>A), F11Y (19834T>A), F13R (19839T>C 19840T>G), G14V (19843G>T), G15A (19846G>C 19847T>G), R16F (19848C>T 19849G>T), E17N (19851G>A 19853G>C), L18F (19854C>T), E19A (19858A>C 19859G>C), D21R (19863G>C 19864A>G), A22E (19867C>A), I23L (19869A>C), D24E (19874C>G), F25G (19875T>G 19876T>G), E26D (19880G>C), P27del (19881_19883delCCC), G29I (19887G>A 19888G>T), V31F (19893G>T), S32del (19896_19898delTCC), G33E (19900G>A), D35A (19906A>C), L37_V40del (19911_19922delCTCCTGGTCGTC), D43S (19929G>T 19930A>C 19931C>T), L45P (19936T>C 19937C>G), T46N (19939C>A), Y47G (19941T>G 19942A>G 19943C>G), R48L (19945G>T 19946G>C), V49L (19947G>C), A50V (19951C>T 19952G>T), E51V (19954A>T 19955A>T), S52del (19956_19958delTCC), E55D (19967G>C), R56G (19968C>G 19970G>A), D58T (19974G>A 19975A>C 19976T>C), R59Y (19977C>T 19978G>A 19979A>C), *60R (19980T>C 19981A>G 19982G>A)								
hypothetical protein (YP_919070.1)	1	493	55.4%	1547.0	82.8%	266 (97.4%)	215 (78.8%)	0/7/0/0	1
	M1L (20365A>C 20367G>C), A2Q (20368G>C 20369C>A 20370C>G), T3M (20372C>T 20373A>G), S5D (20377T>G 20378C>A 20379A>C), D6N (20380G>A), L10F (20392C>T), D12E (20400C>G), H15R (20408A>G), D16E (20412T>G), L17F (20413C>T), A19D (20420C>A 20421G>C), M20A (20422A>G 20423T>C 20424G>A), T25S (20437A>T), S26Q (20440T>C 20441C>A 20442G>A), A27E (20444C>A), E30A (20453A>C), E31V (20456A>T), F32M (20458T>A 20460T>G), D33G (20462A>G 20463C>T), A35S (20467G>T 20469C>G), P36T (20470C>A 20472G>C), T37A (20473A>G 20475G>C), E40R (20482G>C 20483A>G), S41D (20485T>G 20486C>A 20487G>T), N44E (20494A>G 20496C>G), L46V (20500C>G 20502G>C), K47Q (20503A>C), D48E (20508C>G), K49H (20509A>C 20511G>C), G50del (20512_20514delGGC), P52D (20518C>G 20519C>A), R57E (20533C>G 20534G>A 20535C>G), E58T (20536G>A 20537A>C), N60H (20542A>C), E61V (20546A>T 20547G>C), Y63_G66del (20551_20562delTATGTCGACGGA), P68R (20567C>G), G69A (20570G>C 20571C>T), W70_Q71del (20572_20577delTGGCAG), A72D (20579C>A 20580G>T), A73D (20582C>A), A74S (20584G>T 20586C>T), A75E (20588C>A 20589C>G), K76E (20590A>G), D77P (20593G>C 20594A>C), D78S (20596G>T 20597A>C 20598T>A), Q79E (20599C>G 20601G>A), E80A (20603A>C), T83S (20611A>T), D121E (20727C>G), A122T (20728G>A 20730C>A), I221V (21025A>G), T262D (21148A>G 21149C>A 21150G>C)								
hypothetical protein (YP_919071.1)	1	53	100%	344.0	91.5%	53 (98.1%)	48 (88.9%)	1/0/0/0	1
	S8A (21861T>G), S8_H9insS (21863_21864insAGC), A34S (21939G>T), S36A (21945A>G 21946G>C 21947C>G), R37H (21949G>A 21950G>C), D44E (21971C>G)								
hypothetical protein (YP_919072.1)	1	37	100%	181.0	56.2%	37 (94.9%)	22 (56.4%)	2/0/0/0	1
	T2S (22005C>G), A3D (22008C>A), Q4T (22010C>A 22011A>C 22012A>C), D6P (22016G>C 22017A>C), T7I (22020C>T), N8P (22022A>C 22023A>C 22024C>A), D9S (22025G>T 22026A>C), D11Y (22031G>T), G12R (22034G>C), G12_R13insPN (22036_22037insCCGAAC), P20R (22059C>G), H21L (22062A>T 22063C>G), K25V (22073A>G 22074A>T 22075A>C), H29C (22085C>T 22086A>G), F32L (22096C>G), A33M (22097G>A 22098C>T 22099T>G)								
hypothetical protein (YP_919073.1)	1	2	1.9%	10.0	100%	2 (100%)	2 (100%)	0/0/0/0	0
hypothetical protein (YP_919074.1)	4	54	94.4%	427.0	93.0%	51 (100%)	46 (90.2%)	0/0/0/0	1
	D5E (22430C>G), D9G (22441A>G), E26D (22493G>C), T30A (22503A>G), T45P (22548A>C 22550T>G)								
hypothetical protein (YP_919075.1)	1	170	18.8%	155.0	62.8%	32 (97.0%)	21 (63.6%)	1/0/0/0	1
	P148S (23015C>A 23016C>G 23017A>C), V149I (23018G>A 23020G>C), E150D (23023A>C), E152T (23027G>A 23028A>C), H154N (23033C>A), V157I (23042G>A 23044T>C), N163E (23060A>G 23062C>A), D165G (23067A>G 23068C>T), T168D (23075A>G 23076C>A), T168_E169insD (23077_23078insGAC), E169A (23079A>C)								
hypothetical protein (YP_919076.1)	1	434	96.9%	1937.0	63.7%	417 (94.6%)	268 (60.8%)	7/17/0/0	0

	Begin	End	Coverage	Score	Concordance	Matches	Identities	I/D/M/F*	Stop Codons
	S2_A3insVAS (23085_23086insGTGGCGTCG), A3P (23086G>C), A5S (23092G>T 23094A>G), A32V (23174C>T), I54L (23239A>C), E58Q (23251G>C), V60E (23258T>A 23259C>G), L66M (23275C>A 23277C>G), A67G (23279C>G), G73A (23297G>C 23298G>C), Y78L (23311T>C 23312A>T), F93Y (23357T>A), W123S (23447G>C), G126S (23455G>A), W130Y (23468G>A 23469G>T), N131S (23471A>G), F133Y (23477T>A), G136A (23486G>C 23487C>G), Y138F (23492A>T), C139G (23494T>G 23496T>C), R140A (23497C>G 23498G>C 23499C>T), I141V (23500A>G), C143M (23506T>A 23507G>T 23508T>G), V146P (23515G>C 23516T>C 23517C>G), G147S (23518G>A), P150_P151del (23527_23532delCCGCCG), N152G (23533A>G 23534A>G), T153E (23536A>G 23537C>A 23538C>A), D154V (23540A>T 23541C>A), Y156H (23545T>C), D157E (23550C>G), H159T (23554C>A 23555A>C), E160G (23558A>G 23559G>C), L162I (23563C>A 23565G>C), I163T (23567T>C 23568C>G), Q166L (23576A>T 23577G>C), T168G (23581A>G 23582C>G 23583G>C), E169V (23585A>T 23586G>C), I170D (23587A>G 23588T>A), N173K (23598C>G), T175Q (23602A>C 23603C>A 23604G>A), S179L (23614T>C 23615C>T), N182E (23623A>G 23625C>A), Q186A (23635C>G 23636A>C 23637G>A), F187A (23638T>G 23639T>C 23640C>A), Y188W (23642A>G 23643C>G), D189E (23646C>G), T191L (23650A>C 23651C>T 23652T>G), R193K (23656C>A 23657G>A 23658C>G), Q194R (23660A>G 23661G>C), V195Q (23662G>C 23663T>A 23664C>G), N196D (23665A>G), D199G (23675A>G 23676C>T), E200D (23679G>T), L202I (23683C>A 23685T>C), P203del (23686_23688delCCG), W204G (23689T>G), A205L (23692G>C 23693C>T), Q207del (23698_23700delCAG), M208I (23703G>T), E209D (23706G>C), V212T (23713G>A 23714T>C 23715C>G), T215del (23722_23724delACC), E216V (23726A>T), H217L (23729A>T), N218D (23731A>G), T219N (23735C>A 23736A>T), L220P (23738T>C), L221Y (23740C>T 23741T>A), P222L (23744C>T), P223T (23746C>A 23748G>C), L226del (23755_23757delCTC), I229E (23764A>G 23765T>A 23766C>G), R230_R231insFK (23769_23770insTTCAAA), Q232K (23773C>A), F233Y (23777T>A 23778C>T), K234Q (23779A>C), G235D (23783G>A 23784G>C), R238K (23791C>A 23792G>A 23793C>G), G242A (23804G>C), E251T (23830G>A 23831A>C 23832G>C), D256S (23845G>T 23846A>C 23847T>C), A257G (23849C>G 23850G>C), T259S (23854A>T 23856G>T), Q261E (23860C>G), T262Q (23863A>C 23864C>A 23865A>G), R265V (23872C>G 23873G>T 23874G>C), D266E (23877T>G), A267H (23878G>C 23879C>A), D268A (23882A>C), V270A (23888T>C 23889C>G), R271V (23890C>G 23891G>T), A275E (23903C>A 23904C>A), A279R (23914G>C 23915C>G 23916G>A), M280I (23919G>C), N288K (23943C>G), L294V (23959C>G 23961T>C), D295E (23964C>G), I296L (23965A>C), R297G (23968C>G 23970G>C), K298A (23971A>G 23972A>C), H300D (23977C>G), A301Y (23980G>T 23981C>A), G302D (23984G>A 23985A>C), W307L (23998T>C 23999G>T 24000G>T), Q309V (24004C>G 24005A>T), K312E (24013A>G 24015G>A), E314G (24020A>G), S315T (24022T>A), L317V (24028C>G), A318_P323del (24031_24048delGCCGAGCTGGTCGATCCC), D324E (24051C>G), L327I (24058C>A), P328G (24061C>G 24062C>G), A329_P333del (24064_24078delGCCGACGTCGACCCG), A336E (24086C>A 24087C>A), G337R (24088G>C 24090T>C), R338H (24092G>A), R338_P339insDK (24093_24094insGACAAG), R340K (24097C>A 24098G>A 24099A>G), V343I (24106G>A), Y344F (24110A>T), A350G (24128C>G 24129T>C), H351D (24130C>G), E353Q (24136G>C), Q354K (24139C>A 24141G>A), R356K (24145C>A 24146G>A), K357R (24148A>C 24149A>G 24150G>C), N359G (24154A>G 24155A>G 24156C>G), A362V (24164C>T), V363T (24166G>A 24167T>C), K364E (24169A>G 24171G>A), L369I (24184C>A), G371A (24191G>C), D374A (24200A>C), H375E (24202C>G 24204C>G), S378R (24211T>C 24212C>G 24213A>G), A381E (24221C>A 24222G>A), M382R (24223A>C 24224T>G 24225G>C), E385N (24232G>A 24234G>C), G386D (24236G>A 24237G>C), P388V (24241C>G 24242C>T 24243G>C), V390L (24247G>T), T393S (24256A>T 24258T>C), R395K (24262C>A 24263G>A 24264A>G), G397Q (24268G>C 24269G>A 24270T>G), L399A (24274C>G 24275T>C), I400V (24277A>G 24279C>T), Q401R (24281A>G 24282G>C), S405G (24292T>G 24293C>G 24294G>C), D409E (24306C>G), K415Q (24322A>C 24324G>A), Q417E (24328C>G), A420V (24338C>T), A423S (24346G>A 24347C>G 24348G>C), L428I (24361C>A 24363G>C), F429Y (24365T>A), H431N (24370C>A)								
portal protein (YP_919077.1)	11	196	22.1%	815.0	79.5%	144 (100%)	110 (76.4%)	0/0/0/0	0
	H14N (24466C>A), L18M (24478C>A 24480C>G), E21S (24487G>T 24488A>C 24489G>T), D23A (24494A>C), L24V (24496C>G), A25S (24499G>T), S30T (24515G>C 24516T>G), I33V (24523A>G 24525C>G), P34S (24526C>T 24528C>G), H36R (24533A>G 24534C>G), Q39R (24542A>G), H41L (24548A>T), N42D (24550A>G), V45I (24559G>A 24561G>C), N46K (24564C>G), I68V (24628A>G), S72A (24640T>G 24642G>A), Y74W (24647A>G 24648C>G), A123T (24793G>A), R127E (24805C>G 24806G>A 24807C>G), L144I (24856C>A), M146I (24864G>C), L157F (24895C>T 24897T>C), Y159H (24901T>C 24903T>C), V165I (24919G>A 24921T>C), V167R (24925G>C 24926T>G 24927C>G), R169K (24931C>A 24932G>A 24933A>G), N172S (24941A>G 24942C>T), Y185F (24980A>T), G188A (24989G>C), A191G (24998C>G), I193V (25003A>G)								
XkdF-like putative serine protease domain-containing protein (YP_919079.1)	304	346	9.6%	54.0	18.6%	43 (95.6%)	11 (24.4%)	2/0/0/0	0
	D307E (27510T>G), G309E (27515G>A 27516C>G), G310T (27517G>A 27518G>C), S311E (27520T>G 27521C>A), D316E (27537C>G), T317A (27538A>G), T318A (27541A>G), M319E (27544A>G 27545T>A), S320K (27548G>A 27549C>G), N321P (27550A>C 27551A>C 27552C>T), D322A (27554A>C 27555C>G), N323W (27556A>T 27557A>G 27558C>G), G324A (27560G>C 27561C>G), D325E (27564C>G), D325_D326insKM (27564_27565insAAAATG), D326E (27567C>G), G327E (27569G>A 27570A>G), S328R (27571A>C 27573C>G), G329L (27574G>C 27575G>T 27576C>G), G331R (27580G>C), G332I (27583G>A 27584G>T 27585G>C), V333A (27587T>C), P334A (27589C>G), D335E (27594C>G), T337A (27598A>G), L338D (27601C>G 27602T>A), A339T (27604G>A 27606C>G), E340D (27609G>C), E341Q (27610G>C), N342T (27614A>C), A343E (27617C>A), E344K (27619G>A 27621A>G)								
hypothetical protein (YP_919080.1)	19	149	36.3%	602.0	67.6%	131 (100%)	87 (66.4%)	0/0/0/0	0
	G24D (28019G>A), A26T (28024G>A 28026C>G), E27T (28027G>A 28028A>C 28029A>G), V35A (28052T>C), V37I (28057G>A), E43T (28075G>A 28076A>C 28077G>T), K47N (28089G>C), G48E (28091G>A 28092C>G), Q50E (28096C>G), G53D (28106G>A 28107G>C), M54L (28108A>C), A61D (28130C>A 28131G>T), M65Q (28141A>C 28142T>A), E66D (28146G>C), H78D (28180C>G), E83D (28197G>C), E88A (28211A>C 28212G>T), N89A (28213A>G 28214A>C 28215C>G), A92N (28222G>A 28223C>A 28224G>C), E93D (28227G>C), S94D (28228T>G 28229C>A 28230T>C), S96Q (28234T>C 28235C>A), V97Y (28237G>T 28238T>A), K98S (28240A>T 28241A>C), N100D (28246A>G), K104R (28258A>C 28259A>G 28260G>C), S105Q (28261T>C 28262C>A 28263C>G), K113T (28286A>C), L117I (28297C>A), H121N (28309C>A 28311C>T), Y122L (28312T>C 28313A>T), G123S (28315G>T 28316G>C 28317G>C), P124D (28318C>G 28319C>A 28320A>C), F127W (28328T>G 28329C>G), D129E (28335C>G), Y130L (28336T>C 28337A>T), D133G (28346A>G), Q134E (28348C>G), I136T (28355T>C), G140A (28367G>C 28368C>G), L143V (28375C>G), G144A (28379G>C), M146I (28386G>C)								
hypothetical protein (YP_919092.1)	90	211	30.8%	591.0	70.8%	122 (100%)	80 (65.6%)	0/0/0/0	0
	Q93E (35722C>G 35724G>A), I94V (35725A>G), E95D (35730A>C), Q98E (35737C>G 35739G>A), D108E (35769C>G), I109V (35770A>G), Y112W (35780A>G 35781C>G), T113S (35782A>T 35784A>G), N118K (35799C>G), R122T (35809C>A 35810G>C 35811G>C), K123A (35812A>G 35813A>C), S124T (35815T>A), Q125H (35820G>C), L129V (35830C>G), P133I (35842C>A 35843C>T), A137D (35855C>A), G141P (35866G>C 35867G>C), T146L (35881A>C 35882C>T 35883A>C), I152L (35899A>C 35901C>G), S154A (35905A>G 35906G>C 35907T>G), L158R (35918T>G), A161T (35926G>A), V162L (35929G>C), E165T (35938G>A 35939A>C), V172E (35960T>A), Q173R (35963A>G), T175E (35968A>G 35969C>A), T181D (35986A>G 35987C>A 35988G>C), F184H (35995T>C 35996T>A), D186A (36002A>C), F190Y (36014T>A), A194S (36025G>A 36026C>G 36027G>C), L195E (36028C>G 36029T>A), S196T (36031T>A), E199K (36040G>A), F202Y (36050T>A 36051C>T), I203L (36052A>C), V206L (36061G>C 36063C>G), A207E (36065C>A), K208N (36069G>C), D209N (36070G>A)								
hypothetical protein (YP_919094.1)	260	819	15.7%	778.0	75.3%	144 (100%)	104 (72.2%)	0/0/0/0	0
	E263R (39015G>C 39016A>G), V266G (39025T>G 39026C>G), R268K (39030C>A 39031G>A 39032G>A), Q283R (39076A>G 39077G>C), T288V (39090A>G 39091C>T 39092C>G), I289V (39093A>G), V296I (39114G>A), E300A (39127A>C), V302I (39132G>A 39134G>C), I304T (39139T>C 39140C>G), E308A (39151A>C), L309I (39153C>A), R310E (39156C>G 39157G>A 39158G>A), H313D (39165C>G), R315V (39171C>G 39172G>T 39173C>G), T316V (39174A>G 39175C>T 39176C>G), E317R (39177G>C 39178A>G 39179G>C), Q318D (39180C>G 39182G>C), D321S (39189G>A 39190A>G), L327A (39207C>G 39208T>C), I751L (40479A>C), S755T (40492G>C 40493T>A), T765E (40521A>G 40522C>A 40523C>G), E766A (40525A>C), Y767F (40528A>T), Q768E (40530C>G), I769V (40533A>G), D771V (40540A>T), Y773F (40546A>T), T774D (40548A>G 40549C>A), K783T (40576A>C 40577G>A), A789E (40594C>A), D799S (40623G>A 40624A>G), I804L (40638A>C), A805T (40641G>A 40643C>G), A806G (40645C>G), G812R (40662G>A), A813T (40665G>A)								

*: Inserts / Deletes / Misaligned / Frameshifts

Detailed Results of Haloferax tailed virus 1 (NC_062739.1)

NGS details

Assembly

**Coverage length**	4743 (11 contig(s))
**Est. depth of coverage**	39.6
**Est. number of reads**	2212
**Ambiguities**	0

??coverage-details?? ??variant-analysis??

Assignment

**Type**	Haloferax tailed virus 1 (Taxonomy ID: 2507575)
**Reference Genome**	NC_062739.1 (Length: 38059bp)
**NT Identity (%)**	62.9046
**NT Quality**	0.45098

Alignment

**Alignment score**	2139 (NT) + 6715 (AA) = 8854
**Concordance (%)**	45.724
**Alignment method**	Local, heuristic, nucleotide (BLASTN)

Genome Region

Sequence starts at position null and ends at position null relative to the null reference sequence for null.

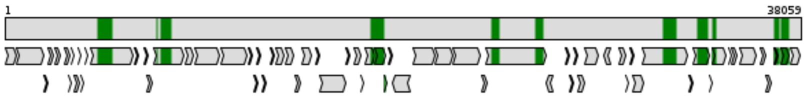

Alignment Detailed Statistics

	Begin	End	Coverage	Score	Concordance	Matches	Identities	I/D/M/F*	Stop Codons
**NT**	4448	37520	12.5%	2139.0	23.7%	4621 (97.2%)	2915 (61.3%)	13/122	
**CDS**									
M1M17_gp13	108	332	33.3%	817.0	52.8%	220 (97.8%)	111 (49.3%)	0/5/0/0	0
M1M17_gp17	48	286	42.2%	876.0	65.8%	196 (96.1%)	125 (61.3%)	1/7/0/0	0
M1M17_gp35	89	159	44.7%	281.0	62.0%	71 (100%)	39 (54.9%)	0/0/0/0	2
M1M17_gp36	1	160	97.0%	686.0	71.8%	152 (95.0%)	99 (61.9%)	0/8/0/0	0
M1M17_gp37	1	6	11.8%	26.0	72.2%	6 (100%)	4 (66.7%)	0/0/0/0	0
M1M17_gp44	84	907	26.3%	1153.0	66.3%	251 (99.6%)	162 (64.3%)	1/0/0/0	0

	Begin	End	Coverage	Score	Concordance	Matches	Identities	I/D/M/F*	Stop Codons
M1M17_gp56	316	550	32.2%	1002.0	68.3%	230 (97.9%)	144 (61.3%)	0/5/0/0	0
M1M17_gp58	86	242	58.6%	801.0	66.8%	156 (98.1%)	94 (59.1%)	2/1/0/0	0
M1M17_gp60	5	71	30.6%	374.0	76.2%	67 (100%)	50 (74.6%)	0/0/0/0	0
M1M17_gp66	1	76	100%	294.0	57.9%	74 (97.4%)	38 (50.0%)	0/2/0/0	0
M1M17_gp67	1	130	97.0%	405.0	50.6%	117 (90.7%)	71 (55.0%)	0/12/1/1	0
**Proteins**									
portal protein (YP_010355895.1)	108	332	33.3%	817.0	52.8%	220 (97.8%)	111 (49.3%)	0/5/0/0	0
	S111R (4460T>A), L114I (4467T>A 4469A>C), D116N (4473G>A), A117Q (4476G>C 4477C>A), H120N (4485C>A), L128M (4509T>A 4511A>G), G130E (4516G>A 4517C>A), L133T (4524C>A 4525T>C 4526C>G), W134Y (4528G>A 4529G>T), V139L (4542G>T 4544G>A), I142S (4552T>G), Q143V (4554C>G 4555A>T 4556A>T), I146R (4563A>C 4564T>G 4565T>G), E149G (4573A>G), K151S (4578A>T 4579A>C 4580A>T), E152H (4581G>C 4583A>C), A153V (4585C>T 4586A>T), L154E (4587C>G 4588T>A 4589C>G), P155L (4591C>T), A156V (4594C>T 4595A>T), L161T (4608C>A 4609T>C), M162L (4611A>C 4613G>A), E164L (4617G>C 4618A>T 4619G>C), S165T (4620T>A 4622A>G), A167E (4627C>A 4628T>A), Q168Y (4629C>T 4631A>T), K170D (4635A>G 4637G>C), V171I (4638G>A 4640G>T), H175E (4650C>G 4652C>A), R177_T178del (4656_4661delCGCACC), K179S (4662A>T 4663A>C 4664A>T), T180V (4665A>G 4666C>T 4667A>T), H181Q (4670C>G), G183S (4674G>A), Y184R (4677T>C 4678A>G 4679C>T), Q185E (4680C>G 4682A>G), Q187K (4686C>A), T188R (4689A>C 4690C>G), L189F (4692C>T), P190Q (4696C>A 4697G>A), A191P (4698G>C 4700A>C), D193E (4706C>A), W195G (4710T>G 4712G>C), I199L (4722A>C 4724C>T), A204G (4738C>G), E207K (4746G>A 4748A>G), V208T (4749G>A 4750T>C), E212D (4763A>C), R215Q (4771G>A 4772A>G), K217R (4776A>C 4777A>G 4778A>C), D218E (4781C>A), Q221E (4788C>G 4790A>G), A222T (4791G>A), F223Y (4795T>A 4796C>T), K224R (4798A>G 4799G>A), Q225A (4800C>G 4801A>C 4802A>C), E227A (4807A>C), A228Q (4809G>C 4810C>A 4811A>G), I230M (4817C>G), N231R (4818A>C 4819A>G), Q232E (4821C>G), I234T (4828T>C 4829T>C), L236R (4834T>G 4835C>A), H237L (4836C>T 4837A>T 4838C>G), Q241F (4848C>T 4849A>T 4850G>C), R242L (4851C>T 4852G>T), V246A (4864T>C), K248R (4869A>C 4870A>G 4871A>T), D250N (4875G>A), G251A (4879G>C 4880C>A), A252S (4881G>A 4882C>G 4883A>C), P253Q (4885C>A 4886C>A), V254L (4887G>C 4889T>A), R255N (4890C>A 4891G>A 4892G>C), D258E (4901C>G), V262I (4911G>A), T264N (4918C>A), I265R (4920A>C 4921T>G 4922C>A), D267R (4926G>A 4927A>G 4928C>A), R269_T271del (4932_4940delCGGACCACC), D272G (4942A>G 4943C>T), N274G (4947A>G 4948A>G 4949C>T), T275E (4950A>G 4951C>A), A276T (4953G>A), Y277Q (4956T>C 4958T>A), F278V (4959T>G 4961C>G), Q281P (4969A>C 4970A>G), D284E (4979C>A), V285A (4981T>C 4982G>C), E286N (4983G>A 4985A>C), T287F (4986A>T 4987C>T 4988G>C), L288M (4989C>A 4991C>G), A290G (4996C>G), H291P (4999A>C 5000C>T), N292S (5001A>T 5002A>C 5003C>G), F293V (5004T>G 5006C>T), Y295F (5011A>T 5012C>T), S296G (5013T>G 5014C>G 5015G>C), A297E (5017C>A 5018A>G), H299Q (5024C>A), M301R (5028A>C 5029T>G), M303T (5035T>C), N305M (5041A>T 5042C>G), T307A (5046A>G), L310I (5055T>A 5057G>C), L314I (5067C>A 5069C>T), A316L (5073G>C 5074C>T 5075G>A), G317L (5076G>C 5077G>T 5078C>G), V319E (5083T>A 5084C>G), A321S (5088G>A 5089C>G), K328A (5109A>G 5110A>C)								
P2 gpO-like scaffolding/protease protein (YP_010355899.1)	48	286	42.2%	876.0	65.8%	196 (96.1%)	125 (61.3%)	1/7/0/0	0
	E51D (7258G>C), T54E (7265A>G 7266C>A 7267G>A), E56Q (7271G>C), N58E (7277A>G 7279C>G), D60N (7283G>A), R62Q (7290G>A), V64L (7295G>C 7297G>T), P65D (7298C>G 7299C>A), E68T (7307G>A 7308A>C 7309A>G), D70E (7315C>A), Q122E (7469C>G), N131D (7496A>G), E132G (7500A>G 7501A>T), S136E (7511T>G 7512C>A), T138L (7517A>T 7518C>T 7519C>G), Q139D (7520C>G 7522A>T), P140L (7524C>T), D141E (7528C>G), F142Y (7530T>A 7531T>C), S144del (7535_7537delTCG), E146_F147del (7541_7546delGAGTTC), V152I (7559G>A 7561C>T), I154V (7565A>G 7567C>G), A155S (7568G>A 7569C>G 7570C>T), I158L (7577A>T 7579T>A), H159D (7580C>G 7582C>T), K160_P163del (7583_7594delAAAGGCGGCCCG), K165D (7598A>G 7600A>T), E167N (7604G>A 7606A>C), P168V (7607C>G 7608C>T 7609A>G), H169N (7610C>A 7612C>T), I173V (7622A>G 7624C>T), D178E (7639C>A), L182V (7649T>G), D183R (7652G>C 7653A>G 7654C>A), D185N (7658G>A), K187D (7664A>G 7666G>C), G191A (7677G>C), I193V (7682A>G 7684C>G), I194V (7685A>G), D196Q (7691G>C 7693C>G), R197T (7694C>A 7695G>C 7696A>C), N198D (7697A>G), D200G (7704A>G 7705C>A), A203Q (7712G>C 7713C>A 7714G>A), A205L (7718G>T 7719C>T 7720A>G), T207D (7724A>G 7725C>A), L209I (7730C>A 7732C>T), K210R (7734A>G 7735G>A), N215S (7748A>T 7749A>C), D216G (7752A>G 7753C>T), T218A (7757A>G 7759G>A), A219V (7761C>T 7762A>C), M228T (7788T>C 7789G>C), T230L (7793A>C 7794C>T 7795C>G), E231R (7796G>C 7797A>G), K233Q (7802A>C), A235T (7808G>A 7810G>A), A236P (7811G>C), A236_H237insN (7813_7814insAAC), H239Q (7822C>A), E241D (7828A>C), A245T (7838G>A 7840A>G), E248R (7847G>C 7848A>G 7849G>C), T250S (7854C>G 7855G>T), G253A (7863G>C 7864A>G), L254F (7867G>T), K261R (7887A>G 7888G>A), S262T (7889T>A 7891C>G), Q270A (7913C>G 7914A>C 7915G>T), G279V (7941G>T 7942T>A), K281Q (7946A>C), D282S (7949G>A 7950A>G), A283T (7952G>A)								
tail tube protein (YP_010355917.1)	89	159	44.7%	281.0	62.0%	71 (100%)	39 (54.9%)	0/0/0/0	2
	D94S (17493G>A 17494A>G 17495T>C), S95N (17497G>A 17498C>T), M98A (17505A>G 17506T>C 17507G>A), K99L (17508A>C 17509A>T), L102I (17517C>A 17519C>T), N104D (17523A>G), T106A (17529A>G 17531G>C), P107T (17532C>A 17534G>A), S110D (17541A>G 17542G>A), G112E (17548G>A 17549T>G), F113Y (17551T>A 17552C>T), T117V (17562A>G 17563C>T), A118V (17566C>T 17567C>T), V119A (17569T>C 17570G>C), E120D (17573G>T), N121K (17576C>A), L127F (17592C>T 17594C>T), L129I (17598C>A 17600T>C), D130L (17601G>C 17602A>T), A132K (17607G>A 17608C>A 17609A>G), T133S (17610A>T), Y140W (17632A>G 17633C>G), S141D (17634T>G 17635C>A 17636A>C), S148E (17655T>G 17656C>A 17657C>G), V149I (17658G>A 17660G>T), T150S (17662C>G), N151Q (17664A>C 17666C>A), L152I (17667C>A 17669C>T), A153E (17671C>A 17672A>G), S156N (17679T>A 17680C>A 17681G>C), G157* (17682G>T 17684T>A), V158A (17686T>C)								
hypothetical protein (YP_010355918.1)	1	160	97.0%	686.0	71.8%	152 (95.0%)	99 (61.9%)	0/8/0/0	0
	T2S (17697C>G), E3D (17701G>C), Q4_L11del (17702_17725delCAAGAACCCGTCCAAGACGAGTTA), E13K (17729G>A), E14D (17734A>C), D17E (17743C>G), I19M (17749C>G), L20Q (17750T>C 17751T>A), Q25E (17765C>G), E28Q (17774G>C), N29K (17779C>G), L30R (17781T>G 17782C>T), A31E (17784C>A), A35E (17796C>A 17797A>G), E38D (17806G>C), A46Q (17828G>C 17829C>A 17830C>A), V48T (17834G>A 17835T>C 17836C>G), Q52E (17846C>G), L55I (17855C>A), I56V (17858A>G 17860C>T), E58D (17866G>C), T60V (17870A>G 17871C>T), L63I (17879C>A), K64R (17882A>C 17883A>G 17884A>T), K74Q (17912A>C), I78M (17926T>G), S80A (17930T>G 17932G>C), I82L (17936A>C), H84R (17943A>G 17944C>A), I85L (17945A>C 17947C>T), Q86E (17948C>G 17950G>A), S87A (17951A>G 17952G>C 17953T>C), G88E (17955G>A 17956C>G), D89E (17959T>G), M92A (17966A>G 17967T>C 17968G>C), V98A (17985T>C 17986C>T), R101N (17993C>A 17994G>A 17995G>T), E113G (18030A>G 18031G>C), S114D (18032T>G 18033C>A 18034A>T), E118S (18044G>A 18045A>G 18046G>C), Q122A (18056C>G 18057A>C 18058A>T), A123V (18060C>T), E125K (18065G>A), S126E (18068T>G 18069C>A 18070C>G), L129I (18077T>A 18079G>T), A130Q (18080G>C 18081C>A 18082C>G), T134V (18092A>G 18093C>T 18094G>C), I136L (18098A>C 18100T>C), S137K (18102G>A 18103C>G), I140F (18110A>T), D141E (18115T>A), K147R (18131A>C 18132A>G 18133G>T), M151Q (18143A>C 18144T>A), D155E (18157T>G)								
hypothetical protein (YP_010355919.1)	1	6	11.8%	26.0	72.2%	6 (100%)	4 (66.7%)	0/0/0/0	0
	M1R (18157T>G)								
baseplate hub (YP_010355926.1)	84	907	26.3%	1153.0	66.3%	251 (99.6%)	162 (64.3%)	1/0/0/0	0

	Begin	End	Coverage	Score	Concordance	Matches	Identities	I/D/M/F*	Stop Codons
	D87E (23285C>A), N88Q (23286A>C 23288T>G), T91V (23295A>G 23296C>T 23297C>A), Y93F (23302A>T 23303C>T), E96N (23310G>A 23312A>C), R97A (23313C>G 23314G>C 23315C>T), R98A (23316C>G 23317G>C), E101D (23327A>T), Q102D (23328C>G 23330A>C), Q103D (23331C>G 23333A>C), D106E (23342T>A), V107Q (23343G>C 23344T>A 23345A>G), S114T (23364T>A), R121G (23385C>G), N122D (23388A>G 23390C>T), T123E (23391A>G 23392C>A 23393C>A), T125S (23397A>T 23399G>T), I133V (23421A>G 23423T>G), S134F (23424A>T 23425G>T 23426C>T), Q135E (23427C>G), A138E (23437C>A 23438C>A), I140V (23442A>G), Q142T (23448C>A 23449A>C 23450G>A), D144_T145insV (23456_23457insGTT), T145P (23457A>C 23459G>A), R148E (23466C>G 23467G>A 23468G>A), F149W (23470T>G 23471C>G), A151P (23475G>C 23477C>G), I154V (23484A>G 23486C>T), T155E (23487A>G 23488C>A), G157V (23494G>T), Q160V (23502C>G 23503A>T 23504A>T), R164Q (23515G>A 23516G>A), Y166F (23521A>T), D169E (23531C>A), N173V (23541A>G 23542A>T), A174V (23545C>T), F178L (23558C>A), T183G (23571A>G 23572C>G 23573C>T), D186A (23581A>C 23582C>A), G190N (23592G>A 23593G>A 23594C>T), D192S (23598G>A 23599A>G 23600T>C), L193R (23601T>C 23602T>G 23603G>A), Q198R (23616C>A 23617A>G), V203K (23631G>A 23632T>A 23633C>G), V209I (23649G>A 23651G>A), R216N (23671G>A 23672A>T), S804T (25434T>A), A806G (25441C>G 25442G>C), R807S (25443C>A 25445A>T), L810S (25452C>T 25453T>C 25454C>A), A812W (25458G>T 25459C>G 25460C>G), G817A (25474G>C 25475G>C), F819Q (25479T>C 25480T>A 25481C>A), Y820F (25483A>T 25484C>T), V833I (25521G>A), A836S (25530G>A 25531C>G), T837D (25533A>G 25534C>A 25535G>T), S845D (25557T>G 25558C>A 25559G>C), P846A (25560C>G 25562G>A), D847E (25565T>G), T848S (25567C>G 25568C>T), N849S (25569A>T 25570A>C 25571C>T), N853R (25581A>C 25582A>G 25583T>G), I854L (25584A>C 25586C>T), S855G (25587T>G 25588C>G 25589G>C), L856F (25590C>T 25592A>T), N857S (25593A>T 25594A>C), Y859I (25599T>A 25600A>T 25601T>C), S861T (25605T>A), S863T (25611T>A), T864D (25614A>G 25615C>A 25616G>T), Q868L (25627A>T 25628G>T), T869E (25629A>G 25630C>A 25631G>A), N872D (25638A>G 25640C>T), A873T (25641G>A 25643A>G), D877S (25653G>A 25654A>G), N878S (25656A>T 25657A>C 25658T>A), E880N (25662G>A 25664A>T), Y882F (25669A>T), I885P (25677A>C 25678T>C 25679T>C), V888I (25686G>A 25688T>C), L889F (25689C>T 25691T>C), G894A (25705G>C 25706C>A), V895T (25707G>A 25708T>C), T896A (25710A>G 25712A>G), S898V (25716T>G 25717C>T 25718G>C), P904A (25734C>G 25736C>A)								
MCM (YP_010355938.1)	316	550	32.2%	1002.0	68.3%	230 (97.9%)	144 (61.3%)	0/5/0/0	0
	N318H (31455A>C 31457T>C), E323D (31472A>C), K326V (31479A>G 31480A>T 31481G>C), N327D (31482A>G), I329L (31488A>C), G332S (31497G>A), T334V (31503A>G 31504C>T 31505G>C), G336_I338del (31509_31517delGGCGAGATC), D340E (31523C>A), R341Q (31525G>A 31526T>G), I342V (31527A>G), L344R (31534T>G), G347A (31543G>C 31544A>G), M350L (31551A>T), M354V (31563A>G 31565G>C), V356A (31570T>C 31571G>C), D357K (31572G>A 31574C>A), K358S (31575A>T 31576A>C 31577A>C), A359P (31578G>C), A363T (31590G>A 31592A>C), L364T (31593C>A 31594T>C), T370Q (31611A>C 31612C>A 31613A>G), M372L (31617A>C 31619G>T), S375G (31626T>G 31627C>G), Q382S (31647C>T 31648A>C), I383L (31650A>C 31652A>C), L384M (31653C>A 31655T>G), E385D (31658A>C), M388A (31665A>G 31666T>C), L390Y (31671C>T 31672T>A), T391S (31674A>T), R393K (31680C>A 31681G>A 31682C>G), R396T (31689C>A 31690G>C), V402A (31708T>C 31709C>T), T405A (31716A>G 31718G>C), L407V (31722C>G 31724T>C), S410A (31731T>G 31733T>A), Q413R (31741A>G 31742A>C), Q420E (31761C>G), T422S (31767A>T 31769G>A), K430Q (31791A>C 31793G>A), T435V (31806A>G 31807C>T), I438V (31815A>G 31817C>T), G446P (31839G>C 31840G>C 31841C>G), A448V (31846C>T 31847A>G), L449V (31848C>G 31850C>T), T458R (31875A>C 31876C>G), V461L (31884G>C 31886A>G), D462S (31887G>T 31888A>C 31889C>A), E469T (31908G>A 31909A>C 31910A>G), T470A (31911A>G 31913C>G), R471E (31914C>G 31915G>A 31916A>G), Q473A (31920C>G 31921A>C 31922A>G), S474C (31924C>G 31925C>T), S477A (31932T>G 31934A>G), T485E (31956A>G 31957C>A 31958G>A), N486H (31959A>C), I490V (31971A>G 31973C>T), G491D (31975G>A 31976C>T), P492del (31977_31979delCCG), I494Q (31983A>C 31984T>A), E495K (31986G>A 31988A>G), S496T (31989T>A), Y497del (31992_31994delTAC), V498I (31995G>A 31997T>C), L503V (32010C>G 32012G>T), D505P (32016G>C 32017A>C 32018C>G), L507M (32022C>A 32024C>G), S509T (32029G>C 32030C>A), M511L (32034A>C 32036G>T), A513T (32040G>A 32042G>A), V514I (32043G>A 32045G>T), L516V (32049C>G 32051C>G), K518R (32055A>C 32056A>G 32057A>C), E520R (32061G>C 32062A>G 32063A>C), D522N (32067G>A), E524D (32075G>C), R525D (32076C>G 32077G>A 32078A>T), G527A (32083G>C), E528A (32086A>C), I529K (32089T>A 32090C>G), I533M (32102C>G), I534L (32103A>T 32105C>G), D535N (32106G>A), T539S (32118A>T), N542A (32127A>G 32128A>C 32129C>G), D544E (32135T>A), V546E (32140T>A 32141T>A)								
phosphoadenosine phosphosulfate reductase (YP_010355940.1)	86	242	58.6%	801.0	66.8%	156 (98.1%)	94 (59.1%)	2/1/0/0	0
	Q89E (33148C>G), Q91H (33156A>C), A97S (33172G>T), L98R (33175C>A 33176T>G 33177T>A), S100G (33181T>G 33182C>G 33183G>C), T101I (33185C>T 33186A>C), A102M (33187G>A 33188C>T 33189A>G), K105C (33196A>T 33197A>G 33198A>T), L111I (33214T>A 33216G>A), G112Q (33217G>C 33218G>A), T116S (33230C>G 33231G>C), L117T (33232C>A 33233T>C 33234C>G), A118V (33236C>T 33237A>C), H119del (33238_33240delCAT), P120S (33241C>T 33243G>C), K121G (33244A>G 33245A>G 33246A>C), E122D (33249G>C), V124H (33253G>C 33254T>A), S127T (33262T>A), S131A (33274A>G 33275G>C 33276T>G), A132L (33277G>C 33278C>T 33279T>C), K135D (33286A>G 33288G>C), S138M (33296G>T 33297T>G), L139R (33299T>G 33300C>A), N140T (33302A>C), P143A (33310C>G 33312G>A), I144T (33314T>C 33315A>T), D145A (33317A>C), D145_D146insEH (33318_33319insGAGCAC), A147G (33323C>G 33324T>G), P148G (33325C>G 33326C>G), K151T (33335A>C), V153H (33340G>C 33341T>A 33342G>C), S154A (33343T>G 33345G>T), D160S (33361G>A 33362A>G), D162G (33368A>G), K163E (33370A>G), K165Q (33376A>C 33378A>G), S166R (33379T>C 33380C>G), D169A (33389A>C 33390T>C), D170A (33392A>C), R171M (33395G>T), I173L (33400A>C 33402C>G), K175R (33406A>C 33407A>G), E177P (33412G>C 33413A>C 33414A>G), I178L (33415A>C), N195S (33467A>G 33468C>T), K199V (33478A>G 33479A>T 33480G>C), F205A (33496T>G 33497T>C), A207H (33502G>C 33503C>A 33504A>C), D208G (33506A>G), Y209E (33508T>G 33510C>G), E212D (33519G>C), E215R (33526G>C 33527A>G 33528A>C), D216E (33531C>G), D219Q (33538G>C 33540T>G), T220S (33541A>T 33543T>C), K226E (33559A>G 33561A>G), T227K (33563C>A), T229R (33568A>C 33569C>G 33570G>A), F235L (33586T>C 33588C>G), K238P (33595A>C 33596A>C 33597A>C), Q240R (33602A>G)								
DNA methylase (YP_010355942.1)	5	71	30.6%	374.0	76.2%	67 (100%)	50 (74.6%)	0/0/0/0	0
	A11C (33848G>T 33849C>G), A18Q (33869G>C 33870C>A 33871C>G), E21A (33879A>C 33880A>C), E22D (33883A>C), E24D (33889A>C), D27T (33896G>A 33897A>C 33898C>G), E32D (33913G>C), E35A (33921A>C), K36R (33923A>C 33924A>G 33925G>C), D38E (33931C>G), I41R (33938A>C 33939T>G 33940A>C), A43V (33945C>T), Q55H (33982A>C), E56D (33985G>C), T67E (34016A>G 34017C>A 34018A>G), Q68E (34019C>G 34021A>G)								
hypothetical protein (YP_010355948.1)	1	76	100%	294.0	57.9%	74 (97.4%)	38 (50.0%)	0/2/0/0	0
	D6Q (36819G>C 36821C>G), Q8R (36825C>A 36826A>G 36827A>G), D9A (36829A>C 36830C>T), E10K (36831G>A 36833G>A), A12E (36838C>A 36839C>G), E13S (36840G>T 36841A>C), D20E (36863C>G), H22V (36867C>G 36868A>T), Q23H (36872A>C), R24Q (36874G>A 36875C>A), F26L (36879T>C), E31D (36896A>C), L32I (36897C>A 36899C>T), D33P (36900G>C 36901A>C), D34E (36905C>A), A35S (36906G>T 36908T>A), T36E (36909A>G 36910C>A 36911C>A), Q37_T38del (36912_36917delCAGACG), L42I (36927C>A), T43R (36930A>C 36931C>G), N45H (36936A>C), V46L (36939G>T), S47T (36943G>C 36944T>G), L49V (36948C>G 36950C>G), M50R (36951A>C 36952T>G 36953G>C), T51N (36955C>A), W53Y (36961G>A 36962G>C), K54R (36963A>C 36964A>G 36965A>C), E55D (36968G>C), T56L (36969A>C 36970C>T 36971A>C), R57K (36972C>A 36973G>A), I63V (36990A>G 36992C>G), K64R (36993A>C 36994A>G 36995A>C), L66M (36999C>A), K68E (37005A>G), E70D (37013G>C)								
GIY-YIG nuclease (YP_010355949.1)	1	130	97.0%	405.0	50.6%	117 (90.7%)	71 (55.0%)	0/12/1/1	0
	Q5A (37143C>G 37144A>C 37145G>A), M6I (37148G>A), H7T (37149C>A 37150A>C), G8E (37153G>A 37154T>A), R9T (37155C>A 37156G>C), E12T (37164G>A 37165A>C), K13R (37167A>C 37168A>G 37169A>C), A19_E30del (37185_37222delGCGCACTCACCTGGCACCTACGCCATTGCAGTCGAGAC), D34T (37230G>A 37231A>C), S37G (37239A>G), W42F (37255G>T 37256G>T), Q44E (37260C>G), E45V (37264A>T 37265A>C), V46G (37267T>G 37268A>T), P49C (37275C>T 37276C>G 37277A>C), L50P (37279T>C 37280T>G), S51P (37281T>C), K53G (37287A>G 37288A>G 37289A>C), F55L (37293T>C 37295T>G), S59T (37305T>A 37307G>C), S61D (37311A>G 37312G>A 37313T>C), K62R (37314A>C 37315A>G 37316G>C), C64A (37320T>G 37321G>C), K70T (37339A>C 37340G>C), N74T (37351A>C 37352C>G), Q77M (37359C>A 37360A>T), A82G (37375C>G 37376G>C), S83K (37378G>A 37379T>G), K86Q (37386A>C 37388G>A), L88T (37392T>A 37393T>C), R91E (37401C>G 37402G>A 37403C>A), V92A (37405T>C), A94N (37410G>A 37411C>A), D97G (37420A>G), S104E (37440T>G 37441C>A), D105S (37443G>T 37444A>C), S106D (37446A>G 37447G>A 37448C>T), R109D (37455C>G 37456G>A 37457T>C), D110A (37459A>C 37460T>C), S113N (37467T>A 37468C>A 37469G>C), K114R (37470A>C 37471A>G 37472A>C), L116V (37476T>G 37478G>T), G120R (37488G>C 37490T>G), E121Q (37491G>C), V124T (37500G>A 37501T>C 37502T>G), A125V (37504C>T), S127C (37510C>G)								

*: Inserts / Deletes / Misaligned / Frameshifts
